# 17^th^ EUNOS Congress: Milan, Italy, 4–6 June, 2026Editors’ Introduction

**DOI:** 10.1080/01658107.2026.2651025

**Published:** 2026-05-29

*Neuro-Ophthalmology* is pleased to once again publish as a supplement the abstracts of the European Neuro-Ophthalmology Society (EUNOS) Meeting. The 17h EUNOS meeting will be held from June 4 to June 6, 2024 in Milan, Italy. *Neuro-Ophthalmology* is proud to be the official journal of EUNOS.

*Neuro-Ophthalmology* is international in scope. With online submission and review, *Neuro-Ophthalmology* can rapidly process manuscripts from all corners of the globe. Manuscripts are published online within a matter of weeks of acceptance.

*Neuro-Ophthalmology* publishes six issues per year. All dues paying members of EUNOS receive online access to the journal as well as a print copy. *Neuro-Ophthalmology* publishes original papers related to clinical and basic science topics, case reports, editorials, letters to the editor, reviews, selected abstracts, society tidings, and book reviews. Presently, *Neuro-Ophthalmology* has 45,000 downloads/views per year which continue to increase.

Manuscripts may be submitted online to *Neuro-Ophthalmology*. We invite and encourage all participants at the 17th EUNOS Meeting to submit their manuscripts to *Neuro-Ophthalmology*. On behalf of the entire editorial board and production team, we wish all attendees an enjoyable and productive conference in Milan.

*Dr. Walter M. Jay*

*drwalterjay@hotmail.com*

*Dr. Dan Milea*

*mileadk@yahoo.com*

*Editors-in-ChiefNeuro-Ophthalmology*

## EUNOS President’s Introduction June, 2024

Dear Colleagues,

Welcome to the 17th biennial meeting of the European Neuro-Ophthalmology Society (EUNOS), which will take place in the elegant city of Milan from June 4 to 6, 2026. We are grateful to Aki Kawasaki and Piero Barboni for organising what promises to be an inspiring conference with a touch of Italianità.

Following the success of our outstanding 2024 EUNOS meeting in Rotterdam, the Milan conference will again bring together clinicians, researchers, and trainees from across Europe and beyond to share the latest advances in neuro-ophthalmology. The scientific programme will highlight developments in diagnostics, therapies, and technology, complemented by hands-on workshops. The sessions provide a platform for inspiring discussions among established experts and emerging investigators as they present their work.

The high quality and breadth of the abstracts submitted for this meeting and published in this supplement reflect the vitality and continuing growth of our specialty. They showcase innovative research and clinical insights that will shape the future of neuro-ophthalmology.

We hope you enjoy reading these contributions and look forward to welcoming the neuro-ophthalmology community to Milan.

*Konrad P. Weber*

*President, EUNOS*

**Chair of the Organizing Committee’s Welcome to Milan**

Dear Colleagues and Friends,

We extend to you a warm and enthousiastic welcome to the bienniel meeting of the European Neuro-Ophthalmologic Society (EUNOS), this year located in the cosmopolitan city of Milan from June 4-6.

This 17th EUNOS meeting aims to inform and update you in current neuro-ophthalmologic diagnosis, research and treatment. Session and keynote topics include optic nerve ischemic injury and regeneration, genetics in optic nerve disorders, hereditary cerebellar ataxia, pediatric neuro-ophthalmology, visual hallucinations and other perceptual disorders, deep learning and ocular imaging, augmented reality in perimetric testing, pathophysiology of migraine manifestations, and management of low vision.

EUNOS 2026 also invites you to test your clinical acumen in joining us for a session of challenging brain-buster cases!

Enjoy the many diverse topics presented in the spacious poster area and engage in discussion with the authors. This is where new ideas and new collaborations can begin!

After each day of learning and participation at EUNOS 2026, we encourage you to meet and connect with colleagues and friends over a drink and hors d’oeuvres at the evening social reception. The EUNOS gala dinner will be hosted at the famous Leonardo Da Vinci Museum of Science and Technology.

We sincerely hope that EUNOS 2026 in Milan will excite you and invigorate you to explore the many facets that form the field of neuro-ophthalmology.

*Aki Kawasaki*

*University of Lausanne*

*Piero Barboni*

*IRCCS San Raffaele*

## TABLE OF CONTENTS

DISORDERS OF THE ANTERIOR VISUAL PATHWAY (OPTIC NERVE AND CHIASM)

1, 2, 3, 5, 7, 10, 12, 15, 22, 23, 24, 25, 26, 30, 31, 34, 36, 37, 38, 39, 40, 42, 43, 44, 46, 47, 48, 49, 51, 52, 54, 55, 58, 67, 68, 69, 70, 71, 73, 74, 81, 82, 84, 89, 92, 93, 95, 100, 101, 102, 104, 108, 109, 113, 115, 118, 119, 120, 121, 124, 125, 126, 127, 128, 129, 131, 132, 134, 135, 136, 137, 138, 139, 140, 141, 146, 147, 151, 153, 154, 155, 156, 157, 160, 161, 162, 163, 164, 165, 168, 169, 171

DISORDERS OF RETROCHIASMAL PATHWAYS AND VISUAL PROCESSING

9, 18, 123, 142

INTRACRANIAL HYPERTENSION

13, 14, 21, 28, 45, 59, 60, 62, 88, 99, 105, 106, 110, 116, 133, 148, 149, 159, 167, 170

OCULAR MOTILITY and EYELIDS

4, 11, 16, 19, 20, 41, 66, 77, 87, 96, 107, 111, 112, 117, 130, 144, 158

NEURODEGENERATIVE AND SYSTEMIC DISEASES

32, 50, 61, 63, 64, 65, 94, 98,

ARTIFICIAL INTELLIGENCE

6, 17, 35, 90,

MISCELLANEOUS

8, 27, 29, 33, 53, 56, 72, 75, 76, 78, 79, 83, 85, 86, 91, 97, 103, 114, 143, 145, 150, 152, 166

## DISORDERS OF THE ANTERIOR VISUAL PATHWAY (OPTIC NERVE AND CHIASM)

1. **How normal is a normal disc in retrobulbar optic neuritis? OCT reveals focal thickening in the acute stage of the disease**

Ana Banc,^1,2^ Mehdi Ounissi,^1,2^ Aurore Sajust De Bergues De Escalup,^3^ Cédric Lamirel,^1^ Caroline Papeix,^4^ Augustin Lecler,^3,5^ Romain Deschamps,^4^ Dan Milea^1,2,6,7,8^

^1^Department of Neuro-Ophthalmology, Adolphe de Rothschild Foundation Hospital, Paris, France; ^2^Rothschild Computational and Visual Neurosciences Laboratory, Adolphe de Rothschild Foundation Hospital, Paris, France; ^3^Department of Neuroradiology, Adolphe de Rothschild Foundation Hospital, Paris, France; ^4^Department of Neurology, Adolphe de Rothschild Foundation Hospital, Paris, France; ^5^Paris Cité University, Paris, France; ^6^Singapore Eye Research Institute, Singapore National Eye Center, Singapore; ^7^Duke-NUS Medical School, Singapore; ^8^Copenhagen University, Denmark

**Introduction**: Acute retrobulbar optic neuritis (ARON) is a clinical diagnosis, associated by definition with a normal-appearing optic disc, which can make its objective early diagnosis challenging. We investigated whether optical coherence tomography (OCT) could detect subtle retinal changes in ophthalmoscopically normal ARON.

**Methods**: We conducted a case-control study including 58 prospectively enrolled patients with a first episode of strictly unilateral ARON and confirmed normal optic disc appearance, and 58 age- and sex-matched healthy controls. Spectral-domain OCT (Spectralis, Heidelberg Engineering, Heidelberg, Germany) performed within 4 weeks after visual loss measured global and sectoral peripapillary retinal nerve fiber layer (pRNFL) thickness, as well as the thickness of each macular layer. Intra- and interindividual interocular differences were calculated using linear regression analysis. To cross-validate the results, we developed a vision transformer pRNFL segmentation model, followed by a binary classifier artificial intelligence (AI) model to detect ARON-related pRNFL changes by comparing per-degree interocular thickness differences. Averaging across 10° intervals generated 36 pRNFL metrics for each eye.

**Results**: OCT was obtained on average 11 ± 7 days after visual loss, disclosing significant sectoral pRNFL thickening compared with the contralateral eyes, predominantly in the nasal-superior and nasal-inferior sectors. Affected eyes were discriminated from normal eyes with an area under the curve (AUC) of 0.84 (95%CI 0.78–0.91) at a 19 µm cutoff. These structural differences were consistent across causes of ARON and independent of the time elapsed after symptom onset. The AI model further improved detection of ARON, achieving an AUC of 0.89 (0.84–0.94) and identifying the key focally thickened pRNFL regions, in the nasal-superior and nasal-inferior sectors.

**Discussion**: Despite normal fundus appearance, conventional and AI-driven OCT analyses of eyes with ARON are able to detect significant focal pRNFL thickening in the nasal-superior and nasal-inferior sectors. This structural signature reliably distinguishes affected eyes from contralateral healthy eyes, supporting the use of OCT as a sensitive objective tool for early diagnosis of an initial episode of ARON.

**Disclosures**: All authors declare that they have no competing interests or financial relationships that could influence the work reported in this manuscript.

**Keywords**: retrobulbar optic neuritis; acute optic neuritis; multiple sclerosis; optical coherence tomography; retinal imaging; artificial intelligence; deep learning

2. **A case of Leber Hereditary Optic Neuropathy seemingly precipitated by an electric shock**

Abhijeet Yadav

Buckinghamshire Healthcare NHS Trust, Buckingham, UK

**Introduction:** Optic neuropathy secondary to mitochondrial mutations often has a second environmental “hit” that is believed to precipitate the neuropathy. These can be toxin exposure, metabolic conditions or nutrient deficiencies to name a few. I describe a case of Leber hereditary optic neuropathy (LHON) with a homoplasmic pathogenic mutation in MT-DN3 (mitochondrially encoded NADH:ubiquinone oxidoreductase core subunit 3), possibly precipitated by an electric shock two months prior to presentation.

**Case Report:** A 26 year old lady presented in March 2017 after noticing poor vision in the right eye when she happened to cover her left eye. She had suffered from an electrocution two months prior to her left hand from a mulled wine machine (220-240 V), that knocked her back and left her dazed although she did not lose consciousness. She did not notice any visual decline immediately after the electric shock and she did not have any lasting physical injuries. Vision was hand movements only in the right eye at presentation in March and the only finding was mild pallor of the optic disc with a normal left eye exam and function. She subsequently developed visual decline in the left eye in May 2017 with initial telangiectasia over the disc and later optic disc pallor over many months. By the end of the year, vision was around 3/60 in both eyes.

She had extensive investigations including infectious, inflammatory (blood and CSF studies), genetic (LHON 3 common mutations and OPA panel) and radiologic testing that did not find a definitive cause. She was followed up annually until she was seen by the author in 2024. Further genetic testing in the form of mitochondrial genome sequencing was then requested which revealed the homoplasmic MT-DN3 mutation. She had no family history of genetic eye disease. She is currently awaiting consultation with the local genetics team as well as consideration regarding treatment with Idebenone.

**Discussion:** Electric shock related ocular injuries typically follow high voltage injuries significant enough to also cause physical manifestations such as skin burns, with the most commonly involved ocular structures being the lens and retina. Electric shock related optic neuropathies have been described although again these have typically been associated with significant physical extraocular injuries. The electric shock injury in this case may well be incidental but conversely may have acted as a precipitant of neuropathy in genetically susceptible optic nerves.

**Disclosures:** All authors declare that they have no competing interests or financial relationships that could influence the work reported in this manuscript.

3. **Optic Perineuritis in a 17-Year-Old Patient: A diagnostic challenge**

Adriana Molino^1^, Alessia Bargagli^1,2^, Francesco Sicurelli^1^, Alessandra Rufa^1,2^, Nicola De Stefano^1^

^1^Neurogical Unit, Department of Medicine, Surgery and Neuroscience, University of Siena, Siena, Italy; ^2^Eye-tracking & Visual Application EVALab, Department of Medicine, Surgery and Neuroscience, University of Siena, Siena, Italy

**Introduction**: Optic perineuritis (OPN) is an inflammatory process primarily involving the optic nerve sheath, presenting with gradual decline of visual function, eye pain, and/or pain on eye movements, disc edema and various features of optic nerve dysfunction, including visual field defects. In most cases the disease is isolated, however, it can also be associated with a wide range of underlying systemic diseases. OPN is clinically distinct from optic neuritis, and the diagnosis is radiologically confirmed based on the finding of circumferential perineural enhancement of the optic nerve sheath on magnetic resonance imaging (MRI).^1^

**Case Report**: A 17-year-old male presented with a 3-month history of LE visual loss and mild ocular pain during eye movements or physical exercises. The onset was characterized by acute visual loss in the left eye, predominantly affecting the inferior visual field, associated with marked photophobia and retro-orbital pain. During the acute phase, the patient experienced severe headaches lasting several days.

To investigate the cause, he underwent three serial brain and orbital MRI examinations, optical coherence tomography (OCT), visual field testing, pattern visual evoked potentials (PEV), cerebrospinal fluid analysis, and extensive autoimmune and infectious screening including ANA, ENA, ANCA, anti-AQP4, and anti-MOG antibodies. Genetic testing for hereditary optic neuropathies was also performed, and multidisciplinary neuro-ophthalmology assessments were conducted to correlate clinical findings with imaging and functional evaluations.

MRI demonstrated subtle FLAIR hyperintensity along the intracanalicular and retrobulbar segments of the left optic nerve with mild perineural contrast enhancement, suggestive of perineuritis. No more lesions were noted in the brain. OCT showed mild bilateral retinal nerve fiber layer thickening. Visual field testing confirmed a stable inferior hemifield defect, with partial papillo-macular bundle involvement. VEPs were within normal limits. Cerebrospinal fluid analysis was unremarkable.

The patient received high-dose intravenous methylprednisolone followed by a tapering regimen with minimal visual improvement. Follow-up MRI demonstrated partial regression of optic nerve sheath hyperintensity and contrast enhancement, consistent with partial resolution of the inflammatory process. The right eye remained unaffected throughout follow-up.

**Discussion and Conclusion**: This case highlights the diagnostic challenge of optic perineuritis, which can mimic demyelinating optic neuritis while lacking typical autoantibody or cerebrospinal fluid abnormalities. MRI is crucial for detecting optic nerve sheath inflammation and monitoring disease progression. The response to high dose corticosteroids may be less prominent in OPN than ON, emphasizing the need for careful multidisciplinary follow-up and consideration of alternative immunomodulatory strategies. Early recognition and comprehensive assessment are essential to prevent permanent visual sequelae in this rare but potentially sight-threatening condition.

1.Saitakis
G, Chwalisz
BK.
Optic perineuritis. Curr Opin Ophthalmol. 2022 Nov
1;33(6):519–524. doi: 10.1097/ICU.0000000000000900. Epub 2022 Aug 26. PMID: 36066374.36066374


5. **When NAION is not NAION: Bilateral optic disc edema during semaglutide therapy**

Agata Anna Wykrota

Department of Ophthalmology, San Maurizio Hospital, Bolzano, Italy

**Introduction**: Glucagon-like peptide-1 receptor agonists (GLP-1 RAs), particularly semaglutide, are increasingly prescribed for type 2 diabetes mellitus and obesity because of their favorable metabolic and cardiovascular profiles. With expanding use, attention has turned to potential neuro-ophthalmic adverse events. Observational studies, pharmacovigilance analyses, and isolated case reports have suggested a possible association between semaglutide and optic nerve disorders, including non-arteritic anterior ischemic optic neuropathy (NAION) and optic disc edema. Distinguishing true ischemic optic neuropathy from reversible disc edema remains clinically challenging and has important diagnostic and prognostic implications.

**Case Report** A 60-year-old woman presented with a one-week history of a shadow in the visual field of the right eye. Her medical history included Brugada syndrome with pacemaker implantation, hypothyroidism, and type 2 diabetes mellitus. Current medications were levothyroxine, bisoprolol, and oral semaglutide. Ophthalmologic examination revealed uncorrected visual acuity of 20/20 in both eyes, normal intraocular pressure, no relative afferent pupillary defect (RAPD), and full ocular motility. Color desaturation was present in the right eye. Fundoscopy demonstrated bilateral optic disc blurring and hyperemia with otherwise normal retinal findings. Humphrey visual field 30-2 testing showed an inferonasal defect in the right eye; the left eye was unremarkable. Optical coherence tomography (OCT) revealed retinal nerve fiber layer (RNFL) thickening that was nearly circumferential in the right eye and predominantly superior in the left eye, with normal macular architecture. Fluorescein angiography showed late papillary leakage in the right eye only. A comprehensive neurological work-up, including brain and orbital magnetic resonance imaging, autoimmune antibody testing, and lumbar puncture, was unrevealing; cerebrospinal fluid opening pressure was 15–17 mmHg. Inflammatory markers and metabolic parameters were unremarkable. At follow-up, visual acuity remained normal, color desaturation and the visual field defect in the right eye persisted, and OCT demonstrated complete resolution of RNFL thickening in both eyes without evidence of optic nerve atrophy.

**Discussion** Although the initial visual field defect raised suspicion for NAION, several features argued against a definitive ischemic optic neuropathy: preserved visual acuity, absence of RAPD, bilateral optic disc edema, and full structural recovery without RNFL or ganglion cell complex thinning. These findings are more consistent with reversible optic disc edema with asymmetric functional involvement, mimicking NAION in the right eye. Recent literature describes similar cases of optic disc edema temporally associated with semaglutide therapy and pharmacovigilance signals for optic nerve-related adverse events, while systematic reviews emphasize the lack of conclusive evidence for causality. This case adds to emerging data suggesting that semaglutide may be associated with a NAION-like presentation driven by non-ischemic mechanisms, such as transient axoplasmic flow disturbance or altered intracranial pressure regulation. Recognizing this distinction is essential to avoid misclassification as NAION and to guide management, including medication review and close ophthalmologic follow-up.

**Disclosures** The author declares no conflict of interest related to this work.

7. **Optic disc drusen masquerading as optic neuritis: An electrophysiological pitfall**

Aida Jallouli^1^, Imen Zone Abid^1^, Yosra Maalej^1^, Amira Trigui^1^

^1^Ophtalmology Department, Habib Bourguiba University Hospital Sfax, Tunisia

**Introduction**: While optic disc drusen (ODD) are a frequent etiology of pseudo-papilledema, deeply buried variants present a significant diagnostic conundrum. Their ability to mimic the structural and functional profiles of optic neuropathy represents a major diagnostic pitfall, often leading to extensive, invasive, and potentially unnecessary clinical investigations.

**Case presentation**: A 10-year-old girl presented with recurrent headaches associated with vomiting, without fever. Her medical history was notable for chronic inflammatory polyarthralgia of unknown etiology.

Ophthalmological examination revealed a best-corrected visual acuity of 10/10 in both eyes. Funduscopic examination showed bilateral optic disc swelling, more pronounced in the right eye.

Fluorescein angiography was normal. However, electrophysiological assessment showed a significant prolongation of the P100 latency on visual evoked potentials. Goldman visual field testing confirmed the presence of an inferonasal visual field defect. Optical coherence tomography of the optic nerve head and ganglion cell complex (GCC) revealed a localized superotemporal retinal nerve fiber layer (RNFL) defect associated with corresponding GCC thinning.

The patient’s systemic inflammatory history and functional deficits led to a presumptive diagnosis of right optic neuritis, despite unremarkable brain MRI findings (aside from an incidental arachnoid cyst). However, this diagnosis was overturned through longitudinal follow-up and multimodal imaging. Serial VEPs showed stationary P100 latencies, inconsistent with the typical recovery or sequelae of demyelinating neuropathy. The definitive diagnosis of buried Optic Disc Drusen (ODD) was established via B-scan ultrasonography and OCT, which identified pathognomonic hypo-reflective ovoid structures.

**Discussion**: The diagnostic challenge of buried ODD stems from their ability to induce structural and functional impairments that closely mimic inflammatory optic neuropathies.

Structural alterations in the Retinal Nerve Fiber Layer (RNFL) are a hallmark of ODD, occurring in 83.6% of cases. The process typically begins with segmental swelling of the peripapillary RNFL and Ganglion Cell Complex (GCC), followed by atrophy, particularly in the superotemporal sector, while the papillomacular bundle is characteristically spared. These structural changes directly impair nerve conduction; Visual Evoked Potentials (VEP) show P100 latency prolongation in up to 83% of patients, often accompanied by reduced amplitudes. These anomalies reflect chronic mechanical axonal compression and axoplasmic stasis rather than inflammatory demyelination. Furthermore, functional impairment manifests as visual field defects, with nasal defects being the most prevalent (54%), followed by field constriction (21%) and isolated enlargement of the blind spot (18%).

This case underscores that in the presence of pediatric papilledema and delayed VEPs, clinicians must maintain a high index of suspicion for buried ODD, even when a systemic inflammatory history may act as a confounding factor. Multimodal assessment, integrating both structural and functional testing, is crucial to establish an accurate diagnosis and to preempt inappropriate management, particularly when faced with confounding systemic inflammatory history.

10. **Epidemiology and clinical outcomes of traumatic optic neuropathy in Taiwan: A multicenter retrospective study**

Ching-Lung Chen^1^, Meng-Hung Lin^2^, Yung-Kang Chen^1^, Nai-Hua Kuo^1^, Ching-Yun Wang^1^

^1^Department of Ophthalmology, Chiayi Chang Gung Memorial Hospital, Chiayi County, Taiwan (R.O.C.); ^2^Health Information and Epidemiology Laboratory, Chiayi Chang Gung Memorial Hospital, Chiayi County, Taiwan (R.O.C.).

**Introduction**: Traumatic optic neuropathy (TON) is a vision-threatening consequence of craniofacial trauma that presents with acute visual loss and a relative afferent pupillary defect. Current management options include corticosteroid therapy, surgical decompression, or observation, but no standard treatment has been established. This retrospective multicenter study in Taiwan evaluates the epidemiology and visual outcomes of TON to identify clinical predictors of visual recovery.

**Methods**: This retrospective multicenter study analyzed TON cases identified from the Chang Gung Research Database (2007–2021), categorized by management into surgical decompression, intravenous corticosteroids, oral corticosteroids, or observation. Visual acuity was converted to logMAR and evaluated longitudinally at 6, 12, and 24 months relative to a 3-month baseline, with outcomes classified as improved, unchanged, or worsened. Statistical analyses included one-way ANOVA for baseline comparisons, the Stuart–Maxwell test for longitudinal paired categorical changes, and chi-square tests for comparing outcome distributions between management groups. Additionally, the Cochran–Armitage test assessed temporal trends in traffic accident–associated cases, with significance defined as *p* < 0.05.

**Results**: A total of 937 patients with TON were included, of whom 137 underwent surgical decompression, 108 received intravenous corticosteroids, 42 received oral corticosteroids, and 650 were managed with observation. Follow-up declined over time, with evaluable cases decreasing from 599 at 3 months to 136 at 2 years. The proportion of TON cases attributable to traffic accidents declined significantly between 2007 and 2021 (χ ^2^ = 44.4, *p* < 0.001). Baseline logMAR visual acuity at 3 months did not differ significantly among management groups (*p* = 0.120). Overall, visual outcomes demonstrated a significant shift toward improvement at 6 months, 1 year, and 2 years relative to baseline (all *p* < 0.05). In contrast, comparisons of visual outcome distributions across management groups showed no significant differences at 6 months (*p* = 0.196), 1 year (*p* = 0.582), or 2 years (*p* = 0.273). Although numerical differences were observed, including higher early improvement rates in the intravenous corticosteroid group and greater long-term stability in the observation group, these trends did not reach statistical significance.

**Discussion**: Over the 15-year study period, the incidence of TON in Taiwan declined significantly, driven by a reduction in traffic accident–related cases. While the overall cohort showed significant visual improvement over 24 months, no statistically significant differences in outcome distributions were found between management groups at any follow-up interval. These findings are limited by a substantial reduction in sample size over time, which introduces potential follow-up bias where patients with poor recovery may be more likely to remain in the database.

**Disclosures**: The authors declare no conflicts of interest.

12. **Brain MRI abnormalities along the visual pathways in subacute Leber Hereditary Optic Neuropathy: Prevalence and clinical correlations**

Giulia Amore^1^, Michele Carbonelli^2^, Martina Romagnoli^1^, Caterina Tonon^1,2^, Raffaele Lodi^1,2^, Valerio Carelli^1,2^, Chiara La Morgia^1^

^1^IRCCS Istituto delle Scienze Neurologiche di Bologna, Bologna, Italy; ^2^DIBINEM Department of Biomedical and Neuromotor Sciences, University of Bologna, Bologna, Italy.

**Introduction**: Brain MRI abnormalities along the visual pathways are historically considered an uncommon feature of Leber Hereditary Optic Neuropathy (LHON) at difference with inflammatory optic neuritis. We aimed at describing the prevalence of such MRI abnormalities in subacute LHON patients, correlating with clinical and genetic characteristics.

**Methods**: We retrospectively analysed clinical, genetic and ophthalmological data from 40 genetically-confirmed LHON patients. MRI data performed within 6 months from onset, including T2-weighted FLAIR/Fat-Sat and T1-weighted pre and post-gadolinium sequences, were collected. Prevalence and distribution of MRI abnormalities were calculated. Best corrected visual acuity (BCVA), retinal nerve fiber layer (RNFL) thickness and visual fields parameters were compared between patients with and without MRI abnormalities at time of MRI execution and at follow-up (≥24 months). Linear mixed models were applied to account for inter-eye correlation, with clinical, genetic and therapeutic predictors as covariates.

**Results**: MRI abnormalities along the visual pathways were found in 20/40 (50%) patients. Optic nerve hyperintensity was present in 18/40 (45%), chiasm and optic tract involvement in 8/40 (20%) respectively, and contrast enhancement of the optic nerve in 9/40 (22%) patients. No difference in terms of age, gender, mutation time and disease duration was found between patients with and without MRI abnormalities. At time of MRI execution, optic nerve hyperintensity resulted significantly associated with worse BCVA (β=–0.474, p=0.002) and visual field parameters (MD β =–6.77, p=0.007; fovea β =10.16, p=0.040). Optic nerve contrast enhancement was selectively associated with thinner temporal RNFL (β = 22.86, p = 0.046), while no association was found with chiasm and tracts involvement. 25/40 patients had a follow up of at least 24 months and all received idebenone 900 mg/die. Long-term BCVA as well as average and inferior RNFL thickness were significantly influenced by mutation type, with rare mutations showing better outcomes compared to m.11778/ND4 and m.3460/ND1 mutations. MRI abnormalities were not prognostic, except for a significant association of contrast enhancement with reduced average ganglion cell layer (GC-ILP) thickness (β =2.85, p = 0.011). As exploratory analysis, high-dose steroid therapy at onset (19/40 patients) showed association with better MD (β =12.53, p=0.004) and a borderline effect on BCVA (β =0.49, p=0,057) at follow-up compared with 6 untreated patients.

**Discussion**: MRI abnormalities of the visual pathways are a frequent finding in subacute LHON (50%) likely reflecting early axonal damage. Optic nerve hyperintensity resulted the most common finding (45%) associated to worse visual function in early phases but not to long-term prognosis. Contrast enhancement, when present (22%), showed stronger association with structural damage, suggesting the presence of a transient inflammatory component in early phases of LHON (supported also by the observation that early steroid therapy may influence final functional parameters).

**Disclosures**: consultant and travel fees from Chiesi Pharmaceuticals

15. **Differentiation between arteritic and nonarteritic anterior ischemic optic neuropathy using optical coherence tomography**

Andreas Worm Bendtsen^1^, Michael Stormly Hansen^1^, Oliver Niels Klefter^1^, Steffen Hamann^1^

^1^Department of Ophthalmology, Copenhagen University Hospital, Copenhagen, Denmark

**Introduction**: The role of optical coherence tomography (OCT) in distinguishing arteritic anterior ischemic optic neuropathy (A-AION) from nonarteritic AION (NA-AION) remains insufficiently investigated. A recent study suggests that the combination of two retinal biomarkers, paracentral acute middle maculopathy (PAMM) and peripapillary intra- or subretinal fluid (IRF/SRF) with macular extension, can assist in the early differentiation between these conditions. Here, we aim to further evaluate the prevalence of these retinal biomarkers in A-AION and NA-AION and to estimate the diagnostic value of combining them.

**Methods**: We retrospectively investigated OCT scans of AION patients seen at the Department of Ophthalmology at Copenhagen University Hospital - Rigshospitalet, Denmark, between January 2021 and December 2024. Macular OCT scans obtained within 30 days of reported visual loss were independently analyzed by two masked graders. The primary outcomes were presence of PAMM and peripapillary IRF/SRF with macular extension, defined as fluid extending continuously from the peripapillary region into the Early Treatment Diabetic Retinopathy Study (ETDRS) grid centered on the fovea. Bilateral cases were restricted to the eye with the highest degree of papilledema. The final clinical diagnosis of A-AION or NA-AION was used as the reference standard. Finally, we calculated the sensitivity and specificity (with 95 % confidence intervals) of the two distinct OCT biomarker patterns for diagnosing A-AION and NA-AION, respectively. Results below are preliminary and based on a single independent grader, as second grading is ongoing.

**Results**: PAMM was present in 7 of 26 patients (26.9 %) with A-AION and in none of the 149 patients with NA-AION. Peripapillary IRF/SRF with macular extension was present in 1 of 26 patients (3.8 %) with A-AION – who also had PAMM - and in 90 of 149 patients (60.4 %) with NA-AION. Combined presence of PAMM and absence of IRF/SRF demonstrated a sensitivity of 23.1 % (9.0 - 43.6 %) and a specificity of 100.0 % (97.6 – 100 %) for diagnosing A-AION. Combined presence of IRF/SRF and absence of PAMM demonstrated a sensitivity of 60.4 % (52.1 – 68.3 %) and a specificity of 100.0 % (86.8 – 100.0 %) for diagnosing NA-AION. Collectively, 96 of 175 patients (54.9%) with AION were correctly classified based on these OCT-derived biomarker patterns alone.

**Discussion**: An isolated finding of PAMM in patients with AION appears highly indicative of an arteritic etiology, although the pattern is relatively uncommon. In contrast, isolated peripapillary IRF/SRF with macular extension appears to be much more common in NA-AION than in A-AION. Collectively, these results suggest that OCT-derived biomarkers may be a valuable tool in the early differentiation between A-AION and NA-AION due to the high specificity of these patterns.

**Disclosures**: The authors have nothing to declare.

22. **Clinical features and Genotype-Phenotype correlations in Bosch-Boonstra-Schaaf Optic Atrophy Syndrome**

Samia Ghazali^1^, Reda Ghazali^2^, Veeral Shah^3^, Christian Schaaf^4^

^1^Université d’Angers, Angers, France; ^2^Université Libre de Bruxelles, Brussels, Belgium; ^3^Cincinnati Children’s Hospital Medical Center, Cincinnati, OH, USA; ^4^Universitätsklinikum Heidelberg, Heidelberg, Germany

**Introduction**: Bosch–Boonstra–Schaaf optic atrophy syndrome (BBSOAS) is a recently described autosomal dominant neurodevelopmental disorder caused by a pathogenetic mechanism of haploinsufficiency of the nuclear receptor subfamily 2 group F member 1 gene (*NR2F1*, also known as COUP-TF1). The syndrome is characterized by a broad spectrum of ophthalmologic and neurodevelopmental abnormalities - including optic atrophy and impaired intellectual development. We aimed to characterize the clinical manifestations, neuroimaging features, and molecular specificities of BBSOAS and to evaluate genotype–phenotype correlations, with a particular focus on visual outcomes.

**Methods:**: Taking advantage of a 2018 international family conference that brought patients with BBSOAS and scientific experts, individuals with genetically confirmed BBSOAS were enrolled. Data were prospectively collected and analyzed from January 1, 2019, through December 3, 2023. Clinical findings, neuroimaging features, and molecular data were obtained to characterize the phenotype and assess genotype–phenotype correlations. Associations between genotype and visual acuity, optic nerve abnormalities, and brain MRI findings were evaluated using a three-tier group stratification based on *NR2F1* mutation location: (1) DNA-binding domain, (2) ligand-binding domain, and (3) other regions. Statistical analyses were performed to compare outcomes across the three groups.

**Results**: The study included 26 patients with genetically confirmed *NR2F1* mutations (11 males [42%] and 15 females [58%]). The mean age at evaluation was 8.1 years (range: 2–37 years). Comprehensive neuro-ophthalmic assessment of the full cohort demonstrated optic atrophy with or without optic nerve hypoplasia in 92%; decreased visual acuity in 88% of patients, nystagmus in 65%, while 65% of the cohort screened positive for cerebral visual impairment (CVI).

Visual acuity, expressed in logMAR, differed according to genotype. Patients harboring ligand-binding domain mutations exhibited better visual outcomes, a lower incidence of nystagmus, and less severe optic nerve abnormalities compared with the other two groups (p < 0.005).

Neuroimaging was available for 19 of 26 patients; 18 of 19 (95%) demonstrated a characteristic, recently described but nonspecific finding of mesial temporal dysgyria.

**Discussion**: In this 5-year prospective cohort study, we provide the first evidence of genotype–phenotype correlations linking *NR2F1* mutation location to optic nerve abnormalities and visual acuity. Our clinical and neuroimaging findings expand the phenotypic spectrum of BBSOAS and highlight the high prevalence of neurologic and ocular abnormalities in BBSOAS patients. Optic atrophy emerged as the hallmark ocular feature and should prompt consideration of BBSOAS in the appropriate clinical context. These results may inform patient counseling, and help guide clinical management.

**Disclosures**: The authors report no conflicts of interest.

23. **Structure–function analysis of OPA1-associated autosomal dominant optic atrophy using AI-segmented OCT and visual field metrics**

Vasileios Batis,^1^ Mehdi Ounissi^1,2^, Elina Priquet^1^, Ana Banc^1,2^, Catherine Vignal-Clermont ^1^, Dan Milea^1,2^

^1^Department of Neuro-Ophthalmology, Rothschild Foundation Hospital, Paris, France; ^2^Rothschild Computational and Visual Neurosciences (BRAIN) Laboratory, Rothschild Foundation Hospital, Paris, France

**Introduction**: Autosomal dominant optic atrophy (ADOA) is the most common inherited optic neuropathy and is frequently caused by pathogenic variants in OPA1. Clinical expression is heterogeneous, with variable trajectories of visual acuity, visual field loss and characteristic structural changes on optical coherence tomography (OCT), including thinning of the peripapillary retinal nerve fiber layer (pRNFL) and macular cell layers (GCL-IPL). Recent artificial intelligence–based segmentation enables reproducible extraction of OCT-derived biomarkers, including sector-level pRNFL and GCL–IPL measurements, which may improve anatomical–functional characterization beyond conventional global or quadrant summaries. We aimed to investigate structure–function relationships in a genetically confirmed OPA1-ADOA cohort by relating AI-segmented OCT metrics to visual field indices and visual acuity, with adjustment for age.

**Methods**: We performed a retrospective chart review of patients with genetically confirmed OPA1 variants followed at the Neuro-Ophthalmology Department of the Foundation Adolphe de Rothschild Hospital (2014–2026). OCT B-scan exports were processed using an in-house Vision Transformer segmentation pipeline to delineate retinal layer boundaries and derive peripapillary RNFL and macular ganglion cell–inner plexiform layer (GCL–IPL) thickness metrics. pRNFL thickness profiles were sampled circumferentially and summarized into 36 contiguous 10° sectors after alignment, while GCL–IPL thickness was extracted from macular scans. Visual function outcomes included best-corrected visual acuity and standard automated perimetry parameters (mean deviation and foveal sensitivity), as well as a visual field pattern grade (0-5) based on defect morphology. Associations between structural and functional measures were assessed using Spearman correlations with Benjamini–Hochberg false discovery rate correction and age-adjusted partial correlations; multivariable performance was evaluated using cross-validated ridge regression and ROC-AUC models.

**Results**: Sixty-four participants were included (33 male/31 female). Mean age at last examination was 38.9 years (range 8.3–78.8). Across all parameters tested, the strongest predictors of visual acuity (VA) were central visual field (VF) measures: foveal sensitivity showed the highest correlations (Spearman ρ=0.64–0.79; FDR q≤7.10×10⁻^5^) and VF mean deviation (MD) showed more moderate correlations (ρ≈0.48–0.49; q≈2.25×10⁻^2^); these associations remained significant after age adjustment. In contrast, OCT-derived metrics from AI-segmented pRNFL and GCL–IPL showed only weak-to-moderate associations with VA (maximum |ρ|≈0.36), and none remained significant after FDR correction.

**Discussion**: In this genetically confirmed OPA1-ADOA cohort, visual acuity was primarily associated with central visual field function, with foveal sensitivity showing the strongest and age-robust relationships. Although AI-segmented pRNFL and GCL–IPL measures showed substantial variability, their associations with visual acuity were weak and did not remain significant after correction for multiple testing. These findings support prioritizing central perimetry–based measures when assessing functional impact in OPA1-ADOA, while OCT segmentation remains valuable for anatomical characterization.

**Disclosures**: None declared.

24. **Visual prognosis in late-onset Leber Hereditary Optic Neuropathy in a large Italian cohort**

Marco Battista,^1^ Michele Carbonelli,^2^ Luigi Brotto,^3^ Giulia Amore,^2^ Catarina Coutinho,^4^ Martina Romagnoli,^5^ Alice Galzignato,^4^ Paolo Nucci,^3^ Leonardo Caporali,^5^ Claudio Fiorini,^5^ Chiara La Morgia,^2^ Valerio Carelli,^2,5^ Francesco Bandello,^1^ Piero Barboni^1,4^

^1^IRCCS Ospedale San Raffaele, Milan, Italy; ^2^Department of Biomedical and Neuromotor Sciences, University of Bologna, Bologna, Italy; ^3^Università degli Studi di Milano, Milan, Italy; ^4^Studio Oculistico d’Azeglio, Bologna, Italy; ^5^IRCCS Istituto delle Scienze Neurologiche di Bologna, Programma di Neurogenetica, Bologna, Italy

**Purpose:** To identify demographic and clinical features associated with visual recovery in patients affected by late-onset Leber Hereditary Optic Neuropathy (LHON).

**Methods:** A retrospective analysis was conducted on 398 patients affected with LHON to label cases with disease onset occurring after 40 years of age. In particular, demographics, clinical features (i.e. best-corrected visual acuity (BCVA)) were collected; mutation type, smoking history, comorbidity conditions, idebenone therapy and the visual recovery rate were considered for the analysis.

**Results:** The frequency of late-onset LHON in the entire cohort was 19.3% (77/398 patients). The prevalent mutation was m.11778 mutation (64.9%), followed by m.3460 mutation (15.6%), m.14484 mutation (13.0%) and other rare mutations (6.5%). The haplogroup data disclosed a higher frequency of haplogroup J (20.9%) and T (11.9) compared with controls (9.4 and 8.2% respectively). Fourteen patients were active or former moderate smokers (18.7%), 28 patients were active o former heavy smokers (>10 cigarettes per day, 37.3%) while 33 had never smoked (44.0%). Seven women (21.1%) had gone through menopause when the disease started. The most frequent ocular co-existing conditions was glaucoma (7.8%). Sixty-eight patients (89.5%) were treated with idebenone, while 5 patients did not receive any therapy. Twenty-nine patients experienced visual recovery (37.7%) which increase up to 43.5% excluding glaucoma and hormonal factors (worse prognosis, only 2/15 experienced visual recovery). In particular, the group who underwent idebenone therapy 900mg/day experienced better visual recovery (47.2%) in comparison with patients with regimen <900mg/day or no therapy. Furthermore, the heavy-smokers group had a better rate of recovery in comparison with the moderate and no-smokers.

**Conclusions:** This study disclosed the general features of patients affected by late-onset LHON. A better response to daily 900mg idebenone therapy was observed, with the main improvement observed in heavy smokers. Furthermore, systemic (hormonal) or local (glaucoma) factors should be considered negative prognostic factors. Late-onset LHON is a genetic-based disease in which an important role seems to be played by toxic factors, thus therapy needs to be aimed to counteract toxic effects.

25. **Analysis of parent-of-origin effects on clinical parameters in *OPA1* autosomal dominant optic atrophy**

Berthold Pemp^1^, Johannes Schrittwieser^1^, Karl Kircher^1^, Martin Bertich^1^, Christoph Stapf^1^, Reginald E. Bittner^2^, Wolfgang M. Schmidt^2^, Andreas Reitner^1^

^1^Department of Ophthalmology and Optometry, Medical University of Vienna, Vienna, Austria; ^2^Neuromuscular Research Department, Center of Anatomy and Cell Biology, Medical University of Vienna, Vienna, Austria

**Purpose**: Parent-of-origin effects on disease expression have previously been shown for several autosomal inherited diseases that impair mitochondrial function, pointing to an important role of mito-nuclear interaction in mitochondrial disease. To investigate a possible impact of the inheritance mode in *OPA1*-related autosomal dominant optic atrophy (ADOA), we explored if clinical parameters vary differently in case of paternal compared to maternal transmission.

**Methods**: Pairs of affected parent and offspring, genetically confirmed with a disease-causing heterozygous variant in *OPA1*, were analyzed retrospectively. Examined parameters included best-corrected visual acuity (VA), global mean deviation (MD) from 30-2 threshold perimetry, MD in the papillomacular bundle (PMB) subfield, and inner retinal layer thickness results from spectral-domain optical coherence tomography (OCT). The average values of both eyes were used to calculate differences between parent and offspring. The absolute values of differences were stratified by sex of the parent and then compared using Mann-Whitney-U tests.

**Results**: Complete data were available for 27 parent-offspring pairs with ADOA, of which 12 had maternal and 15 had paternal inheritance. The difference in PMB-MD between the affected father and offspring was significantly greater compared to families with maternal transmission, with median differences of 5.58 dB (IQR 2.67–10.83) and 0.71 dB (IQR 0.38–1.96), respectively (*P* = 0.0001). Generational differences in VA, global MD and OCT parameters were not significantly influenced by the sex of the parent-of-origin. Patient age (maternal: 36.6 ± 17.4 years, paternal: 34.1 ± 18.9 years) and the age difference (maternal: 28.0 ± 5.1 years, paternal: 31.5 ± 7.2 years) were comparable between both groups (*P* = 0.74 and *P* = 0.26, respectively).

**Conclusions**: The severity of central scotoma in *OPA1*-related ADOA shows greater differences between affected generations in cases of paternal inheritance. This possibly indicates that impairment of retinal ganglion cell function due to a disease-causing variant in *OPA1* is to some part modified by the mitochondrial DNA background, which changes when the pathogenic allele is inherited from the father. This could either improve or worsen cellular adaptation to *OPA1* deficiencies and might contribute to the clinical heterogeneity in ADOA.

**Disclosures**: None.

26. **Retinal hyperreflective foci: A new potential biomarker in optic neuritis and multiple sclerosis**

Betul Okutan^1^, Michael Larsen^2^, Jette Lautrup Frederiksen^1^

^1^Department of Neurology, Clinic of Optic Neuritis, The Danish Multiple Sclerosis Center (DMSC), Rigshospitalet and University of Copenhagen, Copenhagen, Denmark; ^2^Department of Ophthalmology, Rigshospitalet and University of Copenhagen, Copenhagen, Denmark

**Introduction**: Retinal hyperreflective foci (HRF), presumed to represent aggregated microglial cells, have been detected on optical coherence tomography (OCT) in 70.5% of patients with relapsing-remitting multiple sclerosis (RRMS) who have no history of optic neuritis (ON). This study investigates the presence of HRF on OCT in patients with ON and examines their potential association with established biomarkers in RRMS.

**Methods**: In this cross-sectional study, patients with unilateral ON referred for evaluation of potential demyelinating disease, along with healthy controls, underwent high-resolution spectral-domain optical coherence tomography (SD-OCT). Exclusion criteria included any prior ophthalmic pathology, systemic condition affecting the visual system, and other etiologies for ON. HRF were defined according to the latest international consensus criteria as hyperreflective structures measuring less than 30 μm in diameter, without back shadowing, and located within the avascular outer nuclear layer (ONL) of the retina. Both affected (ON) and healthy (NON) eyes were examined in a dark room without the use of a mydriatic agent. A total of 121 macula volume scans, spaced 60 μm apart, were obtained from each eye. HRF were quantified within the macular region.

**Results**: A total of 21,538 OCT scans from 178 eyes were analysed in 93 patients with ON (RRMS = 60, CIS/idiopathic ON = 33) and 53 healthy controls. HRF were detected in at least one eye in 89.3% of patients compared to 1.8% in controls. HRF were present in the ON eye in 72% (median: 7, IQR: 1-18) and in the NON eye in 80.7% (median 4, IQR: 1-10). A higher prevalence of HRF were found in ON eyes (p<0.001). HRF count in the ON eye correlated negatively with ganglion cell and inner plexiform layer (GCIPL) thickness (p=0.033). Further analyses regarding MRI lesions and CSF are ongoing.

**Discussion**: HRF are prevalent in both ON and NON eyes, suggesting a widespread inflammatory process. The negative correlation between HRF count and GCIPL thickness may indicate early disease activity and neurodegeneration in multiple sclerosis.

**Disclosures**: The authors declare no conflicts of interest.

30. **Macular edema secondary to rozanolixizumab: A case report**

Bernardo Sánchez Dalmau^1^Núria Domènech López^1^, Pau Otal Aran^1^, Diego Santolaria^1^, Rafel Alcubierre Bailac^1^, Covadonga Menéndez Acebal^1^, Anna Camós Carreras^1^, M. Sepúlveda^2^

^1^Department of Ophthalmology, Hospital Clínic de Barcelona, Barcelona, Spain; ^2^Department of Neurology, Hospital Clínic de Barcelona, Barcelona, Spain

**Purpose**: To describe a case of cystoid macular edema (CME) temporally associated with rozanolixizumab treatment in a patient with Myelin Oligodendrocyte Glycoprotein Antibody-Associated Disease (MOGAD).

**Methods**: Observational case report of a patient enrolled in a phase 3 clinical trial evaluating rozanolixizumab for relapse prevention in MOGAD. Clinical history, ophthalmologic findings, treatment course, and outcomes were reviewed.

**Results**: A 70-year-old woman with relapsing MOGAD-related optic neuritis, refractory to previous immunosuppressive therapy, was enrolled in the phase 3 cosMOG clinical trial and received 30 weekly subcutaneous doses of rozanolixizumab (420 mg). Her ocular history included myopia and ocular hypertension treated with bimatoprost and timolol. During the study, she experienced two further episodes of optic neuritis requiring high-dose corticosteroids, one of which additionally required plasmapheresis and intravenous immunoglobulins.

Following the 30th dose of rozanolixizumab, she presented with blurred vision in the right eye. Best-corrected visual acuity was 20/32. Fundus examination and optical coherence tomography revealed cystoid macular edema without signs of anterior chamber or vitreous inflammation. Topical antiglaucoma medications were discontinued and topical ketorolac was initiated, with no improvement. Two weeks after discontinuation of rozanolixizumab, complete resolution of the macular edema was observed, and visual acuity recovered to 20/20, consistent with baseline status.

**Conclusions**: Cystoid macular edema is not a recognized feature of MOGAD-related optic neuritis and has not previously been reported as an adverse event associated with rozanolixizumab. The temporal relationship between drug exposure and both onset and resolution of CME suggests a possible association. Rozanolixizumab is already approved in Europe for generalized myasthenia gravis, and its clinical use is expected to expand as new indications are investigated. Clinicians should be aware of potential visual adverse events, and patients receiving this therapy should be advised to promptly report any changes in visual acuity. Further pharmacovigilance and reporting are warranted to clarify this potential relationship.

31. **Optic neuritis: A single-centre case series**

Ailbhe Burke^1^, Lola Ogunbowale^1^

^1^Moorfields Eye Hospital, London, UK

**Background**: Optic neuritis (ON) is a subacute inflammatory disorder of the optic nerve which often represents the first manifestation of central nervous system demyelinating disease though isolated episodes occur. In some cases early recognition and appropriate management are critical for visual recovery, and in all cases important to determine long-term neurological care and outcomes.

We present a single-centre case series describing the clinical characteristics, radiological findings, treatment approaches, and visual outcomes of patients diagnosed with optic neuritis within the Moorfields Eye Hospital London Neuro-emergency clinic after it transitioned under our direct care Spring 2020 to 2025.

**Methods**: We conducted a retrospective observational review of patients diagnosed with optic neuritis at a tertiary care centre 2020-2025. Clinical data including demographics, presenting symptoms and history of neurogenic symptoms, visual acuity, colour vision, pupillary reflexes, visual fields, fundoscopic findings in particular optic disc swelling, MRI brain and orbit results, treatment administered, and follow-up outcomes including ultimate diagnosis were collated from medical records. Visual recovery was assessed at standard follow up intervals which includes the aim of a rebaselining outcome evaluation at least 3.5 months after the onset of the episode. Harms and benefits of our aggressive oral steroids approach were looked for and evaluated

**Conclusion**: In our single-centre experience, optic neuritis predominantly affected young adults but was found across a wide age range, in men and in a range of ethnicities. The majority of patients experience good documented recovery of visual function even in the presence substantial atrophy, and our view is that OCT is now an essential part of diagnostic evaluation in this presentation. MRI findings were valuable in identifying patients with or at higher risk for demyelinating disease (multiple sclerosis) while bloods and other tests were more helpful in other causes of optic neuritis.

34. **Retinal imaging features as diagnostic biomarkers in ARSACS**

Maria Lucia Cascavilla^1^, Antonio De Santis^2^, Lorenzo Bianco^1^, Maurizio Battaglia Parodi^1^

^1^Ophthalmology Department, IRCCS San Raffaele Scientific Institute, Milan, Italy; ^2^IRCCS Azienda Ospedaliera Universitaria di Parma, Parma, Italy

**Introduction**: Autosomal recessive spastic ataxia of Charlevoix-Saguenay (ARSACS) is a rare neurodegenerative disorder caused by biallelic *SACS* mutations. Retinal imaging has identified distinctive developmental abnormalities, including absence of the foveal avascular zone (FAZ) and reduced fovea-to-disc distance (FDD). These features have emerged as highly characteristic but underrecognized biomarkers. The aim of this study was to evaluate FAZ morphology, foveal vascular density, and FDD in patients with ARSACS and to compare these findings with device-based and literature normative reference values.

**Methods**: Ten patients (20 eyes) with genetically confirmed ARSACS underwent swept-source optical coherence tomography (SS-OCT) and optical coherence tomography angiography (OCTA) using the DRI Triton SS-OCT (Topcon, Tokyo, Japan). FAZ morphology and foveal vascular density were assessed on 6×6 mm OCTA scans of the capillary plexus. FDD was manually measured on structural OCT as the linear distance between the foveal center and the optic disc center. Imaging findings were interpreted using published normative data and the device-based reference ranges provided by the Triton platform, in the absence of an internal healthy control group.

**Results**: Complete absence of a physiological FAZ was observed in all examined eyes (100%). According to Triton reference values and published normative data, normal superficial foveal vascular density is typically <20%. In contrast, all ARSACS eyes showed persistent foveal capillary networks with markedly increased superficial foveal vascular density (mean 34.3 ± 4.2%). All patients also demonstrated a significantly reduced FDD (mean 3.69 ± 0.17 mm), compared with published normal values of approximately 4.5–5.0 mm. Shortened FDD was consistently associated with foveal hypoplasia and contributed to apparent macular segmentation errors on automated analyses.

**Discussion**: This study confirms that complete absence of the FAZ and abnormally increased foveal vascular density, together with reduced FDD, represent consistent and highly distinctive retinal features of ARSACS. Comparison with Triton-based and literature normative values supports a primary developmental abnormality of foveal vascularization and positioning rather than a secondary microvascular process. These parameters represent reliable, non-invasive imaging biomarkers that may facilitate the differential diagnosis of hereditary ataxias and improve understanding of sacsin-related neurodevelopmental mechanisms.

**Disclosures**: The authors report no relevant financial or proprietary interests.

36. **Ocular syphilis with optic nerve involvement: A neuro-ophthalmologic case series from a tertiary center**

Celso Costa^1^, Telma Machado^1^, Miguel Raimundo^1,2,3^, Rosa Sá ^5^, Fernando Rodrigues^5^, Cristina Fonseca^1,2,3^, Pedro Fonseca^1,2,3^

^1^Department of Ophthalmology, Unidade Local de Saúde de Coimbra, Coimbra, Portugal; ^2^Faculty of Medicine, University of Coimbra (FMUC), Coimbra, Portugal; ^3^Clinical Academic Center of Coimbra (CACC), University of Coimbra, Coimbra, Portugal; ^4^Department of Infectious Diseases, Unidade Local de Saúde de Coimbra, Coimbra, Portugal; ^5^Department of Clinical Pathology, Unidade Local de Saúde de Coimbra, Coimbra, Portugal

**Introduction**: The rising incidence of syphilis has led to more cases of ocular syphilis (OS), a rare but potentially sight-threatening condition with variable clinical presentations. Optic nerve (ON) involvement is frequent and diagnostically challenging, sometimes representing the initial or predominant feature. It may present as optic disc edema or optic neuritis, occasionally without overt intraocular inflammation, reflecting presumed neurosyphilis and requiring multimodal imaging. This study aims to characterize the neuro-ophthalmologic spectrum of OS, focusing on optic nerve involvement and visual outcomes.

**Methods**: We conducted a retrospective, single-center study of patients diagnosed with OS by serology from January 2015 to December 2023. Demographics, systemic findings, symptom duration, and ophthalmologic features were collected. All patients underwent comprehensive ophthalmologic evaluation with OCT, wide-field fundus photography, and FAF. Optic neuropathy was distinguished from secondary optic disc edema by the absence of anterior or posterior uveitis. BCVA at presentation by eye and final follow-up was analyzed in relation to clinical phenotypes, particularly optic nerve involvement.: All patients were admitted and underwent a 2-week endovenous penicillin course.

**Results**: Fifty-four patients were included. Most (90.74%) presented with decreased visual acuity; 46.30% had symptoms <1 week. Systemic findings were present in 40.74%, mainly skin rash. Bilateral involvement occurred in 66.67%. Posterior segment involvement was detected in 85.19%, most commonly posterior placoid chorioretinitis (50.0%) and multifocal outer retinitis (21.74%). Panuveitis occurred in 38.89%.: ON involvement was observed in 22 patients (40.74%); 7 (31.82%) had isolated optic neuropathy, while 15 (68.18%) had optic disc edema secondary to uveitis. Among the 7 isolated optic neuropathies, 3 were bilateral simultaneous, 2 unilateral, and 2 bilateral sequential. Subtle posterior segment findings were often detectable only on FAF or OCT. OCT showed outer retinal disruption in 37.04%; cystoid macular edema was present in 18.52%.: Patients with ON involvement had better baseline BCVA (0.40 ± 0.59logMAR) than those without (0.98 ± 0.81logMAR; Z=3.368, p<0.001) and better final BCVA (0.11 ± 0.32logMAR vs 0.37 ± 0.55logMAR; Z=4.470, p<0.001), while patients without ON involvement showed greater improvement. Patients with Isolated optic neuropathy had similar baseline BCVA to others (0.41 ± 0.55logMAR vs 0.75 ± 0.79logMAR; p=0.142), but significantly better final BCVA (0.02 ± 0.04logMAR vs 0.30 ± 0.50logMAR; Z=3.003, p<0.01).

**Discussion**: ON involvement is a frequent and clinically relevant manifestation of OS, presenting as isolated optic neuropathy or reactive disc edema. Isolated optic neuropathy without overt uveitis reflects variable mechanisms of ON involvement. Subtle posterior segment findings highlight the role of multimodal imaging. Although patients with ON involvement had better baseline and final BCVA, those without showed greater visual improvement, reflecting differences in associated retinal or inflammatory involvement rather than a protective effect. Early recognition is crucial to prevent irreversible visual loss.

**Disclosures**: The authors report no relevant financial or proprietary interests.

37. **Neuro-ophthalmological findings in a Brazilian cohort of patients with Wolfram syndrome treated with idebenone compared with untreated controls**

Filipe Chicani

Neurovision Institute, Sao Paulo, Brasil

**Summary**: Twelve Brazilian patients with Wolfram syndrome were followed for a mean period of 56 months (range: 12–114 months; 1–9.5 years), undergoing biannual neuro-ophthalmological evaluations. Assessments included clinical history (new complaints, medication use), ocular history (surgical procedures, refractive correction), family history (ocular and neurological diseases), and comprehensive ophthalmological examination: best-corrected visual acuity (distance and near), inspection, ocular motility, slit-lamp biomicroscopy, tonometry, color vision testing (Ishihara plates), pupillary reflexes, Humphrey visual field testing, fundoscopy, retinography, and optical coherence tomography (OCT). Laboratory monitoring included liver function tests.

Patients were divided into two groups:Group 1: Treated with idebenone (7 patients)Group 2: Followed without clinical treatment (5 patients)

**Introduction**: Wolfram syndrome, also known as DIDMOAD, is a rare inherited disorder characterized by diabetes insipidus, childhood-onset diabetes mellitus, optic atrophy leading to progressive visual loss, and deafness. Additional manifestations may include bladder and bowel dysfunction, vestibular deficits, impaired thermoregulation, ataxic gait, olfactory deficits, and psychiatric symptoms such as anxiety and depression.

Two forms have been described: type 1, caused by mutations in the WFS1 gene, and type 2, associated with CISD2 mutations. Both are usually inherited in an autosomal recessive manner, although autosomal dominant type 1 cases have been reported. Diagnosis is suspected in patients with childhood-onset diabetes mellitus associated with optic atrophy not attributable to diabetic complications.

Currently, no definitive treatment exists, and management is mainly supportive. Given the role of endoplasmic reticulum stress and calcium homeostasis, therapeutic strategies targeting these mechanisms may slow disease progression.

Idebenone, a coenzyme Q10 analogue with antioxidant properties, has shown benefits in Leber hereditary optic neuropathy by improving mitochondrial energy production. Although optic atrophy in Wolfram syndrome differs pathophysiologically, mitochondrial dysfunction has been implicated, supporting the rationale for idebenone use.

**Demographics**: In the treated group, mean treatment duration was 91 months (range: 50–113 months), with a mean age at treatment initiation of 16.5 years. Six patients were male and one female. Idebenone doses were determined and adjusted over time according to age, weight, and severity of the visual function impairment. In the untreated group, mean follow-up was 39 months, with a mean age of 22.8 years; four patients were female and one male.

**Results**: In the treated group, visual acuity worsened bilaterally in three patients, unilaterally in one, and remained stable in three. Visual fields improved in at least one eye in three patients, worsened bilaterally in two, and remained stable in two. OCT showed RNFL improvement in both eyes in two patients and in one eye in one patient.

In the untreated group, visual acuity worsened bilaterally in four patients. Visual fields could not be performed in three due to severity of visual impairment. OCT showed RNFL worsening in both eyes in three patients.

**Conclusions**: Idebenone appeared safe, with no clinical or laboratory adverse effects. Treated patients showed relative stabilization of visual function compared with untreated patients, who demonstrated progressive decline consistent with the natural history of Wolfram syndrome. Some of the treated patients maintained normal and stable visual acuity, visual fields and unchanged RNFL in OCT measurements over the entire period of follow-up, which not usually seen for this disease, in untreated patients. This represents one of the longest follow-up studies evaluating idebenone use in Wolfram syndrome.

38. **Prospective study of retinal diffusion-weighted imaging in central retinal artery occlusion**

Charlotte Pietrock^1^, Milena Miszczuk^2^, Eberhard Siebert^2^, Leon Alexander Danyel^1^

^1^Department of Neurology with Experimental Neurology, Charité – Universitätsmedizin Berlin, Corporate Member of Freie Universität Berlin, Humboldt-Universität zu Berlin, and Berlin Institute of Health, Berlin, Germany; ^2^Institute of Neuroradiology, Charité-Universitätsmedizin Berlin, Corporate Member of Freie Universität Berlin, Humboldt-Universität zu Berlin, and Berlin Institute of Health, Berlin, Germany.

**Introduction**: Retinal diffusion restriction (RDR) is frequently observed on diffusion-weighted imaging (DWI) in central artery occlusion (CRAO) and may aid in the diagnosis of retinal ischemia. As previous studies were limited by retrospective design and heterogenous DWI sequence employment, the optimal imaging protocol for RDR in CRAO remains unclear.

**Methods**: In this prospective cohort study, we compared the diagnostic performance of 3T DWI using conventional echo-planar imaging (EPI-DWI) with readout-segmented echo-planar imaging (RESOLVE) for the detection of RDR in acute CRAO. Adults with an ophthalmological diagnosis of CRAO underwent cerebral magnetic resonance imaging within 14 days of symptom onset. The acquired EPI-DWI and RESOLVE sequences were independently evaluated by two blinded neuroradiologists for the presence of RDR. Detection rates, standard test quality criteria and interrater agreement were analysed for both sequences.

**Results**: 39 CRAO patients (36% female, mean age 73.4 ± 12.5, median visual acuity 0.005 [IQR 0.005 – 0.030]) with 39 EPI-DWI and 36 RESOLVE scans were included in the study. Median time to imaging after onset was 68 hours [IQR 41.0 – 105.6]. RDR detection rates were higher in EPI-DWI (Reader 1: 0.51 (95% CI: 0.35 – 0.68); Reader 2: 0.67 (0.50 – 0.81)) than in RESOLVE (Reader 1: 0.45 (0.28 – 0.62); Reader 2: 0.52 (0.34 – 0.68) sequences. EPI-DWI had a sensitivity of 0.59 (95% CI: 0.47 – 0.70), specificity of 0.95 (0.87 – 0.99); positive predictive value of 0.92 (0.81 – 0.98) and negative predictive value of 0.70 (0.60 – 0.78) in detecting RDR. In contrast, RESOLVE sequences had a sensitivity of 0.49 (0.37 – 0.61), specificity of 0.99 (0.93 – 1.00), positive predictive value of 0.97 (0.85 – 1.00) and negative predictive value of 0.66 (0.56 – 0.75). Interrater agreement was “substantial” for EPI-DWI (unweighted κ = 0.65; 95% CI: 0.44 – 0.85) and “moderate” for RESOLVE (unweighted κ = 0.51; 95% CI: 0.27 – 0.75).

**Discussion**: In this first prospective study to compare DWI sequences for RDR detection in CRAO, EPI-DWI outperformed RESOLVE. However, sensitivities for both techniques were lower than previously reported. Our findings underscore the continued need to optimize imaging protocols for reliable detection of retinal ischemia using DWI.

39. **Recurrent missense variant in PPIB gene is associated with isolated late-onset dominant optic atrophy in Italian cohort**

Chiara La Morgia^1,2^, Claudio Fiorini^1^, Alberto Pietro Pasti^2^, Danara Ormanbekova^2^, Piero Barboni^3,4^, Michele Carbonelli^1^, Giulia Amore^2^, Marco Battista4, Luigi Brotto^5^, Paolo Nucci^5^, Leonardo Caporali^2^, Valerio Carelli^1,2^

^1^Department of Biomedical and Neuromotor Sciences, University of Bologna, Bologna, Italy; ^2^IRCCS Institute of Neurological Sciences of Bologna, Neurogenetic Unit, Bologna, Italy; ^3^Studio Oculistico d’Azeglio, Bologna, Italy; ^4^Department of Ophthalmology, University Vita-Salute, IRCCS Ospedale San Raffaele, Milan, Italy; ^5^Department of Clinical Science and Community Health, University of Milan, Milan, Italy

**Introduction:** A recurrent heterozygous missense variant (c.538C>T; p.Arg180Trp) in *PPIB* gene, encoding peptidylprolyl isomerase B, has been recently associated to late-onset isolated optic atrophy in 19 individuals from 9 families in Austria. Patient-derived fibroblasts revealed altered mitochondrial morphology, as well as subtle respiratory chain defects and mitochondrial network abnormalities (Valentin et al., 2026).

**Methods:** We screened a cohort of 210 optic neuropathy patients without a molecular diagnosis for the recently reported missense variant in *PPIB* gene. Neurophthalmological evaluation included assessment of best corrected visual acuity (BCVA), computerized visual fields (MD), fundus exam, optical coherence tomography (OCT), neurological exam and brain MRI

**Results:** We found the heterozygous missense variant c.538C>T (p.Arg180Trp) in *PPIB* gene in 9 additional cases belonging to 5 Italian families. In one family the co-occurrence of *Wolframin* variants was also documented, with the proband carrying also the missense c.2206G>A (p.Gly736Ser) and c.2029G>A (p.Ala677Thr) variants in the *WFS1* gene. Moreover, the two daughters carry the c.2029G>A and the c.2206G>A variants, respectively, while also harboring the pathogenic *PPIB* variant. The clinical phenotype of the proband, now 73-year-old, is characterized by diffuse optic atrophy with severe visual loss (BCVA 0.04 in OD, 0.032 in OS) starting at 20 years, diabetes mellitus and deafness since 60 years, and more recently diabetes insipidus, normal pressure hydrocephalus, neurogenic bladder and neurogenic orthostatic hypotension. The daughter carrying both the c.2206G>A variant in *WFS1* and the variant in *PPIB* genes presented isolated optic atrophy (BCVA 0.8), whereas the other carrying the c.2029G>A variant in *WFS1* and the variant in *PPIB* had both mild temporal asymptomatic optic atrophy (BCVA 1.25) and sensorineural deafness. The other 6 individuals all presented late-onset (age at onset ranging between 35 and 62 years of age) isolated optic atrophy with a predominant temporal optic pallor. Brain MRI was unremarkable.

**Discussion:** We here report a case-series of 9 italian dominant optic atrophy (DOA) cases carrying the same PPIB variant as recently reported with a similar phenotype (late-onset isolated optic atrophy) with the exception of the family in which also variants in *WFS1* gene were identified. PPIB is encoding a protein, which is not a mitochondrial protein, belonging to the group of cyclophilins targeted to the ER during the ER-stress response playing a role in the clearance of misfolded proteins, and involved in the collagen metabolism (recessive forms of PPIB are associated with osteogenesis imperfecta). The occurrence of secondary mitochondrial dysfunction has been preliminarily reported. Further functional studies are needed to better elucidate PPIB role in driving mitochondrial dysfunction.

**Disclosures**: None

40. **Retinal microRNA dynamics underlying the pathophysiology of traumatic optic neuropathy and the therapeutic effect exerted by an E3 ubiquitin ligase inhibitor**

Ching-Lung Chen^1^, Ching-Yun Wang^1^^*^ ^*^Corresponding author(s)., Shun-Ping Huang^2^*^^

^1^Department of Ophthalmology, Chang Gung Memorial Hospital, Chiayi, Taiwan; ^2^ Department of Biomedical Science and Technology, National Chiayi University, Chiayi, Taiwan

**Introduction:** No definite therapeutic approach has been validated for Traumatic optic neuropathy (TON). Our previous study demonstrated that an E3 ubiquitin ligase inhibitor (compound 056) exerts neuroprotective effects in a rodent-based optic nerve crush (ONC) model(doi:10.1016/j.exer.2025.110489). Transcriptomic analysis suggests that compound 056 influences the ubiquitination-to-autophagy switch, thereby decreasing the severity of optic neuritis and repopulating RGCs. This study aimed to examine the regulatory role of microRNA in the pathophysiology of TON and the therapeutic effect of a ubiquitin ligase inhibitor.

**Methods**: Fifty adult outbred Wistar rats were distributed into 3 experimental arms (ONC, control, ONC+ compound 056). The ONC model was established via a 60-gram microvascular clamp. Compound 056 was subcutaneously injected into the experimental rodent on day 1. On day 3, part of the animals in each experimental arm were euthanized to extract RNA samples from their retina and optic nerve. The rest of the animals received regular optic coherence tomographic examinations, retrograde retinal ganglion cell labeling, flashed-evoked potentials examinations, post-mortem TUNEL assay, and immunoblotting. MicroRNA sequencing data were analyzed using the SciPy (v 1.17.0), skbio.stats.composition.ancom (v 0.7.2) libraries, DAVID bioinformatics, and visualized using the matplotlib (v 3.5.3) package.

**Results**: The ONC rat had prolonged FVEP P1 latency, decreased RGC density, and an elevated intra-retinal apoptotic trend, all of which were rescued by compound 056 administration (Published data: doi:10.1016/j.exer.2025.110489). MicroRNA analysis showed that ONC retina exhibited upregulation of 15 miRNAs and downregulation of 9 miRNAs. Compound 056 administration reversed 8 out of the 15 upregulated miRNAs in ONC retina (NovelmiRNA-17, NovelmiRNA-149, NovelmiRNA-405, NovelmiRNA-248, NovelmiRNA-219, NovelmiRNA-186, NovelmiRNA-308, NovelmiRNA-304). Target prediction analysis showed several high-affinity transcript targets of these microRNAs. Furthermore, functional annotation of the predicted transcript targets showed significantly enriched GOBP terms: cellular response to nutrient level, negative chemotaxis, negative regulation of TORC1 signaling, positive regulation of TORC1 signaling, TORC1 signaling, and cell-cell adhesion mediated by cadherin.

**Discussion**: In the ONC rodent, subcutaneous injection of the E3 ubiquitin ligase inhibitor (compound 056) alleviated neuroinflammation, restored visual function, and repopulated RGCs. Simultaneously, a group of non-coding RNAs was reciprocally regulated following the compound 056 administration. Previous studies have already confirmed that mTORC1/mTORC2 dynamics at the translational level alter the autophagic flux and mediate ONC recovery. We proposed a higher-order, microRNA-wise, regulatory network for mTORC1 signaling underlying the mechanisms of optic nerve injury and its recovery.

**Disclosures**: The authors have no conflict of interest to disclose.

42. **Neuro-structural involvement of optic pathways in adult neurofibromatosis type 1: A comparative study of Optical Coherence Tomography (OCT) and Magnetic Resonance Imaging (MRI)**

Claudia Mainetti^1^, Claudia Cesaretti^1^, Marco Nassisi^1^, Laura Dell’Arti^1^, Cecilia Serra^2^, Alessandro Draghi^1^, Federica Natacci^1^, Francesco Viola^1^

^1^Fondazione IRCCS Ca’ Granda Ospedale Maggiore Policlinico Milano, Milan, Italy; ^2^Università Statale di Milano, Milan, Italy

**Introduction**: Neurofibromatosis type 1 (NF1) is a rare multisystem genetic disorder. A major complication in pediatric populations is optic pathway glioma (OPG), occurring in 15–20% of patients. However, the involvement of optic pathways in adults is less documented. Assessment traditionally relies on visual acuity (VA) and Magnetic Resonance Imaging (MRI); however, Optical Coherence Tomography (OCT) is emerging as a sensitive biomarker for monitoring retinal nerve fiber layer (RNFL) and ganglion cell layer (GCL) thickness. We evaluated the correlation between OCT, MRI findings, and visual function in a large population of adult NF1 patients.

**Methods**: A retrospective longitudinal study was conducted (December 2024 – May 2025) at the Policlinico of Milan. Data included VA (logMAR), OCT (RNFL/GCL), and optic pathway MRI. Eyes were categorized into four groups based on the intersection of OCT (RNFL status normal/abnormal based on normative database) and MRI (optic pathway involvement) findings. The annual rate of change (slope) was calculated to evaluate the potential progression of structural los**s** in both the RNFL and GCL.

**Results**: A total of 162 adult patients were enrolled (320 eyes).

At baseline, mean RNFL was 83±14 µm and mean GCL was 48.5±8 µm. Longitudinal follow-up (mean 25±12 months) was available for 203 eyes.

A significant association was found between abnormal RNFL and abnormal MRI (p < 0.034), with an Odds Ratio (OR) of 1.91 (95% CI: 1.04-3.52).

However, among the 48% of eyes with abnormal RNFL (154/320) only 20.7% (32/154) had MRI evidence of optic pathway involvement (OPI).

Longitudinal analysis showed stability across all sectors; no clinical or radiological parameters connected to NF1 significantly influenced the annual rates of RNFL and GCL change (P>0.05).

**Conclusions**: A marked discordance exists between OCT structural damage and MRI findings in adult NF1. While MRI abnormalities are a significant predictor of axonal damage (OR 1.91; 95% CI: 1.04-3.52),

neuroimaging alone does not exhaust the diagnostic assessment, as 48% of eyes exhibit RNFL thinning despite normal MRI.

The stability of longitudinal slopes suggests that structural damage in adults represents a stable event, likely resulting from regressed OPGs or primary NF1-related neuronal manifestations rather than an active

neurodegenerative process.

Consequently, MRI-negative patients with abnormal OCT may not require aggressive radiological

monitoring; instead, further research is warranted to determine how specific genetic variants and clinical phenotypes influence these structural retinal alterations.

43. **Monoallelic variants in *UCHL1* are an emerging cause of autosomal dominant optic atrophy (ADOA)**

Claudio Fiorini^1^^#^ ^#^These authors contributed equally, Giada Capirossi^1#^, Chiara La Morgia^1,2^, Xavier Dieu^3^, Federico Sadun^4^, Maria Lucia Cascavilla^5^, Marco Battista^5^, Lorenzo Bianco^5^, Piero Barboni^5^, Elisabetta Racano^6^, Fiorenza Soli^6^, Enza Maria Valente^7,8^, Ilaria Palmieri^8^, Neringa Jurkute^9^, John Britton^9^, Patrick Yu-Wai-Man^9^, Danara Ormanbekova^1^, Flavia Palombo^1^, Eleonora Pizzi^1^, Valentina Del Dotto^2^, Valerio Carelli^1,2^, Alessandra Maresca^1^^#^, Patrizia Amati-Bonneau^3,10^^#^, Leonardo Caporali^1^#^^

^1^IRCCS Istituto delle Scienze Neurologiche di Bologna, Programma di Neurogenetica, Bologna, Italy; ^2^Department of Biomedical and NeuroMotor Sciences (DIBINEM), University of Bologna, Italy; ^3^Université d’Angers, Centre National de la Recherche Scientifique (CNRS 6015), Institut National de la Santé et de la Recherche Médicale (INSERM U1083), Unité Mixte de Recherche (UMR) MITOVASC, 49000 Angers, France; ^4^Ospedale Oftalmico Roma, Rome, Italy; ^5^IRCCS Ospedale San Raffaele, Milan, Italy; ^6^Azienda provinciale per i servizi sanitari di Trento, Trento, Italy; ^7^Department of Molecular Medicine, University of Pavia, Pavia, Italy; ^8^Neurogenetics Research Center, IRCCS Mondino Foundation, Pavia, Italy; ^9^Moorfields Eye Hospital NHS Foundation Trust, London, UK; ^10^Département de Biochimie et Biologie Moléculaire, Centre Hospitalier Universitaire d’Angers, 49933 Angers, France.

The ubiquitin carboxyl-terminal hydrolase isozyme L1 (UCH-L1) is a deubiquitinating enzyme highly expressed in neurons, accounting for approximately 1-2% of total cytosolic proteins. It plays a key role in neuronal homeostasis and has been implicated in brain injury responses and neurodegenerative disorders. Biallelic *UCHL1* variants have been associated with a complex childhood-onset neurodegenerative phenotype characterized by spastic paraplegia, cerebellar ataxia, optic atrophy, and peripheral neuropathy. More recently, several pedigrees were identified with autosomal dominant spastic paraplegia and/or cerebellar ataxia and optic atrophy due to heterozygous *UCHL1* loss-of-function variants.

We performed NGS screenings in an international cohort of patients with inherited optic neuropathies (ON), after exclusion of pathogenic variants in known ON-associated nuclear genes and mitochondrial DNA. Candidate genes were prioritized, then fibroblasts derived from three affected individuals from two unrelated pedigrees were used for functional validation of the molecular findings and the pathogenetic mechanisms.

We identified 23 patients from 14 unrelated pedigrees harboring heterozygous *UCHL1* variants. All individuals presented with isolated dominant optic atrophy (DOA), predominantly affecting the temporal sector and papillomacular bundle, without additional neurological features. Age at onset ranged from childhood to adulthood, although in some cases it could not be precisely determined. Most variants were, as expected, loss-of-function, but we also report for the first time missense *UCHL1* variants associated with autosomal dominant disease, namely p.(Pro10Leu), p.(Leu17Pro), and p.(Leu95Arg). In addition, one patient carried a deletion encompassing exons 1–6, detected by MLPA analysis.

Patient-derived fibroblasts showed a marked reduction of UCH-L1 protein levels and, given previous evidence linking UCH-L1 deficiency to mitochondrial impairment, we investigated key components of mitochondrial function. While mitochondrial dynamics and respiratory function appeared preserved, we observed a trend toward reduced levels of OXPHOS complex subunits, especially significant for complex I. No significant changes were detected in extracellular lactate levels or mitochondrial DNA copy number. Interestingly, we also observed a tendency toward reduced expression of CRYAB, a mitochondrial chaperone recently implicated in autosomal dominant subacute optic neuropathy.

In conclusion, our findings expand the phenotypic spectrum associated with *UCHL1* variants to include isolated DOA and support the inclusion of *UCHL1* in diagnostic gene panels for optic neuropathies. Although the putative mitochondrial dysfunction appears subtle, the reduction in complex I levels may suggest a contributory role in disease pathogenesis.

44. **An unusual case of myelin oligodendrocyte glycoprotein optic neuritis with disc edema and prepapillary infiltration**

Catharina Mönig^1^, Walid Bouthour^1^

^1^Department of Ophthalmology, Geneva University Hospitals, Geneva, Switzerland

**Introduction**: Acute optic disc edema in myelin oligodendrocyte glycoprotein optic neuritis (MOG-ON) is common, as inflammation often involves the anterior, intraorbital part of the optic nerve. Other ophthalmological manifestations of MOG-associated disease are less frequent and may include uveitis and neuroretinitis. Here, we present an unusual case of MOG-ON with optic nerve head and prepapillary infiltration.

**Case Report**: A 78-year-old white woman presented with a five-day history of painless, blurred vision of her left eye. Her past medical history was notable for presumed left optic neuritis twenty-five years prior with full and spontaneous recovery, and right optic neuritis twenty years prior and residual right visual acuity of 20/30. She had no other symptoms and was not on any treatment. On examination, best-corrected visual acuity was 20/30 in each eye. Color plates were 10/11 in the right eye and 9/11 in the left eye. A relative afferent pupillary defect was present in the left eye. Automated visual field testing was normal in the right eye and showed diffuse depression in the left eye. Slit-lamp examination was unremarkable, and fundus examination revealed mild pallor of the right optic disc and high grade optic disc oedema in the left eye with an ill-defined infiltrative appearance and flame haemorrhages. The rest of her neurological and systemic examination was unremarkable. Optical coherence tomography of the optic discs showed left optic disc edema with prepapillary infiltration. Fluorescein angiography showed late left optic disc dye leakage and no vasculitis. Magnetic resonance imaging (MRI) of the brain and orbits with and without contrast revealed bilateral optic nerve sheath enhancement but no optic nerve hyperintensity nor enhancement, and no other demyelinating lesion. A lumbar puncture was performed and cerebrospinal fluid analysis demonstrated normal white cell count, moderately elevated protein, and no oligoclonal bands. Laboratory testing was positive for MOG antibodies (1:100) on cell-based assay and negative for aquaporin-4 antibodies. The patient was treated with high-dose intravenous methylprednisolone. Optic disc edema and the prepapillary infiltrate resolved. Her visual acuity in the left eye unfortunately worsened to 20/50 and remained unchanged at eight-month follow-up.

**Discussion**: Our patient had acute left MOG-ON with optic disc edema and atypical prepapillary infiltrate without uveitis, vasculitis, or neuroretinitis. Optic disc edema and prepapillary vitreous opacities were described in Behçet’s panuveitis, varicella-zoster virus optic nerve infiltration, leukemia, and neuroretinitis. Preretinal vitreous cells can sometimes be seen in high-grade papilledema but they are usually sparse, less dense, and less circumscribed. In our patient, MOG-related inflammation presumably affected the left optic disc and spared the intraorbital portion of the optic nerve. The prepapillary infiltrate is likely inflammatory in nature, as it resolved with corticosteroid treatment.

**Disclosures**: None.

46. **A not-so-luxurious darkness**

Covadonga Menendez Acebal^1^, Elena Brotons Muñoz^1^, Pau Marjalizo^1^, Pau Otal Aran^1^, Rafel Alcubierre Bailac^1^, Anna Camos Carreras^1^, Bernardo Sánchez Dalmau^1^

^1^Ophthalmology Department, Hospital Clínic de Barcelona, Barcelona, Spain

**Introduction**: Unilateral optic disc oedema represents a diagnostic challenge. A thorough history assessment and ophthalmological exploration are required to reach a successful evaluation that allows for a correct management of the patient.

**Case Report**: A 71-year-old male presents to the emergency room with sudden, unilateral, altitudinal and painless vision loss on his right eye (OD). He had suffered a few episodes of fluctuating vision loss on the same eye that lasted seconds during the previous four days. At the exploration, he showed a visual acuity of 20/400, a relative afferent pupillary defect (RAPD), and a loss of his inferior visual field on his OD. On fundus examination, an oedema on the superior half of his papilla could be observed together with temporal peripapillary telangiectasia, as well as an elevation of the macula around the papillomacular bundle. On the OCT exam, the retinal nerve fibre layer (RNFL) was elevated, and intraretinal fluid was found in the papillomacular bundle together with foveal subretinal fluid. The differential diagnosis included inflammatory, infectious and ischaemic causes, mainly neuroretinitis, optic neuritis and anterior ischaemic optic neuropathy (AION). Serum inflammatory markers were within normal range, and results were negative for bartonella, syphilis and other infectious diseases. A brain and orbit MRI scan were taken that showed no gadolinium enhancement, and a fluorescein angiography was performed that showed leakage of contrast through the swollen segment of the optic nerve head and peripapillary area, but no macular leakage. Serum myelin oligodendrocyte glycoprotein (MOG) and aquaporin-4 (AQP-4) antibody titres were negative. After much testing, it was finally oriented as luxury perfusion from a non-arteritic AION (NA-AION).

**Discussion**: This case is an example of the complex differential diagnosis that entails unilateral papillary oedema, with multi-modal tests needed to exclude alternative diagnoses –especially if the case exceeds the classical parameters of the disease. Luxury perfusion represents a self-regulatory, vascular reaction to the ischaemia from the AION. It involves increased vascular permeability that translates as dilated and tortuous peripapillary vessels, that may be accompanied by oedema in the papillomacular bundle. It is important to distinguish this entity from infectious and inflammatory causes –especially neuroretinitis, as the management of each differs greatly.

**Disclosures**: None.

47. **Lenadogene nolparvovec gene therapy for Leber Hereditary Optic Neuropathy in the real-life setting**

Catherine Vignal-Clermont^1^, Chiara La Morgia^2^, Valerio Carelli^2^, Patrick Yu-Wai-Man^3^, Mark L. Moster^4^, Robert C. Sergott^4^, Sarah Thornton^4^, Sean P. Donahue^5^, Hélène Dollfus^6^, Thomas Klopstock^7^, Claudia Priglinger^8^, Rabih Hage^9^, Vasily Smirnov^10^, Catherine Cochard^11^, Marie-Bénédicte Rougier^12^, Emilie Tournaire-Marques^12^, Pierre Lebranchu^13^, Caroline Froment^14^, Frédéric Pollet-Villard^15^, Marie-Alice Laville^16^, Claudia Prospero Ponce^17^, Scott D. Walter^18^, Francis Munier^19^, Pauline Zoppe^20^, Magali Taiel^21^, José-Alain Sahel^22^

^1^Service de Neuro-ophtalmologie et d’Urgences Ophtalmologiques, Hôpital Fondation Rothschild, Paris, France; ^2^IRCCS Istituto delle Scienze Neurologiche di Bologna, Programma di Neurogenetica, Bologna, Italy; ^3^John van Geest Centre for Brain Repair and MRC Mitochondrial Biology Unit, Department of Clinical Neurosciences, University of Cambridge, Cambridge, UK; ^4^Departments of Ophthalmology and Neurology, Wills Eye Hospital and Thomas Jefferson University, William H. Annesley, Jr., EyeBrain Center, Philadelphia, PA, US; ^5^Department of Ophthalmology Neurology, and Pediatrics, Vanderbilt University, and Vanderbilt Eye Institute, Vanderbilt University Medical Center, Nashville, TN, US; ^6^Institut de Génétique Médicale d’Alsace, Les Hôpitaux Universitaires de Strasbourg, Strasbourg, France; ^7^Department of Neurology, Friedrich Baur Institute, Ludwig Maximilians University Hospital, LMU Munich, Germany; ^8^Department of Ophthalmology, Ludwig Maximilians University Hospital, LMU Munich, Germany; ^9^Centre Hospitalier National D’Ophtalmologie des Quinze Vingts, Paris, France; ^10^Service Explorations de la Vision et Neuro-Ophtalmologie, CHU de Lille, Lille, France; ^11^Service d’Ophtalmologie, CHU de Rennes, Rennes, France; ^12^Service d’Ophtalmologie, Groupe Hospitalier Pellegrin, CHU de Bordeaux, Bordeaux, France; ^13^Service d’Ophtalmologie, CHU de Nantes, Nantes, France; ^14^Service de Neuro-Cognition et Neuro-Ophtalmologie, Hôpital Pierre Wertheimer, Hospices Civils de Lyon, Lyon, France; ^15^Service d’Ophtalmologie, Centre Hospitalier de Valence, Valence, France; ^16^Service d’Ophtalmologie, CHU de Caen-Normandie, Caen, France; ^17^Texas Tech University Health Sciences Center, El Paso, TX, US; ^18^Department of Ophthalmology, Hartford Hospital, Hartford, CT, US; ^19^Service d’Ophtalmologie, Hôpital Ophtalmique Jules-Gonin, Lausanne, Switzerland; ^20^Centre Hospitalier de Wallonie Picarde, Tournai, Belgium; ^21^GenSight Biologics, Paris, France; ^22^Department of Ophthalmology, University of Pittsburgh School of Medicine, Pittsburgh, PA, USA

**Introduction**: Early access programs (EAP) provide efficacy and safety data on the use of lenadogene nolparvovec (LN) gene therapy in real-life conditions in patients with Leber hereditary optic neuropathy (LHON) caused by the m.11778G>A *ND4* variant.

**Methods**: LN was provided based on patient or physician requests, and its use was authorized by local/national regulations in 4 countries (France, Italy, UK and US). Patients received LN as a unilateral or bilateral intravitreal injection. Baseline characteristics, efficacy and safety data were collected.

**Results**: A total of 63 *ND4-*LHON patients received LN in EAPs, mainly in France (35 [55.6%]) and the US (18 [28.6%]). Most (42 [66.7%]) patients received bilateral injections, all but one on the same day. At the time of the first injection, patients were on average (SD) 33.7 (+/-16.6) years old, with 6 (9.5%) children aged 13 or 14 years and 6 patients over the age of 60. The mean (SD) duration of disease at the first injection was 11.4 (+/-9.5) months. Most (84.1%) patients were treated with idebenone at or after LN injection. Mean (SD) best-corrected visual acuity (BCVA) at baseline was 1.74 (+/-0.63) LogMAR. Follow-up ranged from 1 to 49 months, with a mean of 24.5 (11.4) months. The mean change in BCVA from nadir reached -0.42 (+/-0.54) LogMAR (+21 ETDRS letters) at 1 year and -0.48 (+/-0.59) LogMAR (+24 ETDRS letters) at the last observation. An improvement of at least -0.3 LogMAR (+15 ETDRS letters) from nadir was observed in 74.6% of patients at the last observation. The safety of LN was comparable to those of the 189 patients from clinical trial studies, with no difference in the incidence of intraocular inflammation between patients treated bilaterally and unilaterally (51.2% vs 52.4%).

**Discussion**: Injection of LN in the real-life setting was associated with a clinically meaningful improvement in visual acuity from nadir and a favorable safety profile.

**Disclosures**: CVC is a consultant for GenSight Biologics and Chiesi. CLM is consultant for Gensight Biologics, Chiesi, Regulatory PharmaNet, Thenewway srl and First Class srl and has received financial support from Gensight Biologics, Chiesi, Regulatory PharmaNet, Thenewway srl and First Class srl. VC is a consultant for GenSight Biologics, Chiesi, and Pretzel Therapeutics. PYWM is a consultant for GenSight Biologics, Chiesi and Neurophth Therapeutics. MLM is a consultant for GenSight Biologics and has received research support from GenSight Biologics. RCS is a consultant for GenSight Biologics. TK received research support and/or personal compensation for presentations and/or consulting from GenSight Biologics and Chiesi Farmaceutici. MT is an employee of GenSight Biologics. JAS is a consultant for Avista Therapeutics, Essilor Luxottica and Lightstone ventures; is owner/co-owner/founder/co-founder and has personal financial interests (stock/stock options) in Gensight Biologics, Sparing Vision, Prophesee, Tilak Healthcare, Avista, Tenpoint, SharpEye, Cilensee and Netramind; is a member of the scientific advisory board of Gilbert Foundation, Foundation Fighting Blindness and Institute of Ophthalmology Basel; is an observer at Sparing Vision and Avista; is president of Fondation Voir et Entendre and StreetLab; is director of GenSight Biologics; is executive Vice-President of UPMC Enterprises; has patents for allotopic expression, Rod-Derived Cone Viability Factor and related patents; is a recipient for patent royalties and GenSight Biologics.

48. **Utility of propensity score weighting to improve external comparator matching in the LEROS study of idebenone efficacy in Leber Hereditary Optic Neuropathy**

Daniela Garavaglia^1^, Nancy J. Newman^2,^ Patrick Yu-Wai-Man^3-6^, Alfredo A. Sadun^7^, Valerio Carelli^8,9^, Rod Gossen^10^, Karen Sison Lance^10^, Caroline Fradette^10^, Xavier Llòria^1^, Thomas Klopstock^11-13^

^1^Chiesi Farmaceutici S.p.A, Parma, Emilia-Romagna, Italy; ^2^Departments of Ophthalmology, Neurology, and Neurological Surgery, Emory University School of Medicine, Atlanta, GA, USA; ^3^John van Geest Centre for Brain Repair, and MRC Mitochondrial Biology Unit, Department of Clinical Neurosciences, University of Cambridge, Cambridge, UK; ^4^Cambridge Eye Unit, Addenbrooke’s Hospital, Cambridge University Hospitals, Cambridge, UK; ^5^Moorfields Eye Hospital NHS Foundation Trust, London, UK; ^6^Institute of Ophthalmology, University College London, London, UK; ^7^Doheny Eye Institute, Department of Ophthalmology, University of California, Los Angeles, CA, USA; ^8^IRCCS Istituto di Scienze Neurologiche di Bologna, Bologna, Italy; ^9^Department of Biomedical and Neuromotor Sciences, University of Bologna, Bologna, Italy; ^10^Chiesi Canada Corp. Woodbridge, Ontario, Canada; ^11^Friedrich Baur Institute at the Department of Neurology, LMU University Hospital, LMU Munich, Munich, Germany; ^12^German Center for Neurodegenerative Diseases (DZNE), Munich, Germany; ^13^Munich Cluster for Systems Neurology (SyNergy), Munich, Germany.

**Introduction**: External comparator designs are increasingly used in rare diseases such as Leber hereditary optic neuropathy (LHON), where randomized trials may be impractical. In LEROS (ClinicalTrials.gov NCT02774005), long-term idebenone (IDE) efficacy was compared with outcomes from an untreated, external natural history (NH) cohort. The matching of cohorts (based solely on time since onset of symptoms [OoS]) was statistically significant but left residual baseline imbalances. This post-hoc analysis evaluated whether propensity score weighting could strengthen cohort comparability and provide a more reliable framework for assessing IDE efficacy.

**Methods**: Untreated patients from NH studies (case record survey [CRS]-1 and CRS-2) and patients in the LEROS IDE cohort were eligible for matching if they had a baseline and ≥1 post-baseline visual acuity (VA) assessment, and an m.11778G>A, m.3460G>A, or m.14484T>C mitochondrial DNA (mtDNA) variant. For NH patients, baseline was the first VA assessment following diagnosis.

Stabilized inverse probability of treatment weights derived from a propensity score model were used to match the baseline characteristics and demographics of the NH and IDE cohorts in a synthetic weighted sample. Prognostic covariates used in the model included: sex, age at onset, time since OoS at baseline, baseline best-corrected VA in the better-seeing eye (best BCVA), and mtDNA variant. Patients with missing data for these variables were excluded. Baseline characteristics and demographics were assessed across propensity score weighted (PSW) and unweighted groups. In PSW groups, results were calculated according to patient weights rather than patient numbers.

**Results**: In total, 148 of 199 (82%) LEROS IDE patients, and 159 of 601 (26%) NH control patients were included. At baseline in the respective unweighted groups, mean (standard deviation [SD]) age was 33.6 (14.8) and 29.4 (15.3) years, 73.0% and 82.4% were male, mean (SD) time since OoS was 18.4 (15.8) and 19.5 (70.3) months, mean (SD) best BCVA was 1.29 (0.61) and 0.96 (0.61) logMAR, and the mtDNA variant distribution was 60.8% and 71.1% m.11778G>A, 19.6% and 18.2% m.3460G>A, 19.6% and 10.7% m.14484T>C. Demographics and baseline characteristics were well matched across the PSW LEROS IDE (N=149.7) and NH control (N=158.5) groups. At baseline, the respective weighted mean (SD) age was 31.4 (14.2) and 31.5 (15.9) years, 77.4% and 77.5% were male, mean (SD) time since OoS was 19.3 (16.2) and 20.0 (64.6) months, mean (SD) best BCVA was 1.09 (0.65) and 1.11 (0.64) logMAR, and the mtDNA variant distribution was 66.6% and 66.4% m.11778G>A, 18.5% and 17.8% m.3460G>A, 14.9% and 15.7% m.14484T>C.

**Discussion**: Compared with the LEROS matching algorithm, use of PSW improves alignment of baseline characteristics and demographics between the LEROS IDE and NH cohorts, mitigating potential biases in evaluating the efficacy of IDE and highlighting the importance of bias-reducing statistical techniques in rare-disease research.

**Disclosures**: DG and XL are employees of Chiesi Farmaceutici S.p.A. NJN has been a consultant for Chiesi, GenSight, Stoke, Phelcom, and Eli Lilly. PYWM received research support and consultation fees from Chiesi, GenSight Biologics, Stoke Therapeutics, and PYC Therapeutics. AS has been a consultant for Chiesi and GenSight Biologics and has received research support from GenSight Biologics and Stealth Therapeutics. VC and TK received research support and consultation fees from Santhera Pharmaceuticals, Chiesi, and GenSight Biologics. RG, KSL, and CF are employees of Chiesi Canada Corp. Medical writing support was provided by Cactus Life Sciences, and funded by Chiesi Farmaceutici S.p.A.

49. **A retrospective longitudinal cohort study describing visual outcomes in patients with paediatric optic nerve gliomas and identifying predictors of poor visual outcomes.**

Divya Agrawal^1,2^, Emma Kerr^1^, Brinda Muthusamy^1^

^1^Cambridge University Hospitals NHS Foundation Trust, Cambridge, UK; ^2^Cambridge Medical School, Cambridge, UK

**Introduction**: Optic nerve gliomas are rare paediatric tumours associated with neurofibromatosis type 1 (NF1). This retrospective review evaluates the outcomes of patients with NF1, since there is limited data on long term visual outcomes and prognostic indicators of optic nerve gliomas.

**Methods**: A retrospective review between 2014 to 2025 of paediatric patients with gliomas identified 80 children of whom 25 had an optic nerve glioma. Data collected included initial MRI findings, the presence or absence of underlying NF1, strabismus, nystagmus. Immunohistochemistry was reviewed. Management pathways were analysed, including craniotomy, ventriculoperitoneal (VP) shunt and chemotherapy. Visual outcomes were assessed using best-corrected visual acuity (BCVA, logMAR), visual fields, Optical Coherence Tomography (OCT) retinal nerve fibre layer (RNFL) thickness, and ganglion cell layer (GCL) measurements where available. Statistical analysis reviewed whether NF1 status, strabismus, nystagmus or treatment with chemotherapy impacted final visual outcomes.

**Results:** Mean age of participants at presentation was 4.77. The mean follow-up time was 4.34 years. Participants were divided into three categories based on their visual acuity: ≥1.3, 0.3<x<1.3 and ≤0.3 logMAR units. For patients whose acuity was ≥1.0, the Freiburg visual acuity test was used to convert this to logMAR. These patients were not included in statistical analysis, as their acuity was not accurately measured. Patients with a visual acuity ≥1.3 at presentation lost three lines on average (+0.38). Those with a presenting visual acuity ≤0.3 had approximately stable visual acuity at final review (-0.07). Eighteen of the 25 patients had NF1. Of the 25 patients, 12 underwent biopsy, and 6 had a craniotomy and 18 had chemotherapy. VP shunts were inserted in eight patients. Immunohistochemistry showed GFAP positivity in 6 patients. Strabismus was present in fifteen patients and nystagmus in ten. 8 of the 15 patients that had visual field testing had abnormal visual fields.

The difference between the visual acuity of NF1 positive and negative patients was not significant (p =0.80). Two sample t-tests showed there was no significant difference in visual acuities of patients with and without nystagmus (p =0.67) or strabismus (p =0.41). No significant difference was found between patients treated with and without chemotherapy (p =0.17). Overall, the mean change in BCVA was +0.09 logMAR (ranging from –2.00 to +2.34).

Of the 11 patients with OCT imaging, mean RNFL thickness decreased from 86.9 µm at baseline to 71.7 µm at follow-up. Additionally, the mean change in RNFL and GCL per year was -4.50 µm and -0.54 µm respectively.

**Implications**: Overall, this study did not show that NF1 or presence of strabismus or nystagmus was associated with a poorer visual outcome. Additionally, there was not a statistically significant difference in visual outcomes between patients treated with and without chemotherapy.

51. **Beyond peripheral nerves: Bilateral optic neuropathy in Charcot-Marie-Tooth type 2A2**

Daniel Cardoso^1^, Lígia Ribeiro^1^, Dália Meira^1^

^1^Ophthalmology Department, Unidade Local de Saúde Gaia/Espinho, Vila Nova de Gaia, Portugal

**Introduction**: Charcot-Marie-Tooth diseases (CMT) are classically described as a group of clinically and genetically heterogeneous rare disorders (1:2500) of the peripheral nervous system. The autosomal dominant form, CMT type 2A (CMT2A), is most commonly caused by mutations in the *MFN2* gene and is increasingly recognized to include expanded phenotypes such as optic neuropathy, which represents a diagnostic and therapeutic challenge due to its phenotypic variability. This case details the progressive visual decline in a patient with genetically confirmed CMT2A2.

**Case Report**: A man in his early twenties with known CMT2A2 (MFN2 mutation) presented with acute, painless bilateral visual loss. Initial examination revealed reduced best-corrected visual acuity (BCVA), temporal optic disc pallor, and a temporal visual field defect, prompting magnetic resonance imaging to exclude a compressive lesion, which was normal. Visual evoked potential testing confirmed bilateral optic nerve dysfunction. Genetic testing for Leber’s hereditary optic neuropathy (LHON) was negative. Over ten years, he experienced progressive bilateral decline: BCVA worsened from 1.0 to 0.3 (right eye) and 1.0 to 0.16 (left eye). Visual fields evolved into cecocentral scotomas. Optical coherence tomography showed progressive thinning of the temporal and global retinal nerve fiber layer and ganglion cell complex. A therapeutic trial with idebenone (600mg/day) provided no benefit.

**Discussion**: This case illustrates progressive bilateral optic neuropathy as a major manifestation of CMT2A2. Mitofusin 2, encoded by *MFN2*, is essential for mitochondrial fusion and axonal transport. Optic nerve involvement aligns with the vulnerability of energy-dependent neuronal pathways to mitochondrial dysfunction The phenotype of progressive visual acuity loss with cecocentral scotomas represents one end of a variable spectrum that may also include severe peripheral field loss with relative central sparing. Such presentations can mimic other optic neuropathies, making exclusion of compressive lesions and LHON essential. The lack of response to idebenone underscores a distinct pathophysiology from primary mitochondrial disorders, highlighting the need for alternative neuroprotective strategies. This report underscores the importance of regular ophthalmological surveillance in patients with CMT2A, particularly those carrying *MFN2* mutations, to enable early detection and management of visual impairment.

**Disclosures**: The authors have no conflicts of interest or financial disclosures to report.

52. **Isolated optic neuropathy in Bosch–Boonstra–Schaaf syndrome due to a novel NR2F1 variant**

David Alves^1^, Ana Faria-Pereira^1^, Olinda Faria^1^, Sérgio Estrela Silva^1,2^

^1^Department of Ophthalmology, Unidade Local de Saúde de São João, Porto, Portugal; ^2^Department of Surgery and Physiology, Faculty of Medicine, University of Porto, Porto, Portugal

**Introduction:** Bosch–Boonstra–Schaaf optic atrophy syndrome (BBSOAS) is a rare autosomal dominant neurodevelopmental disorder caused by pathogenic variants in the *NR2F1* gene. First described in 2014, approximately 200 cases have been reported worldwide to date. While classically associated with early-onset visual impairment due to optic nerve hypoplasia or atrophy, often accompanied by variable neurodevelopmental features, isolated ocular presentations may occur, leading to diagnostic delay.

We report a case of childhood-onset bilateral optic neuropathy associated with a novel *de novo NR2F1* variant.

**Case Report:** A 29-year-old woman, the first child of non-consanguineous parents, with no family history of visual or neurological disease, has been followed at our ophthalmology department since 3 years of age due to bilateral visual impairment. At that time, ophthalmological examination revealed a BCVA of 0.4 in both eyes, while fundus examination demonstrated bilateral optic disc pallor.

During follow-up, the patient underwent serial complementary diagnostic investigations. Automated visual fields (30-2) consistently showed inferior altitudinal defects bilaterally, with severe peripheral visual field constriction. OCT revealed marked global RNFL thinning and diffuse GCL loss.

VEP revealed reduced P100 amplitude (25–50% of normal) and prolonged latency, indicating optic pathway dysfunction. pERG showed preserved P50 waves with bilateral absence of the N95 component, consistent with bilateral optic nerve dysfunction. Color vision testing revealed a mild red–green axis defect on Hardy–Rand–Rittler plates.

Extensive laboratory investigations, including hematologic, metabolic, infectious, autoimmune (anti-AQP4 and anti-MOG), and nutritional studies, were unremarkable. Brain and orbital MRI demonstrated diffuse thinning of both optic nerves along their entire course to the optic chiasm, without additional abnormalities.

Given the suspicion of a hereditary optic neuropathy, next-generation sequencing (NGS) of a 15-gene optic neuropathy panel identified a heterozygous *NR2F1* variant (c.469C>G; p.Gln157Glu), not previously reported in literature. Bioinformatic analysis predicted a deleterious effect, and parental testing confirmed a *de novo* mutation.

**Discussion:** This case illustrates a mild, predominantly ocular phenotype of BBSOAS. Although typically described as a multisystem disorder, isolated optic neuropathy may represent the leading or sole manifestation, particularly in adulthood. The absence of systemic involvement highlights the need for increased clinical suspicion and comprehensive genetic testing in patients with early-onset, non-progressive optic atrophy.

Genotype–phenotype correlations in BBSOAS are increasingly recognized, with variants outside the DNA-binding domain often associated with milder presentations. Isolated optic nerve thinning on neuroimaging further supports the diagnosis in such cases. Identification of a de novo *NR2F1* variant has important implications for genetic counseling, prognosis, and family planning. This report reinforces the value of NGS in the diagnostic work-up of unexplained optic neuropathies and contributes to refining the expanding clinical and molecular spectrum of BBSOAS.

**Disclosures:** The authors declare no conflicts of interest.

**Abbreviations**: AQP4- Aquaporin-4; BCVA – Best Corrected Visual Acuity; GCL - Ganglion Cell Layer; MOG - Myelin Oligodendrocyte Glycoprotein; OCT – Optical coherence tomography; pERG- Pattern Electroretinogram; RNFL - Retinal Nerve Fiber Layer; VEP – Visual Evoked Potential

54. **Unilateral optic neuropathy resulting from vitreopapillary traction syndrome with concurrent epiretinal membrane**

Danijel Pericic^1^, Ari August^1^, Andrew Garfield^2^

^1^Wills Eye Hospital, Neuro-ophthalmology. Philadelphia, PA, USA; ^2^Cooper Medical School of Rowan University. Camden, NJ, USA

**Introduction**: Vitreopapillary Traction Syndrome (VPT) is a rare condition that results from incomplete vitreous separation from the optic nerve head, which can cause decreased axoplasmic flow and an irreversible optic neuropathy. Epiretinal membrane (ERM) is a distinct but related condition that also relates to vitreous abnormalities. An ERM can form when a posterior vitreous detachment (PVD) causes a migration of microglial cells through a disrupted internal limiting membrane (ILM) which then differentiate into fibroblasts and cause the formation of the membrane.

**Case Report**: A seventy-two-year-old Caucasian male presented with a 7-month history of an inferotemporal peripheral visual disturbance, as well as wavy vision in his right eye. The patient had a visual acuity of 20/60 in the affected eye, with an afferent pupillary defect, as well as diminished color vision on Ishihara color plates. A workup for GCA and other intracranial causes was normal with laboratory and MRI testing. Slit lamp exam showed mild, symmetric cataracts in both eyes, and a dense ERM with VPT in the right eye. A fluorescein angiogram showed diffuse mild vascular leakage and disc staining in the affected eye, but an inflammatory workup was negative as well. OCT testing showed cystic macular changes with a dense ERM extending from the macula to the nasal optic nerve, with the ERM applying severe vitreopapillary traction, causing optic disc elevation clinically on dilated fundoscopic exam, as well as thickening of the retinal nerve fiber layer (RNFL) and artificially thickened ganglion cell layer (GCL) due to the ERM. On several follow up visits over one year, his vision, OCT, and visual field testing remained stable. He was sent to a retina specialist for consideration of a pars plana vitrectomy (PPV) to relieve the VPT and to remove the ERM.

**Discussion**: The presence of both VPT and ERM coexisting has been previously described with reports of up to 44% of patients with ERM also exhibiting VPT. The presence of macular edema has also been reported in up to 22% of patients with ERM and has been associated with more advanced stages of ERM. This illustrates that there may be a pathological link to ERM and VPT, and that they may be on a similar spectrum of disease. Patients with VPT exhibit altered optic disc architecture and can be misdiagnosed with other causes of unilateral disc edema. Generally, the prognosis for VPT is quite good with observation alone, which is why it is critical that VPT is recognized, especially in the era of OCT, where the traction can be visualized, to avoid expensive and unnecessary workups for patients.

**Disclosures:**

Amgen Inc. – Consultant, speaker

Tarsus Pharmaceuticals – Consultant, speaker

55. **Rarity within rarity: Unilateral Leber’s Hereditary Optic Neuropathy**

Michele Carbonelli^1,2^, Anna Maria De Negri^3^, Stefania Bianchi Marzoli^4^, Giulia Amore^5^, Piero Barboni^2,6^, Marco Battista^6^, Luigi Brotto^7^, Catarina P. Coutinho^8^, Martina Romagnoli^5^, Valerio Carelli^1,5^, Chiara La Morgia^1,9^

^1^Dipartimento di Scienze Biomediche e Neuromotorie, Università di Bologna, Bologna, Italy; ^2^Studio Oculistico d’Azeglio, Bologna, Italy; ^3^Azienda Ospedaliera San Camillo-Forlanini, Rome, Italy; ^4^Neuro-Ophthalmology Center and Electrophysiology Laboratory, Department of Ophthalmology, IRCCS Istituto Auxologico Italiano, Milan, Italy; ^5^IRCCS Istituto delle Scienze Neurologiche di Bologna, Programma di Neurogenetica, Bologna, Italy; ^6^Department of Ophthalmology, University Vita-Salute, IRCCS Ospedale San Raffaele, Milan, Italy; ^7^Department of Clinical Science and Community Health, University of Milan, Milan, Italy; ^8^Department of Pharmacy and Biotechnology, University of Bologna, Bologna, Italy; ^9^IRCCS Istituto delle Scienze Neurologiche di Bologna, UOC Clinica Neurologica, Bologna, Italy

**Purpose**: Leber hereditary optic neuropathy (LHON) is a genetic disease caused by point variants in the mitochondrial DNA (mtDNA) that manifests as a subacute, rapid and severe loss of central vision affecting usually both eyes simultaneously or sequentially over a period of weeks or months. Unilateral involvement has been rarely reported in the literature and remains a puzzling feature. We thus describe the ophthalmologic and genetic characteristics of unilateral LHON cases, and their evolution over time to investigate the presence of structural and functional features that might protect the contralateral eye

**Methods**: We screened our cohort of over 400 LHON affected patients to identify those with unilateral disease with a follow-up of at least 2 years and without involvement of the fellow eye. Neurophthalmological evaluation included assessment of best corrected visual acuity (BCVA), computerized visual fields (MD), fundus exam and optical coherence tomography (OCT).

**Results**: We found five patients, from five unrelated Italian pedigrees, with monocular involvement (1,25%), four patients with the m.11778G>A/MT-ND4 variant and one patient with the rare m.14258G>A/MT-ND6 variant. Two of these patients presented a childhood onset (before 12 years old), two in adolescence and one had a late onset (after 40 years old). All five patients received idebenone therapy, 900 mg/day for the adults and 405 mg/day for the two children. All patients experienced subacute visual decline with a mean BCVA at the nadir of LogMar 1.4, reached around a time window of 6-12 months from onset (mean 8 months), and a mean BCVA of 0.5 LogMar at the last follow-up visit (mean 7.9 years, max-min 18.4-2 years) with a vision recovery in the 80% of cases. At last OCT, compared to the unaffected eye, the affected eye showed a mean disc area 0.16 mm^2^ smaller, a reduction in the mean peripapillary retinal nerve fiber layer (pRNFL) thickness of 24% (mean difference of 38 µm, 96 µm vs 116 µm) and a reduction in the mean temporal quadrant RNFL thickness of 56% (mean difference of 40,6 µm, 41 µm vs 72 µm). All patients showed a central defect at the visual field in the affected eye (mean MD -3,8) versus a clear visual field in the contralateral eye.

**Conclusions**: The acute conversion of LHON and its catastrophic evolution, often resistant to treatment during the subacute phase, remains largely unexplained. Unilateral onset represents an additional rare and poorly understood phenomenon; in our cohort it accounted for a small proportion of cases (around 1.5%) and may occur at any age. Preliminarily, no clearcut difference distinguishes the affected eyes from the fellow spared ones, but additional anatomical factors are currently under investigation.

**Disclosures**: None.

58. **I am not confused, I am just well mixed–The jar with double-negative (DN) optic neuritis**

Durga Priyadarshini Santhakumar^1^, Ambika Selvakumar^1^, Shikha Bassi Talwar^1^

^1^Department of Neuro ophthalmology, Sankara Nethralaya, Chennai, India

**Objective**: To understand and analyze the clinical profile and behaviour of double-seronegative optic neuritis patient who presented between June 2018 to December 2019.

**Study Design**: Retrospective analysis of patients with acute optic neuritis who presented within 4 weeks of onset, underwent MRI brain and orbit, tested negative for aquaporin-4 antibody (NMO Ab), myelin oligodendrocyte glycoprotein antibody (MOG Ab) and had at least one post-treatment follow-up.

**Results**: In this cohort, 59 eyes of 40 patients were analyzed. There was an equal distribution of male and female patients (50%). Bilateral involvement was noted in 19 patients (47.5%) and associated pain was noted in 22 patients (55%). Mean duration of vision loss was 15.75±12.5 days. Prodromal viral symptoms were noted in 10 patients (25%). Mean vision on presentation was log MAR 1.58± .868. Disc edema was noted in 22 eyes (37.3%). Magnetic resonance imaging of brain was abnormal with T2-hyperintense signals in 12patients (30%). Optic nerve T2-hyperintense signals were noted in 36 patients and thinning of the optic nerve was in 4 patients. Contrast enhanced study reports were available for 19 patients, of whom 16 patients had optic nerve enhancements. Long-segment posterior optic nerve involvement was noted in 15 patients, short-segment anterior optic nerve involvement in 21 patients, and perioptic nerve sheath involvement with fat stranding in 2 patients. The mean visual outcome at last follow-up was logMAR 0.347± 0.662. (P <0.0001). CNS symptoms were associated in 3 patients, 2 of whom had ADEM. Transverse myelitis was noted in 4 patients. The mean follow-up was 10.3 ±19.3 months. Recurrence was noted in 6 patients (15%). Residual visual deficit of logMAR 0.40 or worse at last follow-up was noted in 13 patients (32.5%). One patient who was initially DN during the study period was diagnosed with MOGAD, 5 years later following a relapse.

**Conclusion**: Double-negative optic neuritis shows no sex predilection with equal unilateral and bilateral presentations and painful attacks. Although vision loss on presentation was profound, our cohort showed statistically significant recovery at last follow-up visit. Recurrence is not uncommon and repeating the seromarker testing during relapse is vital. However, this group requires further long-term follow-up and reassessment of seromarkers in cases of relapse or new disease activity.

67. **Pediatric autosomal recessive LHON: A report of two cases**

Eva Roomets

Tallinn Children´s Hospital, Tallinn, Estonia

**Introduction**: Leber hereditary optic neuropathy (LHON) is a progressive optic neuropathy typically linked to mitochondrial DNA mutations. In 2021 an autosomal recessive form (arLHON) caused by biallelic mutations in the nuclear *DNAJC30* gene was identified by Stenton et al. Paediatric LHON (onset before the age of 12 years) remains rare and is characterized by a more variable clinical course and a potentially better visual prognosis than adult-onset cases. However, diagnosis in this age group is often more challenging and protracted, frequently leading to significant delays in clinical recognition.

**Case Reports**

**Case 1** An 11-year old girl presented with subacute vision loss and mild papilloedema in 2022. Brain MRI revealed T2 hyperintensity in the posterior segments of both optic nerves and the optic chiasm. As the remainder of the neuroimaging was normal, a presumptive diagnosis of chiasmal optic neuritis was made. Given the lack of treatment response and the absence of inflammatory markers, LHON was subsequently suspected. Mitochondrial DNA analysis was negative for the whole mtDNA sequencing. Subsequent nuclear DNA sequencing revealed a homozygous pathogenic variant in DNAJC30 (c.152A>G, p.Tyr51Cys). Idebenone treatment was initiated 4.5 months after the onset of symptoms and continued for two years. The patient demonstrated an excellent therapeutic response with significant visual recovery.

**Case 2** In 2013, an 8-year-old boy presented with acute vision loss and mild optic disc edema. At the time, brain MRI was interpreted as normal, and neurological, toxic, and nutritional causes were excluded, and the patient showed no clinical response to high-dose corticosteroid therapy. While LHON was clinically suspected, standard mitochondrial DNA testing was negative. The etiology remained unconfirmed until in 2024 clinical re-evaluation and targeted genetic testing identified a homozygous *DNAJC30* mutation (c.152A>G, p.Tyr51Cys). Retrospective re-evaluation of the initial MRI (performed in 2013) revealed T2 hyperintensity in the posterior segments of both optic nerves, consistent with the findings observed in Case 1. Despite the 11-year delay, idebenone treatment was started and continued for 12 months. Although the patient did not achieve clinically significant recovery of the visual acuity, the treatment showed a remarkable positive effect on visual fields.

**Discussion**: These cases highlight the importance of considering arLHON in children with acute or subacute vision loss, particularly when they are unresponsive to standard immunomodulatory treatments. While often unremarkable, the characteristic MRI finding in early-stage LHON is a subtle T2 optic nerve hyperintensity. This contrasts with the prominent swelling and intense enhancement typical of pediatric autoimmune inflammatory neuropathies. Consequently, these subtle MRI changes are easily overlooked by clinicians anticipating more robust inflammatory features.

These cases underscore the diagnostic necessity of sequencing the *DNAJC30* gene in parallel with mitochondrial DNA and re-evaluating old cases.

**Disclosures** The author declares no conflicts of interest.

68. **Optic disc edema in juvenile macular dystrophy**

Francesca Allegrini

Department of Ophthalmology, Hospital IRCCS Sacro Cuore Don Calabria - Negrar (VR), Italy

**Introduction**: Juvenile macular dystrophy is a group of rare, inherited retinal degeneration diseases which affects children and young adults. Stargardt disease is the most common type and causes severe macular involvement. Progressive central vision loss is the main feature of the disease and may hide other ocular conditions. This case report describes a singular association between juvenile macular dystrophy and idiopathic intracranial hypertension.

**Case Report**: A 20-year-old man was referred to our Ophthalmology Department complaining constant mild headache in the last week. No changes in vision were observed, and no other symptoms were reported. The patient was known to be affected by inherited macular dystrophy clinically compatible with Stargardt disease. At our examination vision of 20/200 OU was reported, unchanged in comparison to the last visit. Impaired colour vision was detected, while confrontational visual field test showed bilateral central scotomas, consistent with his retinal disease. Intraocular pressures were 11 mmHg OD and 9 mmHg OS. Fundus examination revealed onset of bilateral optic disc edema, not present at previous examinations. Central macular atrophy surrounded by flecks-like deposits in both eyes was stable. Optical Coherence Tomography (OCT) documented elevated optic discs associated with Peripapillary Hyperreflective Ovoid Mass-like Structures (PHOMS). Ocular ultrasonography was negative for optic disc drusen. Magnetic Resonance Imaging (MRI) and Magnetic Resonance Angiography (MRA) of the brain excluded structural signs and venous sinus thrombosis, although signs consistent with idiopathic intracranial hypertension were detectable. The lumbar opening pressure confirmed the suspect, with a value of 35 cm H_2_O. He was finally diagnosed with Idiopathic Intracranial Hypertension associated with Stargardt Disease. After a therapy with Acetazolamide 250 mg twice a day he reported improvement of headache’s episodes and optic disc edema reduced in the following months.

**Discussion**: Juvenile macular dystrophy is a group of rare, inherited juvenile macular degeneration diseases which affects children and young adults. Severe central vision loss, dyschromatopsia and central vision scotomas are early manifestations of juvenile macular dystrophy and may occult other ocular conditions. For this reason, careful attention should be given to the onset of other symptoms, like headache, which may be suspicious of concomitant disease’s conditions. At our knowledge only one other case report described the association between Stargardt disease and idiopathic intracranial hypertension.

**Disclosures**: No financial disclosures.

69. **Silent retinal neurodegeneration in multiple sclerosis: Minimum rim width (MRW) as a possible early marker of progressive neuronal loss**

Francesca Pilati^1^, Francesca Bosello^2^, Rocco Micciolo^3^, Emilio Pedrotti^2^, Chiara Zaffalon^2^, Damiano Marastoni^2^, Polinelli^4^, Matilde Zambon^2^

^1^AOUI Verona, Verona, Italy; ^2^University Hospital of Verona, Verona, Italy ; ^3^University of Trento, Trento, Italy; ^4^IRCCS Ospedale Sacro Cuore Don Calabria, Negrar di Valpolicella, Italy

**Introduction**: Neurodegeneration occurs early in multiple sclerosis (MS) and represents a main mechanism of disability accumulation, even in relapsing–remitting MS (RRMS). Optical coherence tomography (OCT) allows in vivo assessment of retinal neuroaxonal integrity and can detect subclinical neurodegeneration, especially in eyes without a history of optic neuritis (ON). The OCT parameters most commonly studied in relation to neurodegenerative deterioration are the peripapillary retinal nerve fiber layer (pRNFL), the ganglion cell layer (GCL), and the inner plexiform layer (IPL). We aimed to investigate minimum rim width (MRW) as a potential earlier biomarker of retinal neurodegeneration in RRMS.

**Methods**: Seventy-two patients with RRMS (49 females, 23 males; 144 eyes) were longitudinally evaluated. Functional and structural assessments were performed at baseline (within the first year after MS diagnosis) and follow-up (2.8 ± 1.4 years after MS diagnosis). Functional measures included high- and low-contrast visual acuity, Pelli–Robson contrast sensitivity, and Ishihara test. Structural OCT parameters (MRW, pRNFL, GCL, IPL) were recorded using spectral-domain OCT (Heidelberg Engineering).

Primary analysis compared all eyes baseline and follow-up measurements. Secondary analysis stratified eyes into three subgroups: group A (eyes of non-neuritic patients), group B (neuritic eyes of neuritic patients), and group C (non-neuritic eyes of neuritic patients).Paired Student’s *t*-tests were used for statistical analysis.

**Results**: Primary analysis showed significant reductions in global and sectoral MRW (p < 0.01), RNFL N (p < 0.05), RNFL T (p < 0.05), RNFL TI (p < 0.01), and global GCL thickness (p < 0.05). Global pRNFL (p = 0.125), while functional visual measures remained stable.

In group A, thinning of global MRW (p = 0.0001) and all MRW sectors (p < 0.05) was significant and more pronounced than RNFL. A significant focal RNFL reduction in temporal and temporal-inferior sectors was observed in the total group and confirmed in the groups A and B (p < 0.05).Overall cohort analysis revealed significant GCL thinning in temporal, superior, and inferior macular areas (p < 0.05) and IPL reduction in the nasal sector (p < 0.05). In group A, both GCL and IPL thinning were confirmed, notably in the temporal sector (p < 0.001). In group B, IPL reduction in the nasal sector was also significant (p < 0.05), confirming overall findings.

**Discussion**: OCT represents a valuable tool for detecting subclinical and early neurodegeneration in MS. MRW appears to be a more sensitive and earlier indicator of optic nerve head neuroaxonal damage than pRNFL, particularly in eyes without previous ON, where it may capture early neurodegenerative processes.

The concomitant thinning of macular GCL-IPL further supports the presence of chronic, subclinical retinal neurodegeneration. Our findings suggest that MRW may represent a valuable biomarker for monitoring silent progression in MS, even in patients without clinically evident optic neuritis.

**Disclosures**: No financial disclosures to declare

70. **Functional–structural dissociation in hereditary optic neuropathies**

Gabriella Cammarata^1^, Lisa Melzi^1^, diletta Santarsiero^1^, Alessandra Criscuoli^1^, Virginia Raccagni^1^, Stefania Bianchi Marzoli^1^

^1^Neuro-Ophthalmology Center and Electrophysiology Laboratory, Department of Ophthalmology, IRCCS, Istituto Auxologico Italiano – Milan, Italy

**Introduction**: Hereditary optic neuropathies cause dysfunction of retinal ganglion cells (RGC) and their axons leading to abnormal pattern visual evoked potential (VEP) responses.

Minimal VEP changes are uncommon but may be observed in asymptomatic genetic carriers or in the very early preclinical phase, prior to significant RGC loss. Once the disease becomes clinically manifest, VEP recording shows reduced amplitude and prolonged latency. We aimed to describe a subset of patients with genetically confirmed chronic hereditary optic neuropathy exhibiting a clear clinical phenotype and significant structural damage yet showing minimal VEP responses abnormalities.

**Methods**: In this retrospective observational case series fourteen patients evaluated at the Neuro-Ophthalmology Center, IRCCS Istituto Auxologico Italiano, Milano, Italy, between 2023 and 2025 were included. The cohort comprised 9 patients with autosomal dominant optic atrophy (ADOA; 4 males, 5 females; mean age 40±15 SD years; 4 patients with Leber hereditary optic neuropathy (LHON; 2 males, 2 females; mean ± SD: age: 54±12 years), and 1 female patient with AFG3L2-related optic neuropathy (age 45 years).

All patients underwent complete neuro-ophthalmologic evaluation and Spectral -domain optical coherence tomography (SD-OCT; Heidelberg Engineering). Molecular diagnosis was confirmed in all cases. All patients had a clinically manifest phenotype for a mean disease duration of 7 years at the time of the electrophysiological testing.

Pattern electroretinogram (PERG 30° and 15°) and pattern-reversal VEP (60’ and 15’) were recorded according to ISCEV standards in all patients.

**Results**: BCVA was moderately reduced (mean ± SD: 0.8 ± 0.3). A cecocentral or temporal scotoma was observed in all patients, associated with a moderately reduced MD value (mean ± SD: −5 ± 7 dB). Reduced retinal nerve fiber layer (RNFL) thickness and macular ganglion cell layer (GCL) thickness were detected in all patients (mean ± SD: 75 ± 14 µm and 26 ± 9 µm, respectively).

PERG recording showed irregular waveforms with frequently poorly defined N95 components. P50 and N95 amplitude was slightly reduced at 30° and 15° (mean ± SD: 6±3 µV and 7±2 µV, respectively, and 3±1 µV and 4±1 µV, respectively). N95/P50 ratio at 30° and at 15° was calculated (mean ± SD: 1.0± 0.3 and 1± 0.8, respectively).

VEP waveforms were morphologically regular. P100 latency and amplitude at 60’ and 15’ were minimally abnormal (mean ± SD: 100± 19 ms and 11± 4 µV, respectively, and 115±9 ms and 9±4 µV, respectively)

**Conclusions**: We report a subgroup of patients with clinically evident chronic optic neuropathy, marked structural damage of the RNFL and GCL, mild reduction of PERG N95 component and minimal changes of P100 latency and amplitude.

This functional profile may reflect selective or patchy or peripheral fiber involvement of RGC axons, resulting in a structural*–*functional dissociation. Residual redundancy and functional reserve within the visual pathway may be sufficient to preserve VEP responses despite significant RGC loss.

71. **The fairytale of the sight-saving cat and virus**

Gabriella Szatmary^1^, Siena Dhillon^1^

^1^University of North Carolina at Chapel Hill, Chapel Hill, NC, USA

**Introduction**: Mucopolysaccharidosis VI (MPS VI) is an autosomal recessive disorder with severe early ophthalmic involvement. It is caused by mutations in the arylsulfatase B (ARSB) gene, which results in ARSB enzyme deficiency, and buildup of glycosaminoglycans (GAGs), dermatan (DS) and chondroitin-4-sulfate (CS). In the absence of a functional ARSB, GAGs accumulate within lysosomes, leading to cellular dysfunction, connective tissue thickening, and fibrosis.

The ophthalmic manifestations of MPS VI include corneal clouding from DS accumulation in corneal stroma. Glaucoma has been reported frequently in MPS VI, but severe vision loss and optic neuropathy are not always explained by cornea opacification and IOP elevation.

Optic nerve head (ONH) thickening has been attributed to GAG deposition in the sclera, including the ONH lamina cribrosa, in the dura mater, including the optic nerve sheath (ONS). Another pathophysiology that can lead to ONH edema is intracranial hypertension (ICH) in association with impaired cerebrospinal fluid (CSF) dynamics at the narrowed optic canal and/or craniocervical junction due to skeletal dysplasia.

**Case Report**: These mechanisms are illustrated in a 14-year-old male diagnosed with MPS VI at age 13 and treated with weekly enzyme replacement therapy (ERT) with galsulfase. The patient also has skeletal dysplasia, airway narrowing and cardiac valve disease, reflecting advanced connective tissue pathology.

Despite systemic treatment, he demonstrates progressive bilateral though asymmetric ocular involvement with increased corneal thickness. Neuro-ophthalmologic examination revealed severe ONH thickening with corresponding diffusely thickened retinal nerve fiber layer on OCT, consistent with MPS VI-related optic neuropathy. MRI demonstrated severe craniocervical junction stenosis and ONS prominence. These findings and lack of ICH-related symptoms, support a GAG-related “infiltrative” optic neuropathy predominantly driven by connective-tissue remodeling rather than a pressure-mediated process.

**Discussion**: Current ERT improves pulmonary function and urinary GAG levels but has limited penetration into barrier-protected tissues including the cornea and optic nerve and therefore cannot reverse corneal clouding. Substrate reduction therapy with odiparcil can reduce DS/CS in ocular tissues and improve corneal micro-architecture in animal models, demonstrating drug access to the eye; however, in established disease, biochemical and histologic improvement does not translate into reversal of corneal opacity.

In contrast, local ocular gene therapy directly targets the underlying enzymatic deficiency within corneal tissue. At our institution, in a feline model of MPS VI, a single intrastromal adeno-associated virus (AAV)-mediated delivery of human ARSB produced rapid and sustained reversal of corneal clouding within weeks, alongside improved stromal collagen organization, reduced fibrosis, and decreased GAG storage without significant ocular inflammation. These results suggest that localized gene therapy may overcome the limitations of systemic treatments. For patients where progressive corneal opacity and ONH thickening coexist, an AAV ARSB gene addition approach targeting the ONH prior to degeneration may reverse MPSVI-related sight threatening optic neuropathy.

**Disclosures**: Siena Dillon has nothing to disclose. Gabriella Szatmary reports the following: speaker for Amgen, advisory board for Argenx and Catalyst.

73. **Mitochondrial optic neuropathies: The shifting clinical trial landscape**

Grace A. Borchert^1,2,3^, Megan F. Baxter^3,4,5^

^1^Nuffield Laboratory of Ophthalmology, Nuffield Department of Clinical Neurosciences, University of Oxford, Oxford, UK; ^2^Oxford Eye Hospital, Oxford University NHS Foundation Trust, Oxford, UK; ^3^School of Medicine and Dentistry, Griffith University, Gold Coast, Australia; ^4^Wellcome Trust Centre for Human Genetics, University of Oxford, Oxford, UK; ^5^Guy’s and St Thomas’ Hospital, London, UK

**Background and aims**: Leber’s Hereditary Optic Neuropathy (LHON) and Dominant Optic Atrophy (DOA) are the two principal mitochondrial optic neuropathies and represent the leading cause of inherited optic nerve degeneration. Until recently despite both being associated with significant visual morbidity, management has predominantly been supportive to date. In recent years however there has been significant progress and understanding of the disease aetiology and pathogenicity that has driven targeted treatment strategies with promising potential to help individuals affected. This study aimed to firstly provide a systematic update of the latest interventional clinical trials underway for LHON and DOA. Secondly provide insights into current limitations and differences in progress across the disease.

**Methods**: A search was conducted in ClinicalTrials.gov to retrieve the clinical trials in Phase II, III or IV registered for LHON and DOA in January 2026.

**Results**: There was a total of 27 interventional clinical trials for LHON (n=25) or DOA (n=2) in January 2026 in Phase II, III or IV. There was 19 at Phase II, 7 at Phase III, and 1 at Phase IV. In terms of status, 12 were completed, 2 active, 5 recruiting, 1 not yet recruiting, 4 terminated, 1 withdrawn, and 2 unknown study status. Study outcomes and results across completed studies were evaluated. The most common primary outcomes were ocular treatment emergent adverse events, best corrected visual acuity, clinically relevant recovery of visual acuity from baseline, and change from baseline in Newcastle Paediatric Mitochondrial Disease scale.

Across LHON studies, multiple therapies demonstrated improvements in visual function, with the most consistent and sustained benefits observed in gene-based and mitochondrial-targeted approaches. In contrast, DOA has only a small number of early-stage interventional studies, with limited evidence of sustained efficacy to date. This is likely secondary to a greater genetic and mechanistic heterogeneity, technical challenges in therapeutic targeting, slower disease progression, and less well-established natural history data and outcome measures for DOA.

Interpretation across trials is however complicated by heterogeneity in study design, outcome measures, and follow-up duration, as well as spontaneous visual recovery in LHON.

**Conclusion**: Mitochondrial optic neuropathies have been previously focused on supportive care. There has however been a significant shift in the clinical trial landscape for optic neuropathy with emerging targeted therapeutics. An understanding of the current clinical trials for mitochondrial optic neuropathies will be helpful in guiding future trial work.

74. **OCT RNFL sector-specific analysis for papilloedema detection: PHOMS coexistence in 41% of cases does not impair diagnostic performance**

Guillaume Mignot^1^, Amna Irfan^1^, Ramith Ginige^1^, Shivona Chetty^2^

^1^Aberdeen Royal Infirmary, NHS Grampian, Scotland; ^2^Ophthalmology Department, Sligo University Hospital, Sligo, Ireland

**Background**: Peripapillary hyperreflective ovoid mass-like structures (PHOMS) and optic disc drusen are known to occur simultaneously alongside true papilloedema. However, their impact on the accuracy of OCT-RNFL (Optical Coherence Tomography Retinal Nerve Fibre Layer) thickness measurements in diagnosing papilloedema has not been established. This is particularly important given the fact that clinicians may interpret the presence of PHOMS as an indication that their patient may have pseudo, rather than true papilloedema. As community optometrists become increasingly equipped with OCT facilities, establishing how to safely and efficiently process electronic referrals will be crucial. This is particularly important in sparsely populated rural regions where patient transport is an issue.

**Methods**: Retrospective analysis of 78 consecutive patients with suspected disc swelling seen at Aberdeen Royal Infirmary (August 2023-January 2024) serving Scotland’s second largest health board. Raised Intra-Cranial Pressure (ICP) was confirmed by lumbar puncture (>25 cmH2O). OCT RNFL measured across six sectors (Heidelberg Spectralis). Enhanced depth imaging identified PHOMS/drusen. Direct comparison between: (1) normal discs; (2) PHOMS without raised ICP; (3) PHOMS with raised ICP; (4) raised ICP alone. Virtual triage validated through 16-month follow-up of 40/128 rejected e-referrals.

**Results**: Twenty-two patients (28.2%) had raised ICP (20 Idiopathic Intracranial Hypertension, 1 hydrocephalus, 1 tumour). Mean age 28.7 years, 73.8% female. OCT RNFL demonstrated excellent discrimination (AUC 0.858, 95% CI 0.731-0.963), with raised ICP showing marked elevation (203.9±98.1 vs 120.3±25.5 μm, p<0.001, Cohen’s d=1.48). Temporal-inferior achieved perfect sensitivity (100%, AUC 0.889) at 165 μm; nasal-inferior excellent specificity (95.7%) at 162.5 μm. Amongst 17 raised ICP cases with enhanced imaging, PHOMS/drusen were present in 7 (41.2%, 95% CI 21.6%-64.0%). Patients with both PHOMS and raised ICP maintained significantly elevated RNFL versus PHOMS alone (150.7±39.5 vs 124.5±28.1 μm, Cohen’s d=0.807, p=0.017), validating OCT despite coexistence. PHOMS attenuated elevation versus raised ICP alone (150.7 vs 235.0 μm, p=0.017) but did not reverse the relationship. Virtual triage achieved zero missed cases over 16 months (0/40 rejected referrals). However, 18.2% of raised ICP cases showed neither clinical nor OCT abnormalities (RNFL 113.2 vs 120.3 μm, p=0.71), representing early disease.

**Conclusion**: Identification of PHOMS or optic disc drusen on EDI-OCT must not provide false reassurance in symptomatic patients. Coexistence with raised intracranial pressure occurs in 41% of papilloedema cases. Clinicians should maintain high suspicion and proceed with lumbar puncture in patients with typical IIH demographics and symptoms, regardless of drusen/PHOMS presence as a substantial minority of cases with confirmed raised ICP present with no detectable increase in RNFL thickness. Virtual triage incorporating comprehensive clinical assessment is safe and effective, achieving zero missed cases over 16 months. OCT-RNFL sector specific analysis, especially looking at the infero-nasal sector, may help improve the safety and accuracy of telemedicine practices in neuro-ophthalmology across Europe.

81. **Virus antibody responses in patients with ON as part of MOGAD versus RRMS**

Jette Lautrup Frederiksen^1^, Nicole Hartwig Trier^2^, Rasa Petraitytė-Burneikienė ^3^, Danguolė Žiogienė ^3^, Rimantas Slibinskas^3^, Evaldas Ciplys^3,4^, Gunnar Houen^2^

^1^Clinic of Optic Neuritis, Rigshospitalet Copenhagen and University of Copenhagen, Denmark; ^2^Department of Neurology, Rigshospitalet, Copenhagen, Denmark; ^3^Institute of Biotechnology, Vilnius University, Vilnius, Lithuania; ^4^Department of Biochemistry and Molecular Biology, University of Southern Denmark, Odense M, Denmark

**Background:** Myelin oligodendrocyte glycoprotein antibody-associated disease (MOGAD) is an antibody mediated inflammatory disorder of the central nervous system, which is characterized by demyelination targeting the optic nerve, brain and spinal cord.

The etiology of MOGAD still remains to be determined. Here we performed a retrospective study comparing virus antibody levels in sera of ON patients with MOGAD patients to ON patients with RRMS. Additionally, cerebrospinal fluid analyses to Epstein-Barr virus (EBV) antigens were determined in MOGAD patients and compared to RRMS patients, as EBV plays a central role in the development of MS.

**Methods:** We enrolled 19 patients with MOGAD and 30 patients with RRMS referred to the Department of Neurology at Rigshospitalet Glostrup. Moreover, 15 healthy controls (HCs) were included. Patients and HCs were matched according to age and gender when possible. Virus antibody levels to EBV, HHV6, JCV and CMV were measured by enzyme-linked immunosorbent assay.

**Results:** No differences in virus antibody levels to HHV6, CMV and JCV were observed between MOGAD patients and HCs. Similarly, no significant differences in antibody levels to HHV6, CMV and JCV were found for MOGAD patients and RRMS patients. However, RRMS patients presented with significantly increased EBV EBNA1 IgG levels in when compared to MOGAD patients and HCs.

**Conclusions:** Our findings suggest that development of MOGAD is neither associated with HHV6, CMV nor JCV. Furthermore, EBV serology is different in MOGAD patients compared to RRMS, suggesting that the etiology of MOGAD is different from RRMS.

82. **Idiopathic unilateral optic disc edema in a young man: A diagnostic challenge**

Joana da Silva Rocha^1^, Ana Rita Viana^1^, Alberto Lemos^1^

^1^Ophthalmology Department, Hospital Pedro Hispano, Matosinhos, Portugal

**Introduction**: Unilateral optic disc edema with preserved visual acuity is uncommon and represents a diagnostic challenge in neuro-ophthalmology. Differentiating pseudopapilledema from true optic neuropathy, as well as distinguishing between ischemic, infectious, inflammatory, and pressure-related causes, is essential to guide management. Multimodal imaging and longitudinal structural assessment play a key role in investigation.

**Case Report**: A 32-year-old male presented with blurred vision of the right eye (RE) for the previous two days. He denied headache, ocular pain, fever, rash, or arthralgia. Past medical history included obstructive sleep apnea (OSA), dyslipidaemia, ex-smoker, and depression. He had recently adopted two cats. Visual acuity was 10/10 bilaterally. A relative afferent pupillary defect with marked red desaturation was presented in the RE. Fundoscopy revealed hyperaemic optic disc edema in the RE without hemorrhages or vitritis; however scarce unspecific hypo- and hyperpigmented peripheral lesions were visualized. The left eye (LE) was normal. Optical coherence tomography (OCT) demonstrated optic disc edema in the RE and superior and inferior retinal nerve fiber layer (RNFL) thinning in the LE.

Fundus autofluorescence showed no optic disc hyperautofluorescence suggestive of optic disc drusen. Fluorescein angiography demonstrated optic disc leakage in the RE without vasculitis or other retinal abnormalities. Laboratory work-up was unremarkable except for isolated Borrelia IgM positivity with subsequent negative confirmatory testing. Empirical treatment with doxycycline and rifampicin was initiated, followed by oral corticosteroids. Brain and orbital magnetic resonance imaging (MRI) showed nonspecific periventricular and subcortical white matter hyperintensities without signs of raised intracranial pressure or venous sinus thrombosis, together with a normal MRI of the neuroaxis. Cerebrospinal fluid (CSF) analysis revealed borderline opening pressure, no pleocytosis, mildly elevated protein, and one unmatched oligoclonal band. Serum anti–myelin oligodendrocyte glycoprotein and anti–aquaporin-4 antibodies were negative.

Follow-up revealed resolution of optic disc edema with significant RNFL thinning and early ganglion cell complex involvement on OCT. Automated perimetry (Humphrey 30-2) demonstrated a dense inferior and milder superior arcuate defects in the RE, with preserved central visual field (visual field index (VFI) 41%, mean deviation (MD) −24.07 dB) and a milder inferior peripheral defect in the LE (VFI 80%, MD −11.48 dB).

**Discussion**: At present, non-arteritic anterior ischemic optic neuropathy (NAION) was considered the most likely diagnosis given the acute presentation, preserved initial visual acuity, rapid post-edema RNFL thinning, and the presence of vascular risk factors including OSA and dyslipidaemia. OCT and visual field findings were compatible with this diagnosis. However, inflammatory demyelinating optic neuritis remains an important differential diagnosis considering the patient’s young age and the presence of optic disc atrophy in the fellow eye, although currently there aren’t sufficient clinical, imaging, or laboratory findings supporting this diagnosis. Infectious neuroretinitis was also considered despite atypical features and negative microbiological studies.

**Disclosures**: The authors report no conflicts of interest.

84. **Inner retinal layer thinning, age and genetic variant class influence visual function in OPA1 autosomal dominant optic atrophy**

Johannes Schrittwieser,^1^ Andreas Reitner,^1^ Karl Kircher,^1^ Martin Bertich,^1^ Christoph Stapf,^1^ Stephanie Lilja,^2^ Amina Paquay,^2^ Gregor S. Reiter,^1^ Reginald E. Bittner,^2^ Wolfgang M. Schmidt,^2^ Berthold Pemp^1^

^1^Department of Ophthalmology and Optometry, Medical University of Vienna, Vienna, Austria; ^2^Neuromuscular Research Department, Center for Anatomy and Cell Biology, Medical University of Vienna, Vienna, Austria

**Purpose**: To evaluate the impact of inner retinal layer thinning on visual function measured by visual acuity (VA) and visual field (VF) testing in patients with *OPA1*-related autosomal dominant optic atrophy (ADOA), and to evaluate the influence of age and genetic variant class on visual function and retinal structure.

**Methods**: In this retrospective, cross-sectional study we analyzed clinical data of ADOA patients genetically confirmed with a disease-causing heterozygous variant in the *OPA1* gene. In addition to best corrected VA, we collected global mean deviation (MD) and the MD in the papillomacular bundle (PMB) subfield from the results of 30-2 automated threshold perimetry. Peripapillary retinal nerve fiber layer (pRNFL) thickness and macular ganglion cell layer (mGCL) thickness were evaluated using spectral-domain optical coherence tomography. Furthermore, we assessed differences between patients with haploinsufficiency-causing and missense variants. Statistical analysis was performed using mixed-effects models correcting for age and sex.

**Results**: Clinical data of 108 patients (66 males, 42 females; mean age 33 ± 17 years) harboring one of 54 different variants in *OPA1* were analyzed. Mean VA was 0.55 ± 0.39 logMAR, median MD was -3.62 (-6.26; -1.94) dB, and median PMB-MD was -4.67 (-11.00; -2.67) dB. Mean mGCL thickness measured 21.69 ± 3.68 µm, and pRNFL thickness was 64.31 ± 12.49 µm. Age negatively impacted PMB-MD (*P*=0.041) and mGCL thickness (*P*=0.028). In multivariable analysis, VA was significantly impacted by mGCL reduction (-0.03 [-0.05; -0.01] logMAR/µm; *P*<0.001) and by pRNFL reduction (-0.01 [-0.01; -0.00] logMAR/µm; *P*=0.008). Furthermore, pRNFL reduction was significantly associated with global MD loss (0.21 [0.11; 0.31] dB/µm; *P* <0.001), and mGCL thinning was associated with a reduction in PMB-MD (1.02 [0.56; 1.47] dB/µm; *P*<0.001). Patients harboring missense variants showed worse VA (0.83 vs. 0.49 logMAR), MD (-11.48 vs. -3.01 dB) and PMB-MD (-16.25 vs. -4.17 dB) than patients with haploinsufficiency variants (all *P* <0.001), and also exhibited more pronounced thinning in the mGCL (20.12 vs. 21.97 µm; *P*=0.015) and the pRNFL (52.41 vs. 66.41 µm; *P*<0.001).

**Conclusion**: In *OPA1*-related ADOA, inner retinal layer thinning is significantly associated with changes in VA and VF defects, especially in the PMB subfield. mGCL loss and central scotoma show worse outcomes with increasing age, and patients harboring missense variants exhibit more pronounced functional and structural impairment.

**Disclosures**: None.

89. **Late-onset Leber hereditary opyic neuropathy: A case series of 16 patients diagnosed above 65 years of age**

Putcuijps Kato^1^, Lushchyk Tanya^2^, Cassiman Catherine^3^, Van Everdingen Judith^2^

^1^University Hospitals of Leuven, Belgium; ^2^Het Oogziekenhuis Rotterdam, The Netherlands; ^3^Department of Ophthalmology, UZ Leuven, Leuven, Belgium

**Background**: Leber hereditary optic neuropathy (LHON) is a mitochondrial optic neuropathy that typically presents in young adult males. New-onset LHON at advanced age is considered rare and remains insufficiently characterised and poorly recognised. Improved understanding of late-onset disease is essential for accurate diagnosis and management in elderly patients presenting with unexplained optic neuropathy.

**Case Reports**: We performed a retrospective case series of patients aged over 65 years who were diagnosed with new-onset LHON between 2004 and 2025 at two tertiary referral centers (University Hospitals Leuven, Belgium, and Rotterdam Eye Hospital, The Netherlands). Demographic characteristics, genetic findings, environmental and systemic risk factors, as well as visual outcomes were analysed. Best-corrected visual acuity (BCVA) was assessed at baseline, at nadir, and at least one year after disease onset. Treatment response to Idebenone was evaluated using predefined criteria for clinically relevant recovery (CRR).

Sixteen patients were included, all of whom were male, with a mean age at onset of 72.8 years. The most frequently identified mutation was MT-ND4 (m.11778G>A), present in eleven patients, followed by MT-ND6 (m.14484T>C) in three patients and MT-ND1 (m.4135T>C) in one patient. Seven patients reported a family history of LHON, although none had a symptomatic mother. At initial presentation, visual loss was already severe in most cases, with half of the cohort demonstrating best-corrected visual acuity below 0.1 (Snellen) in both eyes. Twelve patients received Idebenone therapy, of whom six demonstrated a clinically relevant recovery. However, final visual acuity remained poor in the majority of eyes. Environmental and systemic risk factors were common, including smoking history in 56% of patients, high-risk alcohol consumption in 50%, diabetes mellitus in 31%, and open-angle glaucoma in 19%.

**Discussion**: LHON possibly manifests itself for the first time at all ages in male and female patients, carrying common mitochondrial DNA mutations. This case series highlights that late-onset LHON represents a clinically relevant but underrecognised phenotype with severe visual impairment and limited therapeutic response to Idebenone treatment. The delayed disease expression likely reflects a multifactorial process in which genetic susceptibility interacts with cumulative environmental exposures, systemic comorbidities, and age-related mitochondrial dysfunction. Progressive mitochondrial decline reduced neuronal reserve, and diminished compensatory mechanisms may lower the threshold for retinal ganglion cell degeneration later in life, allowing pathogenic mtDNA variants to become clinically manifest only at advanced age. Awareness of this late-onset phenotype is essential to improve diagnostic accuracy, avoid misclassification as other optic neuropathies, and provide appropriate genetic counseling and management for patients and their families.

**Disclosures**: The authors report no conflicts of interest related to this work.

92. **Reduction in retinal ganglion cell layer and macular nerve fiber layer loss with privosegtor in acute optic neuritis: Post-hoc analysis of OCT scans from the phase 2 ACUITY study**

Leila S. Eppenberger^1^, Martin S. Zinkernagel^1^, Céline Louapre^2^, Sophie Bonnin^3^, Louise-Laure Mariani^2,4^, Mikael Cohen^5^, Caroline Froment Tilikete^6^, Mark Kupersmith^7^, Leonard A. Levin^8^, Sabri Markabi^9^, Caroline Papeix^10,11^, Valérie Touitou^12^, Rossella Medori^13^, Arshad M. Khanani^14^, Pablo Villoslada^15^, Sebastian Wolf^1^

^1^Department of Ophthalmology, Inselspital, University of Bern, Bern, Switzerland; ^2^Department of Neurology, APHP Pitié-Salpêtrière and Sorbonne University, Paris Brain Institute, CIC Neurosciences, Paris, France; ^3^Department of Ophthalmology, Foundation A. de Rothschild Hospital, Paris, France; ^4^Department of Medical Pharmacology, APHP Pitié-Salpêtrière and Sorbonne University, Paris, France; ^5^CRCSEP Neurologie Pasteur 2, CHU de Nice, Université Côte d’Azur, UMR2CA (URRIS), Nice, France; ^6^Neuro-Ophthalmology Department, Hospices Civils de Lyon, Lyon, France; ^7^New York Eye and Ear Infirmary, New York, NY, USA; ^8^Department of Ophthalmology and Visual Sciences, McGill University, Montréal, Québec, Canada; ^9^Health R&D LLC, Miami, FL, USA; ^10^Department of Neurology, Foundation A. de Rothschild Hospital, Paris, France; ^11^Paris-Cité University, Paris, France; ^12^Department of Ophthalmology, APHP Pitié-Salpêtrière Hospital and Sorbonne University, Paris, France; ^13^Prexton Therapeutics SA, Geneva, Switzerland; ^14^ Sierra Eye Associates, Reno, NV, USA and University of Nevada, Reno School of Medicine, Reno, NV, USA^15^Department of Neurology, Hospital del Mar and Pompeu Fabra University, Barcelona, Spain

**Introduction**: The ACUITY study (NCT04762017) demonstrated that privosegtor significantly improves vision and reduces ganglion cell-inner plexiform layer (GCIPL) damage in patients with acute optic neuritis (AON). In ACUITY, GCIPL and macular retinal nerve fiber layer (mRNFL) thickness were assessed. In this post-hoc analysis, we evaluated if mRNFL and GCIPL measurements obtained from additional macular regions yield comparable results.

**Methods**: Thirty-three participants were randomized and treated in the ACUITY study. We included the privosegtor 3 mg/kg/day group (n=15) and the placebo group (n=14) in this analysis. GCIPL and mRNFL thickness were collected using spectral domain optical coherence tomography (SD-OCT; with Spectralis [Heidelberg Engineering, Heidelberg, Germany]). We evaluated macula volume scans within the 6mm ETDRS grid, collected at baseline, day 5, and months 1, 3, and 6. We compared the mean GCIPL and mRNFL thickness for three regions: the 6mm circle excluding the 1mm central area (inner and outer ring), the 6mm circle excluding the inner 3mm circle (outer ring), and the 3mm circle excluding the 1mm central area (inner ring). Statistical analyses were performed using mixed models for repeated measures to estimate least-squares (LS) mean change from baseline with standard error (SE) and 90% confidence intervals (CI).

**Results**: The privosegtor group eyes had significantly reduced GCIPL thinning compared with placebo group eyes at months 3 and 6 across all ETDRS regions analyzed. At 3 months, the inner ring (3mm) measurement (–12.0 ±3.2 µm vs –20.9 ±3.5 µm; p=0.07) showed the greatest treatment effect. The outer ring showed a smaller but statistically significant difference (–6.0 ±1.6 µm vs –11.5 ±1.8 µm; p=0.03). Results at month 6 were similar. Privosegtor eyes showed a trend of reduced mRNFL for all regions. At 3 months, the mean change in the inner ring was -1.88 ±1.79 µm in the privosegtor group eyes versus -2.96 ±2.07 µm in the placebo group. The outer ring, mean RNFL thinning was -4.64 ±4.11 µm versus -8.07 ±6.02 µm for privosegtor and placebo groups, respectively.

**Discussion**: In patients with AON, privosegtor significantly reduced GCIPL thinning within the 6-mm central macular region at both 3 and 6 months and showed a trend toward reduced mRNFL thinning up to month 6. Especially for RNFL optic disc swelling may be a confounding at baseline. The strongest treatment effect was observed in the region closest to the fovea, likely reflecting more robust OCT measurement due to the higher density of ganglion cells in this area. However, data collected across a larger macular region remained consistent between mRNFL and GCIPL. Together with the previously demonstrated improvements in visual function in ACUITY, these findings of improved neuronal structural preservation suggest privosegtor represents a novel first-in-class neuroprotective treatment for acute optic neuritis.

**Disclosures:** Leila S. Eppenberger reports no conflicts of interest. Martin S. Zinkernagel has been a consultant for Bayer, Roche, Alcon, and Oculis, and has received grants from Bayer. Céline Louapre has received speaker or consultancy fees from Biogen, Sanofi, Novartis, and Merck, not related to the present study, and is consultant for Oculis. Sophie Bonnin is a consultant for AbbVie, AstraZeneca, Bayer, Luneau, Oculis SA, Optic 2000 and Roche. Louise-Laure Mariani is a consultant for Oculis SA and received research support grants from the CNES, the French Parkinson Association, Fondation of France, Paris Brain Institute, and the Axa Foundation; received honorarium for scientific advice or lectures for Elivie, Sanofi-Genzyme, Accure Therapeutics, Owkin, Emeis, and the Paris Brain Institute; and received travel funding from Merz, outside of the submitted work. Mikael Cohen has received personal compensation for consulting, serving on a scientific advisory board, speaking, or other activities with Biogen, Novartis, Sanofi, Roche, Alexion, Horizon Therapeutics and Ad Scientiam. Caroline Froment Tilikete reports no conflicts of interest. Mark Kupersmith is a consultant to Oculis SA. Leonard A. Levin is a consultant to Dompe, Life Biosciences, Oculis, and Perceive. Sabri Markabi is a consultant to Oculis SA. Caroline Papeix has been an advisory board member or conference speaker for Roche, Biogen, Novartis, Amgen, and Alexion. Valérie Touitou reports no conflicts of interest. Rossella Medori reports no conflicts of interest. Arshad M. Khanani is a consultant to Oculis SA. Pablo Villoslada holds several patents covering privosegtor composition of matter and its use for the treatment of CNS and ophthalmologic diseases. He holds stocks or stock options and has received consultancy fees from Oculis SA, Bionure Therapeutics, and Accure Therapeutics as part of the development of this program. Sebastian Wolf is consultant for Celltrion Healthcare, Novartis Pharma, Oculis SA, and Priothera SAS.

93. **Long-term OCT and visual outcomes after hypofractionated radiosurgery for optic nerve sheath meningioma: 48-month monocentric prospective study**

Ludovica Gargiulo^1^, Marcello Marchetti^3^, Gabriella Cammarata^1^, Paola Ciasca^1^, Alberto Raggi^2^, Giorgia Camarda^2^, Sara Morlino^3^, Laura Fariselli^3^, Stefania Bianchi Marzoli^1^

^1^Neuro-Ophthalmology Center and Electrophysiology Laboratory, Department of Ophthalmology, IRRCS Istituto Auxologico Italiano, Milan, Italy; ^2^SC Neurologia Salute Pubblica e Disabilità, Fondazione IRCCS Istituto Neurologico Carlo Besta, Milan, Italy; ^3^Radiotherapy Unit Department of Neurosurgery, Fondazione IRCCS Istituto Neurologico Carlo Besta, Milan, Italy

**Introduction**: Optic nerve sheath meningioma (ONSM) is a rare, benign tumor that can cause progressive visual loss through chronic compression of the optic nerve. Hypofractionated stereotactic radiosurgery (HypoSRS) has been shown to provide durable tumor control while respecting dose constraints of the optic pathway. We evaluated long-term visual functional and structural outcomes after HypoSRS for unilateral ONSM.

**Methods**: In this exploratory, prospective, single-arm study, we included 50 patients diagnosed with unilateral chronic optic neuropathy due to ONSM who underwent CyberKnife HypoSRS (25 Gy in 5 fractions). Neuro-ophthalmological assessments were performed at baseline, 24 months, and 48 months. Parameters considered for statistical analysis included best-corrected visual acuity (BCVA), mean deviation (MD, dB) assessed by Humphrey automated perimetry, and macular ganglion cell complex (GCC, µm) and retinal nerve fiber layer (RNFL, µm) thickness measured by spectral-domain OCT (RTVue). Patients who had undergone OCT analysis using a different instrument were excluded from the analysis.

**Results**: Thirty-nine patients were included (82% female and 18% male; mean age 49.8 ± 11.7 years). In ONSM-affected eyes at baseline, mean BCVA was 4.6, MD was −19.3 dB, and GCC thickness was 75.7 µm; RNFL edema was detected in eight eyes. Statistically significant improvements in BCVA and MD were observed between baseline and 24 months (mean 5.2, p = 0.010; −13.8 dB, p < 0.001) and between 24 and 48 months (mean 5.8, p = 0.010; −13.5 dB, p < 0.001). A mild but significant decrease in GCC thickness was observed between baseline and 24 months (mean 72.3 µm, p = 0.007) and between 24 and 48 months (mean 70.7 µm, p = 0.007). No statistically significant changes were observed in the contralateral healthy eyes for the same parameters.

**Conclusion**: In this prospective study we demonstrated improvement in visual function and long-term stabilization after CyberKnife HypoSRS in patients with severe optic neuropathy related to ONSM. Mild GCC thinning may reflect progression of pre-treatment retrograde degeneration and, in eyes with baseline RNFL edema, may also be related to resolution of optic nerve swelling.

95. **Migraine-related retinal arterial occlusion: Two cases supporting a vasospastic mechanism**

Laura Sánchez Vela^1^, Laura Vigués^1^, Ruth Martín^1^

^1^Vall d’Hebron University Hospital, Barcelona, Spain

**Introduction**: Migraine is a common neurovascular disorder that has been increasingly associated with ocular vascular events, particularly in patients without traditional cardiovascular risk factors. Retinal arterial occlusions related to migraine are rare and are thought to be mediated by transient vasospasm, endothelial dysfunction, or dysregulation of retinal blood flow. We report two cases of retinal arterial occlusion temporally associated with migraine attacks, highlighting the importance of recognizing migraine as a potential risk factor for retinal ischemia.

**Case Report**: The first case involved a 28-year-old woman with a known history of migraine who presented to the emergency department with the acute onset of a paracentral scotoma in her left eye following a migraine attack. Best-corrected visual acuity was preserved. Automated visual field testing revealed a centrocecal scotoma in the left eye. Funduscopic examination was unremarkable. Spectral-domain optical coherence tomography (OCT) demonstrated focal atrophy of the inferior inner retina, consistent with a branch retinal arterial occlusion. Systemic evaluation revealed no cardiovascular risk factors, embolic sources, or hypercoagulable conditions. Given the temporal relationship with migraine and the absence of alternative etiologies, the occlusion was attributed to retinal arterial vasospasm associated with migraine.

The second case involved a 52-year-old woman with a long-standing history of migraine who developed a persistent scotoma in her right eye following a prolonged migraine episode. Visual field testing revealed a superior hemifield defect in the affected eye. OCT imaging showed atrophy of the inferior retinal layers, consistent with retinal arterial ischemia. No embolic source or systemic vascular pathology was identified on further evaluation. The clinical and imaging findings supported a diagnosis of retinal arterial occlusion secondary to migraine-related vasospasm.

**Discussion**: These cases illustrate retinal arterial occlusion as a potential ocular complication of migraine, likely mediated by transient retinal vasospasm. Both patients lacked conventional vascular risk factors, and imaging findings supported localized retinal ischemia corresponding to visual field defects. Migraine-associated retinal ischemic events may be underrecognized, particularly when funduscopic findings are subtle or absent in the acute phase. Optical coherence tomography and visual field testing play a critical role in diagnosis. Clinicians should consider migraine-related vasospasm in young or middle-aged patients presenting with unexplained retinal arterial occlusion, as early recognition may prompt appropriate systemic evaluation and counseling regarding migraine management and vascular risk.

**Disclosures**: The authors report no financial interests, conflicts of interest, or proprietary relationships related to this work.

100. **Recessive variants in mitochondrial Complex I nuclear subunits are an underrated cause of optic atrophy**

Claudio Fiorini^1,#^, Neringa Jurkute^2,3,4,#^, Alessandra Torraco^5#^, Chiara La Morgia^1,6^, Daniele Ghezzi^7,8^, Gaia Tioli^9^, Laura Rigobello^9^, Danara Ormanbekova^1^, Alessandro Berghella^6^, Alberto Pietro Pasti^1^, Flavia Palombo^1^, Piero Barboni^10^, Maria Lucia Cascavilla^10^, Federico Sadun^11^, Annamaria De Negri^12^, Enrico Bertini^13^, Olimpia Musumeci^14^, Anna Ardissone^15^, Teresa Rizza^5^, Giancarlo Iarossi^16^, Gabriella Silvestri^17,18^, Salvatore Rossi^17^, Anastasia Altobelli^5^, Antony T. Moore^2,3^, Thomas Cullup^19^, Andrew R Webster^2,3^, Indran Davagnanam^2^°, Michel Michaelides^2,3^, Samantha Malka^2^, Hana Ptackova^21^, Hana Stufkova^21^, Marketa Tesarova^21^, Petra Liskova^22^, Leopold Zeng^23^, Thomas Klopstock^23,24,25^, Robert Kopajtich^26,27^, Christiane Neuhofer^26,27, 28^, Holger Prokisch^26,27,29^, Costanza Lamperti^7^, Alfredo A. Sadun^3^° ^,31^, Patrick Yu-Wai-Man^2,3,32,33^, Valerio Carelli^1,6^, Francesco Musiani^9^, Luisa Iommarini^9*^, Rosalba Carrozzo^5*^, Gavin Arno^2,3,34*^, Leonardo Caporali^1*§^

^1^IRCCS Istituto delle Scienze Neurologiche di Bologna, Programma di Neurogenetica, Bologna, Italy; ^2^Moorfields Eye Hospital NHS Foundation Trust, London, UK; ^3^Institute of Ophthalmology, University College London, London, UK; ^4^The National Hospital for Neurology and Neurosurgery, Queen Square, University College London Hospitals NHS Foundation Trust, London, UK; ^5^Laboratory of Medical Genetics, Translational Cytogenomics Research Unit, Bambino Gesù Children’s Hospital IRCCS, Rome, Italy; ^6^Department of Biomedical and Neuromotor Sciences (DIBINEM), University of Bologna, Bologna, Italy; ^7^Fondazione IRCCS Istituto Neurologico Carlo Besta, Unit of Medical Genetics and Neurogenetics, Milan, Italy; ^8^Department of Pathophysiology and Transplantation, University of Milan, Milan, Italy; ^9^Department of Pharmacy and Biotechnology (FABIT), University of Bologna, Bologna, Italy; ^10^IRCCS Ospedale San Raffaele, Milan, Italy; ^11^Ospedale Oftalmico Roma, Rome, Italy; ^12^Azienda Ospedaliera San Camillo-Forlanini, Rome, Italy; ^13^Research Unit of Neuromuscular and Neurodegenerative Disorders, Bambino Gesù Children’s Hospital IRCCS, Rome, Italy; ^14^Department of Experimental and Clinical Medicine, University of Messina, Messina, Italy; ^15^Fondazione IRCCS Istituto Neurologico Carlo Besta, Child Neurology Unit, Milan, Italy; ^16^Ophthalmology Unit, Bambino Gesù Children’s Hospital IRCCS, Rome, Italy; ^17^Department of Neuroscience, Università Cattolica del Sacro Cuore-Sede di Roma, Rome, Italy; ^18^Neurology Unit, Fondazione Policlinico Universitario Agostino Gemelli IRCCS, Largo A. Gemelli, Rome, Italy; ^19^North Thames Genomic Laboratory Hub, Great Ormond Street Hospital, London, UK; ^20^Department of Brain Repair and Rehabilitation, University College London Institute of Neurology, Faculty of Brain Sciences, UCL, London, UK; ^21^Department of Paediatrics and Inherited Metabolic Disorders, First Faculty of Medicine, Charles University and General University Hospital in Prague, Prague, Czech Republic; ^22^Department of Ophthalmology, First Faculty of Medicine, Charles University and General University Hospital in Prague, Prague, Czech Republic; ^23^Friedrich-Baur-Institute, Department of Neurology, University Hospital, Ludwig-Maximilians University, Munich, Germany; ^24^German Center for Neurodegenerative Diseases, Munich, Germany; ^25^Munich Cluster for Systems Neurology (SyNergy), Munich, Germany; ^26^Institute of Human Genetics, Computational Health Center, Helmholtz Zentrum München, Neuherberg, Germany; ^27^Institute of Human Genetics, School of Medicine, Technical University of Munich, Munich, Germany; ^28^Department of Clinical Human Genetics, University Medical Center Regensburg, Regensburg, Germany; ^29^German Center for Child and Adolescent Health (DZKJ), partner site Munich, Munich, Germany; ^30^Doheny Eye Institute, Pasadena, CA, USA; ^31^Department of Ophthalmology, David Geffen School of Medicine, UCLA, Los Angeles, CA, USA; ^32^John van Geest Centre for Brain Repair and MRC Mitochondrial Biology Unit, Department of Clinical Neurosciences, University of Cambridge, Cambridge, UK; ^33^Cambridge Eye Unit, Addenbrooke’s Hospital, Cambridge University Hospitals NHS Foundation Trust, Cambridge, UK; ^34^Greenwood Genetic Center, Greenwood, SC, USA

Our understanding of the genetic landscape of inherited optic neuropathies has grown significantly over the past decades, and it is now known to involve many genes found in both the nuclear and mitochondrial genomes, exhibiting all possible inheritance patterns. Furthermore, pathogenic variants in nuclear genes of mitochondrial respiratory Complex I (CI) subunits have been identified in some cases of ION, in addition to the more common severe presentation of CI deficiencies, usually with early onset.

We conducted NGS screening of CI genes to identify potential causative variants in patients with optic atrophy, also performing comprehensive clinical assessments, including neuroimaging studies (MRI) and neurological evaluations. Detailed molecular structure modeling was performed to better evaluate the damaging effects of both novel and previously reported variants in the relevant CI subunits.

We identified and characterized candidate causative variants in 31 patients from 23 unrelated families, with biallelic or hemizygous variants in 11 different nuclear CI-related genes encoding polypeptides involved in the structure of CI, including 3 core subunits (*NDUFS7, NDUFV1, NDUFV2*), 4 accessory subunits (*NDUFA1, NDUFA10, NDUFA12, NDUFB11*), and 4 assembly factors (*NDUFAF2, NDUFAF3, NDUFAF4, NDUFAF8*). Notably, defects in core CI subunits in this cohort lead to isolated optic atrophy, while defects in accessory CI subunits and assembly factors resulted in a spectrum of phenotypes, from isolated to syndromic optic atrophy. For 12 cases, the subacute onset of vision loss enabled us to associate or confirm novel genes (*NDUFS7, NDUFV1, NDUFAF2, NDUFAF4, NDUFAF8*) with the autosomal recessive Leber Hereditary Optic Neuropathy (arLHON) phenotype. Moreover, in the NDUFS7 subunit a partial spatial segregation was noted for missense variants causing either Leigh syndrome or isolated optic atrophy, hinting at possible disease-specific molecular defects.

Our case series broadens the genetic spectrum of inherited optic neuropathies, emphasizing the crucial role of nuclear CI genes in its pathogenesis. The arLHON phenotype emerges as linked to numerous nuclear CI genes for which an insidious onset of optic atrophy is also reported, and in some cases the same variant may underlie both phenotypes. Overall, we highlight the possibly so far underestimated prevalence of CI nuclear subunits in the molecular diagnosis of ION, prompting to include all CI-related genes in the standard diagnostic screening.

101. **Comparative OCT neuroretinal profiles in acute LHON and atypical optic neuritis**

Leonie F Keidel^1,2^, Alexander Kufner^1^, Jonathan Gernert^3^, Jana Mattar^1^, Patrick Yu-Wai Man^2,4,5,6^, Claudia Priglinger^1^, Bettina von Livonius^1^, Thomas Klopstock^7^, Linus Keidel^7^, Siegfried G Priglinger^1^, Joachim Havla^3^*, Neringa Jurkute* *Shared first author
^2,5^

^1^Department of Ophthalmology, LMU University Hospital, LMU Munich, Munich, Germany; ^2^Department of Neuroophthalmology, Moorfields Eye Hospital, NHS Foundation Trust, London, UK; ^3^Department of Neuroimmunology, LMU University Hospital, LMU Munich, Munich, Germany; ^4^John von Geest Centre for Brain Repair and MRC Mitochondrial Biology Unit, Department of Clinical Neurosciences, University of Cambridge, Cambridge, UK; ^5^Institute of Ophthalmology, University College London, London, UK; ^6^Cambridge Eye Unit, Addenbrooke’s Hospital, Cambridge, University Hospitals, Cambridge, UK; ^7^LMU Munich Friedrich Baur Institute at the Department of Neurology, LMU University Hospital, LMU Munich, Munich, Germany; ^8^Department of Neurology, LMU University Hospital, LMU Munich, Munich, Germany; ^9^Department of Neuroophthalmology, The National Hospital for Neurology and Neurosurgery, London, UK

**Purpose**: Leber hereditary optic neuropathy (LHON) may be misdiagnosed as inflammatory optic neuritis during its early clinical course due to overlapping acute presentations. This study aimed to compare OCT (optical coherence tomography)-based neuroretinal parameters, such as peripapillary retinal nerve fiber layer (pRNFL) and macular ganglion cell/inner plexiform layer (mGCIPL) thickness in the acute stages of optic neuropathy in patients with LHON to patients with myelin oligodendrocyte glycoprotein antibody–associated disease (MOGAD), neuromyelitis optica spectrum disorder (NMOSD), and multiple sclerosis (MS)–associated optic neuritis to improve early differential diagnosis.

**Design**: Cross-sectional, multicenter, retrospective case-control study performed at the Department of Ophthalmology and Department of Neuroimmunology of the Ludwig Maximilians-University, Munich, as well as at the Department of Neuroophthalmology, Moorfields Eye Hospital, London.

**Methods**: All patients diagnosed with LHON, MOGAD, NMOSD or MS-associated optic neuritis, for whom an OCT scan was available within 21 days after symptom onset were included. OCT was used to quantify pRNFL and mGCIPL thicknesses. Patients were stratified into the four above mentioned etiological groups, and OCT metrics in LHON were directly compared with those of the inflammatory optic neuritis groups to assess disease-specific structural patterns.

**Results**: 12 eyes of 9 patients with LHON, 14 eyes of 10 patients with NMOSD/MOG, and 16 eyes of 16 patients with MS-associated optic neuritis were included. The interval between symptom onset and OCT acquisition did not differ significantly between groups (14.6 days for LHON, 13.7 days for NMOSD/MOG (p=0.416), 12 days for MS-associated optic neuritis (p=0.317)). GCIPL thickness differed significantly across groups, with LHON patients showing significantly smaller GCIPL thickness (536.08µm) compared with MOGAD/NMOSD (733.03µm, p<0.0001), and MS optic neuritis (760.81µm, p<0.0001). In contrast, overall pRNFL thickness was significantly greater in the NMOSD/MOG subgroup (1051.8µm) compared to the LHON subgroup (771.78µm, p=0.00).

**Conclusions**: In the acute stage of optic neuropathy, OCT reveals distinct retinal structural profiles that may help differentiate LHON from immune-mediated optic neuritis, including severe inflammatory phenotypes such as NMOSD and MOGAD. Pronounced GCIPL thinning in acute LHON may reflect early ganglion cell loss, whereas relatively increased pRNFL thickness in acute NMOSD/MOG optic neuritis may relate to inflammatory edema. These findings support OCT as a valuable adjunct for early diagnostic discrimination between mitochondrial and demyelinating optic neuropathies.

102. **Adalimumab as a novel potential risk factor for Posterior Ischaemic Optic Neuropathy**

Luca Seregni^1^, Antonio Calcagni^2^, Ailbhe Burke^3^

^1^Fondazione IRCCS Policlinico, Milan, Italy; Moorfields Eye Hospital, London, UK; ^2^Moorfields Eye Hospital, London, UK; University College London, London, UK; ^3^Moorfields Eye Hospital, London, UK; National Hospital for Neurology & Neurosurgery Queen Square, London, UK

**Introduction**: A 49-year-old female patient presented with an unusual right optic neuropathy. She was investigated extensively and a novel diagnosis of adalimumab induced posterior ischaemic optic neuropathy (PION) is proposed.

**Case Report**: A 49-year-old female patient presented to the Eye Casualty with severe, painless visual loss in the right eye upon awakening, without premonitory symptoms.

She had a history of seronegative inflammatory arthritis treated with adalimumab, as well as burning sensation in the right thigh onset 18 months prior and right trigeminal paraesthesiae for 6 months.

Visual acuity was counting fingers with dense right relative afferent pupillary defect. Ocular motility was full and painless. Dilated slit lamp examination was unremarkable, with no cells and no disc or fundus abnormalities.

High-dose oral methylprednisolone (1200 mg daily for 5 days) was initiated on the day of symptom onset followed by oral prednisolone taper, and she was referred acutely to Neurology for further investigations.

OCT was normal, including ganglion cell volume and nerve fibre layer thickness, without inner retinal ischaemic changes or subretinal fluid.

Electrophysiological findings were more typical for an ischaemic rather than a demyelinating aetiology, with subnormal but not delayed pattern reversal and flash VEPs in the right eye, and no significant interocular pattern ERG and full field ERG asymmetry.

Orbital MRI with contrast the following day was normal. Subsequent MRI brain confirmed multiple periventricular T2-hyperintense lesions suspicious for MS, while spinal MRI was normal.

Follow-up OCT 20 days later showed isolated progressive right optic atrophy and subsequent fluorescein and indocyanine green angiography were normal.

Cerebrospinal fluid was acellular, with normal protein and glucose; oligoclonal bands are pending. Serum anti-aquaporin-4 and anti-MOG antibodies were negative. A wider infection and inflammation screen was also negative, except for weakly positive anti-β2 glycoprotein antibodies and positive ANCA with negative MPO/PR3.

Vision did not improve despite high-dose corticosteroid therapy. Escalation to intravenous steroids and plasma exchange was proposed to the treating team but not pursued.

A clinical diagnosis of PION was made. Rheumatology is investigating adalimumab-induced anti-phospholipid syndrome. Co-morbid multiple sclerosis is suspected as the cause of her sensory symptoms and investigations to confirm this are ongoing.

**Discussion**: Adalimumab has been reported as a cause of ischaemic events including non-arteritic anterior ischaemic optic neuropathy but has not been previously associated with PION. In many of those cases the ischaemia was attributed to adalimumab-induced anti-phospholipid syndrome.

Adalimumab is also a well-known risk factor for multiple sclerosis (MS) exacerbation and may unmask clinically silent disease, as appears to be the case here. We note also that APS can mimic MS. Further investigations are planned to clarify this prior to presentation at EUNOS.

**Disclosures**: None

104. **Improvement of diabetic papillopathy with intravitreal anti-vascular endothelial growth factor: A case report**

Mambretti Manuela^1^, Bartoccini Matilde^1^, Covelli Antonio^2^, Radice Paolo^1^

^1^Ophthalmology Department, Ospedale di Circolo e Fondazione Macchi, ASST Sette Laghi, Varese, Italy; ^2^Department of Neurology and Stroke Unit, Ospedale di Circolo e Fondazione Macchi, ASST Sette Laghi, Varese, Italy

**Introduction**: Diabetic papillopathy is a optic nerve disease linked to rapid change in glycemic control in diabetes. We present the case of a patient who presented with worsening diabetic papillopathy in the left eye and was successfully managed with anti-vascular endothelial growth factor (VEGF) treatment.

**Case Report**: A 25-year-old woman with type I diabetes mellitus presented with asymptomatic disk edema in the left eye.

Six months before, she had been evaluated for painless acute visual loss with disk edema in the fellow eye, which had been noted following a night of Methylenedioxymethamphetamine (MDMA) and alcohol abuse, in the setting of poorly controlled diabetes. The edema resolved spontaneously but resulted in persistent inferior visual field defect. When she presented for a follow-up visit, she was asymptomatic in the left eye, with 20/20 visual acuity bilaterally and no color disturbances. Visual field testing revealed a stable defect in the right eye and normal retinal sensitivity in the left eye. Fundus examination showed optic disk pallor in the right eye and blurred disk margins in the left eye, along with vessel tortuosity, flame hemorrhages and microaneurysms. As she reported recent improvement in glycemic control, a diagnosis of diabetic papillopathy was made.

In the following months, however, the disk edema of the left eye continued to worsen until she presented to the emergency room with visual acuity of 20/25 in the left eye and mild dyschromatopsia. Fluorescein angiography in the left eye also demonstrated diffuse leakage and pre-ischemia changes in the retinal periphery. Due to concern for progression of optic nerve disease and neovascular risk, a single intravitreal antiVEGF injection was administered. Improvement of visual acuity, disk edema and retinopathy was noted at follow up 2 weeks later. Findings remained stable at 6-month follow-up.

**Discussion**: Diabetic papillopathy has typically been associated with minimal visual symptoms and a benign course. Permanent sequela, however, have been described and management should be individualized. Treatment with anti-VEGF has been shown in case reports and small case series to improve diabetic papillopathy, possibly by reducing permeability of epipapillary vessels. Systemic studies are needed to identify patients at risk of visual loss from diabetic papillopathy and to evaluate the effect of antiVEGF on optic nerve disease.

**Disclosures**: No disclosures to declare.

108. **Assessment and quantification of RAPD using slit lamp**

Marko Hawlina

Eye Hospital, University Medical Centre Ljubljana, Ljubljana, Slovenia

**Introduction**: Since the first publication in 2016, we are assessing relative afferent pupillary defect (RAPD) using a slit lamp. Assessment of RAPD using hand lamp or direct ophthalmoscope may be difficult in patients with small pupils, very slight RAPD or dark iris, or with iris abnormalities that may be overlooked without slit lamp examination.

**Methods**: Slit lamp of Haag-Streit type was used in which the width of the slit is set to 1 mm (mark 10) whilst the height of the slit is precisely set first to vertical diameter of the pupil of the affected eye. The light is directly shone into the eye to overcome accommodative component by the strong light reflex. Then the slit is quickly swung to the unaffected eye and the height of the slit on the non-affected pupil is adjusted to the edges of the pupil and compared to affected eye. Differences were measured and compared with pupillography in patients with various uniocular optic neuropathies.

**Results**: If the RAPD was present, it was clearly visible under the slit lamp magnification and could be accurately measured. In none of the cases was this method less accurate in comparison with hand lamp or direct ophthalmoscope and accommodation component was suppressed. Slit lamp enabled more accurate quantification of RAPD and highly correlated with pupillography results. Clinical examples will be presented.

**Discussion**: Assessment of RAPD using slit lamp is a reliable and simple method of testing of this important clinical sign in every ophthalmologists practice. Visualization under slit lamp magnification increases the accuracy of pupil observation, especially in ambiguous cases, and provides a simple method of quantification of RAPD. In the same time, this method provides detection of eventual abnormal structure or function of the iris that may not be apparent with conventional swinging light test.

**Disclosures**: No conflicts of interest to disclose.

109. **Discovery of PHB1 as a novel candidate gene in dominant optic atrophy**

Martina Jarc Vidmar^1^, Ales Maver^2^, Marko Hawlina^1^, Borut Peterlin^2^, Ana Fakin^1^, Lea Kovac^1^, Maja Sustar Habjan^1^, Lucija Malinar^1^, Sanja Petrovic Pajic^3^, Tanja Visnjar^2^, Nusa Trost^2^, Rok Romih^2^, Urska Dragin^2^

^1^Eye Hospital, University Medical Centre, Ljubljana, Slovenia; ^2^Clinical institute of genomic medicine, University Medical Centre, Ljubljana, Slovenia; ^3^Eye Hospital, University Medical Centre, Belgrade, Serbia

**Introduction**: Hereditary optic neuropathies represent a genetically heterogeneous group of disorders caused by pathogenic variants in mitochondrial and nuclear genes. Although the diagnostic yield in patients with hereditary optic neuropathies is increasing, a notable proportion of patients remains undiagnosed. Our aim is to report on the novel candidate heterozygous variant in the PHB1 (Prohibitin 1) gene in a large family affected by autosomal dominant optic neuropathy.

**Methods**: A three-generation family with five members affected by optic neuropathy, five healthy members and one unrelated age-matched control was examined by neuroophalmologists and recruited for genetic counseling. Affected patients exhibited slowly progressive visual acuity loss and all signs of optic neuropathy (impaired colour vision, central scotoma in the visual field, retinal nerve fiber layer and ganglion cell layer thinning on optical coherence tomography and abnormal electrophysiology pattern ERG and visual evoked potentials testing). Pedigree analysis suggested an autosomal dominant inheritance pattern. The whole exome and whole genome-based linkage mapping were performed to identify possible genetic cause, while protein modelling and in vitro experiments have been carried out for supporting evidence

**Results**: Family-based WGS linkage mapping identified a heterozygous missense variant c.440C>T (p.Ser147Phe) in the PHB1 gene in all five affected members with optic atrophy. The identified variant results in the p.Ser147Phe amino acid substitution in the evolutionarily conserved alpha-helix domain of the PHB protein, a conserved mitochondrial protein with multiple functions. In silico structural modelling revealed that the p.Ser147Phe in the PHB1 protein is likely to disrupt protein stability and function due to loss of hydrogen bonding, steric hindrance and altered hydrophobic interactions. In vitro experiments indicate changes in mitochondrial dynamics in family members carrying the PHB1 variant compared to those without, resulting in an altered ratio of L-OPA1 to S-OPA1.

**Discussion**: We report a novel candidate gene PHB1 gene that may be associated with the dominant optic atrophy. GS mapping identified a variant c.440C>T in the PHB1: gene, that is encoding prohibitin 1, an evolutionarily conserved, multifunctional protein that is present in various cellular compartments. This is the first report of the PHB1 gene associated with a Mendelian disease, and further studies are needed to confirm our findings.

**Disclosures**: All authors have nothing to disclose.

113. **Gross bilateral optic disc swelling with immune checkpoint inhibitor use in oesophageal adenocarcinoma: Leptomeningeal spread vs immune-related neurotoxicity?**

Matthew Mo^1^, Guillaume Mignot^2^, Amna Irfan^2^

^1^Royal Victoria Hospital, Northern Ireland; ^2^Aberdeen Royal infirmary, NHS Grampian, Scotland

**Introduction and Case report**: We present an unusual case of an asymptomatic 69-year gentleman with a background of oesophageal adenocarcinoma, who had been treated with eight cycles of FOLFOX (leucovorin, fluorouracil, oxaplatin) and nivolumab. He presented to our eye emergency department with gross haemorrhagic bilateral optic nerve swelling with a visual acuity of 6/9 in both eyes. The patient had been referred during a routine community optometry check and denied any visual symptoms or symptoms suggestive of raised intra-cranial pressure. Enhanced depth optical coherence tomography demonstrated an up-going Bruch’s membrane in both eyes. CT venogram and magnetic resonance imaging (MRI) of his brain and orbits was unremarkable and a lumbar puncture demonstrated a normal opening pressure. However, he had markedly raised cerebrospinal fluid protein (2674 mg/L) in addition to pleocytosis and raised glucose (7.6 mmol/L) levels. Cytology analysis showed few scattered lymphocytes (low cellularity sample) with positive staining with CD45. These showed positivity with Ber EP4, CK20 and CK 7 (weak).

The patient’s condition rapidly deteriorated thereafter and he sadly passed away one month later.

**Discussion**: When our patient was initially seen in our urgent access ophthalmology clinic, the grossly swollen appearance of his optic nerves and up-going Bruch membranes raised the spectre of papilloedema secondary to intracranial metastases or CSF obstruction.

With normal imaging and normal opening pressure, leptomeningeal spread became the most likely differential diagnosis to consider. However, this diagnosis can be elusive as it may not show any changes even on MRI imaging.

With normal imaging, the use of immune checkpoint inhibitors causing immunotherapy-related encephalitis could not be excluded. Both differentials were very important to consider in this case as they require different management. In leptomeningeal spread, cancer cells infiltrate the meninges and subarachnoid peri-optic space causing disruption of the blood-CSF barrier leading to an inflammatory response, venous congestion and protein leakage into the CSF. On the other hand, immune checkpoint inhibitors may cause an immune-related inflammatory reaction leading to CNS inflammation and bilateral optic nerve inflammation. Both conditions may present with pseudo-papilloedema, unremarkable neuroimaging and markedly raised CSF protein. In our case, cytology of the CSF and the patient’s poor clinical course suggested leptomeningeal spread.

**Conclusion**: This case demonstrates the challenges that come with diagnosing patients with asymptomatic haemorrhagic bilateral optic nerve swelling in the context of oncological disease, receiving both cytotoxic and immune checkpoint inhibitor medications. It is important for ophthalmologists to bear these diagnoses in mind and communicate their suspicion to neurologists, since imaging may be normal and diagnosis often requires a large volume of CSF due to low cellularity.

**Financial disclosures:** None.

115. **Optic disc edema in APMPPE**

Michele Fantini

Department of Ophthalmology, Hospital IRCCS Sacro Cuore Don Calabria, Negrar di Valpolicella (VR), Italy

**Introduction**: Acute Posterior Multifocal Placoid Pigment Epitheliopathy (APMPPE) is a rare, self-limited disease typically affecting both eyes of men and women at the age of 20-40. It is characterized by vision loss, central or paracentral scotomas and placoid lesions at the retinal pigment epithelium (RPE). This report describes an uncommon case of an elderly woman presenting with unilateral APMPPE complicated with optic disc edema.

**Case Report**: An 83-year-old woman came to the Ophthalmology Department because of progressive and painless loss of vision in her right eye. She presented with otherwise good health condition, taking only medications for systemic hypertension. Best corrected visual acuity (BCVA) was 20/63 OD and 20/25 OS. Intraocular pressures were 17 mmHg OD and 11 mmHg OS. Mild cataracts were noted in OU. Fundoscopy of the right eye revealed optic disc edema and yellowish subretinal deposits at the posterior pole. The examination of the left eye’s fundus was unremarkable. A small central scotoma OD was noted with Humphrey 24-2 Visual Field Test. Optical Coherence Tomography (OCT) evaluation showed subretinal hyperreflective macular deposits OD; no lesions were noted in the left eye.

Fluorescein angiography OD showed optic disc’s leakage due to papillary edema. Multiple hypofluorescent dark dots were noted in the macular region of the left eye in the early phase of indocyanine green angiography.

Blood tests revealed a slight elevated (6,03 mg/L) C-reactive Protein and slight elevated lysozyme (17,4 mg/L). Quantiferon Tubercolosis Test was negative. High-Resolution Chest Computed Tomography (HRCT) was unremarkable. Diagnosis of APMPPE complicated with optic disc edema was made. No treatment was prescribed to the patient.

BCVA OD spontaneously improved to 20/32 in one week. Optic disc edema disappeared after two months. The yellowish subretinal deposits of the macula slowly faded after 6 months. No macular- or retinal nerve fiber layer atrophy remained afterwards.

**Discussion**: APMPPE is a rare inflammatory chorioretinopathy classified among the White Dot Syndromes. The characteristic findings of this disease are multiple yellow- or grey-placoid lesions of the RPE. It is typically bilateral and most common in the second to fourth decade of life. APMPPE is self-limited, with visual recovery in weeks. The pathogenesis is still uncertain; inflammation of RPE and occlusive vasculitis are the prevailing theories. Association with optic disc edema has been reported in a few case reports. The advanced age of this patient and the unilateral presentation represent another atypical feature of this disease.

**Disclosures**: The author has no financial disclosures.

118. **Pediatric Myelin Oligodendrocyte Glycoprotein associated optic neuritis presenting with pre-retinal haemorrhage and raised intracranial pressure**

Marco Piergentili^1^, Vasiliki Panteli^1^, Cheryl Hemingway^1^, Richard Bowman^1^

^1^Clinical and Academic Department of Ophthalmology, Great Ormond Street Hospital for Children NHS Foundation Trust and UCL Great Ormond Street Institute of Child Health, London, UK

**Introduction**: Myelin Oligodendrocyte Glycoprotein antibody–associated disease (MOGAD) is an important cause of optic neuritis in children, frequently presenting with bilateral acute visual loss with optic disc oedema. Although retinal haemorrhages have been described in MOG-associated optic neuritis, large subhyaloid haemorrhage (SH) is exceptionally rare and may mask the underlying inflammatory optic neuropathy. We report a paediatric case of bilateral MOG-associated optic neuritis presenting with SH and raised intracranial pressure.

**Case Report**: A previously healthy 7-year-old boy presented with four days of visual loss in the right eye associated with peri-ocular pain and headaches. There was no history of trauma or recent infection. Visual acuity was light perception in the right eye. Fundus examination showed a dense premacular SH. Blood tests, including full blood count, coagulation profile, inflammatory markers, renal and liver function, were normal.

One week later, he represented with acute visual loss in the left eye. Visual acuity was light perception in the right eye and hand movements in the left. Fundoscopy demonstrated bilateral optic disc oedema with persistent right SH. Magnetic resonance imaging of the brain and orbits showed bilateral enlargement and T2-FLAIR hyperintensity of the intraorbital optic nerves with posterior globe flattening, consistent with bilateral optic neuritis. Lumbar puncture revealed an opening pressure of 31.5 cmH_2_O (normal <25cm) with otherwise normal cerebrospinal fluid.

The patient was treated with five days of intravenous methylprednisolone with early visual improvement. Anti-MOG antibodies returned at >1/1000, confirming MOGAD. Due to slow recovery, plasma exchange followed by intravenous immunoglobulin was administered.

At four months, visual acuity improved to 0.06 LogMAR in the right eye and 0.5 LogMAR in the left. Colour vision improved to 13/13 and 9/13 Ishihara plates, respectively. Fundoscopy showed complete resolution of the subhyaloid haemorrhage with mild bi-temporal optic disc pallor. Optical coherence tomography demonstrated bilateral asymmetric retinal nerve fibre layer and macular ganglion cell loss.

**Discussion**: Retinal haemorrhages and venous stasis retinopathy have been previously described in in MOG-associated optic neuritis. We describe to our knowledge, the first case of paediatric SH associated with bilateral MOG+ optic neuritis and raised intracranial pressure. SH are extremely rare in children and usually related to trauma or intracranial pathology. In the absence of history of trauma, haematological diseases must be excluded. It is also important to consider optic neuritis as a rare cause of SH since timely recognition of this entity and prompt treatment are crucial to improve visual outcomes. A Terson-like mechanism related to raised intracranial pressure, combined with severe inflammatory optic nerve swelling and venous outflow impairment, may explain this finding. In this context, the diagnosis of optic neuritis can be potentially overlooked by the presence of the SH and delay treatment. Prompt neuro-ophthalmic assessment and early treatment are crucial to optimise visual recovery.

**Disclosures**: The authors do not have any interests or disclosure to declare.

119. **A recurrence after decades of silence**

Mubarika Sami Sami

Epsom And St Helier Hospitals, UK

**Introduction**: We report a case of MOGAD optic neuritis which recurred after an unusually protracted interval of forty years. The previous longest interval between episodes reported is seventeen years.

**Case Report**: A fifty-eight-year-old patient presented with subacute reduced vision in his left eye and pain on eye movements.

He recalled similar unilateral symptoms when he was 18 years old which improved with steroids. There were no further episodes until the current one.

On examination his left visual acuity was 0.5 on the LOGMAR chart. He had a left RAPD. The colour vision was reduced to 9/15 Ishihara plates and there was a left inferior scotoma on HES. His left optic disc showed grade 4 swelling.

He was treated in the eye casualty department with PO methylprednisolone 500 mg OD for 5 days with PPI cover. His vision rapidly improved. The HVF and Ishihara were normal and there was no RAPD. The optic disc swelling resolved showing a very good steroid response.

His MRI head and orbits with GAD scan showed a long, enhanced segment of the left intraorbital and intracanalicular optic nerve. There were no brain lesions.

Blood investigations included FBC, serum inflammatory markers, CRP, ANA, ACE, syphilis serology, Quantiferon Gold were all normal. Aquaporin 4 antibody and anti MOG antibody were sent to Oxford.

Three weeks later his right vision was similarly reduced with pain on eye movement. He was treated by his GP with PO methylprednisolone 500mg OD for 5 days with PPI cover.

The vision improved but there was residual optic disc swelling.

Aquaporin 4Ab was negative while MOG antibody was reported weakly positive.

He was referred urgently to Neurology.

Further investigations included MRI spinal cord which showed no lesions and lumbar puncture with no CSF oligoclonal bands.

He was started on PO Prednisolone 1mg/kg/day and gradually weaned off over 4 months.

His vision remains stable and the right optic disc swelling resolved

**Discussion**: He has not had further recurrence since stopping treatment 9 months ago. In case of further recurrence, second line immunosuppressants such as Mycophenolate have been effective while slowly weaning off steroids.

Recurrent optic neuritis is characteristic of multiple sclerosis, neuromyelitis optica spectrum and anti MOG associated neurological diseases. The myelin oligodendrocyte glycoprotein (MOG) is expressed on the myelin sheath’s outer surface; therefore, it is likely to be targeted by antibodies.

MOG optic neuritis is more common in children and males and often bilateral. MOG Ig antibodies also cause encephalitis and transverse myelitis. Anti MOG optic neuritis is the most prevalent of these (41-60%). Combined optic neuritis and transverse myelitis occurs in only 6-21%.

**Disclosures:** None.

120. **An unusual painful optic neuropathy in severe axial myopia mimicking optic neuritis**

Kevin Muggler^1^, Tim Beltraminelli^1^, Aki Kawasaki^1^

^1^Department of Neuro-ophthalmology, Hôpital Ophtalmique de Jules - Gonin, Lausanne, Switzerland.

**Introduction**: Painful optic neuropathy is most commonly attributed to inflammatory or demyelinating optic nerve disorders. However, non-inflammatory mechanisms, including structural orbital abnormalities, may also cause optic nerve dysfunction and pain. Severe axial myopia is associated with marked globe elongation and distortion of optic nerve anatomy. We report an unusual case of painful optic neuropathy related to mechanical optic nerve distortion in a highly myopic patient, clinically mimicking optic neuritis and optic perineuritis.

**Case Report**: A 40-year-old woman with severe asymmetric myopia and untreated left amblyopia presented with recurrent left periocular pain lasting several hours, consistently provoked and exacerbated by eye movements, markedly relieved by minimizing ocular motility, without subjective visual loss. Examination showed best-corrected visual acuity (VA) of 1.0 in the right eye and 0.6 in the left eye, normal color vision, a left relative afferent pupillary defect (RAPD), normal intraocular pressure (IOP), and no significant proptosis.

Visual field (VF) testing revealed a mild central temporal scotoma in the left eye. Fundus examination demonstrated tilted optic discs with myopic staphyloma, more pronounced on the left. Visual evoked potentials (VEP) showed delayed P100 latency and reduced amplitude in the left eye. Cerebrospinal fluid analysis was normal, and serum and CSF testing for anti–aquaporin-4 (AQP4) and anti–myelin oligodendrocyte (MOG) glycoprotein antibodies was negative. Brain and spinal magnetic resonance imaging (MRI) revealed no demyelinating or inflammatory lesions. Orbital MRI demonstrated marked anteroposterior elongation of the left globe with a posterior myopic staphyloma and distortion of the optic nerve, without optic nerve or sheath enhancement. Axial length (AL) measured 29.25 mm in the left eye, and ultrasonography showed no posterior scleral thickening. High-dose corticosteroids and pregabalin were ineffective, whereas nonsteroidal anti-inflammatory drugs (NSAIDs) partially relieved pain. At one-year follow-up, visual acuity and color vision remained stable, with persistent but non-progressive VF abnormalities and VEP delayed P100 latency.

**Discussion**: This case illustrates a rare mechanical cause of painful optic neuropathy in severe axial myopia, closely mimicking inflammatory optic neuropathies. The absence of radiological inflammation, lack of inflammatory biomarkers, and failure to respond to corticosteroids argue strongly against optic neuritis or optic perineuritis. In contrast, extreme axial elongation with posterior staphyloma and oblique optic nerve exit provides a plausible anatomical substrate for gaze-related mechanical stress.

Although pain is not a typical feature of myopia-associated optic neuropathy, biomechanical studies demonstrate that eye movements generate increased strain on the optic nerve and peripapillary tissues in highly myopic eyes, particularly in the presence of posterior staphyloma. The posterior sclera and peri-optic region are innervated by trigeminal nociceptive fibers, including mechanonociceptive afferents capable of responding to mechanical deformation in the absence of overt inflammation. Mechanical interaction between the distorted optic nerve, peripapillary scleral ring, and posterior sclera may therefore explain gaze-evoked pain in this patient.

Visual stability over one year suggests that mechanically induced optic neuropathy in severe myopia may remain stable or progress slowly.

**Conclusion**: Mechanical optic nerve distortion should be considered in highly myopic patients presenting with painful optic neuropathy. Recognition of this entity is essential to avoid unnecessary treatment and to guide appropriate management and follow-up.

121. **OCT device agreement is parameter-specific and disease-dependent in optic disc drusen**

Christoph Mits^1^, Karl Kircher^2^, Elena, Terekhova^2^, Berthold Pemp^2^

^1^Department of Ophthalmology, KDO, Vienna Health Association, Vienna, Austria; ^2^Medical University of Vienna, Austria

**Background**: Cross-device comparability of optical coherence tomography (OCT)–derived optic nerve parameters is essential for clinical diagnosis and longitudinal monitoring. Measurement agreement may be affected by device-specific segmentation differences, particularly in eyes with structural pathology such as optic disc drusen (ODD). We evaluated cross-device comparability of retinal nerve fiber layer (RNFL) thickness and optic disc-derived area measurements and quantified the structural relationship between Bruch’s membrane opening minimum rim width (BMO-MRW) and neuroretinal rim area across two OCT platforms.

**Methods**: RNFL measurements obtained using Heidelberg Engineering Spectralis spectral-domain OCT (SD-OCT) and Carl Zeiss Meditec Cirrus high-definition OCT (HD-OCT) were compared using Passing–Bablok regression and Bland–Altman analysis with limits of agreement (LoA). Optic disc size comparability was assessed using Passing–Bablok regression between Bruch’s membrane opening (BMO) area and neuroretinal disc area. The relationship between BMO-MRW and rim area (distinct anatomical constructs) was assessed using Passing–Bablok regression as a descriptive structural association. Analyses were performed in 65 eyes of 30 participants (healthy: 17 eyes of 9 participants; ODD: 42 eyes of 21 participants). Multivariable analyses evaluated effects of age and axial length. Bland–Altman analysis was restricted to same-unit parameters (RNFL).

**Results**: RNFL agreement was disease dependent. In ODD eyes, Bland–Altman LoA were wide (−15.6 to +13.9 µm). In healthy eyes, LoA were narrower (+1.0 to +11.6 µm) but showed residual systematic offset. In the overall cohort, LoA remained wide (−12.4 to +15.6 µm). Disease status remained associated with RNFL device differences after adjustment for age and axial length. Optic disc size measurements demonstrated better cross-device comparability. Passing–Bablok regression yielded slope 0.97 (95% CI 0.82–1.20) in ODD eyes, 0.74 (95% CI 0.44–0.97) in healthy eyes, and 0.91 (95% CI 0.79–1.07) overall. For BMO-MRW versus rim area, Passing–Bablok regression demonstrated moderate and variable association (overall slope 0.75, 95% CI 0.52–1.02; ODD slope 1.12, 95% CI 0.61–1.90; healthy slope 0.61, 95% CI 0.34–1.50).

**Conclusions**: Cross-device comparability between Spectralis and Cirrus OCT is parameter dependent. RNFL thickness demonstrated disease-dependent systematic differences and limited agreement, whereas disc size demonstrated substantially better cross-device comparability. These findings support parameter-specific interpretation of OCT measurements across imaging platforms and highlight the importance of device consistency for longitudinal structural monitoring.

124. **When acute vision loss is not giant cell arteritis: A case of fungal sinusitis affecting the orbital apex**

Nuala Pepper^1^, Emily Stedman^2^, Ruth Batty^2^, Thomas Green^2^

^1^Foundation Year 2, United Lincolnshire Hospitals NHS Trust, Lincoln, UK; ^2^Sheffield Teaching Hospitals NHS Trust, Sheffield, UK

**Introduction:** Invasive fungal sinusitis is a potentially life-threatening infection that predominantly affects immunocompromised or diabetic patients. When fungal proliferation extends beyond the paranasal sinuses into the orbital apex, it can lead to abrupt visual loss, ophthalmoplegia, cranial neuropathies, and bony destruction. These early manifestations often overlap with more common causes of acute visual loss, such as giant cell arteritis (GCA), creating a diagnostic challenge.

**Case Report:** A 59-year-old woman with type 2 diabetes mellitus presented with sudden onset, complete visual loss in the left eye and left-sided headache originating retro-orbitally, radiating to the temple. She reported fatigue, shoulder discomfort and jaw pain. She was afebrile. Visual acuity was 6/6 in the right eye and no perception of light in the left, with a left relative afferent pupillary defect. Fundoscopy revealed normal optic discs, with no disc swelling or cherry-red spot in the left eye. ESR was 108 mm/h and CRP was 104 mg/L, with a normal platelet count.

A presumed diagnosis of posterior ischaemic optic neuropathy secondary to GCA was made. Intravenous methylprednisolone was commenced. The following day, new 2mm left proptosis, reduced left eye abduction, and decreased sensation in a V1 distribution with reduced corneal sensitivity were noted. Urgent MRI orbits revealed an opacified sphenoid sinus with enhancement at the left orbital apex suggesting intraorbital extension of sinus disease, with possible fungal aetiology. Steroids were discontinued, the planned temporal artery biopsy was cancelled and she was referred urgently to Ear, Nose and Throat specialists. She underwent functional endoscopic sinus surgery (FESS). Histological assessment of intraoperative samples detected invasive fungal sinusitis, with hyphae raising suspicion of *Mucoromycetes*. Later CT scans showed osteomyelitis with bone destruction at the left orbital apex and sphenoid sinus.

Over subsequent months she required two further FESS procedures with Amphotericin B wash out and wide debridement of the left ethmoid, maxillary and sphenoid sinuses. Although no fungal organism was cultured, she responded well to long-term antifungal systemic therapy directed by infectious disease specialists.

She developed complete left-sided ophthalmoplegia with ptosis and remains blind in the left eye, which now has optic atrophy. She subsequently developed exposure keratopathy secondary to lagophthalmos and an anaesthetic cornea, requiring regular ocular lubricants.

**Discussion**: Acute visual loss with elevated inflammatory markers should prompt consideration of GCA. However, in diabetic patients with significant pain, invasive fungal infection must also be included in the differential diagnosis. Early MRI is crucial to distinguish between these entities as treatments diverge sharply; GCA requires urgent high-dose steroids, whereas invasive fungal disease can worsen dramatically with steroid exposure.

Our case emphasises the importance of excluding optic nerve compression at the orbital apex in patients with an initial normal fundal examination, before a diagnosis of posterior ischaemic optic neuropathy is made.

125. **Sequential optic nerve injury in MOG antibody–associated optic neuritis: A mixed optic neuropathy**

Nuno Álvaro Silva^1^, Ricardo Reis^2^, Olinda Faria^1^, Ana Faria Pereira^1^

^1^Centro Hospitalar e Universitário de São João, Serviço de Oftalmologia, Porto, Portugal; ^2^Centro Hospitalar e Universitário de São João, Serviço de Neurologia, Porto, Portugal

**Introduction**: Myelin oligodendrocyte glycoprotein antibody–associated optic neuritis (MOGAD-ON) often follows an atypical clinical course when compared to multiple sclerosis–associated optic neuritis. Although less frequent, unilateral involvement is well described in adults aged 20–40 years. Disease activity correlates with MOG-IgG seropositivity.

Often the optic nerve may be simultaneously exposed to additional pathological insults, resulting in clinical features that do not fit neatly within a single category. These cases may be better understood as representing diagnostic and pathophysiological overlaps between different types of optic neuropathy. Such borderline presentations pose significant diagnostic challenges and remain insufficiently characterized in literature and require further recognition.

**Case Report**: A 33-year-old female presented to the emergency department with a 3-day history of decreased vision in the left eye (OS), described as a central white scotoma, associated with ocular pain on eye movements and dyschromatopsia. Upon initial examination, best-corrected visual acuity (BCVA) was 7/10 OS with no relative afferent pupillary defect (RAPD), and no optic disc edema. Given the relatively preserved visual acuity and absence of fundoscopic abnormalities, she was discharged with close outpatient follow-up.

Ten days later, she was re-evaluated due to visual deterioration and progression to an enlarged dark central scotoma. At that time BCVA had declined to 3/10 OS and a mild RAPD was detected with persistent absence of optic disc edema.

Orbital magnetic resonance imaging demonstrated diffuse T2 hyperintensity and enhancement of the left optic nerve. Serum testing revealed positivity for MOG-IgG antibodies.

High-dose intravenous corticosteroid therapy was administered for 5 days, resulting in significant improvement.

Over a two-year follow-up period, after subsequent seroconversion to MOG-IgG negativity, progressive bilateral optic disc cupping enlargement was observed, accompanied by corresponding retinal fiber layer (RNFL) thinning, predominantly in inferotemporal sectors. At this stage, a detailed anamnesis revealed a positive family history of glaucoma. Bruch´s membrane opening-minimum rim with (BMO-MRW) analysis demonstrated markedly reduced values bilaterally, supporting the presence of a concomitant glaucomatous optic neuropathy. Intraocular pressure was 18 mmHg in the right eye and 16 mmHg in the OS.

Topical prostaglandin analogue therapy was initiated accordingly, leading to stabilization and reduction in the rate of RNFL loss.

**Discussion**: Retrobulbar optic neuritis may be overlooked at initial presentation due to minimal fundoscopic findings and relatively preserved visual acuity. In this case, following recovery from MOGAD-ON and seroconversion, progressive cupping and RNFL/BMO-MRW changes suggested glaucomatous optic neuropathy rather than persistent inflammatory damage. This case supports a sequential model of optic nerve injury in which MOGAD-ON acted as a precipitating event, with subsequent exposure to additional mechanisms that individually might have limited functional impact but collectively contributed to a mixed optic neuropathy. Long-term structural monitoring is therefore essential in patients with MOGAD, even after apparent clinical recovery.

126. **Positive 5-year benefit/risk ratio of bilateral injection of Lenadogene nolparvovec gene therapy for Leber hereditary optic neuropathy**

Nancy J. Newman^1^, Patrick Yu-Wai-Man^2^, Prem S. Subramanian^3^, Sarah Thornton^4^, An-Guor Wang^5^, Sean P. Donahue^6^, Bart P. Leroy^7^ , Valerio Carelli^8^, Valérie Biousse^1^, Catherine Vignal-Clermont^9^, Alfredo A. Sadun^10^, Robert C. Sergott^4^, Gema Rebolleda Fernández^11^, Bart K. Chwalisz^12^, Rudrani Banik^13^, Magali Taiel^14^, José-Alain Sahel^15^, for the LHON REFLECT Study Group.

^1^Departments of Ophthalmology, Neurology and Neurological Surgery, Emory University School of Medicine, Atlanta, GA, USA; ^2^John van Geest Centre for Brain Repair and MRC Mitochondrial Biology Unit, Department of Clinical Neurosciences, University of Cambridge, Cambridge, UK; ^3^Sue Anschutz-Rodgers University of Colorado Eye Center, University of Colorado School of Medicine, Aurora, CO, USA; ^4^Departments of Neurology and Ophthalmology, Wills Eye Hospital and Thomas Jefferson University, Philadelphia, PA, USA; ^5^Department of Ophthalmology, Taipei Veterans General Hospital, National Yang Ming Chiao Tung University, Taipei, Taiwan; ^6^Department of Ophthalmology, Neurology, and Pediatrics, Vanderbilt University, and Vanderbilt Eye Institute, Vanderbilt University Medical Center, Nashville, TN, USA; ^7^Department of Ophthalmology and Center for Medical Genetics, Ghent University Hospital, and Department of Head & Skin, Ghent University, Ghent, Belgium; ^8^IRCCS Istituto delle Scienze Neurologiche di Bologna, Programma di Neurogenetica, Bologna, Italy; ^9^Department of Neuro Ophthalmology and Emergencies, Rothschild Foundation Hospital, Paris, France; ^10^Doheny Eye Institute, UCLA School of Medicine, Los Angeles, CA, USA; ^11^Department of Ophthalmology, Alcala University, Madrid, Spain; ^12^Department of Ophthalmology, Massachusetts Eye & Ear, Harvard Medical School, Boston, MA, USA; ^13^Department of Ophthalmology, Icahn School of Medicine at Mount Sinai, New York, NY, USA; ^14^GenSight Biologics, Paris, France 15. Department of Ophthalmology, University of Pittsburgh School of Medicine, Pittsburgh, PA, USA

**Introduction**: Lenadogene nolparvovec (LN) is an AAV2-based gene therapy for patients with Leber hereditary optic neuropathy (LHON) carrying the m.11778G>A MT-*ND4* variant. REFLECT (NCT03293524) is a clinical study designed to evaluate the efficacy and safety of bilateral injection of LN gene therapy in patients with MT-*ND4* LHON.

**Methods**: This is a randomized, double-masked, placebo-controlled phase 3 study. The first-affected eye received LN gene therapy; the other eye (second-affected or not yet affected) was randomly injected with LN or placebo. The final data 5 years after LN administration are presented.

**Results**: Forty-eight patients were treated bilaterally and 50 unilaterally. The mean improvement in best-corrected visual acuity (BCVA) from nadir to 5 years was -0.38 (+19 ETDRS letters) and -0.35 (+18 ETDRS letters) LogMAR in the first and second-affected eyes of bilaterally treated patients, and -0.40 (+20 letters) and -0.28 (+14 letters) LogMAR in the first and second-affected eyes of unilaterally treated patients. Clinically relevant recovery from nadir in at least one eye of patients at 5 years was observed in 75% of bilaterally treated patients and 60% of unilaterally treated patients. An improvement of at least 0.3 LogMAR (+15 letters) from nadir in at least one eye was achieved in 69% of patients receiving bilateral LN injections and 66% of patients receiving unilateral LN injections. Overall, LN safety was favorable, with no difference in the incidence of adverse events between bilaterally and unilaterally treated patients, except for ocular inflammation occurring in 65% of treated eyes, usually mild and resolving with conventional treatment.

**Discussion**: The final results at 5 years confirm the long-term benefit/risk ratio of LN gene therapy for patients with MT-*ND4* LHON. Bilateral administration of LN provided additional benefit compared with unilateral administration of LN.

**Disclosures**: NJN is a consultant for GenSight Biologics, Chiesi, Stoke Therapeutics, Eli Lilly and Phelcom; has received research support from GenSight Biologics. PYWM is a consultant for GenSight Biologics, Chiesi and Neurophth Therapeutics. PSS is a consultant for GenSight Biologics. BPL is a consultant for GenSight Biologics and has received research support from GenSight Biologics. VC is a consultant for GenSight Biologics, Chiesi, and Pretzel Therapeutics. VB is a consultant for GenSight Biologics, Chiesi, Topcon and Phelcom. CVC is a consultant for GenSight Biologics and Chiesi. AAS is a consultant for GenSight Biologics and Chiesi. RCS is a consultant for GenSight Biologics. MT is an employee of GenSight Biologics. JAS is a consultant for Avista Therapeutics, Essilor Luxottica and Lightstone ventures; is owner/co-owner/founder/co-founder and has personal financial interests (stock/stock options) in Gensight Biologics, Sparing Vision, Prophesee, Tilak Healthcare, Avista, Tenpoint, SharpEye, Cilensee and Netramind; is a member of the scientific advisory board of Gilbert Foundation, Foundation Fighting Blindness and Institute of Ophthalmology Basel; is an observer at Sparing Vision and Avista; is president of Fondation Voir et Entendre and StreetLab; is director of GenSight Biologics; is executive Vice-President of UPMC Enterprises; has patents for allotopic expression, Rod-Derived Cone Viability Factor and related patents; is a recipient for patent royalties and GenSight Biologics.

127. **FALCON natural history study: Identifying sensitive measures of change in *OPA1‑*associated ADOA**

Piero Barboni^1^, Marc Bouffard^2^, Julie Falardeau^3^, Chiara La Morgia^4^, Byron Lam^5^, Michael Larsen^6^, Yaping Joyce Liao^7^, Raghu Mudumbai^8^, Marcela Votruba^9,10^, Patrick Yu-Wai-Man^11–14^, Alice Wyse Jackson^15^, Kelly Saluti^15^, Charles Liu^15^, Barry Ticho^15^, Steven Gross^15^

^1^IRCCS San Raffaele Scientific Institute, Milan, Italy; ^2^Massachusetts Eye and Ear Infirmary, Harvard Medical School, Boston, MA, USA; ^3^Casey Eye Institute, Oregon Health & Science University, Portland, OR, USA; ^4^Department of Biomedical and Neuromotor Sciences, University of Bologna, Bologna, Italy; ^5^Miller School of Medicine, University of Miami, Miami, FL, USA; ^6^Rigshospitalet, University of Copenhagen, Copenhagen, Denmark; ^7^Byers Eye Institute, Stanford University, Palo Alto, CA, USA; ^8^Department of Ophthalmology, University of Washington, Seattle, WA, USA; ^9^School of Optometry and Vision Sciences, Cardiff University, Cardiff, UK; ^10^Eye Clinic, University Hospital Wales and Cardiff University, Cardiff, UK; ^11^John van Geest Centre for Brain Repair and MRC Mitochondrial Biology Unit, Department of Clinical Neurosciences, University of Cambridge, Cambridge, UK; ^12^Cambridge Eye Unit, Addenbrooke’s Hospital, Cambridge University Hospitals NHS Foundation Trust, Cambridge, UK; ^13^Moorfields Eye Hospital NHS Foundation Trust, London, UK; ^14^Institute of Ophthalmology, University College London, London, UK; ^15^Stoke Therapeutics, Bedford, MA, USA

**Introduction**: Autosomal dominant optic atrophy (ADOA) is the most common hereditary optic neuropathy with a prevalence between 1:12,000 to 1:50,000. Approximately 70% of ADOA cases are caused by *OPA1* variants that lead to reduced OPA1 protein expression and mitochondrial dysfunction. Retinal ganglion cells are preferentially affected, which ultimately results in progressive optic nerve degeneration and visual impairment. Here, we report results from FALCON, a multicenter natural history study that assessed functional and structural changes in the retina and optic nerve of patients with *OPA1* ADOA over 24 months.

**Methods**: FALCON enrolled 48 patients with ADOA across 10 study sites. Patients were aged 8–60 years and had genetically confirmed heterozygous *OPA1* variants. Key primary endpoints included change from baseline to 24 months in peripapillary retinal nerve fiber layer (pRNFL) thickness and best-corrected high-contrast (HCVA) and low-contrast (LCVA) visual acuity.

**Results**: At screening, the mean patient age was 28.1 years and mean HCVA was 0.57 logMAR. Of the 47 patients analyzed, minimal changes from baseline were observed in pRNFL thickness and HCVA over 24 months, and no patients demonstrated improvements in best-corrected visual acuity over the study duration. By contrast, a modest decline in LCVA was observed over 24 months, with a median Early Treatment Diabetic Retinopathy Study (ETDRS) letter change of −0.5 at Month 24. Of the 8/33 patients (24%) who experienced ≥5 LCVA ETDRS letter loss at Month 24, no significant changes in pRNFL thickness were detected over 24 months. For all visits combined, temporal and inferior temporal pRNFL thickness were correlated with HCVA and LCVA (*P*<0.0001 for both correlations).

**Discussion**: The FALCON prospective natural history study is the first to systematically assess, over a 2-year period, functional and structural biomarkers of ADOA. The data presented here indicate that, although ADOA progresses slowly, measurable functional decline can be detected over 24 months. Correlations between pRNFL sectors and HCVA highlight the association between neural integrity and functional impairment. The decline in LCVA over 24 months in the absence of changes in retinal anatomy suggest that short‑term LCVA decline may reflect reversible cellular dysfunction. Overall, the data support LCVA as a sensitive, clinically meaningful endpoint for detecting potential treatment efficacy in ADOA.

**Disclosures:**

Piero Barboni: Clinical trial support from Stoke Therapeutics. Marc Bouffard: Clinical trial support from Stoke Therapeutics. Julie Falardeau: Clinical trial support from Stoke Therapeutics. Chiara La Morgia: Clinical trial support from Stoke Therapeutics. Byron Lam: Research support from Stoke Therapeutics. Michael Larsen: Consulting for and clinical trial support from Stoke Therapeutics. Yaping Joyce Liao: Consulting for Stoke Therapeutics. Raghu Mudumbai: Clinical trial support from Stoke Therapeutics. Marcela Votruba: Consulting for and speaker honoraria from Stoke Therapeutics. Patrick Yu-Wai-Man: Consulting for Stoke Therapeutics. Alice Wyse Jackson, Kelly Saluti, Charles Liu, Barry Ticho, and Steven Gross are employees of Stoke Therapeutics.

128. **Peripapillary hyperreflective ovoid mass-like structures in a myopic adolescent mimicking papilledema**

P. Giannopoulos^1^, W.A.J. Birkhoff^1^, G. Thepass^1^, J.A.M. van Everdingen^1^, T. Lushchyk^1^

^1^Rotterdam Eye Hospital, Department of Neuro- and Paediatric Ophthalmology, Rotterdam, Netherlands

**Introduction**:Peripapillary hyperreflective ovoid mass-like structures (PHOMS) are increasingly recognized findings on enhance depth imaging optical coherence tomography (EDI-OCT) that may simulate papilledema, particularly in myopic children and adolescents. Differentiating PHOMS from true papilledema or optic disc drusen (ODD) is essential to prevent misdiagnosis and unnecessary neurological investigations. We report a case of bilateral PHOMS in a myopic adolescent evaluated at our tertiary referral centre.

**Case Report**: A 15-year-old female with a history of myopia was referred to our tertiary ophthalmology centre following a relocation. She had previously been treated with atropine eye drops for myopia control, which were discontinued one year earlier because of systemic side effects. There was no relevant medical history, and family history was notable for mild myopia and a maternal grandparent with retinal detachment. Upon history taking, the patient reported symptoms of blurriness, headache and dizziness.

On examination, visual acuity was reduced accompanied by a refractive error. Anterior segment examination was unremarkable in both eyes. Fundoscopy revealed bilaterally full optic discs with mildly blurred inferior margins, without haemorrhages or exudates. Retinal vessels were normal, and no relative afferent pupillary defect was present. Multimodal imaging revealed no evidence of autofluorescence. Spectral-domain EDI-OCT demonstrated bilateral hyperreflective mass-like lesions external to- and surrounding large parts of the disc, without signs of true papilledema or subretinal fluid. B-scan ultrasound revealed markedly thin retrobulbar segment of the optic nerve and no hyperreflective material at the optic disc, thereby excluding ODD. These findings were consistent with PHOMS, and after refractive correction with new spectacles, visual acuity improved and headaches resolved completely.

At six-month and one-year follow-up, visual acuity and optic disc appearance remained stable, and EDI-OCT findings were unchanged. The patient remained asymptomatic, and an expectative follow-up strategy was continued.

**Discussion**: This case highlights the diagnostic challenge of distinguishing PHOMS from true papilledema in myopic paediatric patients. It is suggested that PHOMS are formed due to herniation of distended axons into the peripapillary retina and exoplasmic stasis and have been associated with a myopic shift during adolescence. EDI-OCT typically demonstrates PHOMS as strictly peripapillary, mass-like lesions positioned above Bruch’s membrane, producing an upward deflection of the overlying retinal layers. They are not visible on B-scan ultrasound despite their superficial location, in contrast to ODD. Although symptoms such as headache and dizziness may coexist and raise concern for increased intracranial pressure, they are not necessarily causally related. Recognition of the characteristic EDI-OCT appearance of PHOMS, combined with careful clinical assessment and follow-up, is essential to prevent misdiagnosis and avoid unnecessary neurological investigations.

**Disclosures**: The authors declare no conflicts of interest.

129. **When flow goes wrong, the eyes speak strong: Case report of advanced ocular ischemic syndrome**

Patrycja Duda^1^, Iwona Grabska-Liberek^1^

^1^Clinic of Ophthalmology, Medical Center of Postgraduate Education Prof. W. Orłowski Independent Public Clinical Hospital, Warsaw, Poland

**Introduction**: Ocular ischemic syndrome (OIS) is a condition associated with symptoms resulting from carotid artery stenosis or occlusion. We present a case of advanced OIS.

**Case report**: A 75-year-old Caucasian woman was referred from the neurology department for an ophthalmological consultation because of decreased visual acuity (VA) and retrobulbar pain in the left eye (LE) lasting for two months. She had a history of dizziness for several months, cigarette smoking and alcohol consumption. On clinical examination VA in the right eye (RE) was 0.2 and hand motions in the LE. Intraocular pressure was 10 mmHg in the RE and 5 mmHg in the LE. The LE also presented: restriction of ocular mobility in all directions, mild corneal edema, iris atrophy, slightly enlarged pupil, focal posterior synechiae and a pale optic disc. Although the patient did not report any symptoms in the RE, examination revealed cataract and very thin choroid in optical coherence tomography. Computed tomography angiography of the carotid and intracranial vessels showed advanced atherosclerotic changes, critical bilateral stenosis of the internal carotid arteries, absence of blood flow in the left external carotid artery and the left subclavian artery. The vascular surgeon disqualified the patient from surgical intervention due to the condition of the vessels and the high procedural risk.

**Discussion**: OIS can have many clinical manifestations. In the described case, both eyes suffered from OIS but presented different symptoms. These signs may mimic other diseases.

Therefore, OIS should be considered in differential diagnosis in many ophthalmological disorders.

**Disclosures:** none of the authors have conflicts of interest.

131. **Optic disc swelling in emergency ophthalmology: Diagnostic and management pathways.**

Joanna Przybek-Skrzypecka^1,2#^, Dominik Sokalski^1^, Jan Bombuy Gimenez^1^, Janusz Skrzypecki^3^,, Jacek P Szaflik^1,2^, Wolf Lagrèze^4^

^1^Department of Ophthalmology, Medical University of Warsaw, Poland; ^2^SPKSO Ophthalmic University Hospital, Warsaw, Poland; ^3^Department of Experimental Physiology and Pathophysiology, Medical University of Warsaw, Warsaw, Poland; ^4^Eye Center, Medical Center, Faculty of Medicine, University of Freiburg, Freiburg, Germany.

**Background**: Optic disc swelling is a frequent reason for referral. Rapid and accurate diagnosis is essential. However, diagnostic approaches vary considerably. The aim of this study was to evaluate the workflow and key clinical parameters influencing decision-making in the emergency setting.

**Methods**: We retrospectively analyzed a cohort of 262 patients referred with optic disc swelling to the emergency eye department of the Independent Public University Eye Hospital in Warsaw, Poland, from 2007 to 2025. Subgroups were stratified by laterality and visual acuity (Group 1 logMAR ≥ 1.0, Group 2 logMAR < 1.0).

**Results**: 71 patients had bilateral and 191 unilateral disc swelling. The mean age was lower in patients with bilateral compared to unilateral disc swelling (46 ± 20 vs. 59 ± 19 years), while the median symptom duration was 7 days in both groups. Most patients presented with any level of decreased visual acuity (n = 227, 87%) and visual field defects (n = 103, 39%), whereas general symptoms were less frequent and included headache (12.4%) and other complaints (6.2%), mainly paresthesia. Vascular changes (e.g. haemorrhages, venous dilation) of the posterior pole were observed in 83 patients (32%), with no significant difference between unilateral and bilateral presentations (34% vs. 25%, p = 0.23). A preliminary diagnosis for unilateral disc swelling was made in 81%, most commonly anterior ischemic optic neuropathy (n = 67, 26%) and optic neuritis (n = 12%). Notably, 18 patients were diagnosed with pseudopapilloedema (9%). Neurological consultation was requested in 128 cases (48.9%), while internal medicine in 75 cases (28.6%). Hospital admission was required for 35 patients (13.4%), 16 ophthalmology and 19 in the neurology unit. Cranial MRI was performed in 74 patients (28.3%), while basic laboratory tests were obtained in 25 patients (9.5%). In visual acuity subanalysis, patients with logMAR ≥ 1 were significantly older (p < 0.001) and more frequently presented with vascular retinal changes, hypertension, diabetes, other cardiovascular risk factors (stroke, myocardial infarction), ocular symptoms, or required internal medicine consultations.

**Conclusions**: We identified a lack of clear, standardized guidelines for the management of patients with optic disc swelling. Our findings highlight the need for the development of international, evidence-based guidelines to improve the quality and consistency of patient care. In addition, interdisciplinary education and collaboration among ophthalmology, neurology, and internal medicine are essential for accurate diagnosis and optimal management. Finally, artificial intelligence–based screening programs may serve as valuable adjuncts to clinical judgment and decision-making

132. **OSPREY: A Phase 1, open-label study evaluating the safety and tolerability of the antisense oligonucleotide STK-002 in patients with *OPA1* autosomal dominant optic atrophy**

Patrick Yu-Wai-Man^1–4^, Piero Barboni^5^, Michael Larsen^6^, Lyubomyr Lytvynchuk^7^, Berthold Pemp^8^, Katarina Stingl^9,10^, Marcela Votruba^11,12^, Karen Anderson^13^, Jeff Hoger^13^, Alice Wyse Jackson^13^, Kelly Saluti^13^, Barry Ticho^13^, Steven Gross^13^

^1^John van Geest Centre for Brain Repair and MRC Mitochondrial Biology Unit, Department of Clinical Neurosciences, University of Cambridge, Cambridge, UK; ^2^Cambridge Eye Unit, Addenbrooke’s Hospital, Cambridge University Hospitals NHS Foundation Trust, Cambridge, UK; ^3^Moorfields Eye Hospital NHS Foundation Trust, London, UK; ^4^Institute of Ophthalmology, University College London, London, UK; ^5^IRCCS San Raffaele Scientific Institute, Milan, Italy; ^6^Rigshospitalet, University of Copenhagen, Copenhagen, Denmark; ^7^Department of Ophthalmology, Faculty of Medicine, Justus-Liebig-University Giessen, Giessen, Germany; ^8^Department of Ophthalmology and Optometry, Medical University of Vienna, Vienna, Austria; ^9^Department for Ophthalmology, University Eye Clinic, Eberhard Karls University of Tübingen, Tübingen, Germany; ^10^Center for Rare Eye Diseases, Eberhard Karls University of Tübingen, Tübingen, Germany; ^11^School of Optometry and Vision Sciences, Cardiff University, Cardiff, UK; ^12^Eye Clinic, University Hospital Wales and Cardiff University, Cardiff, UK; ^13^Stoke Therapeutics, Bedford, MA, USA

**Introduction**: Autosomal dominant optic atrophy (ADOA) is primarily caused by pathogenic variants of the *OPA1* gene that lead to reduced OPA1 protein expression. OPA1 protein is critical for mitochondrial integrity and function, and its deficiency results in retinal ganglion cell (RGC) degeneration, leading to progressive optic nerve atrophy and visual impairment. Currently, no approved treatments for ADOA are available. STK-002 is an investigational antisense oligonucleotide designed to upregulate OPA1 protein expression by leveraging the wild‑type copy of the *OPA1* gene to restore expression of normal OPA1 protein. Preclinically, STK-002 has been demonstrated to increase OPA1 protein *in vitro* in cultured human cells and *in vivo* in the retinas of non-human primates (NHPs) and rabbits. Intravitreal injection (IVI) of STK-002 effectively increased OPA1 in RGCs, as demonstrated by enhanced OPA1 immunofluorescence in the RGC layer of NHPs. STK-002 also improved mitochondrial respiratory function in cultured ADOA patient-derived fibroblasts. Clinical ophthalmic exams in NHPs and rabbits indicated that STK-002 was well tolerated at doses producing pharmacological effects in the retina. Altogether, these preclinical data support the clinical development of STK-002 as the first potential disease‑modifying treatment for ADOA. Here, we present the study design for OSPREY (ISRCTN41725621), a first-in-human, Phase 1, open-label, two-part study investigating the safety and tolerability of STK-002 in patients with *OPA1* ADOA.

**Methods**: Inclusion criteria for OSPREY participants include age ≥6 to <55 years, a clinical diagnosis of ADOA, presence of a heterozygous *OPA1* gene variant, and best-corrected visual acuity (BCVA) of ≥35 and ≤70 Early Treatment Diabetic Retinopathy Study (ETDRS) letters in each eye. Key exclusion criteria include extraocular phenotypic manifestations of (syndromic) ADOA (ADOA-plus), a diagnosis of Behr syndrome, and/or the presence of gain‑of‑function or compound pathogenic or likely pathogenic *OPA1* variants. In Part A of the study, patients aged ≥18 to <55 years old will be assigned to one of four cohorts, with each cohort receiving a single IVI of STK-002 to the most affected eye, following a standard 3+3 ascending dose design. Data from Part A will determine a safe dose for Part B, which will include patients ≥6 and <18 years of age, with the first patient treated being ≥12. For both Parts, follow-up is planned for 48 weeks following STK-002 administration.

**Results**: Primary endpoints from the Phase 1 OPSREY study include safety and serum exposure of STK-002. Secondary endpoints include peripapillary retinal nerve fiber layer and macular ganglion cell layer thicknesses, BCVA, contrast sensitivity, visual field, retinal electrical activity, continuous text reading acuity, and quality of life. Exploratory endpoints include analysis of retinal mitochondrial dysfunction.

**Discussion**: OSPREY is the first study to investigate STK-002 as a potential disease‑modifying treatment for patients with *OPA1* ADOA. Recruitment is currently ongoing at European sites.

**Disclosures:** Patrick Yu-Wai-Man: Consulting for Stoke Therapeutics. Piero Barboni: Clinical trial support from Stoke Therapeutics. Michael Larsen: Consulting for and clinical trial support from Stoke Therapeutics. Lyubomyr Lytvynchuk: No relevant disclosures, PI. Berthold Pemp: No relevant disclosures. Katarina Stingl: Clinical trial support from Stoke Therapeutics. Marcela Votruba: Consulting for and speaker honoraria from Stoke Therapeutics. Karen Anderson, Jeff Hoger, Alice Wyse Jackson, Kelly Saluti, Barry Ticho, and Steven Gross are employees of Stoke Therapeutics

134. **Atypical cytomegalovirus optic neuritis in an immunocompetent patient: diagnostic challenges and therapeutic response**

Radina Kirkova^1,2^

^1^Eye Clinic Zrenieto, Sofia, Bulgaria; ^2^Università Vita-Salute San Raffaele, Milan, Italy

**Background/Purpose**: Atypical optic neuritis unresponsive to corticosteroid therapy requires expanded differential diagnosis, including infectious etiologies. Multimodal imaging may support diagnosis and monitor therapeutic response. We report a case of cytomegalovirus (CMV) retrobulbar optic neuritis confirmed by cerebrospinal fluid (CSF) analysis, with structural and functional recovery documented by optical coherence tomography (OCT) and automated perimetry.

**Case Presentation**: A 41-year-old patient presented after five months of systemic corticosteroid treatment for presumed retrobulbar optic neuritis, without clinical improvement and without evidence of multiple sclerosis. Best-corrected visual acuity was 0.2. Examination revealed relative afferent pupillary defect, optic disc edema, and concentric visual field constriction.Magnetic resonance imaging demonstrated optic nerve swelling consistent with inflammatory involvement. Due to the atypical course and steroid refractoriness, extended infectious work-up was initiated. Serology was positive for cytomegalovirus, subsequently confirmed in CSF following lumbar puncture.

**Imaging Findings**: Baseline OCT demonstrated optic nerve head edema with increased peripapillary retinal nerve fiber layer (RNFL) thickness. Functional assessment with automated perimetry revealed concentric visual field constriction correlating with clinical findings.

**Management and Outcome**: High-dose oral antiviral therapy with valacyclovir (off-label regimen) was initiated. Follow-up showed marked recovery: visual acuity improved to 1.0, optic disc edema regressed significantly, and OCT demonstrated reduction of RNFL swelling toward normalization. Visual field testing revealed near-complete functional restoration.

**Discussion**: This case underscores the importance of reconsidering diagnosis in steroid-refractory optic neuritis. Infectious optic neuropathies such as CMV, although rare, can mimic demyelinating disease. CSF analysis is critical for etiological confirmation. Multimodal imaging provides objective documentation of both inflammatory damage and treatment response.

**Conclusion**: CMV infection should be considered in atypical optic neuritis unresponsive to steroids. Early infectious work-up combined with OCT and perimetry follow-up enables accurate diagnosis and monitoring, with timely antiviral therapy leading to favorable visual outcomes.

135. **Severe pain as an indicator of malignancy in patient with optic neuropathy**

Rossul Al-Bahadili^1^, Amarah Kiani^1^ and Anastasia Pilat^1,2^

^1^East Sussex NHS Healthcare Trust, UK ^2^University of Leicester, UK

**Introduction**: Unilateral optic neuropathy in older adults represents a significant diagnostic challenge due to the wide differential diagnosis and frequent overlap in clinical and radiological features between inflammatory, vascular, infectious, and neoplastic conditions.

**Case Report**: A 79-year-old Caucasian female presented with persistent headache and left retro-orbital pain. Visual acuity was reported as 6/5 in the right eye and 6/9 in the left, preserved colour vision, no relative afferent pupillary defect, a few cells in anterior chamber and normal fundus were described. Patient had treatment for possible iritis and has been discharged. Over the following weeks, she developed abrupt deterioration in vision caused by profound painful swelling of the left optic disc, followed by central retinal artery occlusion (CRAO). Serological, autoimmune, paraneoplastic investigations were normal or negative, inflammatory markers remained consistently low; infections screen showed raised level of IgG to the HSV. In several weeks’ time patient developed partial third nerve palsy (NP), raising suspicion for orbital apex syndrome. Lumbar puncture revealed normal cerebrospinal fluid (CSF), biochemistry and a matched oligoclonal IgG pattern between serum and CSF. MRI demonstrated subtle increased T2 hyperintensity of the left optic nerve without additional intracranial abnormalities. Trial of steroid and antiviral treatment did not improve the signs/symptoms. Over the next 3 months’ time patient continuously complained on excruciating left retrobulbar pain with stable clinical symptoms and no changes on repeated imaging. In 3 months’ time patient developed subjective visual decline in the right eye with severe left retrobulbar pain extending into the right side. Follow-up MRI demonstrated diffuse enlargement and oedema of the left optic nerve extending to the chiasm. FLAIR sequence showed hyperintensity with extension around the third ventricle with enlargement of the chiasmal segment of the right optic nerve. The features were consistent with high grade glioma. Given the poor prognosis and limited therapeutic benefit beyond radiotherapy, the patient elected for supportive care and passed away 14 months after the initial presentation.

**Discussion**: The early presentation, characterised by severe retrobulbar pain, disc swelling, and subtle MRI optic nerve signal change, closely resembled inflammatory optic neuritis; however, the patient’s advanced age, persistently normal inflammatory markers, and lack of response to corticosteroid therapy signalled an atypical course. Additional development of CRAO with III NP is unusual and is more commonly associated with infectious, granulomatous, or neoplastic pathology. Continuous severe pain with completely stable neuroimaging retrospectively was the indicator of malignant glioma growth along the chiasm. Literature on glioblastoma demonstrates rapid progression along the visual pathway, often beginning with subtle optic nerve hyperintensity that later evolves into diffuse chiasmal infiltration and hemispheric spread, if undetected. Early consideration of the malignancy with possible biopsy would improve recognition and clinical outcome in patients with optic neuropathy associated with severe pain.

**Disclosure**: The author(s) have no proprietary or commercial interest in any materials discussed in this abstract.

136. **Clinical outcomes following long-term treatment with idebenone in patients with Leber’s hereditary optic neuropathy**

Raluca Iorga^1^, Andreea Moraru^1^, Răzvana Munteanu-Dănulescu^1^, Ciprian Danielescu^1^

^1^“Grigore T. Popa” University of Medicine and Pharmacy, Iasi, Romania

Leber’s hereditary optic neuropathy (LHON) is a rare disease, caused by mutations in mitochondrial DNA (mtDNA).

The aim of this paper is to present our experience with the diagnosis and management of nine LHON patients harboring the three main mutations and the *DNAJC30* mutation. We aimed to report our clinical outcomes data, following long-term treatment with idebenone in clinical practice.

**Materials and Methods:** We conducted a prospective, observational study that included 9 patients diagnosed with LHON between 2018 and 2024, followed for a period of 18 months.

**Results:** Eighteen eyes were investigated and visual acuity (VA), peripapillary retinal nerve fiber layer (pRNFL), and ganglion cells layer (GCL) were assessed at baseline and at 3, 6, 9, 12, and 18 months following idebenone treatment. The mean age of patients was 29.67 ± 8.53 years. Our findings suggest that the impact of treatment varies significantly depending on the disease phase and the mtDNA genotype. In our study, a significant improvement in VA was observed at 9 months, which remained stable at 12 and 18 months. In the acute phase, VA deteriorated from 0.67 logMAR at onset to 0.97 logMAR at 3 months, followed by a slight improvement to 0.88 logMAR at 9 months. In the dynamic phase, VA showed a continuous improvement trend. In the chronic phase, VA improved, with average values decreasing progressively from 1.44 logMAR at onset, to 1.26 logMAR at 12 months. We also observed a consistent treatment benefit over time in eyes harbouring the m.11778G>A mutation. Although the most powerful predictor of visual outcome remains the mtDNA genotype, young age at onset is correlated with a better chance of visual recovery. The impact of treatment varies significantly depending on the disease phase. Thus, in the acute phase, more cases of clinically relevant benefit (CRB) were observed than expected (33.33% versus 22.22% expected) and fewer clinically relevant worsening (CRW) cases (0% versus 11.11% expected). In the chronic phase, fewer CRB cases were observed (27.78% versus 37.05% expected) while CRW cases were more frequent (27.78% versus 18.5% expected).

Regarding OCT measurement, our study highlighted a significant difference in pRNFL thickness between the initial evaluation and at 6 months, with the mean value decreasing from 100.83 µm ± 30.2 at baseline to 96.7 µm ± 24.8 at 6 months.

**Conclusions:** LHON is a complex condition that demands ongoing research to develop effective therapies to prevent vision loss. Our paper demonstrates the benefit of idebenone treatment in improving VA in patients with LHON, highlighting the importance of extending treatment duration.

137. **A unified structural and functional framework for visualizing and quantifying the spatial pattern of retinal neuron loss and tracking progression in optic neuropathies**

Randy Kardon^1^, Mona Garvin^2^, Jui Kai Wang^3^, Edward Linton^1^, Young Kwon^1^

^1^Department of Ophthalmology (Neuro-ophthalmology), University of Iowa Carver College of Medicine and Veterans, Iowa City, IA, USA; ^2^Department of Electrical Engineering and Computer Science, University of Iowa College of Engineering and Veterans, Iowa City, IA, USA; ^3^Department of Ophthalmology, University of Texas SW, Dallas, TX, USA

**Introduction**: To develop a device-agnostic, deep learning-based framework that quantifies and visualizes spatial patterns of retinal ganglion cell (RGC) loss and functional visual field deficits across diverse optic neuropathies using Variational Autoencoders (VAEs) with AI based Deep Learning.

**Methods**: We trained VAEs on large-scale datasets of macular (GC-IPL) and optic nerve head (RNFL) OCT thickness maps, as well as Humphrey Visual Field (HVF) total deviation plots. The model compresses complex high-dimensional data into a low-dimensional latent space described by two main variables. We are integrating these findings using a mathematical anatomical model to derive individualized en face schematics of RGC somas and their axonal trajectories of each eye’s current state. This unified model was applied to patients with glaucoma, optic neuritis, and NAION to map disease-specific clusters visualized in latent space and progression trajectories.

**Results**: The VAE successfully decomposed retinal and functional damage into a continuous latent space, where different optic neuropathies occupied distinct, partially overlapping regions based on their characteristic spatial signatures. By projecting structural data onto an axonal trajectory model, we aim to generate schematic en face views that precisely depict the specific axon bundle loss radiating from the optic nerve head. This approach allowed for the quantification of damage severity and spatial extent, independent of the OCT or perimetry manufacturer. Longitudinal analysis demonstrated that disease progression could be tracked as a continuous movement within the latent space, reflecting damage to additional axon bundles over time.

**Discussion**: Our Deep Learning VAE-driven latent space framework provides a powerful, standardized method to provide a “common final depiction” of an eye’s status and can also be used to Federate data across sites. By distilling multi-modal data into a biologically grounded schematic, we can visualize where an individual eye sits within the spectrum of known pathology. This agnostic approach offers a robust clinical tool for objective diagnosis, precise quantification of neural loss, and highly sensitive monitoring of treatment efficacy in neuro-ophthalmology.

**Disclosures**: None.

138. **Rarefaction of the deep retinal vascular plexus associated with reduced visual acuity in Fabry disease**

Rebecca Wicklein^1^^*^ ^*^Shared first author, Claudia Regenbogen^2*^, Timon Kuchler^2^, Elisabeth Wolf^1^, Johanna Niederschweiberer^1^, Bernhard Hemmer^1,3^, Christoph Schmaderer^2^, Marcus Deschauer^1^, Benjamin Knier^1,4^

^1^Department of Neurology, TUM University Hospital, Universitätsklinikum, School of Medicine, Technical University Munich, Munich, Germany; ^2^Department of Nephrology, TUM University Hospital, Universitätsklinikum, School of Medicine, Technical University Munich, Munich, Germany; ^3^Munich Cluster of Systems Neurology (SyNergy), Munich; ^4^Department of Neurology and Geriatric Neurology, Diakoneo Diak Klinikum Schwäbisch Hall, Schwäbisch Hall, Germany

**Background:** Fabry disease is a rare genetic disorder characterized by the accumulation of globotriaosylceramide in the vascular endothelium, leading to multi-organ involvement. In the retina, optical coherence tomography (OCT) and OCT angiography (OCTA) have demonstrated an increased number of hyperreflective foci (HRF) — potentially reflecting endothelial glycosphingolipid deposits — along with increased retinal vessel tortuosity.

**Objectives:** To assess retinal structural and vascular alterations in patients with Fabry disease and to correlate these findings with visual function and potential paraclinical serum markers, including neurofilament light chain (NfL) and glial fibrillary acidic protein (GFAP).

**Methods:** In this cross-sectional study, 20 patients with Fabry disease were compared with 24 age- and sex-matched healthy controls (HC). Pathogenic (P) variants and variants of uncertain significance (VUS) were identified in 13 and 7 patients, respectively. All participants underwent OCT, OCTA, visual acuity testing, and serum GFAP and NfL measurements. Data are reported as medians with interquartile ranges.

**Results:** Compared with healthy controls, patients with pathogenic variants showed a significant reduction in high-contrast visual acuity (HCVA) (P: 0.5 [0.5, 0.9], VUS: 1.0 [0.9, 1.1], HC: 1.0 [0.8, 1.3]; p = 0.007) and a trend toward higher NfL levels (P: 18 pg/mL [9, 57], VUS: 8.4 pg/mL [8.0, 11.5], HC: 10.9 pg/mL [8.9, 13.2]; p = 0.07). Retinal vessel density was significantly reduced in patients with pathogenic variants, particularly in the deep vascular complex (DVC) (P: 43.7% vessel density [43.2, 45.9], VUS: 46.4% [45.7, 48.0], HC: 45.7% [45.1, 47.1]; p = 0.006), accompanied by atrophy of the inner nuclear layer (INL) (P: 31.5 µm [28.5, 34.2], VUS: 34.1 µm [33.3, 35.5], HC: 34.5 µm [32.8, 36.6]; p = 0.03). This reduction in vessel density primarily resulted from rarefaction of the smallest vessels with diameters <10 µm (p = 0.004). Particularly in the DVC, loss of these smallest vessels correlated with worse visual acuity. In addition, patients with pathogenic variants exhibited an increased number of HRF in the INL (foveal HRF: P: 8.5 [6.3, 10.3], VUS: 7.0 [3.5, 9.0], HC: 5.5 [4.3, 7.3]; p = 0.01), which correlated with DVC reduction. No correlations were found with NfL or GFAP.

**Conclusions:** Loss of the smallest vessels in the DVC is associated with reduced visual acuity and an increased number of HRF in patients with Fabry disease carrying pathogenic variants, while no signficant changes were detected in patients with VUS. Given its ease of use, OCTA may serve as a non-invasive monitoring tool for assessing small-vessel involvement in Fabry disease. Future studies should further investigate the relationship between retinal vascular changes and non-ocular neurological findings as well as MRI imaging and electrophysiology.

**Conflicts of interest**: RW received a poster award from Novartis. CR received honoraria for lectures from Amicus, Sanofi, and Takeda. EW, TK, and CS report no conflicts of interest relevant to the study.. MD received honoraria for lectures/consulting from AstraZeneca, Biogen, Roche, and Sanofi. BH served on scientific advisory boards for Novartis, Hoffmann LaRoche, Amgen and Polpharma; he has served as DMSC member for AllergyCare, Sandoz, Polpharma, Biocon and TG therapeutics; his institution received research grants from Hoffmann LaRoche and Polpharma for multiple sclerosis research. He has received honoraria for counseling (Gerson Lehrmann Group, AlphaSights Ltd).

**Funding:** European Union’s Horizon 2020 Research and Innovation Program [WISDOM EU RIA 101137154], the Deutsche Forschungsgemeinschaft (DFG, German Research Foundation) under Germany’s Excellence Strategy within the framework of the Munich Cluster for Systems Neurology [EXC 2145 SyNergy – ID 390857198] and the Clinspect-M consortium funded by the Bundesministerium für Bildung und Forschung. BK received research support from Novartis Deutschland GmbH; travel expense reimbursements from Novartis Deutschland GmbH and Merck Deutschland GmbH; lecture honoraria from CSL Behring, Heidelberg Engineering, HEXAL AG, Merck Deutschland GmbH, Novartis Deutschland GmbH, and TEVA GmbH; and consulting honoraria from Alexion, Merck Deutschland GmbH, Novartis Deutschland GmbH, and Siemens Healthineers.

**Financial Disclosures**: RW received a research fellowship from TUM University Hospital as well as funding from the German Research Foundation (DFG) within the Munich Cluster for Systems Neurology (EXC 2145 SyNergy – ID 390857198). BK received research support from Novartis Deutschland GmbH and is currently supported by the German Research Foundation (DFG 528297171).

139. **The effect of disease-modifying therapy on retinal thinning in RRMS**

Sanela Sanja Burgic^1^, Mirko Resan^2^, Zoran Vukojevic^3^, Daliborka Tadic^3^

^1^Eye Clinic, University Clinical Center of Republic of Srpska, Banja Luka, Bosnia and Herzegovina; Faculty of Medicine, University of Banja Luka, Banja Luka, Bosnia and Herzegovina; ^2^Eye Clinic, Military Medical Academy, Belgrade, Serbia; Medical Faculty of Military Medical Academy, Belgrade, Serbia; ^3^Neurology Clinic, University Clinical Center of Republic of Srpska, Banja Luka, Bosnia and Herzegovina; Faculty of Medicine, University of Banja Luka, Banja Luka, Bosnia and Herzegovina

**Introduction**: Retinal thinning is increasingly recognized as a structural biomarker of neurodegeneration in multiple sclerosis (MS), reflecting axonal and neuronal loss within the central nervous system. Optical coherence tomography (OCT) provides a non-invasive, quantitative assessment of retinal layers, particularly the retinal nerve fiber layer (RNFL) and the ganglion cell–inner plexiform layer (GCIPL), which are affected early in the disease course. Although disease-modifying therapies (DMTs) effectively reduce inflammatory activity and relapse rates in relapsing–remitting multiple sclerosis (RRMS), their impact on retinal neurodegeneration remains incompletely understood. This study aimed to evaluate longitudinal changes in RNFL and GCIPL thickness in RRMS patients with and without DMT and to examine associations between retinal thinning and clinical disability.

**Methods**: This prospective, single-center study included 40 patients with RRMS (80 eyes) followed for 12 months. Participants were divided into a no-DMT group (n = 18) and a DMT group (n = 22). The DMT group received glatiramer acetate, interferon beta-1a, interferon beta-1b, dimethyl fumarate, fingolimod, ofatumumab, or ocrelizumab. Spectral-domain OCT was performed at baseline, 6 months, and 12 months to measure average RNFL and GCIPL thickness. Neurological disability was assessed using the Expanded Disability Status Scale (EDSS). Statistical analyses included parametric and non-parametric comparisons, longitudinal analyses, Spearman correlation, and linear regression models.

**Results**: Patients not receiving DMT demonstrated significantly greater retinal thinning over 12 months compared with treated patients (p < 0.05). Mean RNFL thinning was 4.03 ± 4.87 µm in the no-DMT group versus 0.69 ± 3.91 µm in the DMT group, while GCIPL thinning was 3.43 ± 3.46 µm versus 2.25 ± 2.44 µm, respectively. From baseline to 6 months, RNFL thinning was 1.31 ± 2.34 µm in untreated patients compared with 0.16 ± 1.71 µm in treated patients, and GCIPL thinning was 1.77 ± 2.18 µm versus 0.81 ± 1.72 µm. Between 6 and 12 months, RNFL thinning remained higher in the no-DMT group (2.72 ± 2.53 µm vs. 0.53 ± 2.20 µm). RNFL and GCIPL thickness correlated negatively with EDSS scores (ρ = −0.42 and −0.38, respectively; p < 0.05). Retinal thickness significantly predicted disability progression (R^2^ = 0.36).

**Discussion**: The reduced rate of RNFL and GCIPL thinning in DMT-treated patients suggests that immunomodulatory therapy may attenuate retinal neuroaxonal loss in RRMS. Greater differences observed in RNFL than GCIPL thinning may indicate higher sensitivity of RNFL to ongoing inflammatory activity, while GCIPL loss may reflect more cumulative neurodegeneration. Persistent retinal thinning in untreated patients across both follow-up intervals supports the presence of continuous neurodegenerative processes even in the absence of clinical relapses. The association between retinal thickness and EDSS underscores the clinical relevance of OCT-derived measures as structural correlates of disability progression and supports their complementary role in disease monitoring.

**Disclosures**: None.

140. **Asian Optic Neuritis Study (AONS)–Preliminary results**

Reuben Foo^1^

^1^Singapore National Eye Centre, Singapore

**Purpose**: Optic neuritis (ON) is a major cause of visual morbidity in working-age adults. Most existing evidence, including the Optic Neuritis Treatment Trial, is derived from predominantly Caucasian populations, with minimal Asian representation. Emerging data suggest that ON in Asian patients differs in presentation, aetiology, and treatment response. This prospective, multi-centre study aims to characterise the epidemiology, clinical features, investigation patterns, and treatment outcomes of ON across a multi-ethnic Asian population in Singapore.

**Methods**: Adults aged ≥13 years with a first episode of ON were prospectively recruited from tertiary centres in Singapore. Diagnosis was confirmed clinically by a neuro-ophthalmologist and radiologically by optic nerve enhancement on Magnetic Resonance Imaging (MRI) of the anterior visual pathway and brain. Standardised data collection included demographics, symptoms, examination findings, MRI, Aquaporin4 (AQP4) and Myelin-Oligodendrocyte Glycoprotein (MOG) serology, infectious and autoimmune markers, and 12-month assessments of visual acuity, colour vision, visual fields, and Optical Coherence Tomography (OCT) Retinal Nerve Fibre Layer (RNFL) and Ganglion Cell Inner Plexiform Layer (GCIPL) thickness. Relapses within five years are recorded. All non-infectious cases received intravenous methylprednisolone followed by oral steroids; immunosuppressants or plasmapheresis were used when indicated.

**Results**: We included 28 patients (33 eyes). Mean age was 43.3 years; 68% were female. Ethnic distribution was Chinese (53.6%), Malay (25%), Indian (10.7%), and other Asian groups (10.7%). Ocular pain occurred in 53.6%, optic disc swelling in 50%, and bilateral involvement in 17.9%. Presenting visual acuity averaged 1.24 LogMAR; HVF MD -20.8 dB; RNFL 148.3 μm. MRI showed optic nerve enhancement in all cases, with chiasmal involvement in 21.4%. Final diagnoses were idiopathic ON (53.6%), MOG associated disease (MOGAD) (21.4%), Neuromyelitis optica spectrum disease (NMOSD) (10.7%), Multiple sclerosis (MS) ON (10.7%), and infectious ON (3.6%). Seven patients underwent plasma exchange and 25% required steroid-sparing immunosuppression. Among the 17 patients with 12-month follow-up, vision improved to 0.16 LogMAR, with the greatest improvement within the first month.

**Conclusions**: Preliminary findings confirm important differences in ON among Asian patients, including higher rates of disc swelling, bilateral disease, and non-MS aetiologies. As recruitment continues, this study will provide the largest prospective dataset of Asian ON to date, enabling improved subtype classification, prognostic modelling, and region-specific treatment strategies.

141. **Transient visual obscurations as the first sign of isolated leukemic optic nerve infiltration**

Ruth Martin Pujol^1^, Laura Vigués Jorba^1^, Laura Sánchez Vela^1^

^1^Ophthalmology department, Hospital Universitari Vall d’Hebron, Barcelona, Spain

**Introduction**: Infiltrative optic neuropathy is an uncommon but serious manifestation of hematologic malignancies and most commonly presents with progressive visual loss, while ocular pain is less frequently reported. Isolated optic nerve involvement as the sole manifestation of central nervous system relapse is rare and may pose a diagnostic challenge, particularly when visual acuity is initially preserved. We report an unusual case of leukemic optic nerve infiltration presenting with transient visual obscurations and ocular pain despite preserved visual function.

**Case Report**: A 33-year-old man presented to the emergency department with a one-week history of recurrent transient visual obscurations in his left eye, lasting seconds to minutes and occurring one to two times daily, associated with ocular pain that was more pronounced with eye movements. His medical history was notable for acute lymphoblastic leukemia, diagnosed four months earlier and currently in remission after systemic chemotherapy, with planned hematopoietic stem cell transplantation.

On examination, best-corrected visual acuity was 20/20 in both eyes, with preserved color vision on Ishihara testing. A mild relative afferent pupillary defect was noted in the left eye. Visual field testing showed an enlarged blind spot in the left eye. Fundoscopic examination revealed 360-degree optic disc edema with multiple peripapillary hemorrhages and exudates in the left eye, while the right eye was unremarkable. Magnetic resonance imaging demonstrated thickening and marked enhancement of the left optic nerve, suggestive of infiltrative optic neuropathy.

Given the suspicion of central nervous system relapse, a lumbar puncture was performed, confirming the presence of malignant cells in the cerebrospinal fluid. Treatment with systemic corticosteroids, intrathecal chemotherapy, and radiotherapy was initiated, resulting in marked improvement of optic disc edema within two weeks and near-complete resolution one month after diagnosis.

**Discussion**: This case illustrates that leukemic relapse may present as isolated optic nerve infiltration, without other central nervous system involvement. The patient’s preserved visual acuity, transient visual obscurations, and ocular pain represent an atypical presentation, differing from the more commonly reported progressive, painless visual loss in optic nerve infiltration. Such unusual manifestations can pose a diagnostic challenge, emphasizing the need for high clinical suspicion and prompt neuro-ophthalmologic evaluation in patients with a history of hematologic malignancy. Early recognition and timely treatment of infiltrative optic neuropathy can lead to favorable anatomical and functional outcomes.

**Disclosures:** None.

146. **Clinical-genetic analysis in Bulgarian patients with Leber’s Hereditary Optic Neuropathy**

S. Cherninkova^1^, B. Zaharova^2^, K. Kamenarova^3,4^, K. Mihova,^3,4^, S. Atemin^5^, T. Todorov^5^, V. Haikin^6^, A. Oscar^6^, I. Tournev^1,7,8^, R. Kaneva^3,4^, A. Todorova^4,5^

^1^Clinic of Neurological diseases, University Aleksandrovska Hospital, Sofia, Bulgaria; ^2^National Genetic Laboratory, Medical University, Sofia, Bulgaria, University Hospital of Obstetrics and Gynecology “Maichin Dom”, Sofia, Bulgaria; ^3^Genome Diagnostics Laboratory, Molecular medicine centre, Medical University, Sofia, Bulgaria; ^4^Department of medical chemistry and biochemistry, Medical University, Sofia, Bulgaria; ^5^Genetic Medico-Diagnostic Laboratory “Genica”, Sofia, Bulgaria; ^6^Clinic of Eye diseases, Department of Ophthalmology, Medical University, Sofia, Bulgaria; ^7^Department of Neurology, Medical University, Sofia, Bulgaria; ^8^Department of Cognitive Science and Psychology, New Bulgarian University, Sofia, Bulgaria

**Introduction**: Leber’s hereditary optic neuropathy (LHON) is a rare maternally inherited disease caused by mitochondrial DNA (mtDNA) point mutations in genes encoding the MT-ND1, MT-ND4, MT-ND4L and MT-ND6 subunits of complex I in the mitochondrial respiratory chain. The most common primary mutations G11778A in MT-ND4 gene, G3460A in MT-ND1 gene and T14484C in MT-ND6 gene cause the disease in 90% of patients but there are also a number of less common mutations. LHON is characterized by bilateral acute or subacute visual failure and usually occurs in young males.

**Purpose**: An assessment of the clinical symptomatology and genetic analysis in Bulgarian patients with LHON.

**Materials and methods**: On the basis of the clinical evaluation and genetic investigation 26 patients - 20 males and 6 females were diagnosed with LHON. A full neuroophthalmologic examination including visual acuity testing, Goldmann kinetic or automated static threshold perimetry, slit lamp biomicroscopy, direct ophthalmoscopy, optical coherence tomography, evaluation of ocular motility and genetic investigation was performed in the patients.

**Results and discussion**: Ten patients from 2 unrelated families had family history of LHON and the remaining 16 patients were isolated (sporadic) cases in their families. The age at onset of visual failure ranged from 3 to 43 years (average=18,9 years). Visual acuity ranged between counting fingers and 0,9 in the patients. Visual field examination revealed the most common finding of bilateral central scotoma. In 4 patients, examined during the acute stage of optic neuropathy, ophthalmoscopy revealed an optic disc hyperemia and retinal vessels tortuosity and in the remaining 22 patients examined after the acute stage bilateral optic atrophy was found. The genetic testing revealed the following gene mutations responsible for the disease: G11778A, MT-ND4 gene in 11 patients; G3460A, MT-ND1 gene in 3 patients; G3635A, MT-ND1 gene in a family with 5 patients; G11778A, MT-ND4 gene and T14484C, MT-ND6 gene in a family with 5 patients and one asymptomatic patient; a variant A15988G, MT-TP gene in one patient and С4640А, MT-ND2 in one patient.

**Conclusion**: Except the most common mutations G11778A in MT-ND4 gene, G3460A in MT-ND1 gene, T14484C in MT-ND6 gene in our patients, a rare mutation G3635A in MT-ND1 was found in 5 affected members of one pedigree. Interestingly a digenic inheritance of G11778A and T14484C was detected in another 5 individuals from a different family with one asymptomatic carrier of T14484C (without being able to exclude low-grade heteroplasmy of G11778A). A variant m.15988A>G in the mitochondrial gene MT-TP with high level of heteroplasmy was found in one patient as well as another patient with a rare mutation - С4640А in MT-ND2 gene.These results indicate that a genetic analysis of the whole mtDNA sequence is recommended in the cases of a strong clinical suspicion of LHON since new rare mtDNA pathogenic variants are increasingly identified.

147. **Arteritic anterior ischaemic optic neuropathy (AAION) without disc swelling and normal inflammatory markers**

Samantha Siaw Zhen Sii^1^, Shery Thomas^1^, Rupa Patel^1^

^1^Nottingham University Hospitals Trust, UK

**Introduction**: This was a rare case of AAION without optic disc swelling, and normal inflammatory markers.

**Case report**: A 79 year old gentleman attended eye casualty. He mentioned that his vision had ‘sparkles’ in it for 2 weeks. He had no headache, no fatigue, loss of weight, night sweats or polymyalgic symptoms. He did have bilateral cheek pain for 4 weeks that he had seen his dentist for.

On examination, his right eye vision was 0.40 LogMAR, Ishihara colour vision 3/17, a right relative afferent pupillary defect and a pale optic disc. His C- reactive protein was normal at 7mg/L. The patient was initially sent home and advised to return for a neuro-ophthalmologist assessment within 2 days. During this assessment, it was noted that the retinal nerve fibre layer (RNFL) thickness was within the normal range, no disc swelling could be seen but the disc was pale. It was determined that the disc was swollen, on a background of optic atrophy. An urgent temporal artery ultrasound scan was positive for Giant Cell Arteritis and he was treated accordingly. Six weeks later, RNFL thinning was seen.

Fundus photos from 4 months prior were obtained from the local optician demonstrating the pre-existing optic atrophy. The cause for the optic atrophy was not found.

**Discussion**: This case demonstrates the importance of not relying on ‘green’ or ‘red’ disease based on optical coherence tomography RNFL analysis but interpreting each case with caution as even ‘green’ or normal RNFL can indicate disc swelling in the setting of pre-existing optic atrophy.

**Disclosure**: The patient provided informed consent for the use of anonymised clinical information and imaging for educational purposes. No identifiable patient data are included. The authors declare no conflicts of interest and received no financial support for this work.

151. **High-dose glucocorticosteroids and neuroprotection in acute optic neuritis: A multicenter retrospective cohort study**

Sebastian Küchlin^1^, Jörg Sahlmann^2^, Wolf Lagrèze^1^

^1^Eye Center, Medical Center – University of Freiburg, Faculty of Medicine, University of Freiburg, Germany; ^2^Institute of Medical Biometry and Statistics – University of Freiburg, Faculty of Medicine, University of Freiburg, Germany

**Introduction**: Multiple sclerosis-associated optic neuritis (ON) is the most common optic neuropathy in young adults. It leads to retinal ganglion cell loss as the substrate of only partially reversible vision loss. High-Dose glucocorticosteroids (GCS) remain the only widely used first-line therapy. In the Optic Neuritis Treatment Trial (ONTT), published in 1992, high-dose GCS accelerated visual recovery without improving long-term visual acuity. Spectral domain optical coherence tomography (OCT), then unavailable, now enables micrometer-resolution quantification of neuroaxonal loss. This raises the possibility that a modern study could detect previously unseen neuroprotection.

**Methods**: This international, multi-centric, retrospective cohort included patients aged 18–60 years with a first episode of unilateral multiple sclerosis-associated or single isolated ON, who had been managed with either high-dose GCS or observation alone, and had OCT follow-up between 45 days and 2 years after symptom onset. The primary endpoint was ganglion cell loss in the affected eye, assessed as the change in macular ganglion cell and inner plexiform layer thickness (mGCIPLT). Secondary endpoints included the change in peripapillary retinal nerve fiber layer thickness (pRNFLT), high contrast visual acuity (HCVA), and low contrast letter acuity (LCLA). Analysis was by treatment group via linear regression with predefined adjustment for sex, center, and baseline HCVA.

**Results**: We analyzed 431 patients from 15 centers in 8 countries. 331 had received high-dose GCS and 100 were untreated. At baseline, GCS-treated patients had poorer HCVA (median [IQR]: logMAR 0.49 [0.15–1.3] vs. 0.20 [0.00–0.50], *P*<.001). In the GCS group, the median time to treatment was 7 days (4–11), and the median dose was 3 g (3–5). Age and sex were balanced between groups. At follow-up, male sex was independently associated with an additional 4.34 µm loss (1.03–7.66), and each line (.1 logMAR) of baseline HCVA deficit was associated with an additional 0.82 µm loss (0.60–1.03). There was no significant difference between GCS and observation (adjusted difference: 2.73 µm less mGCIPL atrophy in favor of GCS; 95% CI: -1.50–6.97). Thinning of the pRNFLT, HCVA, and LCLA were also similar between groups.

**Conclusion**: Retinal ganglion cell loss was similar in patients who received high-dose GCS and patients who were managed by observation only after accounting for predictors of disease severity.

153. **Ethambutol optic neuropathy (EON): Mechanistic insights from proteomic analysis of a stem cell derived retinal ganglion cell model of EON**

Shweta Singhal^1^, Rayhan Al Awdady Bte Ismail^2^, Muhammad Ikhsan Bin Muzakar^2^, Zhou Lei^2^

^1^Singapore National Eye Centre, Singapore Eye Research Institute, Duke NUS Medical School Academic Medicine Centre, Yong Loo Lin School of Medicine, Singapore; ^2^ Singapore Eye Research Institute, Singapore

**Introduction:** Ethambutol optic neuropathy is the leading cause of all toxic optic neuropathies world-wide. Of the 10 million new cases of Tuberculosis (TB) reported by WHO each year, 6-8% develop EON which if not diagnosed and managed early, can quickly cause irreversible legal blindness. Since TB can be life threatening, from a public health perspective, the visual side effects of this drug are often accepted as collateral damage. Our intention was to understand mechanisms that lead to Ethambutol related optic neuropathy on a cellular level with the specific aim of identifying targets that might modify the disease process and prevent irreversible damage to the optic nerve despite exposure to the drug. We present our early results here.

**Methods:** Using known protocols, Human embryonic stem cells (HES) in vitro were differentiated towards a retinal ganglion cell (RGC) fate. The HES derived RGC were exposed to increasing concentrations of EMB and cellular changes assessed to establish a disease phenotype. The minimal dose of the EMB needed to see a disease phenotype was then used to set up a directed iodo -TMT labelled proteomics analysis. This assay was then used to identify differential protein glutathionylation between control and EMB treated cells.

**Results:** Even with sub molar doses (expected concentration in vitro at standard therapeutic oral dose) HES derived RGC demonstrated a clear mitochondrial phenotype. This phenotype was confirmed by change in mitochondrial membrane potential; and maximal respiration on Seahorse mitochondrial respiration assays. A directed quantitation of glutathinonylated proteins revealed that EMB exposure in HES derived RGC resulted in differential glutathionylation of key proteins involved in cGMP-PKG signalling pathways and mRNA metabolic pathways among others.

**Discussion:** HES derived RGC offer an excellent model to study the cellular mechanisms of optic neuropathies. Based on our data we can confidently conclude that EON is a mitochondrial neuropathy and EMB exposure disrupts key mitochondrial pathways in HES derived RGC. These are early results and the pathways identified here need to be evaluated in further detail to establish if they can be targeted in a meaningful way to protect optic nerve health; and prevent irreversibly neuropathy in patients who need ethambutol based treatments.

**Disclosures:** The first author and this work was supported by grant funding from the National Medical Research Council of Singapore.

154. **Optic nerve biopsy: When and why?**

Silvia Muñoz^1^, Eugènia Moix^1^, Maravillas Abia^1^

^1^Ophthalmology Department, Hospital Universitari de Bellvitge, L’Hospitalet de Llobregat, Spain

**Introduction:** Optic nerve or optic sheath biopsy is an invasive diagnostic procedure that may result in irreversible blindness. Advances in neuroimaging techniques have increased the diagnostic yield in optic neuropathies, so that biopsy is rarely needed.

**Aim:** To review the clinical presentation and to determine the agreement rate between pre-biopsy diagnosis and final pathologic findings.

**Method:** Retrospective study of clinical cases during a 10-year span.

**Results:** Four Caucasian women underwent unilateral optic nerve biopsy within 2015 and 2024. Their median age at the procedure was 44,7 years (range from 25 to 59), which was performed within 2 to 144 months (median 62 months) There were three gliomas and one meningioma. The agreement rate was one out of four (25%). Below there is a brief description of the cases.

Firstly, a 59-year-old (yo) lady received fractionated stereotactic radiotherapy for presumed optic nerve sheath meningioma elsewhere. Six years later, MR was reviewed and the suspicion of glioma arose. Apart from ischemic radiotherapy retinopathy, there were concerns of malignant transformation. The biopsy confirmed glioma and ruled out malignancy. Visual acuity had been null since her twenties.

Secondly, a 38 yo woman with acute visual loss attributed to optic neuritis, improved after receiving IV corticosteroids. MR proved thickened optic nerve and partial enhancement of its sheath; no calcification appeared in orbit CT. Either granuloma or meningioma were suspected initially. After 5 years of sustained visual function, she experienced worsening. Then, a follow-up MR suggested glioma. She declined biopsy until visual acuity was hand motion, 12 years after the onset. It proved meningioma.

Thirdly, a 25 yo patient experienced sudden visual loss, pain, proptosis and mild optic disc swelling. Neuroimaging showed optic nerve fusiform enlargement suggestive of glioma. Afterwards it followed a relapsing-remitting visual loss pattern from the worst to nearly normal. Thirty months later the tumor was removed because of painful prominent proptosis and definite vision deterioration. Pathology confirmed the initial diagnosis of glioma.

Lastly, a 36 yo woman developed abrupt visual loss leading to amaurosis in 10 days. There were multiple and diffuse retinal hemorrhages and prominent optic disc swelling. Due to extreme obesity (IMC >60) and poor vein access, only non-contrast MR was obtained. The enlargement of the optic nerve, the rapid onset, and the concerns of hematologic malignancy or glioblastoma led to biopsy obtained 2 months later. The diagnosis was pilocytic astrocytoma.

**Discussion:** When clinical or radiological picture was misleading or incomplete, biopsy had a role to clarify the diagnosis in three out of four cases. As irreversible blindness is a potential unwanted side-effect, patients are understandably reluctant to accept this procedure.

**Conclusion:** Although extremely unusual, complicated cases may require pathological investigation.

**Disclosures:** Authors declare no interest conflicts and deny the use of IA in the abstract writing.

155. **Non-invasive biomarkers for the differential diagnosis of acute anterior ischemic optic neuropathy in an emergency setting**

Stefano Erba^1^, Laura Fumagalli^1^, Enrico Tombetti^2^, Stefano De Angelis^1^

^1^ASST Fatebenefratelli Sacco, Ophthalmic Hospital, Milan, Italy; ^2^ASST Fatebenefratelli-Sacco, Department of Internal Medicine, Fatebenefratelli Hospital, Milan, Italy.

**Purpose**: To identify clinical, functional, structural, and laboratory biomarkers useful for the differential diagnosis between arteritic (A-AION) and non-arteritic anterior ischemic optic neuropathy (NA-AION) in the acute phase, with particular emphasis on non-invasive parameters applicable in an ophthalmic emergency setting.

**Methods**: This retrospective study included patients presenting with acute AION (symptom onset <15 days) at the Ophthalmic Emergency Department of Milan between January 2018 and April 2025. Inclusion criteria were age >50 years and confirmed diagnosis of A-AION or NA-AION. All patients underwent comprehensive ophthalmologic examination, best-corrected visual acuity (BCVA), Humphrey visual field testing 24-2 SITA-Fast (HVF), and spectral-domain Optical Coherence Tomography (SD-OCT) Retinal Nerve Fiber Layer thickness (RNFL-T) and Bruch’s membrane opening (BMO). Peripapillary choroidal thickness (PCT) was manually measured on SD-OCT images. Laboratory parameters included Erythrocyte Sedimentation Rate (ESR), C Reactive Protein, fibrinogen, and complete blood count. An age-matched healthy control group was included for comparison of PCT. Logistic regression models were developed to discriminate A-AION from NA-AION using either a full set (clinical, laboratory, and imaging) or a partial set predictor model (age, BCVA, visual field MD, RNFL-T).

**Results**: Sixty-eight eyes from 61 patients were analyzed (45 NA-AION, 23 A-AION). Patients with A-AION were significantly older than those with NA-AION (mean 78.9 vs 64.9 years, p<0.01). A-AION showed significantly higher inflammatory markers (ESR, CRP, fibrinogen, platelet count) and lower hemoglobin levels (all p<0.01). Visual function was significantly worse in A-AION, with lower BCVA and more severe visual field loss (p<0.01). RNFL thickness and Bruch’s membrane opening did not differ between groups, while mean peripapillary choroidal thickness was significantly reduced in A-AION compared to NA-AION and controls. Peripapillary choroidal thickness negatively correlated with age (r = −0.38, p<0.01). The full set logistic regression model achieved an AUC of 0.90, while the partial set predictor model showed comparable performance (AUC 0.88).

**Conclusions**: A-AION is associated with older age, severe visual impairment, and a marked inflammatory profile. A partial set predictor model based on routinely available clinical and OCT parameters provides high accuracy in distinguishing A-AION from NA-AION in the acute phase and may support rapid clinical decision-making in emergency neuro-ophthalmology.

**Disclosures:** None.

156. **Optic nerve and retinal ganglion cell changes following semaglutide exposure in healthy mice**

Talal Salti^1^, Katia Marzuq^1^, Nitza Goldenberg-Cohen^1,2^

^1^Bruce and Ruth Rappaport Faculty of Medicine, Technion – Israel Institute of Technology, Haifa, Israel; ^2^Department of Ophthalmology, Bnai Zion Medical Center, Haifa, Israel

**Purpose**: Glucagon-like peptide-1 receptor agonists (GLP-1 RAs), including semaglutide, are widely used for metabolic disorders. Recent clinical observations have suggested possible neuro-ophthalmic adverse events; however, their direct effect on the healthy retina or optic nerves is unknown. We evaluated retinal ganglion cell (RGC) and optic nerve integrity after semaglutide administration in healthy mice.

**Methods**: Twenty healthy male C57BL/6 mice (8–10 weeks) received weekly subcutaneous semaglutide (0.25–1 mg/kg) for 4 weeks. Twenty age-matched untreated mice served as controls. After euthanasia on week 5, eyes were sectioned at 20-µm intervals and stained with DAPI. RGC nuclei were quantified at ×20 magnification using systematic sampling (three fields per section, three sections per slide, every fifth slide). Optic nerves underwent immunohistochemical staining for glial and microglial activation markers (GFAP, IBA1).

**Results**: Two of 40 eyes in the semaglutide-treated group demonstrated focal RGC loss accompanied by optic nerve changes on GFAP and IBA1 staining. No RGC loss or optic nerve abnormalities were identified in the 40 control eyes.

**Conclusions**: Semaglutide exposure in healthy mice was associated with RGC loss and optic nerve alterations in a small subset of eyes. The limited sample size and dosing conditions prevent causal inference. Further studies are required to determine whether GLP-1 receptor agonists contribute to optic neuropathy and to clarify underlying mechanisms.

157. **Peripapillary Hyperreflective Ovoid Mass Like Structure (PHOMS): A non-specific OCT marker in Papilledema, Pseudo Disc Edema and Disc Edema**

Tapi Yalu^1^, Swati Aalok Phuljhele^1^, Rohit Saxena^1^, Rebika Dhiman^1^

^1^Ophthalmology, Rajendra Prasad Centre for Ophthalmic Sciences, AIIMS New Delhi, India

**Purpose**: The aim of this study is to evaluate the presence of Peripapillary Hyperreflective Ovoid Mass Like Structure (PHOMS) in Papilledema, Pseudo Disc Edema and Disc Edema patients and correlate Optical Coherence Tomography (OCT) parameters with PHOMS.

**Methods**: This Cross-Sectional Observational study was carried out in a tertiary center in New Delhi, India following ethics approval from the Institutional Ethics Committee. 90 patients were enrolled (50 patients-Papilledema, 10 patients-Pseudo Disc Edema, 30 patients- Optic Disc Edema) and underwent Spectral Domain Optical Coherence Tomography (SD-OCT) to look for the presence of PHOMS. If PHOMS were found, its location, size, volume and associated Retinal Nerve Fibre Layer loss (if any) were assessed.

**Results**: Among the 90 eyes examined, it was found that PHOMS were present in 28% of papilledema patients, 30% of Pseudo Disc Edema patients and 23.3 % of Disc Edema patients. The most common location of PHOMS were found to be Superonasal (33.3%) followed by Nasal (25%) quadrants. The mean horizontal diameter of PHOMS was 479.92±176.06 micrometers with papilledema group demonstrating a statistically significant larger PHOMS (Kruskal-Wallis p=0.046). The mean vertical diameter of PHOMS was 424.96±165.49 micrometers. Although Disc edema group demonstrated a numerically larger vertical diameter compared to papilledema and Pseudo Disc Edema, this difference was not statistically significant (Kruskal-Wallis p= 0.676). The mean volume of PHOMS was 0.17±0.08millimeter square. However, no significant difference in volume of PHOMS was observed among three groups. No statistically significant reduction in Retinal Nerve Fibre Layer was observed.

**Discussion**: The distribution of PHOMS in this cohort aligns with the growing consensus that PHOMS are nonspecific OCT marker of axoplasmic stasis, rather than a diagnostic hallmark of a particular pathology. The current literature suggests that PHOMS are located preferentially in Superonasal quadrant, our results extend these observations by suggesting need for further studies to carry out additional information about interaction between PHOMS and local tissue architecture.

The significant increase in horizontal diameter as found in papilledema group suggests that this may be influenced by disease specific biomechanical forces acting on optic nerve head. However, the absence of significant intergroup differences in vertical diameter and volume correlates with the current studies and emphasizes the need to further explore. Retinal Nerve Fibre Layer thickness abnormalities was not found to be statistically significant indicating that Retinal Nerve Fibre Layer alterations are not consistent features of PHOMS.

**Disclosure**: The authors declare no conflicts of interest related to this study. No external funding or financial support was received for the conduct of this research.

160. **Informative censoring in natural history control cohort influences the interpretation of the long-term efficacy of idebenone in treating visual impairment from Leber hereditary optic neuropathy**

Thomas Klopstock^1-3^, Alfredo A. Sadun^4^, Nancy J. Newman^5^, Patrick Yu-Wai-Man^6-9^, Rod Gossen^10^, Daniela​​​​ Garavaglia^11^, Karen Sison Lance^10^, Caroline Fradette^10^, Xavier Llòria^11^, Valerio Carelli^12,13^

^1^Friedrich Baur Institute at the Department of Neurology, LMU University Hospital, LMU Munich, Munich, Germany; ^2^German Center for Neurodegenerative Diseases (DZNE), Munich, Germany; ^3^Munich Cluster for Systems Neurology (SyNergy), Munich, Germany; ^4^Doheny Eye Institute, Department of Ophthalmology, University of California, Los Angeles, CA, USA; ^5^Departments of Ophthalmology, Neurology, and Neurological Surgery, Emory University School of Medicine, Atlanta, GA, USA; ^6^John van Geest Centre for Brain Repair, and MRC Mitochondrial Biology Unit, Department of Clinical Neurosciences, University of Cambridge, Cambridge, UK; ^7^Cambridge Eye Unit, Addenbrooke’s Hospital, Cambridge University Hospitals, Cambridge, UK; ^8^Moorfields Eye Hospital NHS Foundation Trust, London, UK; ^9^Institute of Ophthalmology, University College London, London, UK; ^10^Chiesi Canada Corp, Woodbridge, Ontario, Canada; ^11^Chiesi Farmaceutici S.p.A, Parma, Emilia-Romagna, Italy; ^12^IRCCS Istituto di Scienze Neurologiche di Bologna, Bologna, Italy; ^13^Department of Biomedical and Neuromotor Sciences, University of Bologna, Bologna, Italy.

**Introduction**: The Phase IV, 24-month, open-label LEROS study (NCT02774005) demonstrated efficacy of idebenone (IDE) for patients with Leber hereditary optic neuropathy (LHON), a mitochondrial disease resulting in progressive, bilateral vision loss. Using alternative analysis methods, including longitudinal reallocation of visual acuity (VA) assessments, this LEROS post‑hoc analysis examined whether differences in assessment timing and follow‑up patterns between cohorts could lead to informative censoring that influences interpretation of long‑term treatment effects.

**Methods**: For NH patients, baseline was defined as the first assessment visit following diagnosis, and post-baseline assessments were allocated to analysis timepoints based on distance from baseline. This differs to previous LEROS analyses where any NH assessment visit was considered a potential baseline, and post-baseline assessments were allocated based on comparison of equal distance, regardless of patient follow-up time. Stabilized inverse probability of treatment weights derived from a propensity score model were used to adjust for baseline differences between IDE and NH cohorts. Five prognostic factors were included in the modelling as covariates: sex, age at symptom onset, time since onset at baseline, baseline best-corrected VA in the better-seeing eye (best BCVA), and mitochondrial DNA variant. Treatment response rates were expressed as patient weights rather than number of patients. Clinically relevant benefit (CRB), as defined in the original LEROS analysis, included an improvement of ≥10 EDTRS letters on-chart or improvement from off-chart to ≥5 ETDRS letters on-chart, and/or maintenance of a baseline best BCVA <1.0 logMAR.

**Results**: At Month 12, IDE-treated patients demonstrated a significantly higher rate of CRB (54% [95% confidence interval {CI}: 46%, 63%]) than the NH cohort (33% [95% CI: 23%, 42%]). However, the difference in CRB rates between the two groups reduced at Month 24 (IDE: 53% [95% CI: 43%, 62%]; NH: 48% [95% CI: 34%, 61%]). Ad-hoc analysis supported the hypothesis that this convergence was driven by informative censoring in the NH cohort. A clear trend in the NH group towards poorer mean best BCVA in patients without Month 24 data, versus those with data, indicated selective loss of patients with worse outcomes in the NH cohort. No effect of best BCVA on study withdrawals was observed with IDE treatment.

**Discussion**: This post-hoc analysis confirms that a greater proportion of IDE-treated patients experienced a CRB compared with NH patients at Month 12. At Month 24, IDE treatment maintained a clinical benefit. An ad-hoc investigation supported the hypothesis that informative censoring in the NH cohort lessened the mean difference across groups at Month 24. The purported informative censoring did not occur in the IDE-treated group, likely due to the interventional nature of the LEROS study, with planned assessments reducing the likelihood of withdrawal. Informative censoring should be considered when interpreting long-term NH comparator data in LHON.

**Disclosures**: TK and VC received research support and consultation fees from Santhera Pharmaceuticals, Chiesi, and GenSight Biologics. AS has been a consultant for Chiesi and GenSight Biologics and has received research support from GenSight Biologics and Stealth Therapeutics. NJN has been a consultant for Chiesi, GenSight, Stoke, Neurophth, and Eli Lilly. PYWM received research support and consultation fees from Neurophth, Stoke Therapeutics, Santhera Pharmaceuticals, Chiesi, and GenSight Biologics. RG, KSL, and CF are employees of Chiesi Canada Corp. DG and XL are employees of Chiesi Farmaceutici S.p.A. Medical writing support was provided by Cactus Life Sciences, and funded by Chiesi Fa.

161. **The MOGnitude of NAION**

Tim Beltraminelli^1^, Kevin Müggler^1^, Aki Kawasaki^1^

^1^Jules Gonin Eye Hospital, University of Lausanne, Lausanne, Switzerland

**Introduction:** Nonarteritic anterior ischemic optic neuropathy (NAION) and myelin oligodendrocyte glycoprotein antibody-associated disease optic neuritis (MOGAD-ON) are two distinct entities; nonetheless their occasionally-overlapping clinical presentation can lead to significant diagnostic delays, particularly in unilateral and painless cases, resulting in unfavorable visual outcomes. Because early recognition directly influences prognosis and therapeutic urgency for MOGAD-ON, distinguishing these conditions remains a critical yet challenging task in neuro-ophthalmic practice in order to orient the correct clinical management.

**Case Report:** We present the case of a 66-year old man who developed a painless, abrupt inferior hemifield visual loss in the right eye upon awakening. The patient has no previous ocular history, and system review was significant for numerous cardiovascular risk factors. Clinical examination revealed reduced acuity (0.4 Snellen equivalent), dyschromatopsia (0/13 Ishihara plates), an inferior visual field defect and a 3+ RAPD in the affected eye. Slit-lamp examination showed sectorial optic disc edema with flame-shaped hemorrhages in the superior portion of the right optic nerve. Examination of the left eye revealed a disc-at-risk, but was otherwise unremarkable. With a presumptive diagnosis of NAION the patient was re-evaluated 1 week later, at which time visual acuity had dropped to no-light perception (NLP). Given this atypical progression, the working diagnosis shifted to papillitis, prompting an extensive work-up with MRI, neurologic evaluation with lumbar puncture, temporal artery biopsy, fluorescein angiography, and blood analyses. High titers of anti-MOG antibodies were detected in both CSF and serum. All other analyses were negative. High-dose corticotherapy was initiated and patient regained partial vision.

**Discussion:** Differentiating NAION from MOGAD-ON is crucial for appropriate clinical management, treatment decisions, and patients’ visual prognosis. The clinical overlap between these two distinct entities poses a significant diagnostic challenge, and remains a pertinent issue in neuro-ophthalmology. A recent study by Tisavipat et al. showed 100% specificity in differentiating NAION from MOGAD-ON based on the presence of a crowded disc, altitudinal visual field defect, disc edema and absence of pain —the latter being a key distinguishing feature. Here, we present a case of MOGAD-ON masquerading as a “textbook” presentation of NAION but following an atypical clinical course. Unusual evolution of “classic NAION” should always raise suspicion. In this case, the dramatic decline to NLP —reported only in about 1.28% of NAION cases—prompted further investigation, ultimately revealing the correct diagnosis. Positivity for anti-MOG IgG antibodies in the serum, detected by cell-based assays, carries a specificity of 98% for MOGAD-ON. False positive rates are very rare (about 8%) and typically associated with low antibody titer. Importantly, MRI findings are not strictly required for diagnosis, based on MOGAD-diagnostic criteria, particularly in the presence of a classic clinical manifestation and MOGAD and presence of anti-MOG serum IgG.

**Disclosures**: The authors declare no financial interests.

162. **A family affair … .**

Salvatore Sottosanti

Casa di Cura La Maddalena, Palermo, Italy

In this presentation, I describe a family in which several members have been identified with bilateral optic disc atrophy. All affected individuals have genetic mutation attributable to both Dominant Optic Atrophy (c.1099C>T in heterozygous OPA1) and Leber Optic Atrophy (variant m.14484T>C/MT-ND6).

163. **Neuroprotective effects of intravitreal injections of umbilical cord Mesenchymal stem cell-derived exosomes in rats with traumatic optic neuropathy (TON)**

Rong-Kung Tsai^1,2^, Ruksana Akter Ruma^1,2^, Tu-Wen Chen^1^

^1^Institute of Eye Research, Hualien Tzu Chi Hospital, Buddhist Tzu Chi Medical Foundation, Hualien 970, Taiwan; ^2^Institute of Medical Sciences, Tzu Chi University, Hualien, Taiwan.

**Background**: Traumatic optic neuropathy (TON) causes irreversible vision loss without effective treatments. Mesenchymal stem cell (MSC)-derived exosomes, a cell-free therapeutic approach, have intrinsic regenerative and immunomodulatory properties. This study investigated the ocular safety and neuroprotective efficacy of intravitreal injections (IVI) of human umbilical cord MSC-derived exosomes (hUCMSC-Exos) in a rat model of optic nerve crush (ONC)

**Methods**: Retinal safety was assessed in Wistar rats following a single intravitreal injection of hUCMSC-Exos using optical coherence tomography (OCT), electroretinography (ERG), and histology. For efficacy experiments, rats were subjected to ONC and received an immediate intravitreal injection of either hUCMSC-Exos or PBS. Visual function and structural integrity were evaluated 14 days post-injury via FVEP, OCT, Retrograde RGC labeling, and TUNEL staining. Neuroinflammation, oxidative stress, and signaling pathways were analyzed using immunohistochemistry and Western blotting.

**Results**: Intravitreal injections of hUCMSC-Exos demonstrated a safety profile with no structural or functional retinal toxicity observed in normal eyes. In the ONC model, hUCMSC-Exos significantly preserved visual function, evidenced by a 2.6-fold increase of P1–N2 amplitudes in FVEP compared with PBS-treated group. Structurally, exosome treatment enhanced RGC survival by 2.1-fold, reduced RGC apoptosis by 2.23-fold, attenuated optic nerve head edema, and preserved retinal nerve fiber layer (RNFL) thickness as compared with the PBS-treated group. hUCMSC-Exos suppressed inflammatory responses (macrophage infiltration, glial activation, and expression of IL-1beta, IL-6, and TNF-alpha) and upregulated antioxidant proteins (Nrf2, HO-1, and SOD2) via the activation of the PI3K/AKT and ERK signaling pathways.

**Conclusions:** IVI of hUCMSC-Exos exhibit ocular biocompatibility and confer neuroprotective effects following ONC injury. The anti-inflammation, anti-apoptosis and anti-oxidant actions were through the modulation of the AKT/ERK-mediated Nrf2 pathway. These findings imply that IVI of exosomes, a cell-free therapeutic strategy, is promising for the preclinical trial of TON.

164. **Nonarteritic anterior ischemic optic neuropathy following sclerectomy for uveal effusion in nanophthalmos: A case report**

Wei-Che Hung^1*^, Hui-Chen Cheng^1,2^, An-Guor Wang^1,2^, Shih-Jen Chen^1,2^

^1^Department of Ophthalmology, Taipei Veterans General Hospital, Taipei, Taiwan; ^2^Department of Ophthalmology, School of Medicine, National Yang Ming Chiao Tung University, Taipei, Taiwan

**Introduction**: Nonarteritic anterior ischemic optic neuropathy (NAION) is an acute ischemic disorder of the optic nerve head that typically affects older individuals or those with systemic vasculopathic risk factors and is uncommon in younger patients without such comorbidities. To date, only two cases of NAION associated with uveal effusion syndrome have been reported, both with limited clinical detail. We report a third case of NAION following sclerectomy for uveal effusion syndrome in a patient with nanophthalmos, featuring comprehensive clinical investigation, including visual field assessment and longitudinal optic nerve evaluation.

**Case Report**: A 47-year-old man with no history of diabetes mellitus, hypertension, or cardiovascular disease presented with a two-month history of progressive blurred and distorted vision in the left eye. He had previously been treated elsewhere for presumed Vogt–Koyanagi–Harada disease with intravenous methylprednisolone without improvement. Family history was notable for an older brother with nanophthalmos and uveal effusion managed surgically.

Best-corrected visual acuity was 20/20 in the right eye and finger counting in the left eye. Refractive error was +7.00 diopters in the right eye and +21.00 diopters in the left eye. Axial lengths measured 19.55 mm and 20.02 mm in the right and left eyes, respectively. Fundus examination revealed a bullous inferior serous retinal detachment in the left eye. Because the detachment persisted, a 1 × 2 mm lamellar sclerectomy was performed over the inferomedial vortex vein. Three weeks postoperatively, subretinal fluid decreased modestly and visual acuity improved to 20/100.

Six weeks after surgery, the patient developed new inferior visual field loss in the left eye, with visual acuity declining to hand motions. Color vision was markedly reduced in the left eye, and a relative afferent pupillary defect was present. Fundus examination showed optic disc edema. Automated perimetry demonstrated a dense inferior altitudinal scotoma. Fluorescein angiography revealed prominent late disc staining without early filling delay. Magnetic resonance imaging showed no optic nerve enhancement or compressive lesion. The patient denied systemic symptoms, and laboratory evaluations were unremarkable.

Over the following month, optic disc edema resolved with development of superior segmental pallor. Visual acuity stabilized at 20/100 after hyperbaric oxygen therapy.

**Discussion**: NAION associated with uveal effusion syndrome is rare but has been reported in relatively young patients without systemic vascular comorbidities. This pattern suggests a potential role of local ocular factors related to uveal effusion in the development of ischemic optic neuropathy. Although sclerectomy is an established treatment for uveal effusion syndrome, NAION may occur postoperatively. Careful etiologic evaluation of optic disc edema in this setting is therefore essential. When optic disc edema is accompanied by an inferior altitudinal visual field defect, NAION should be considered, as visual recovery is often limited.

**Disclosures**: The authors declare no conflicts of interest.

165. **Indirect comparison of lenadogene nolparvovec gene therapy versus Natural History In Patients with m.11778G>A MT-*ND4* Leber Hereditary Optic Neuropathy Over 5 Years**

Valérie Biousse^1^, Patrick Yu-Wai-Man^2^, Nancy J. Newman^1^, Mark L. Moster^3^, Valerio Carelli^4^, Prem S. Subramanian^5^, Catherine Vignal-Clermont^6^, An-Guor Wang^7^, Sean P. Donahue^8^, Bart P. Leroy^9^, Robert C. Sergott^3^, Thomas Klopstock^10^, Alfredo A. Sadun^11^, Gema Rebolleda Fernández^12^, Bart K. Chwalisz^13^, Rudrani Banik^14^, Magali Taiel^15^, and José-Alain Sahel^16^, for the LHON Study Group

^1^Departments of Ophthalmology, Neurology and Neurological Surgery, Emory University School of Medicine, Atlanta, GA, USA; ^2^John van Geest Centre for Brain Repair and MRC Mitochondrial Biology Unit, Department of Clinical Neurosciences, University of Cambridge, Cambridge, UK; ^3^Departments of Neurology and Ophthalmology, Wills Eye Hospital and Thomas Jefferson University, Philadelphia, PA, USA; ^4^IRCCS Istituto delle Scienze Neurologiche di Bologna, Programma di Neurogenetica, Bologna, Italy; ^5^Sue Anschutz-Rodgers University of Colorado Eye Center, University of Colorado School of Medicine, Aurora, CO, USA; ^6^Department of Neuro Ophthalmology and Emergencies, Rothschild Foundation Hospital, Paris, France; ^7^Department of Ophthalmology, Taipei Veterans General Hospital, National Yang Ming Chiao Tung University, Taipei, Taiwan; ^8^Department of Ophthalmology, Neurology, and Pediatrics, Vanderbilt University, and Vanderbilt Eye Institute, Vanderbilt University Medical Center, Nashville, TN, USA; ^9^Department of Ophthalmology and Center for Medical Genetics, Ghent University Hospital, and Department of Head & Skin, Ghent University, Ghent, Belgium; ^10^Friedrich-Baur-Institute, Department of Neurology, LMU University Hospital, Ludwig-Maximilians-Universität München, Munich, Germany; ^11^Doheny Eye Institute, UCLA School of Medicine, Los Angeles, CA, USA; ^12^Department of Ophthalmology, Alcala University, Madrid, Spain; ^13^Department of Ophthalmology, Massachusetts Eye & Ear, Harvard Medical School, Boston, MA, USA; ^14^Department of Ophthalmology, Icahn School of Medicine at Mount Sinai, New York, NY, USA; ^15^GenSight Biologics, Paris, France; ^16^Department of Ophthalmology, University of Pittsburgh School of Medicine, Pittsburgh, PA, USA

**Introduction**: Lenadogene nolparvovec is a gene therapy for patients with Leber hereditary optic neuropathy (LHON) carrying the m.11778G>A MT-*ND4* variant. To estimate the efficacy of LN, we compared the visual acuity of patients with MT-*ND4* LHON treated with LN to the spontaneous evolution of visual acuity in untreated MT-*ND4* patients.

**Methods**: An indirect comparison approach was adopted. Data on best-corrected visual acuity (BCVA) of 174 MT-*ND4* LHON patients who received intravitreal injection in one or both eyes were pooled from four completed phase 3 clinical trials: REFLECT (unilateral or bilateral injection), REVERSE, RESCUE and RESTORE (unilateral injection). Final data at 5 years after LN injection were included to assess the persistence of LN effect over time. The external control group included 208 age-comparable (≥ 15 years old) untreated MT-*ND4* LHON patients from 11 natural history studies. Sensitivity analyses were performed to control for potential confounding factors of clinical interest (gender, ethnicity, age at onset of vision loss and duration of follow-up).

**Results**: Both cohorts included predominantly male patients (81%), with a median age at onset of vision loss of 26.0 years. Mean [standard deviation (SD)] timing of LN administration was 7.9 (3.3) months post vision loss. Mean (SD) time from vision loss to last observation was 4.9 (1.3) years for LN patients and 7.1 (11.0) years for natural history patients. At last observation, mean [95% confidence interval (CI)] improvement in BCVA versus natural history was -0.30 [-0.39; -0.22] LogMAR (+15 ETDRS letters equivalent) (p<0.01). When adjusting for covariates of interest, the estimated mean [95% CI] improvement was -0.46 [-0.56; -0.36] LogMAR (+23 ETDRS letters) versus natural history (p<0.0001). Most treated eyes were on-chart (LogMAR ≤ 1.6) compared to less than half of natural history eyes at last observation (75.3% vs 44.4%; p<0.01).

**Discussion**: This indirect comparison of treated patients with natural history patients confirmed a clinically meaningful and sustained improvement in BCVA induced by LN in patients with MT-*ND4* LHON.

**Disclosures**: VB is a consultant for GenSight Biologics, Chiesi, Topcon and Phelcom. PYWM is a consultant for GenSight Biologics, Chiesi and Neurophth Therapeutics. NJN is a consultant for GenSight Biologics, Chiesi, Stoke Therapeutics, Eli Lilly and Phelcom; has received research support from GenSight Biologics. MLM is a consultant for GenSight Biologics and has received research support from GenSight Biologics. VC is a consultant for GenSight Biologics, Chiesi, and Pretzel Therapeutics. PSS is a consultant for GenSight Biologics. CVC is a consultant for GenSight Biologics. BPL is a consultant for GenSight Biologics and has received research support from GenSight Biologics. RCS is a consultant for GenSight Biologics. TK received research support and/or personal compensation for presentations and/or consulting from GenSight Biologics and Chiesi Farmaceutici. AAS is a consultant for GenSight Biologics and Chiesi. MT is an employee of GenSight Biologics. JAS is a consultant for Avista Therapeutics, Essilor Luxottica and Lightstone ventures; is owner/co-owner/founder/co-founder and has personal financial interests (stock/stock options) in Gensight Biologics, Sparing Vision, Prophesee, Tilak Healthcare, Avista, Tenpoint, SharpEye, Cilensee and Netramind; is a member of the scientific advisory board of Gilbert Foundation, Foundation Fighting Blindness and Institute of Ophthalmology Basel; is an observer at Sparing Vision and Avista; is president of Fondation Voir et Entendre and StreetLab; is director of GenSight Biologics; is executive Vice-President of UPMC Enterprises; has patents for allotopic expression, Rod-Derived Cone Viability Factor and related patents; is a recipient for patent royalties and GenSight Biologic

168. **Enhanced detection of treatment benefit using a novel composite efficacy endpoint to evaluate the visual impact of idebenone in subacute/dynamic and chronic patients with Leber hereditary optic neuropathy: Post-hoc analysis of the LEROS study**

Xavier Llòria^1^, Nancy J. Newman^2^, Patrick Yu-Wai-Man^3-6^, Alfredo A. Sadun^7^, Valerio Carelli^8,9^, Rod Gossen^10^, Daniela Garavaglia^1^, Karen Sison Lance^10^, Caroline Fradette^10^, Thomas Klopstock^11-13^

^1^Chiesi Farmaceutici S.p.A, Parma, Emilia-Romagna, Italy; ^2^Departments of Ophthalmology, Neurology, and Neurological Surgery, Emory University School of Medicine, Atlanta, GA, USA; ^3^John van Geest Centre for Brain Repair, and MRC Mitochondrial Biology Unit, Department of Clinical Neurosciences, University of Cambridge, Cambridge, UK; ^4^Cambridge Eye Unit, Addenbrooke’s Hospital, Cambridge University Hospitals, Cambridge, UK; ^5^Moorfields Eye Hospital NHS Foundation Trust, London, UK; ^6^Institute of Ophthalmology, University College London, London, UK; ^7^Doheny Eye Institute, Department of Ophthalmology, University of California, Los Angeles, CA, USA; ^8^IRCCS Istituto di Scienze Neurologiche di Bologna, Bologna, Italy; ^9^Department of Biomedical and Neuromotor Sciences, University of Bologna, Bologna, Italy; ^10^Chiesi Canada Corp, Ontario, Canada; ^11^Friedrich Baur Institute at the Department of Neurology, LMU University Hospital, LMU Munich, Munich, Germany; ^12^German Center for Neurodegenerative Diseases (DZNE), Munich, Germany; ^13^Munich Cluster for Systems Neurology (SyNergy), Munich, Germany.

**Introduction**: The efficacy of idebenone in the treatment of visual impairment in Leber hereditary optic neuropathy (LHON) was evaluated in RHODOS (NCT00747487), a Phase II, 24-week, placebo-controlled trial; and LEROS (NCT02774005), a Phase IV, 24-month, open-label study. However, the influence of disease phase at treatment initiation on response remains unclear. This post‑hoc analysis evaluated treatment benefit using a novel composite efficacy endpoint in patients initiating idebenone in the subacute/dynamic (≥12 months since symptom onset at baseline) and chronic (>12 months since symptom onset at baseline) disease phases.

**Methods**: An integrated analysis from the RHODOS and LEROS trials assessed change in best-corrected visual acuity in the better-seeing eye (best BCVA) from baseline. The original measure of clinically relevant benefit (CRB) was defined as meeting criteria for clinically relevant recovery (CRR: improvement of ≥10 ETDRS letters on-chart or from off-chart to ≥5 ETDRS letters on-chart) and/or clinically relevant stabilisation (CRS: maintenance of a baseline best BCVA <1.0 logMAR). However, CRS includes any worsening vision that remains below this threshold. Alternatively, a novel composite efficacy endpoint of overall benefit (OB) was utilized, which retains the criteria for CRR but redefines maintenance of best BCVA as a change of -0.2< logMAR <+0.2 in patients who are on-chart at baseline. Clinically relevant worsening (CRW: deterioration of ≥10 ETDRS letters on-chart, or from on-chart to off-chart) was also assessed.

**Results**: At baseline, subacute/dynamic patients had a median best BCVA of 1.38 logMAR compared with 1.52 logMAR in chronic patients. Median change in best BCVA from baseline in logMAR in subacute/dynamic and chronic patients, was 0.00 and -0.04 at Month 6, -0.02 and -0.09 at Month 12, and -0.17 and -0.08 at Month 24, respectively. Rates of OB were greater than CRB across both groups at each timepoint. At Month 24, 48% of patients in both groups achieved a CRB, whereas 65% and 79% of the subacute/dynamic and chronic groups experienced an OB, respectively. Lower rates of CRW were observed in the chronic group when compared with the subacute/dynamic group.

**Discussion**: Treatment benefit measured using CRB or OB was observed in patients initiating idebenone in both disease phases, with a greater magnitude of best BCVA improvement observed in patients who initiated treatment earlier and for a longer duration. The rate of OB was consistently higher than CRB in both groups. The observed rates of CRW align with the typical disease course of LHON. Further investigation is needed to understand the clinical decision-making implications of this novel endpoint.

**Disclosures**: XL and DG are employees of Chiesi Farmaceutici S.p.A. NJN has been a consultant for Chiesi, GenSight, Stoke, Phelcom, and Eli Lilly. PYWM received research support and consultation fees from Chiesi, GenSight Biologics, Stoke Therapeutics, and PYC Therapeutics. AS has been a consultant for Chiesi and GenSight Biologics and has received research support from GenSight Biologics and Stealth Therapeutics. VC and TK received research support and consultation fees from Santhera Pharmaceuticals, Chiesi, and GenSight Biologics. RG, KSL, and CF are employees of Chiesi Canada Corp. Medical writing support was provided by Cactus Life Sciences, and funded by Chiesi Farmaceutici S.p.A.

169. **Changepoint timing of retinal thinning after optic neuritis and its clinical implications**

Yeji Moon^1^, Byung Joo Lee^1^, Eun-Jae Lee^2^

^1^Department of Ophthalmology, Asan Medical Center, University of Ulsan College of Medicine, Seoul, Republic of Korea; ^2^Department of Neurology, Asan Medical Center, University of Ulsan College of Medicine, Seoul, Republic of Korea

**Purpose**: To estimate the timing of structural stabilisation (“changepoint”) in peripapillary retinal nerve fibre layer (pRNFL) and macular ganglion cell–inner plexiform layer (mGCIPL) thinning after optic neuritis (ON), and to identify clinical correlates and prognostic significance.

**Methods**: In this retrospective cohort study, we analysed 70 eyes from 56 adults with ON (AQP4-ON, MOG-ON, MS-ON, and idiopathic ON). Eligible eyes had baseline OCT within 1 month of symptom onset and ≥12 months of recurrence-free follow-up. Longitudinal pRNFL and mGCIPL thickness were interpolated at fixed intervals. Stable-phase thinning was estimated from 6–18 months post-onset. The changepoint was defined as the earliest time point when observed thickness aligned with the extrapolated stable-phase regression within test–retest thresholds (3 *µ*m for pRNFL; 2 *µ*m for mGCIPL). Associations between changepoint timing, clinical variables, and 1-year visual outcomes were assessed using non-parametric statistics.

**Results**: Across all eyes, mean changepoints occurred later for pRNFL than for mGCIPL (4.3 ± 1.2 vs. 3.4 ± 1.2 months, p < 0.001). MOG-ON showed later pRNFL stabilization compared with AQP4-ON and idiopathic ON, while mGCIPL changepoints were comparable across subtypes. Later pRNFL changepoints were associated with younger age and greater baseline pRNFL thickness. Later mGCIPL changepoints were associated with worse baseline visual acuity, lower baseline mGCIPL thickness, and longer onset-to-steroid interval. Importantly, delayed mGCIPL stabilization was significantly associated with poorer 1-year visual outcomes, including worse visual acuity and worse visual field indices.

**Conclusions**: mGCIPL stabilises earlier than pRNFL after ON and its changepoint timing reflects baseline injury severity and treatment delay, with prognostic value for 1-year visual outcomes. Changepoint-based OCT analysis offers a quantitative framework for monitoring ON dynamics and highlights the importance of early anti-inflammatory treatment to limit neuroaxonal injury.

**Disclosures**: The authors report no conflicts of interest.

171. **Structural and functional changes in retina and optic nerve head in NAAION**

Lepša Žorić

Eye Clinic, UCC of Serbia, Belgrade; Faculty of Medicine, UPKM, Kosovska Mitrovica

**Introduction**: Non-arteritic anterior ischemic optic neuropathy (NAAION) is an infarct of optic nerve head (ONH). Optical coherent tomography (OCT) provides insight in retinal ganglion cells (GCL/GCC(GCL and IPL)) and belonging peripapillary retinal nerve fiber layer (RNFL) changes in this disease. OCT angiography (OCTA) enables precise location of excluded parts of the circulation of optic nerve head (ONH) and retina and quantitative insight into reduction of the blood vessels density. Pattern reversal electroretinography (PERG) provides information about the functional state of retinal ganglion cells (N95 wave).

**Method**: Among regularly followed up patients with optic neuropathies, during the past year, 6 had NAAION (3 women and 3 men, average age 42). Data are obtained from OCT, OCTA and PERG, at the time of the first examination, and following up after 1, 3 and 6 months.

**Results**: After initial thickening, RNFL shows thinning, during next months. Ganglion cells layer starts to thin after a couple weeks and this process continues, measured by OCT, as well as their functional state declines, measured by PERG (N95). Blood vessels of superior half and temporal quadrant are most seriously damaged in our patients, according to OCT angiography. The distribution of damages is consistent with the visual field scotomas.

**Conclusion**: Our patients are younger than is usually expected in this pathology. In NAAION damages of retinal nerve fibers and ganglion cells develops rapidly. This can be expected with this etiopathogenesis. The above methods allow for the anatomical location of the damages and monitoring of function of some retinal parts.

**DISORDERS OF RETROCHIASMAL PATHWAYS AND VISUAL PROCESSING**

9. **Repetitive transcranial magnetic stimulation for visual snow syndrome: symptoms relief and changes in functional brain dynamics. A case report.**

Alessia Bargagli^1^, Francesco Neri^2^, David De Monte^2^, Alberto Benelli^2^

^1^Eye Tracking and Visual Application Lab (EVALab), Neurology Unit, Department of Medicine, Surgery and Neuroscience, University of Siena, Siena, Italy; ^2^Siena Brain Investigation and Neuromodulation Lab (Si-BIN Lab), Department of Medicine, Surgery and Neuroscience, Neurology Section, University of Siena, Italy

**Introduction**: Visual Snow Syndrome (VSS) is a rare and debilitating neurological disorder characterized by the persistent perception of dynamic, continuous minute dots across the entire visual field. It is typically accompanied by at least two additional visual symptoms, including palinopsia, photopsia, nyctalopia, and entoptic phenomena. The neuropathophysiology underlying VSS remains poorly understood; however, neuroimaging and electrophysiological studies suggest that it may be linked to dysfunction in visual processing, possibly due to hyperexcitability within the visual brain network.

**Case Report**: We report the case of an 18-year-old female affected by VSS, refractory to currently available pharmacological treatments, who showed a positive response to 1-Hz repetitive Transcranial Magnetic Stimulation (rTMS) targeting the bilateral superior parietal lobule (SPL).

The treatment consisted of ten neuronavigated rTMS sessions administered once daily over two consecutive weeks. Low-frequency (1-Hz) stimulation was delivered bilaterally over the SPL at an intensity set at 100% of the lowest phosphene threshold (PT). Clinical and neurophysiological evaluations were conducted before (T0) and immediately after the intervention (T1). Visual disturbances were assessed using a self-report using a VisionSimulation online platform, which allowed the patient to simulate her subjective visual perception. Changes in resting-state functional connectivity (rs-FC) between the SPL and the rest of the brain were analyzed with a seed-based qualitative analysis. Additionally, five minutes of resting-state electroencephalography (rs-EEG) data were collected.

At T1, the patient reported a reduction in perceived density of visual dots. Functional Magnetic Resonance Imaging (fMRI) analysis revealed a rs-FC reduction between the SPL and the lingual gyrus (LG) in both hemispheres. Resting-state EEG power spectral density (PSD) analysis showed a decrease in both the low and high gamma bands in parieto-occipital regions during both eyes-open and eyes-closed conditions.

**Discussion**: The described case is unique since it shows that subjective improvement is related to changes in brain dynamics, as revealed by fMRI and EEG. At T1, the patient reported significant symptom relief. These subjective clinical improvements were accompanied by a reduction in rs-FC between the SPL and the LG, as well as a decrease in loco-regional rs-EEG gamma-band power.

These findings are likely attributable to the application of 1-Hz rTMS, a protocol known for its inhibitory influence on cortical activity. The observed neurophysiological changes within the visual network suggest that rTMS induced neuromodulation of hyperexcitability within the visual network, helping in restoring physiological neural dynamics. However, a key limitation of this study is the lack of placebo condition and of long-term follow-up data. Further studies warranted to assess the durability of these effects and to optimize stimulation protocols for sustained clinical benefit.

**Disclosures**: The authors declare no conflicts of interest.

18. **Charles Bonnet syndrome: Service audit of 160 patients (2017–2025) and management pathway update**

Anna Visca^1^, Nima Ghadiri^1^

^1^Royal Liverpool University Hospital, Liverpool, UK

**Aim**: To characterize patients diagnosed with Charles Bonnet syndrome (CBS) at a tertiary ophthalmology service and to evaluate documentation of distress and support offered, to inform a revised management pathway.

**Methods**: Retrospective service audit of consecutive patients coded/diagnosed with CBS (2017–2025) using the Medisight electronic record at Royal Liverpool University Hospital. Extracted data included demographics, underlying cause of visual impairment, visual acuity, documented distress/anxiety, and management actions (reassurance/education, counseling, ECLO referral).

**Results**: 160 patients were included; 56% were female and median age was 76 years. Underlying drivers of visual impairment included age-related macular degeneration (44%), glaucoma (12.5%), central retinal vein occlusion (6%) and retinal detachment (6%); neurological visual pathway insults were also recorded (stroke 25%, subarachnoid hemorrhage 12.5%). Visual acuity ranged from 6/12 to no light perception. Although most patients were documented as coping following explanation of CBS, 31% had significant distress/anxiety related to hallucinations. Management was predominantly reassurance and counseling; 22% received formal counseling and 19% were referred to an Eye Clinic Liaison Officer (ECLO)

**Conclusion**: By synthesizing these audit findings with a contemporary literature update, this report identifies a clear gap in the care of the high-anxiety subgroup. We propose a refined clinical pathway that integrates targeted psychological and, where indicated, pharmacological interventions, ensuring a more robust support system for patients who do not respond to reassurance alone.

123. **Vitamin deficiency in patients with visual snow syndrome**

Nelli Galoyan

Department of Ophthalmology, Wexner Medical Center, The Ohio State University, Columbus, OH, USA

**Introduction**: The pathophysiology of visual snow syndrome (VSS) appears to involve cortical hyperexcitability, dysfunctional thalamocortical connectivity, and network disturbances affecting visual processing. Most cases are spontaneous; however, potential secondary causes of VSS have been described, including post-concussion, post-infection, migraine, hallucinogen persisting perception disorder, ocular abnormalities, idiopathic intracranial hypertension, and posterior cortical atrophy. Vitamin deficiencies are not among the known secondary causes of VSS, although they can cause various ophthalmological manifestations.

**Aim**: To assess vitamin levels in patients with VSS and to evaluate subjective improvement of VSS with vitamin supplementation in cases of deficiency.

**Methods**: Twelve patients (4 males and 8 females) who met diagnostic criteria for VSS, with a mean age of 32.5 ± 10.5 years (mean ± SD), were evaluated for vitamin deficiencies. Vitamins A, B1, B9, and B12 were assessed.

**Results**: Seven patients were found to have vitamin B12 levels below 400 pg/mL, ranging from 271 to 379. Five of these seven patients did not notice any improvement of VSS with vitamin B12 supplementation, and two patients were lost to follow-up.Vitamin B1 deficiency was identified in two patients, both of whom reported improvement of VSS with vitamin B1 supplementation.One patient was found to have vitamin B9 deficiency and did not report any improvement of VSS with B9 supplementation.One patient had vitamin A deficiency and did not notice any improvement of VSS with vitamin A supplementation; however, the treatment duration was less than one month.Overall, only three of the twelve patients had normal levels of all four vitamins evaluated.

**Discussion**: Although vitamin deficiency is not a known secondary cause of VSS, it can result in visual disturbances such as decreased visual acuity, impaired color vision, reduced contrast sensitivity, nyctalopia. This case series demonstrates that vitamin deficiencies are common among patients with visual snow syndrome. However, it remains unclear whether secondary VSS may be associated with vitamin deficiency. Vitamin B12 supplementation did not improve VSS symptoms in our patients, despite B12 deficiency being the most common finding in this case series. Notably, both patients with thiamine deficiency reported improvement of VSS following vitamin B1 supplementation. Assessment of vitamin levels and treatment of deficiencies may be beneficial in patients with VSS. Nevertheless, this case series includes only 12 patients, and larger studies are needed to evaluate the association between vitamin deficiency and VSS.

**Disclosers:** I have no financial interests to disclose.

142. **On the right track(t)**

Rogan G. Fraser^1^, Musa China^2^, Patrick Grover^2^, Chandrashekar Hoskote^3^, Eoin Finegan^4^, Neringa Jurkute^4,5^

^1^Moorfields Eye Hospital NHS Foundation Trust, London UK; ^2^Department of Neurosurgery, The National Hospital for Neurology and Neurosurgery (NHNN), University College London Hospitals NHS Foundation Trust (UCLH), London, UK; ^3^Lysholm Department of Neuroradiology, NHNN, UCLH, London, UK; ^4^Department of Neuro-ophthalmology, The National Hospital for Neurology and Neurosurgery, University College London Queen Square Institute of Neurology, The National Hospital for Neurology and Neurosurgery, London, UK; ^5^Institute of Ophthalmology, University College London, London, UK.

**Introduction**: Optic tract lesions can create both diagnostic and therapeutic challenges. While tumours are a common cause of this uncommon condition, cavernous malformations at the optic chiasm are rare. We report a unique case of a chiasmatic cavernous malformation presenting with acute symptoms and with an atypical constellation of clinical signs. This case highlights the clinical and radiological features, multidisciplinary decision-making, and longitudinal structure–function evolution in a dynamic lesion affecting both pre-and post-chiasmatic optic pathway.

**Case Report**: A 32-year-old woman presented with sudden onset left-sided visual loss on waking, associated with retro-orbital pain but no headache, trauma, or focal neurological deficits. Visual acuity was 6/6 bilaterally with preserved colour vision, but with a right relative afferent pupillary defect. Automated perimetry demonstrated a congruous left homonymous hemianopia. A computed tomography scan revealed a hyperdense right suprasellar lesion. Subsequent magnetic resonance imaging (MRI) revealed T2 signal abnormality extending along the right optic nerve but with marked susceptibility on susceptibility-weighted imaging affecting the right optic tract, suspicious for blood products extending from the lesion into the tract.

Initial differential diagnoses included glioma, other primary tumour, and cavernous malformation. Initial multidisciplinary review across neuro-ophthalmology, neuro-oncology, neurosurgery, and neurovascular teams recommended conservative management with interval imaging. Serial examinations during this time showed stable visual acuity and colour vision with slight improvement of left field, a change that was concordant with a change in lesion size on MRI. However, over the same period optical coherence tomography demonstrated progressive thinning of the peripapillary retinal nerve fibre layer and macular ganglion cell layer bilaterally, in a pattern characteristic of an optic tract (or post-chiasmatic) lesion.

Given progressive ganglion cell layer atrophy, and perceived risk of further, potentially sudden decline with intra- or juxta-lesional haemorrhage, surgical resection was ultimately recommended. Post-operatively, the patient retained good central visual acuity, with stabilisation of left field but progression of right sided optic neuropathy as evidenced by worsening field defect and progressive ganglion cell layer atrophy.

**Discussion**: This case illustrates the atypical presentation of a chiasmatic cavernous malformation with initial clinical signs manifesting as a combination of an optic tract syndrome and a (pre-chiasmatic) optic neuropathy. Careful radiological interpretation, longitudinal neuro-ophthalmic assessment, and multidisciplinary collaboration are essential to guide timing of intervention and optimise visual outcomes.

**Disclosures**: None.

**INTRACRANIAL HYPERTENSION**

**13. Case report of papilledema associated with meningioma**

Ana Srdoč Grbac, Maja Mrak, Tea Majnarić, Tamara Mišljenović Vučerić, Tea Čaljkušić-Mance

Department of Ophthalmology and optometrics, Clinical Hospital Centre Rijeka, Rijeka, Croatia,

**Introduction:** Papilledema is a sign of elevated intracranial pressure and may arise from a variety of causes, including mass lesions affecting venous outflow. Early recognition and identification of the underlying etiology are critical to prevent permanent visual impairment.

**Case Report:** A 39-year-old male patient was evaluated at the ophthalmology private practice due to short-term blurring of the image when changing body position, especially when standing up, which had been present for one month. He denied headaches and double vision or any other subjective complaints.

At the time of the initial examination, the patient was undergoing evaluation for allergic dermatitis. Approximately 10 days before the first visit, systemic corticosteroid therapy was initiated (prednisone 25 mg) with gradual dose tapering. The patient had been on desloratadine therapy for one month. He had no history of any other chronic medical conditions and no other chronic medications were reported.

Fundus examination revealed advanced optic disc edema and teleangiectasias in both eyes.

Visual acuity, slit-lamp findings and Ishihara test were normal. Visual field testing showed a minor bilateral temporal scotoma in the far periphery.

Given the clinical findings, the patient was referred for urgent neuroradiological evaluation. Neurological examination was unremarkable. Brain computed tomography (CT) and magnetic resonance imaging (MRI), together with CT venography, revealed no pathological findings. Oral acetazolamide therapy was initiated.

The brain MRI was reevaluated, revealing an expansive lesion compressing the superior sagittal sinus with significant narrowing, most likely representing a meningioma. The patient was evaluated by a neurosurgeon and subsequently underwent surgical treatment, with histopathology confirming the lesion as a meningioma. The patient reported complete resolution of visual disturbances 24 hours after surgery.

**Discussion:** This case report describes secondary intracranial hypertension caused by a parasagittal meningioma compressing the superior sagittal sinus, likely leading to impaired cerebrospinal fluid absorption and subsequent development of papilledema.

Interestingly, initial neuroradiological assessment failed to identify the causative lesion, emphasizing that small or strategically located tumors may be overlooked. Careful reevaluation of imaging studies, preferably by experienced neuroradiologists, is essential when clinical findings strongly suggest elevated intracranial pressure.

The use of systemic corticosteroids in this patient may represent a confounding factor. Although corticosteroids are not a well-established cause of papilledema, their use shortly before the onset of symptoms may have influenced intracranial dynamics. However, the rapid and complete resolution of visual symptoms and papilledema following surgical decompression strongly supports compression of the venous sinus by the meningioma as the primary etiological factor.

**Disclosures:** We declare no conflict of interest.

14. **Visual risk-stratification and management in Idiopathic Intracranial Hypertension**

Ana Belén Tárraga Ibáñez^1^, Sara Khanji-Fustek^1^, Teresa Albert-Albelda^2^, Ángeles Bort-Marti^1^

^1^Ophthalmology department - La Fe University and Polytechnic Hospìtal - Valencia, Spain; ^2^Neurology Department - Bellvitge University Hospital - IDIBELL, University of Barcelona - Barcelona, Spain

**Introduction**: Idiopathic Intracranial Hypertension (IIH) is a rare disease, but its incidence is increasing in Europe (9.3/100,000 inhabitants/year) and it is twice as common in young obese women. Its clinical presentation is extraordinarily variable and none of the symptoms are pathognomonic for the disease, nevertheless, visual symptoms at onset represent a high risk of severe and permanent visual impairment. The recent adaptation by Friedman of the classic modified Dandy diagnostic criteria gives special relevance to the presence of papilloedema or sixth cranial nerve palsy, although overdiagnosis based on errors in the assessment of the optic nerve is common. Controlled and randomised studies in IIH report highly heterogeneous outcomes, resulting in few systematic reviews. The duration, phases of the disease and safe monitoring of visual function are not clearly defined. The proposal for monitoring visual function by Mollan et al., published in 2018, represents a significant advance in the management of these patients.

**Methods**: We present a series of 22 patients diagnosed with IIH between January and December 2025, referred from the Emergency, Neurology, and Neurosurgery Departments of a tertiary hospital. We reviewed the diagnostic criteria applied and the ophthalmologic monitoring performed.

**Results**: Only 11 patients fulfilled Friedman’s criteria A to E with papilloedema confirmed by an expert neuro-ophthalmologist. Only one patient presented with bilateral sixth nerve palsy, corresponding to a case of fulminant IIH with severe papilloedema. Patient monitoring included the thickness of the Retinal Nerve Fiber Layer, values in Ishihara Test and pupillary evaluation, as well as the degree of papilloedema and visual field impairment. Mollan’s proposal was followed in seven patients.

**Discussion**: Despite the recognised importance of visual field testing and fundoscopic evaluation in idiopathic intracranial hypertension, there is currently no clear consensus on how to objectively use these tools to guide follow-up across different disease stages. We propose a risk-based approach using quantitative parameters derived from papilloedema assessment and mean deviation on automated perimetry to better define disease evolution and standardise visual monitoring, particularly in centres without specialised neuro-ophthalmology units.

**Disclosures**: The authors declare no financial interests or conflicts of interest related to this study.

21. **The role of transorbital ultrasonography and optical coherence tomography in the follow-up of idiopathic intracranial hypertension**

Ayşe Kurt^1^, Mert Göbel^1^, Hülya Ertaşoğlu Toydemir^1^, Hacı Ali Erdoğan^1^, İbrahim Acır^1^, Aslı Vural^2^, Vildan Yayla^1^

^1^Bakirkoy Dr. Sadi Konuk Training and Research Hospital, Department of Neurology, Istanbul, Turkey; ^2^Bakirkoy Dr. Sadi Konuk Training and Research Hospital, Department of Ophthalmology, Istanbul, Turkey

**Introduction**: Idiopathic intracranial hypertension is characterized by elevated intracranial pressure without an identifiable secondary cause, and measurement of cerebrospinal fluid opening pressure by lumbar puncture remains a cornerstone of diagnosis. In our clinic, a study conducted in 2024 evaluated the clinical value of transorbital ultrasonography (TOUS) and optical coherence tomography (OCT) alongside CSF opening pressure in the diagnosis of IIH; the findings suggested that these modalities may serve as complementary tools for the noninvasive monitoring of intracranial pressure elevation and papilledema. Patients were followed in the neuro-ophthalmology outpatient clinic, and TOUS and OCT examinations were repeated after approximately one year. The aim of this study is to assess changes in TOUS and OCT parameters before and after treatment in patients with IIH and to correlate these changes with clinical findings, thereby investigating the role of TOUS and OCT in monitoring treatment response and their applicability in patient follow-up.

**Methods**: The study was conducted between June 1 and October 1, 2025, at Bakırkoy Dr. Sadi Konuk Training and Research Hospital. Secondary to elevated intracranial pressure, optic nerve sheath diameter (ONSD) enlargement can be assessed by TOUS, while papilledema can be demonstrated on OCT via increased retinal nerve fiber layer (RNFL) thickness. Of the 31 patients in the reference study, 25 were included. At a mean follow-up of 15 months, TOUS and OCT measurements were repeated in all patients. Patients were followed according to clinical severity with acetazolamide, weight-loss recommendations, or observation without medication. TOUS and OCT measurements were performed according to standard protocols and evaluated separately for each eye; data were analyzed statistically and compared with ONSD and RNFL values from the reference study.

**Results**: Among the 25 participants, 24 were female and 1 was male, the mean age was 35 years. Of the 25 patients scheduled for follow-up, two did not attend; at a mean follow-up of 15 months, 10 of the 23 patients who remained under follow-up were still receiving acetazolamide. Treatment was completed in eight patients, one patient discontinued therapy due to adverse effects, and four patients were monitored without pharmacological treatment. Thirteen patients expressed significant weight loss. Following treatment (medication and/or weight-loss), TOUS and OCT measurements showed statistically significant changes compared to baseline. ONSD assessed by transorbital ultrasonography decreased significantly (p<0.001). OCT demonstrated a significant reduction in RNFL thickness, indicating improvement in papilledema (p<0.001).

**Discussion**: These findings support TOUS-derived ONSD and OCT-derived RNFL thickness as complementary, noninvasive markers for monitoring in idiopathic intracranial hypertension. Changes in ONSD and RNFL measurements may reflect treatment-related improvement and can provide objective support to clinical assessment.

**Disclosure**: The authors declare no conflicts of interest.

28. **Papilledema quantification with OCT: An unsupervised Deep Learning Algorithm outperforms the Heidelberg commercial software**

Bilal Omari^1^, Mehdi Ounissi^2^, Ana Banc^1,2^, Dan Milea^1,2^

^1^Neuro-ophthalmology department. Rothschild Foundation Hospital, Paris, France; ^2^Rothschild Computational and Visual Neurosciences Laboratory, Adolphe de Rothschild Foundation Hospital, Paris, France.

**Introduction**: Optical Coherence Tomography (OCT) has become a standard evaluation tool for the diagnosis and monitoring of papilledema associated with intracranial hypertension. However, the OCT-based quantification of the peripapillary retinal nerve fiber layer (pRNFL) thickness remains imprecise, especially in severe papilledema, due to segmentation errors. Such common errors arise because of the lack of specific built-in OCT algorithms, to auto segment papilledema. The aim of our study was to develop, train and test a new deep learning segmentation algorithm (DLSA), able to automatically segment and quantify pRNFL in papilledema. Its performance was evaluated by comparing the DLSA results with the results provided by the associated commercial software (HEYEX, Heidelberg Eye Explorer software - Spectralis SD-OCT, Heidelberg Engineering, Heidelberg, Germany), validated via a ground truth established by human graders.

**Methods:** The training of the DLSA was performed on OCT data obtained in 128,765 patients (807,512 OCT B-scans), with adaptations for noise and artifact.

Testing was performed on a large, independent retrospective dataset of 260 patients (405 eyes) with papilledema due to confirmed intracranial hypertension, diagnosed on standard modified Dandy criteria. For each eye, colour fundus photographs (CFP) and the corresponding raw data of SD-OCT pRNFL circular scans (Spectralis SD-OCT) were evaluated. The severity of papilledema was anonymously graded on the CFP, using the Modified Frisen Scale. The corresponding pRNFL boundaries were automatically segmented using both the DLSA and HEYEX and validated against a ground truth established by two human observers. Segmentation errors were compared between DLSA and Heyex using the McNemar test with Holm adjustment.

**Results:** Among the included 405 eyes with papilledema, the Frisen scale-based severity was the following: grade 1 (22:5.4%), grade 2 (112:27.6%), grade 3 (127:31.3%), grade 4 (116: 28.6%), and grade 5 (28:6.9%). Our DLSA successfully detected the internal limiting membrane in 97.9% and the pRNFL in 94.8% of scans, compared to 89.6% and 50.8% for HEYEX, respectively. The pRNFL segmentation failure rates for DLSA vs. HEYEX were: Frisen 1 = 0% vs. 59% (p < 0.0001), Frisen 2 = 8% vs. 80.4% (p < 0.0001), Frisen 3 = 8.7% vs. 87.4% (p < 0.0001), Frisen 4 = 19.8% vs. 88.8% (p < 0.0001), and Frisen 5 = 7.1% vs. 100% (p < 0.0001). The cumulative error rate in severe cases (Frisen grades 4–5) was 17.4% with our DLSA, compared to 91%, as provided by HEYEX (p < 0.0001).

**Discussion:** Conventional commercially software-based segmentation and quantification of pRNFL thickness in papilledema is imprecise, especially in severe cases. Our DLSA outperforms the segmentation provided by the available HEYEX software, across all Frisen grades of papilloedema. These preliminary results suggest the potential role of this DLSA to improve the evaluation and management of papilledema due to intracranial hypertension.

**Disclosures**: The authors have no conflicts of interest to disclose.

45. **Recurrent idiopathic intracranial hypertension following unilateral transverse venous sinus stenting**

Casandra MacLeod^1^, M. Shazam Hussain^1^, Devon A. Cohen^1^

^1^Cleveland Clinic Foundation, Cleveland, OH, USA

**Introduction:** The use of transverse venous sinus stenting is becoming more popular for treating medical refractory idiopathic intracranial hypertension (IIH). In patients with recurrent symptoms following initial stent less is known about the indication and utility of secondary stenting.

Description of Cases: 119 Patients were seen at a tertiary care center between August 2020 and June 2025 who met the Dandy criteria for IIH and underwent venous sinus stenting. We present 7 cases who ultimately required a second stent due to recurrent symptoms and/or worsening ophthalmic exam. Patients were all female, median, standard deviation (SD) age was 31 ± 5, body mass index 35.51 ± 11. Initial magnetic resonance venography (MRV) imaging revealed bilateral transverse sinus stenosis in 2 of 7 patients, with remaining having stenosis in the dominant transverse sinus. Each underwent unilateral stenting to a transverse sinus. In the setting of a co-dominant system, the stent was deployed to the side with the higher pressure gradient, median 13 mm Hg ± 5. In a dominant system, the dominant transverse sinus was targeted. The patients re-presented with clinical manifestations including pulsatile tinnitus, headache, or vision loss. Recurrent or worsening optic edema was only present in 2 of 7 patients. One patient without worsening edema had new complete peripheral visual field loss in the right eye. Another patient experienced a decline in visual acuity, from 20/20 -2 OD, 20/25 -1 OS to 20/20 -1 OD, 20/60 +2 OS. The remaining 3 patients had stable or improved neuro-ophthalmic exams. Repeat MRV did not show evidence of in-stent stenosis in any patients. Those with co-dominant systems demonstrated worse contralateral stenosis, while dominant systems demonstrated proximal stenosis to the previous stent. Following repeat cerebral angiogram, a second stent was placed in each patient. One patient with a co-dominant system received both contralateral stenting and a stent proximal to the prior stent. The median time between stent placement was 182 days ± 348. On follow-up, patients reported varying symptomatic relief after re-stenting in conjunction with improvement or stability in their neuro-ophthalmic exam.

**Conclusions:** We report 7 cases of previously stented IIH patients with clinical recurrence who required a second stent. Only 2 patients had co-dominant venous systems, while the remaining 5 had a dominant system and developed proximal stent stenosis. Based on these observations, future prospective studies on stenting in transverse venous sinus stenosis should be considered to optimize stent position and placement in dominant versus co-dominant systems.

59. **The cautious seldom err!!!–pediatric idiopathic intracranial hypertension – our experience**

Vidhya Dharani^1^, Ambika Selvakumar^1^

Department of Neuro ophthalmology, Sankara Nethralaya, Medical Research Foundation, Chennai, India

**Purpose**: To describe clinical manifestations, investigations and treatment response of idiopathic intracranial hypertension (IIH) in pediatric age group.

**Methods**: Electronic medical records of eight children diagnosed as IIH based on modified Dandy’s criteria from 2017-2025 were analyzed retrospectively. We included patients < 18 years of age with minimum post treatment follow up of 6 months. Excluded children with other optic neuropathies, ocular pathologies and poor documentation Detailed history of associated illness, vaccination, demographics, clinical presentation, neuroimaging features, treatment modalities and visual outcomes were recorded

**Results**: This group had male predilection with the ratio of boys to girls being 5:3. The youngest patient was a one year old boy. Five of eight patients had higher body mass index (BMI). Most common presenting symptoms were of headache and diplopia which was present in six patients. Visual acuity in LogMAR units was 0.0 in 5 patients. Seven patients presented with bilateral disc edema with Frisen grade 1-3. MRI brain and orbit revealed widened perioptic CSF space, empty sella in seven patients. MRV brain was normal in all. Average cerebrospinal opening pressure was 310 mmH2O. Spectral domain optical coherence tomography (SD OCT) of retinal nerve fiber layer (RNFL) and of ganglion cell complex (GCC) was available in five children. All were treated with oral acetazolamide 15-30mg/kg body weight in 1-4 divided doses. One patient had fulminant IIH with LogMAR units of 1.50 in right eye, 0.3 in left eye with bilaterally constricted visual fields at presentation needed surgical intervention (optic nerve sheath fenestration).

Treatment duration varied between 3- 9 months. Cerebral antiedema medications were titrated based on resolution of symptoms and disc edema. Seven patients had complete recovery with medical management except the patient with fulminant IIH. The kid with fulminant IIH had persisting profound visual field constriction. One kid developed recurrent papilledema following an unduly weight gain 2 years later.

**Conclusion**: IIH is uncommon in pediatric age group and is less frequently reported in prepubertal age. Early and prompt treatment is needed to prevent permanent loss of vision in young children and adolescent kids. Medical management can be rewarding in early stages of disease but may result in nonsalvageable vision loss as in the atypical fulminant IIH patients. Primary care givers must be aware of adverse effects of obesity in children, healthy life style must be emphasized.

**Disclosures**: The authors declare no financial conflict of interest.

60. **Differentiate with value or die with price!!–Risk factor analysis of visual outcomes of papilledema following ONSF in typical versus atypical IIH**

Selvakumar Ambika^1^, Padmalakshmi Krishnakumar^1^

^1^Department of Neuro ophthalmology, Sankara Nethralaya, Chennai, India

**Objective:** Idiopathic intracranial hypertension, the most common cause of papilledema predominantly occurs in young obese females. There is limited data on the subset of papilledema patients who have atypical IIH features. We aim to evaluate the clinical features and compare visual outcomes of optic nerve sheath fenestration in typical vs atypical IIH patients.

**Methods**: We retrospectively reviewed the medical records of patients who have undergone ONSF at our tertiary eye care centre in South India between 2014-2023 for vision-threatening papilledema due to idiopathic intracranial hypertension and other etiologies. Patients with other ocular pathology, optic neuropathies due to ischemic or infectious etiologies, pediatric group and those with inadequate follow-up post-surgery were excluded from the study. Detailed history including demographic profile, presenting symptoms and their duration, systemic history (including hypercoagulable disease), and prior surgical interventions (CSF diversion procedure, venous sinus stenting, and embolization) were noted. Visual acuity was recorded using Snellen’s visual acuity chart and its logarithm of minimal angle of resolution (logMAR) equivalent was noted. Demographics, neuroimaging features, CSF pressure, pre and post-ONSF clinical features including visual acuity, optic disc changes,OCT - RNFL and GCC measurements and visual fields were analysed.

**Results**: 68 eyes of 34 patients were evaluated. The mean age was 42.2 ± 12.7 years. 17 patients (50 %) were males and 17 (50 %) were females. Bilateral presentation was seen in all 34 patients. Mean initial visual acuity (logMAR) was 1.86 ± 0.81. Idiopathic intracranial hypertension (58.8%) was the most common cause of papilledema. OCT average RNFL thickness was 228±131.5microns and GCC thickness of 50.2 ± 11.88 microns. Unilateral ONSF was done in 32 patients and 2 patients had bilateral surgery. Mean final visual acuity (logMAR) was 1.46 ±1.03. The reduction in RNFL swelling was statistically significant compared to the initial presentation (p <0.001). The mean CSF opening pressure was 32.4 ± 7.2cms H20 (31 patients). The median duration of follow-up was 3 months. An improvement or stabilization in BCVA at final follow-up was considered as surgical success and was seen in 45 of 68 eyes (66.17%) [Atypical IIH - 24 eyes, Typical IIH - 21eyes]

**Conclusion**: ONSF preserved vision not only in papilledema due to idiopathic IIH patients but also in secondary papilledema patients,post-CSF diversion procedures and AVF embolization. However, patients presenting with profound vision drop, optic atrophy and atypical IIH were poor prognostic factors for visual outcome.

62. **Asymptomatic bilateral optic disc edema in an 18-Year-Old myopic patient: Radiologic evidence of incidental diagnosis of idiopathic intracranial hypertension**

Dudău Maria^1*^, Vlad Octavian Bolocan^2^, Alexandru Dimancea^3^, Silviu Dumitrescu^3^

^1^Victor Babes National Institute of Pathology, Bucharest, Romania; ^2^Carol Davila University of Medicine and Pharmacy, Bucharest, Romania; ^3^National Institute of Neurology and Neurovascular diseases, Bucharest, Romania

**Case Presentation:** An 18-year-old female with a history of myopia and astigmatism, presented for a routine ophthalmologic examination without any other complaints. Best-corrected visual acuity was 20/20 bilaterally, and color vision was normal. Anterior segment examination was unremarkable. Her body mass index was within normal limits and without any medication or other medical history. Eye examination revealed bilaterally elevated, pale optic discs with blurred margins, raising suspicion for optic disc edema. Standard automated perimetry demonstrated enlarged blind spots in both eyes. Optical coherence tomography confirmed optic nerve head elevation without evidence of optic nerve head drusen. Magnetic resonance imaging (MRI) of the brain and orbits demonstrated posterior globe flattening, increased optic nerve sheath diameter (more pronounced in the left eye), and partial empty sella.

The patient was referred to a neurologist consult, with unremarkable neurologic and systemic finding, absence of headache, transient visual obscurations, diplopia, or pulsatile tinnitus, suggested to have a MRI venography that revealed findings compatible with intracranial hypertension, including narrowing of traverse sinuses bilateral and no venous sign thrombosis. Lumbar puncture was not initially performed, and no other treatment was first initiated in this case; therefore, the diagnosis was considered radiologically supported intracranial hypertension rather than confirmed IIH. During follow-up, visual acuity, visual fields, and optic nerve appearance remain stable and at 3 months follow up, the patient being asymptomatic.

A diagnostic lumbar puncture is planned to confirm elevated opening pressure and complete the diagnostic workup. The patient is presently under multidisciplinary investigation and after three months of ophthalmologic surveillance, visual acuity, visual fields, and optic nerve appearance remain stable, the patient being asymptomatic.

**Discussion and Conclusion:** This case illustrates an atypical, asymptomatic presentation of radiologically supported intracranial hypertension detected incidentally during routine ophthalmologic examination in a non-obese young patient. Gradual intracranial pressure elevation may allow partial physiological adaptation, explaining the absence of classic symptoms.This case underscores the importance of careful optic nerve evaluation in routine examinations, particularly in myopic patients where disc morphology may be misleading. Early recognition and close multidisciplinary surveillance are essential to preserve visual function, even in clinically asymptomatic cases.

88. **Early mortality for individuals with idiopathic intracranial hypertension**

K.B. Digre^1^, C. Soule, C. O’Neill^3^, J. Shaia^4^, M. Newman^5^, J. E. A. Warner^1^

^1^Moran Eye Center, Departments of Ophthalmology and Neurology, University of Utah, Salt Lake City, UT, USA; ^2^University of Utah Spencer S. Eccles School of Medicine, Salt Lake City, UT, USA; ^3^University of North Dakota, Grand Forks, ND, USA; ^4^Vanderbilt University, Nashville, TN, USA; ^5^Huntsman Cancer Center, Salt Lake City, UT, USA

**Background**: Pseudotumor cerebri syndrome (PTCS) is divided into primary idiopathic intracranial hypertension (IIH) or secondary (PTC related to medication, venous sinus disease, etc.). All-cause mortality in patients with IIH is reported as increased (1). The purpose of this study is to review the number and causes of death in our patients.

**Methods**: We employed two separate methods. A retrospective review was conducted of our center’s database of 907 patients diagnosed with PTCS between 1999 and 2024. Inclusion criteria were based on established diagnostic criteria for PTCS. Data collection included patient age, sex, age at death, and time between diagnosis and death. Cause of death from death certificates or hospital records was reviewed. We then queried the large Utah Population database of Patients with an ICD code for IIH (ICD9 348.2, ICD10G93.2) who had died and compared to controls who had died and matched 5:1 based on body-mass index, sex, and race. Patients were excluded if they had other causes of increased ICP. Causes of death were also evaluated.

**Results**: In our database, 26 deceased patients, the majority were women. IIH had been diagnosed in 19 patients and PTC in 7. Causes of death included vascular disease (10), cancer (4), external (3), endocrine (2), suicide (2), respiratory disease (1), and hepatic disease (1). 3 deaths were of unknown cause. Average age of death for IIH was 50.4 ± 13.4 years and for PTC was 43.7 ± 16.4 years. Our Patients with IIH are dying 27.1 years earlier and with PTC 33.8 years earlier than the national average of 77.52. Suicide rate in our patients was 0.22 % of IIH patients compared with national average of 0.0142%.

Of the 3,412 IIH patients and 21,512 controls in the database population, 35 IIH patients and 1418 controls were deceased. IIH patients died at an average age of 46.34, had an average BMI of 30.33 (SD 7.56), and were 94% female. Controls had an average age of death 60.48 average BMI of 29.17 (SD 3.95) and were 70% female. Patients with IIH had an increased risk of early mortality at 3.70 (95% CI 2.70, 5.08). The Kaplan Meier curve showed distinct differences by 50 years of age (p < 0.0001). Intentional self-harm had an increased rate among IIH patients compared to controls (p < 0.001) with a risk ratio of 9.16 (95% CI 3.98, 21.1).

**Conclusion**: With both methods we found IIH patients who have died had an earlier mortality rate. This may be due in part to a higher percentage of self-harm/suicidality where IIH patients had a 9.16-fold increased risk compared to controls. Further research is needed to analyze cause of death trends in patients with PTCS, especially the rate of suicide.

ReferenceHermes
SM, et al
Neurology. 2020 Aug 18;95(7):e921-e929. Erratum in: Neurology. 2020 Aug 18;95(7):325.10.1212/WNL.000000000000931232221030

99. **Neuroimaging outcomes in suspected papilledema**

Leon Alexander Danyel

Department of Neurology, Charité Universitätsmedizin Berlin – Campus Virchow Klinikum, Berlin, Germany

**Background and Objectives**: Papilledema (PE) due to elevated intracranial pressure may indicate life-threatening conditions such as intracranial mass lesions or cerebral venous sinus thrombosis. This study evaluated neuroimaging outcomes in patients with suspected papilledema to inform diagnostic pathways in the emergency care setting.

**Methods**: This bicentric, retrospective cohort study included consecutive patients who presented to tertiary care emergency departments with suspected PE between 2009 and 2022. Based on a review of clinical and radiological data, diagnostic outcome was categorized as either “confirmed PE” (Group 1; 1a: idiopathic intracranial hypertension IIH; 1b: space-occupying lesions, hydrocephalus or secondary intracranial hypertension) or “PE ruled out” in patients with optic disc edema unrelated to elevated intracranial pressure or pseudopapilledema. Visual acuity, common symptoms at presentation and focal neurological deficits were compared between groups. Diagnostic yield and number needed to scan (NNS) of CT and MRI to identify PE(1b) was calculated. Finally, univariable logistic regression models were performed to identify clinical predictors of PE(1b).

**Results**: 225 adult patients (43 ± 16.8 years, 57% female) were included in the study. PE was confirmed in 124 of 225 patients (55%; 37 ± 13.8 years, 69% female), with IIH (1a) accounting for 80 (64.5%), secondary causes of PE (1b) for 44 (35.5%) of cases. PE was ruled out in 73 of 225 patients (32.4%; 51 ± 17.4 years, 41% female). Primary CT confirmed PE(1b) in 13.3% (NNS: 7.5), while primary MRI confirmed PE (1b) in 16.7% of patients (NNS: 6). In univariable analysis, diplopia (OR 3.3, 95% CI 1.2–9.2; p = 0.02), nausea/vomiting (OR 3.0, 95% CI 1.2–7.1; p = 0.02), and focal neurological deficits (OR 5.4, 95% CI 2.1–13.5; p < 0.001) were significantly associated with PE(1b).

**Discussion**: Secondary intracranial pathology is common in adults with suspected papilledema. As presenting symptoms have limited discriminatory value for identifying secondary causes, and focal neurologic deficits are absent in the majority of patients, timely cerebral imaging should be performed routinely in all patients.

105. **When BMI misleads: idiopathic intracranial hypertension in a non-obese Filipina but with recent rapid weight gain**

Elinor Angelie M. Perez^1^, Marianne Roque. Carabeo^2^

^1^St Luke’s Medical Center Eye Institute Quezon City Philippines; ^2^St Luke’s Medical Center Eye Institute Quezon City Philippines

**Background**: Idiopathic Intracranial Hypertension (IIH) can happen in patients who are not overweight, especially if they have a history of recent, rapid weight gain. The standard diagnostic criterion for lumbar puncture (LP) opening pressure is more than 25 cmH_2_O. However, IIH can also be diagnosed with pressures between 20 and 25 cmH_2_O if certain signs and symptoms, like papilledema or pulsatile tinnitus, and specific neuroimaging findings of an empty sella, sinus stenosis, posterior globe flattening ang optic nerve sheath distention are present.

IIH is typically seen in obese females of childbearing age.

**Case Report**: A 33 year year-old Filipino female with a body mass index (BMI) of 20.4 presented with a 2-month history of recurrent episodes of visual obscurations (TVO) left more frequent than the right, left-sided headache, retro-orbital pain, and left-sided tinnitus. She had a history of Isotretinoin use 1 year prior to the onset of the headaches but was already discontinued 2 months prior to having symptoms. She had a 7Kg intentional weight gain in a span of less than 6 months. Her visual acuity is 20/20 both eyes, normal Ishihara scores, mild abduction deficit bilaterally, with Frisen 3 edema OD and Frisen 4 OS. Neuroimaging (MRI with contrast) and venography were genrally unremarkable, except for posterior globe flattening and mild prominence of the CSF spaces within the optic nerve sheath complexes bilaterally. Notably, lumbar puncture (LP) opening pressure was 21 cmH2O with normal csf constituents. The patient reported improvement of headache after LP and resolution of the TVOs. Her blood works are all negative. Given the combination of symptoms, clinical findings, and supportive findings on imaging, a diagnosis of IIH was made. The patient was started on acetazolamide, with noted improvement of symptoms and serial OCT findings upon follow-up. She was in remission after 5 months after losing 7Kg body weight returning back to being 49Kg.

**Discussion and Conclusion**: Although IIH typically affects obese women of childbearing age, it can also occur in the non-obese population. IIH patients may also rarely present with low to normal CSF pressure, which may reflect a negative fluctuation at that timepoint only, but not accurately reflect the actual pressure. Moreover, Research shows that sudden weight gain plays an important role in IIH, with studies finding that gaining about 5–15% of body weight within a year can increase risk even if BMI remains in the normal range. IIH can also occur with normal or low-normal spinal fluid pressure, particularly in thin, non-obese individuals; when symptoms such as headaches, vision changes, or ringing in the ears are present along with typical MRI findings, this presentation is considered a rare variant of the condition. In non-obese patients, IIH often looks different overall, tending to occur at a younger age and being associated with more severe optic nerve swelling and more frequent, persistent, and disabling headaches compared with obese patients.

ReferencesDaniels et al., *Neurology*, 2007 — **non-obese individuals with recent weight gain are at increased risk**Chen et al., *Curr Neurol Neurosci Rep*, 2014 — **5–15% weight gain sufficient to raise IIH risk**Horev et al., 2024
— confirmed **normal-weight IIH cohorts**

106. **Multidisciplinary management of pediatric papilledema using lumboperitoneal shunting**

Mariantonietta Di Brina^1^, Giacomo Maria Bacci^2^, Barbara Spacca^3^, Regina Mura^4^, Antonio Laborante^5^

^1^Casa Sollievo della Sofferenza, San Giovanni Rotondo, Italy; ^2^Meyer Children Hospital, Florence, Italy; ^3^Azienda ospedaliera universitaria Meyer - IRCCS, Firenze, Italy; ^4^Azienda ospedaliera universitaria Meyer - IRCCS, Firenze, Italy; ^5^Casa Sollievo della Sofferenza, San Giovanni Rotondo, Italy

**Background**: There is a paucity of literature evaluating the effectiveness of lumboperitoneal shunt (LPS) in pediatric patients with pseudotumor cerebri (PTC), particularly regarding objective assessment of papilledema through ophthalmoscopy and optical coherence tomography (OCT) of the peripapillary retinal nerve fiber layer (pRNFL).

**Aim**: To evaluate the efficacy of LPS in the management of PTC-associated papilledema in children, with short- and long-term follow-up.

**Methods**: Eight pediatric patients (4 females, 4 males) with PTC underwent pre- and post-LPS assessment, including visual acuity (logMAR), ophthalmoscopic papilledema grading (Frisen scale), and OCT measurement of pRNFL thickness. Follow-up intervals were individualized according to clinical individual characteristics.

**Results**: Eight pediatric patients (16 eyes; mean age 9.25 years, with a median of 9 years (IQR 7.5–11) were included. All had bilateral papilledema at presentation, and none showed venous sinus thrombosis on MR venography. All patients received acetazolamide, and five also received corticosteroids before surgery. After LPS, mean peripapillary retinal nerve fiber layer (pRNFL) thickness significantly decreased from 178.9 ± 60.1 µm to 116.9 ± 48.5 µm (*p* < 0.001), and mean Frisen grade improved from 2.94 ± 1.29 to 0.88 ± 0.62 (*p* < 0.001). Best corrected visual acuity remained stable (0.07 ± 0.12 vs. 0.03 ± 0.08 logMAR, *p* = 0.105). No patient developed complications such as intracranial hypotension or infection during follow-up.

**Conclusion**: Lumboperitoneal shunting is an effective and safe intervention for pediatric PTC, leading to rapid reduction in intracranial pressure, pRNFL thickness, and papilledema severity. Long-term outcomes indicate preserved visual function and optic nerve integrity.

110. **Optical coherence tomography detection of shunt malfunction and recurrent raised intracranial pressure in an asymptomatic child with bilateral optic atrophy**

Martina Suzani^1^, Andrea Trezza^1^, Giorgio Carrabba^1^, Carlo Giussani^1^, Michele Coppola^1^

^1^Department of Ophthalmology, IRCCS San Gerardo Hospital, Monza, Italy

**Introduction**: Papilledema is a common sign of increased intracranial pressure(ICP) and, in patients with ventriculoperitoneal(VP) shunts, its presence often guides surgical management. However, when optic atrophy develops, the detection of fundoscopic changes becomes challenging, especially in asymptomatic patients.

**Case Report**: An 11-year-old boy with a history of congenital post-hemorrhagic hydrocephalus treated with ventriculoperitoneal shunt placement at birth presented to the emergency department complaining of headache. Visual acuity (VA) was normal in both eyes. Fundoscopic examination revealed bilateral atrophic optic discs. Optical coherence tomography(OCT) demonstrated swelling of the retinal nerve fiber layer(RNFL), and posterior deformation of the peripapillary Bruch’s membrane.

Computed tomography(CT) scan and magnetic resonance imaging(MRI) did not show signs of raised ICP. Due to the presence of headache at night and in the morning and the OCT findings, the patient underwent shunt revision under general anesthesia, which revealed shunt malfunction. After shunt replacement surgery, OCT parameters decreased and headache resolved.

Regular comprehensive neuro-ophthalmological examination every three months was performed, including assessment of visual fields, optic nerve head OCT (ONH-OCT), and RNFL OCT. After 12 months, RNFL and ONH-OCT thickness values increased again, despite the absence of symptoms suggestive of raised ICP. Neuroimaging remained unremarkable. Due to the high suspicion of shunt malfunction based on progressive worsening of the OCT findings, surgical exploration was performed and confirmed shunt malfunction, with subsequent shunt replacement. OCT measurements decreased shortly after surgery.

Monthly neuro-ophthalmological follow-up was instituted to allow early detection of OCT changes. Progressive thickening of the RNFL and ONH-OCT was observed over time. Although the patient remained asymptomatic, intraoperative evaluation again demonstrated shunt malfunction, requiring further revision. Seven months later, OCT once more showed increased RNFL and ONH-OCT thickness. Because of reported mild headache and repetitive vomiting, a further shunt revision surgery was performed. Intraoperative findings demonstrate sub-occlusion of the proximal catheter. A new VP shunt was placed. OCT values normalized after surgery and remined stable since.

**Discussion**: This case highlights the value of OCT as a noninvasive and objective tool for monitoring ICP in patients with optic atrophy, when fundoscopy assessment is unreliable, and particularly in the absence of symptoms. Recurrent RNFL swelling detected by OCT consistently indicated raised ICP and was confirmed at each surgical shunt evaluation. It is well known in pediatric population that symptoms could be absent or not specific. Moreover, in children with a VP shunt since birth, shunt malfunction could not show a related radiological worsening. Early OCT-based identification of shunt malfunction could be a reliable tool to guide surgical intervention in selected cases, preventing worsening of the neurological condition and further optic nerve damage with irreversible visual loss.

116. **What are the benefits of using acetazolamide in Idiopathic Intracranial Hypertension? Insights from a subanalysis of the IIH:Weight Trial**

Michael Lowe^1^; Yousef F Hyder^2,3^; Andreas Yiangou^2,3^; James Mitchell^2,3,4^; Kristian Brock^5^; Susan P Mollan^2,6^; Alexandra J Sinclair^2,3^

^1^Birmingham Neuro-ophthalmology, Queen Elizabeth Hospital, University Hospitals Birmingham NHS Foundation Trust, Birmingham, UK; ^2^Translational Brain Science, Department of Metabolism and Systems Science, College of Medicine and Health, University of Birmingham, Birmingham, UK; ^3^Department of Neurology, Queen Elizabeth Hospital, University Hospitals Birmingham NHS Foundation Trust, Birmingham, UK; ^4^Department of Military Rehabilitation, Defense Medical Rehabilitation Centre, Loughborough, UK; ^5^Cancer Research Clinical Trials Unit, University of Birmingham, Birmingham, UK; ^6^Ophthalmology Department, Queen’s University, Kingston, Ontario, Canada

**Introduction**: Acetazolamide is a commonly used treatment in idiopathic intracranial hypertension (IIH), supported by the results of the IIH Treatment Trial (IIH-TT) demonstrating improved visual outcomes. However, patients may have difficulty tolerating adverse effects. The IIH Weight Trial (IIH-WT) was designed to assess the efficacy of weight management interventions in IIH, demonstrating a correlation between weight loss and intracranial pressure (ICP) and a significant benefit of bariatric surgery. Some patients in this trial were using acetazolamide; we performed a sub-analysis to determine if use of acetazolamide influenced outcomes in this study.

**Methods**: Participants in the IIH-WT (n=66) were randomised to bariatric surgery (n=30) or CWI (n=36). Randomisation was stratified for use of acetazolamide, but acetazolamide use and dose, at enrolment and during the study, was determined by the treating physician, not influenced by study protocol. The primary outcome was ICP (measured by lumbar puncture opening pressure). Secondary outcomes included weight, visual measures, headache measures and quality of life measures. We employed hierarchical regression to analyse outcomes, corrected for baseline differences, between patients taking (n=19) or not taking acetazolamide (n=47). Most participants in the bariatric surgery group stopped acetazolamide by 1 year. A second analysis was therefore performed for the CWI group alone.

**Results**: There were no significant differences in weight loss or ICP with acetazolamide use.

With respect to visual outcomes, there was a significant within-group improvement in perimetric mean deviation (PMD) from baseline to 12 (+1.1±0.4dB, mean±SE p=0.009) and 24 (+1.9±0.5dB, p<0.001) months in participants not taking acetazolamide in the whole cohort analysis. A similar significant improvement was seen in the CWI group analysis. A within-group improvement was not seen in the acetazolamide group.

Headache diary data showed significant within-group reduction in monthly headache days (MHDs) in the acetazolamide group from baseline to 12 and 24 months in the whole cohort and CWI analyses. A significant between-group difference was found favouring acetazolamide at both timepoints (-6.2±3 MHDs at 12 months; -7.5±3.1 at 24 months) in the whole cohort analysis. A similar trend, though not reaching significance, was seen in the CWI analysis.

**Discussion**: Acetazolamide use did not influence weight loss or ICP in the IIH-WT. Surprisingly, participants managed ‘conservatively’ (CWI, no acetazolamide) had significant improvement in PMD whilst participants taking acetazolamide did not. However, there were improvements in headache outcomes with acetazolamide. These results contrast those of the IIH-TT, where improvements in PMD, but not headache outcomes was seen with acetazolamide. This should be interpreted with caution given the small numbers in this exploratory sub-analysis where acetazolamide allocation was not randomised, and lower doses used than in the IIH-TT. Our results do support that some patients with IIH can be managed conservatively (i.e. without medication) without poorer outcomes.

**Disclosures**: ML reports speaker fees (Pfizer); travel (Abbvie, Teva, Lundbeck). SM reports consultancy fees (Invex Therapeutics); advisory board fees (Ocular therapeutix, Dompe); speaker fees (Teva); Travel (Abbvie and European Alliance of Associations for Rheumatology); Receipt of equipment (Heidelberg Engineering). AJS reports she has consulted for Novartis, AbbVie, Vertex and Orion Pharma and speaker fees from Teva Uk and Novartis on unrelated topics.

This clinical trial was funded by grant NIHR-CS-011-028 (clinician scientist fellowship) from the National Institute for Health Research (Dr Sinclair) and grant MR/K015184/1 from the Medical Research Council of the UK (Dr Sinclair). AJS was supported by a Sir Jules Thorn Award for Biomedical Research.

133. **What is vision threatening papilloedema?**

Rebecca Rewbury^1^, Palaniappan Muthiah^1^, Irene Pepper^1^, Simon Hickman^1^ and Joanna Jefferis^1^.

^1^Sheffield Teaching Hospitals, Glossop Road, Sheffield, UK.

**Introduction**: There is currently no evidence-based consensus regarding the timing or type of surgical intervention in idiopathic intracranial hypertension (IIH). Severe papilloedema, when defined by a peri-papillary retinal nerve fibre layer thickness (RNFLT) >400µm on optical coherence tomography (OCT), has been associated with poorer visual outcomes. This study aimed to examine the management and visual outcomes of IIH patients with severe papilloedema (RNFLT >400 µm), to explore how we define sight-threatening disease.

**Methods**: This retrospective study included patients diagnosed with IIH at a single UK tertiary referral centre between 2016 and 2025. Patients were included if RNFLT exceeded 400µm in one or both eyes at any time point in the course of their disease and if follow-up from this time point was at least four weeks. Visual acuity, RNFLT, total retinal thickness (TRT), macular ganglion cell layer volume (GCLV), and area of the I4e isopter on semi-automated kinetic (Octopus) perimetry were recorded at baseline and at most recent follow-up. For each patient, the eye with the highest RNFLT was analysed. Statistical analysis was performed on SPSS, with parametric and non-parametric tests applied as appropriate.

**Results**: Thirty-seven patients (36 female, 1 male) met the inclusion criteria. Twenty-one (56.8%) underwent bilateral optic nerve sheath fenestration (ONSF) and sixteen (43.2%) were managed medically. One patient required a cerebrospinal fluid diversion procedure 8 months after ONSF. Median follow-up was 57 weeks (range 4 weeks – 8 years).

Baseline visual acuity and visual field area were worse in the surgically managed group, although differences were not statistically significant. Baseline RNFLT and TRT were similar between groups. Baseline GCLV was lower in the surgical group (1.05mm^3^ vs 1.17mm^3^, p=0.05). At final follow-up, visual acuity and I4e isopter area improved significantly across the cohort, with no statistically significant difference in the degree of improvement between surgical and medical management. Reductions in RNFLT and TRT were also comparable between groups. The surgically managed group demonstrated a greater decline in GCL volume (-0.23 mm^3^ vs -0.09 mm^3^, p=0.05).

**Discussion**: The results indicate that medical management can be considered in patients with severe optic disc swelling if they have good visual function. There are multiple factors to consider in the management of potentially sight threatening IIH and a biomarker would be a useful tool in risk stratifying patients. These data suggest that RNFLT alone is not sufficient to categorise papilloedema as vision-threatening and therefore in need of surgical intervention.

**Disclosures**: The authors have no disclosures to declare.

148. **Ultrasound measurement of optic nerve sheath diameter for the purpose of evaluating elevated intracranial pressure with or without papilledema–Case series**

Tamara Mišljenović Vučerić ^1,2^, Ana Srdoč ^2^, Tomislav Vidović ^3^, Jelena Metikoš ^4^, Tea Čaljkušić Mance^2^

^1^TIM oftalmologija, Rijeka, Croatia; ^2^Clinical Hospital Canter Rijeka, Department of Ophthalmology, Rijeka, Croatia; ^3^University Hospital Center Zagreb, Department of Ophthalmology, Reference center for neuro ophthalmology, Zagreb, Croatia; ^4^Clinical Hospital Center Osijek, Department of Ophthalmology, Osijek, Croatia

**Introduction:** Ultrasound measurement of optic nerve sheath diameter (ONSD) is becoming an increasingly popular non-invasive method for assessing elevated intracranial pressure (ICP). This method is used in clinical practice for a quick and simple evaluation of patients with suspected elevated ICP, whether papilledema is present or not. The optic nerve sheath is directly connected to the subarachnoid space, which allows changes in ICP to be reflected on its diameter, before the development of papilledema.

**Case report:** This is a case series report of several patients where, by ultrasound measurement of the optic nerve sheath diameter, raised intracranial pressure was suspected or the effect of treatment was monitored.

Patient diagnoses in our case series included: IIH (idiopathic intracranial hypertension), sec. increased intracranial pressure due to a brain tumour, long-term diagnosis of normotensive glaucoma.

Elevated intracranial pressure was not presented by papilledema in all patients. Papilledema was evaluated ophthalmoscopically and on OCT (optical coherence tomography).

**Discussion:** Ultrasound measurement of optic nerve sheath diameter is a useful method for rapid and non-invasive assessment of elevated intracranial pressure. Our case series report shows that this technique can be of great value in various clinical scenarios, especially in emergency situations where rapid assessment of ICP is essential for further treatment.

**Disclosures:** Authors have no conflict of interest to declare.

149. **Effect of semaglutide and hypocaloric diet on visual outcomes in idiopathic intracranial hypertension: a longitudinal randomized study**

Sanja Cejvanovic^1,2^, Nadja Skadkær Hansen^2,3^, Elisabeth Arnberg Wibroe^1,2^, Dagmar Beier^4,5^, Rigmor Højland Jensen^2,3^, Steffen Hamann^1,2,6^

^1^Department of Ophthalmology, Copenhagen University Hospital - Rigshospitalet, Glostrup, Denmark; ^2^Department of Clinical Medicine, University of Copenhagen, Copenhagen, Denmark; ^3^ Danish Headache Center, Copenhagen University Hospital – Rigshospitalet-Glostrup, Copenhagen, Denmark; ^4^ Department of Neurology, Odense University Hospital, Odense, Denmark; ^5^Department of Clinical Research, University of Southern Denmark, Odense, Denmark; ^6^ Rothschild Foundation Hospital, Paris, France

**Introduction**: Idiopathic intracranial hypertension (IIH) causes vision threatening papilledema through elevated intracranial pressure (ICP). Standard care includes weight loss and acetazolamide, but treatment response is difficult to monitor as repeated lumbar punctures for ICP measurement are invasive and impractical for longitudinal follow-up. Non-invasive biomarkers that reflect real-time ICP changes are needed to enable reliable treatment monitoring.

Glucagon-like peptide-1 receptor agonists (GLP-1RAs) show promise for IIH management through weight reduction and potential direct ICP-lowering effects. This study evaluates whether the GLP-1RA semaglutide improves visual outcomes when added to standard care in women with IIH. Additionally, we assessed optical coherence tomography (OCT) parameters as non-invasive surrogate markers for ICP, which could enable longitudinal treatment monitoring without repeated invasive measurements.

**Methods**: This study reports pre-specified visual outcomes from an open-label randomized controlled trial comparing standard care alone (control) to an adjunct weight lowering intervention: a *Very Low-Calorie Diet* (VLCD, 800 kcal daily) for 8 weeks combined with weekly semaglutide injection for 10 months (intervention) in adult women with definite IIH (2013 Friedman criteria). We enrolled participants at two Danish sites from November 2022 to December 2024 with 10-month follow-up. Assessed visual outcomes were change in perimetric mean deviation (PMD) and OCT measures of the optic nerve head (ONH) and macula (peripapillary retinal nerve fiber layer (pRNFL) thickness, macular ganglion cell layer (mGCL) volume, ONH volume and ONH thickness) from baseline to 10 months. Correlation of visual outcomes with lumbar puncture opening pressure based ICP levels were also evaluated.

**Results**: Thirty-six women were randomized (semaglutide: n=20; control: n=16). Baseline characteristics were similar between groups for age, BMI, ICP, and most visual parameters (pRNFL thickness, mGCL volume, ONH volume, ONH thickness), except PMD. The semaglutide group had worse baseline PMD (median 4.5 [IQR 2.7-7.8] dB vs 2.8 [1.4-4.2] dB, p=0.001).

After adjusting for baseline imbalances, PMD at 10 months was similar between groups (adjusted difference 0.43 dB; 95% CI, -0.56 to 1.42; p=0.36). OCT parameters showed no significant differences (all p>0.36) between groups at 10 months.

All OCT measures correlated significantly with both ICP and PMD. For ICP, ONH thickness showed the strongest correlation (β=15.2 μm per cm H_2_O, R^2^=0.77, p<0.001), followed by ONH volume (R^2^=0.63), PMD (R^2^=0.65), and pRNFL (R^2^=0.57). OCT structural parameters also correlated significantly with PMD (all p<0.001).

**Discussion**: In patients with IIH, treatment with semaglutide and initial VLCD plus standard care vs. standard care alone resulted in significant and similar visual improvement over 10 months. OCT structural parameters showed comparable improvement in both groups, indicating effective papilledema resolution regardless of treatment strategy. ONH thickness was the parameter that most strongly correlated with ICP, confirming its value as a non-invasive, easily available surrogate ICP-marker.

**Disclosures:** This study (SC) was supported by the Novo Nordisk Foundation (grant NNF200C005926) for one year. Additional unrelated funding was provided by Candys Foundation. The funders had no role in study design, conduct, data collection, management, analysis, interpretation, or manuscript preparation. The authors report no conflicts of interest.

159. **Misdiagnosis in suspected idiopathic intracranial hypertension in Germany**

Theresia Knoche

Department of Neurology, Charité Universitätsmedizin Berlin, Berlin, Germany

**Introduction**: Idiopathic intracranial hypertension (IIH) is a rare disorder with potentially irreversible visual morbidity. Accurate diagnosis requires careful clinical assessment, neuroimaging, lumbar puncture, and ophthalmologic evaluation. Misdiagnosis may lead to unnecessary intracranial pressure (ICP)–lowering treatment and invasive procedures. We aimed to assess the frequency, mechanisms, and consequences of diagnostic error in patients referred with suspected IIH to a specialized outpatient clinic.

**Methods**: We retrospectively reviewed all patients referred to the specialized IIH outpatient clinic at Charité – Universitätsmedizin Berlin between 1 January and 31 December 2025 for suspected IIH or counselling regarding an established diagnosis. Clinical records were analysed for referral patterns, prior diagnostic work-up, ophthalmologic assessments, treatments, and final diagnoses. Diagnoses were reassessed using the modified Friedman criteria (2013). Diagnostic errors were categorized using the Diagnostic Error Evaluation and Research (DEER) taxonomy. Secondary intracranial hypertension was analysed separately.

**Results**: Ninety patients were included (73 female; mean age 36.7 years; mean body mass index 31.3 kg/m^2^). IIH was confirmed in 38 patients (42%), and secondary intracranial hypertension in 6 patients (7%). Twenty-seven patients (30%) had received a prior false-positive diagnosis of IIH, and one patient was classified as false negative.

All false-positive patients had been treated with ICP-lowering medication. Major contributors to diagnostic error included misinterpretation of cerebrospinal fluid opening pressure, absence of venous imaging, and overemphasis on incidental neuroimaging findings. According to DEER taxonomy, errors most frequently occurred during diagnostic assessment and testing phases. Importantly, several patients had not undergone formal ophthalmologic evaluation prior to diagnosis, despite IIH being defined by optic nerve involvement and visual risk.

Five patients were referred for evaluation of cerebrospinal fluid shunting; four did not meet diagnostic criteria, including one patient who had already undergone ventriculoperitoneal shunt placement. Three patients were referred for venous sinus stenting, one without fulfilling IIH criteria. The most frequent alternative diagnoses were migraine and tension-type headache; some patients had no relevant neurological disease.

**Discussion**: Misdiagnosis of IIH was common in this specialized cohort and frequently associated with insufficient ophthalmologic assessment and incomplete application of diagnostic criteria. Given the rarity of IIH and its primary neuro-ophthalmologic complications, diagnosis and management should involve neuro-ophthalmology and be concentrated in experienced centres to reduce diagnostic error and prevent avoidable harm.

**Disclosures**: The authors report no disclosures.

167. **Intracranial hypertension associated with Terson’s syndrome after epidural blood patch: A case report**

Vanessa Takou Tsapmene,^1^ Rim Maalej,^2^ Ana Banc,^1,2^ Dan Milea^1,2,3,4,5^

^1^ Rothschild Computational and Visual Neurosciences Laboratory, Adolphe de Rothschild Foundation Hospital, Paris, France; ^2^ Department of Neuro-Ophthalmology, Adolphe de Rothschild Foundation Hospital, Paris, France; ^3^ Singapore Eye Research Institute, Singapore National Eye Center, Singapore; ^4^ Duke-NUS Medical School, Singapore; ^5^ Copenhagen University, Denmark

**Introduction**: Headache due to intracranial hypotension is a common complication of spinal anesthesia. When conservative management fails, an epidural blood patch (EBP) is indicated. EBP involves the injection of a small volume of autologous blood into the epidural space to seal cerebrospinal fluid leakage. Severe complications are rare.

**Case report**: A 53-year-old woman developed orthostatic headache, nausea, and vomiting following uterine myomectomy under epidural anesthesia. An EBP was performed for suspected iatrogenic intracranial hypotension, during which she experienced syncope. Six hours later, she reported bilateral blurred vision and floaters. Visual acuity was preserved (10/10 OU), while fundus examination revealed bilateral grade IV papilledema associated with peripapillary, intraretinal, and vitreous hemorrhages. Visual field testing showed nasal and inferior loss in the right eye and blind spot enlargement in the left eye. Optical coherence tomography demonstrated bilateral severe, diffuse peripapillary retinal nerve fiber layer thickening and preserved macular ganglion cell layer. Brain MRI and MRV showed indirect signs of intracranial hypertension without venous sinus thrombosis or other vascular abnormalities; spinal MRI revealed leptomeningeal enhancement. Lumbar puncture demonstrated elevated opening pressure (44 cmH_2_O), neutrophilic pleocytosis, and increased protein levels, with no infectious or malignant etiology identified. A final diagnosis of intracranial hypertension associated with Terson’s syndrome following EBP was established. Treatment with acetazolamide led to progressive resolution of papilledema and hemorrhages, with maintained visual function.

**Discussion**: This case highlights a rare complication of EBP, resulting in rebound intracranial hypertension and visual loss due to bilateral papilledema and associated Terson’s syndrome. The papilledema and intraocular hemorrhages may have been the result of sudden elevation of the intracranial pression, impairing venous drainage and raised intravascular pression, leading to rupture of the internal blood-retinal barrier and peripapillary and retinal hemorrhages.

**Disclosures**: The authors declare no conflict of interest.

170. **Neuro-ophthalmological signs of intracranial hypertension in patients undergoing surgical closure of spontaneous cerebrospinal fluid leak**

Youri Ducluzeau^1^, Caroline Froment^1^, Catherine Vignal^2^

^1^Neuro-Ophtalmology Unit, Pierre Wertheimer Hospital, Claude Bernard Lyon University, Lyon, France; ^2^Neuro-Ophtalmology Unit, Fondation Adolphe de Rothschild Hospital, Paris, France.

**Introduction**: Spontaneous cerebrospinal fluid leaks (SCFL) are associated with idiopathic intracranial hypertension (IIH). Some authors reported that intracranial pressure increases again after surgical closure of SCFL. To date, there is no official recommendation for the management of IIH in these cases. Some teams propose post-operative medical or interventional treatments of IIH without necessarily a proper assessment. Almost no studies looked specifically at the neuro-ophthalmological signs in these patients.

**Method**: Between January 2018 and June 2024, we recruited patients who underwent SCFL closure surgery from two expert centres. We collected data from a systematic postoperative neuro-ophthalmological consultation, as well as MRI data when available.

**Results**: 32 patients were included. Only 12 had received a neuro-ophthalmological evaluation preoperatively. 5 patients had already been diagnosed with IIH before surgery. Post-operative consultation delays varied in our cohort (median 22 months, IQR [6; 34]). Postoperatively, we noted 3 patients with Frisen 1 papilledema, none beyond. A quarter of patients had a visual field with at least one deficit but mostly peripheral and always with preserved visual acuity. On Optical Coherence Tomography (OCT), median peripapillary Retinal Nerve Fiber Layer (pRNFL) was 98 µm (IQR [89;104]). Almost all Magnetic Resonance Imaging (MRI) showed at least one indirect sign of IIH. 19 patients received treatment for IIH after surgery. The only significant difference of examination between the 2 subgroups of patients treated or not for IIH concerned the affected visual fields. They were more frequent in the subgroup treated for IIH (p=0.005). We considered that 7 patients showed signs of active IIH postoperatively, and 6 were treated. But 8 other patients, with no argument for active IIH, also received invasive treatments for IIH.

**Discussion**: The low incidence of papilledema was consistent with previous literature. Furthermore, when present, these papilledema were mild. Our OCT and visual fields were usually normal. When they were not, the alterations were only moderate. Conversely, all MRIs showed at least one sign of IIH or several. However, these signs are not specific to IIH, and there is no consensus on how they evolve in relation to intracranial pressure. Therefore, we remind that they are not sufficient for a diagnosis of IIH and we should not propose invasive IIH treatment simply because they are present.

**Conclusion**: Active IIH after surgical closure of SCFL is possible but does not occur in most patients. The neuro-ophthalmological signs are usually mild to moderate. The first aim of this post-operative neuro-ophthalmology consultation is to detect potential visual complications of IIH. The second is to provide a multidisciplinary assessment of IIH, in order to discuss possible invasive treatments after SCFL surgery considering properly the benefit-risk ratio.

OCULAR MOTILITY AND EYELIDS

4. **Pilot data of smooth pursuit measurement for multiple sclerosis patients with bulbicam infrared camera**

Adriana Zelníková ^1,2^, Dominik Foltán^1,2^, Marie Soukupová ^1,2^, Jakub Potměšil^1,2^, Jana Lízrová Preiningerová^1,2^

^1^Department of Neurology, GUH in Prague, Czech Republic; ^2^Department of Neurology, First School of Medicine, Charles University in Prague, Czech Republic

**Introduction**: Oculomotor abnormalities are frequently underrecognized in clinical practice, as their detection relies largely on the examiner’s experience and lacks objective quantification. Among these, square wave jerks (SWJs)—small, involuntary saccadic intrusions disrupting fixation—are common in disorders affecting brainstem and cerebellar pathways. This study aimed to establish and validate a standardized protocol for quantitative assessment of SWJs using a video-oculography Bulbicam infrared camera in patients with Multiple Sclerosis (MS) and to compare findings with healthy controls (HC)

**Methods**: Patients diagnosed with MS according to the 2017 McDonald criteria, aged 18–50 years, were assessed using Bulbicam infrared oculography. Exclusion criteria included best-corrected visual acuity <20/25 in both eyes, severe refractive error (>10 D myopia or >4 D hyperopia), recent MS relapse (<3 months), or inability to cooperate during the examination. An age-matched healthy control (HC) cohort was also evaluated. Several acquisition protocols were tested, and a short-duration fixation protocol (≤30 seconds) was selected to minimize the effects of fatigue and attention deficits. The protocol explored different definitions of SWJ events, defined as amplitude peaks in the velocity curve of smooth pursuit at varying velocity thresholds (50, 75, and 100 deg/25 msec). The number of velocity peaks was used to calculate the SWJ50, SWJ75, and SWJ100 scores.

**Results**: Preliminary analysis included 40 participants—patients with relapsing-remitting MS and healthy controls. Patients with MS demonstrated significantly higher SWJ50 and SWJ75 scores compared with HC. In the HC cohort, SWJ scores increased with age. Currently, no standardized methodology or normative reference data exist for quantifying tracking quality or SWJ frequency in MS. These findings highlight the need to develop robust, age-specific normative datasets to enable meaningful clinical interpretation of SWJ metrics.

**Discussion**: The increased frequency of SWJs in patients with MS likely reflects subtle impairment of cerebellar and brainstem circuits responsible for gaze stabilization—regions commonly affected by demyelination. These oculomotor abnormalities represent a functional disturbance that is often undetectable during standard clinical or imaging evaluations, yet may correspond to early or diffuse infratentorial involvement. Importantly, the presence of infratentorial lesions in MS has been identified as a negative prognostic factor, associated with more severe disability and faster disease progression. As conventional MRI is limited in detecting small or functionally significant lesions in these regions, quantifying SWJs through infrared oculography provides a sensitive and noninvasive means of detecting such subclinical pathology. Therefore, SWJ analysis may serve as a functional biomarker of infratentorial involvement, offering potential clinical value for prognosis, disease monitoring, and early therapeutic decision-making in MS.

**Disclosures**: The research is supported by funds from the Grant Agency of Charles University in Prague, Czech Republic (GAUK) (586225), institutional support of the research from GUH in Prague MH CZ–DRO–VFN64165 and *Cooperatio Program in Neurosciences*, Charles University.

11. **Two new forms of hemi-seesaw nystagmus: Associated with homonymous hemianopia and paroxysmal with infratentorial graviceptive pathway sparing**

Alexandre Roldão Alferes^1^, Daniela Pereira^2^, Catarina Rato^3^, Gonçalo Costa^4^, João Nabais^4^, Armando Lopes^4^, Ana Inês Martins^1,5^, André Jorge^1,5^, João Lemos^1,5^

^1^Neurology Department, Coimbra Local Health Unit (ULS Coimbra), Coimbra, Portugal; ^2^Neuroradiology Department, Medical Imaging Service, Coimbra Local Health Unit (ULS Coimbra), Coimbra, Portugal.; ^3^Otorhinolaryngology Department, Coimbra Local Health Unit (ULS Coimbra), Coimbra, Portugal; ^4^Neurosurgery Department, Coimbra Local Health Unit (ULS Coimbra), Coimbra, Portugal; ^5^ Faculty of Medicine of the University of Coimbra, Coimbra, Portugal.

**Introduction:** Seesaw nystagmus (SSN) and hemi-seesaw nystagmus (HSSN) constitute forms of continuous vertico-torsional pendular and jerk nystagmus, respectively. These have been reported in patients with bitemporal hemianopias and patients with brainstem lesions affecting the graviceptive pathways. SSN seems to mostly reflect miscalibration of retinal error signals usually sent to the visuovestibular apparatus via the accessory optic system (AOS), while HSSN mostly arises from interruption of the graviceptive pathways. We present two new forms of HSSN: paroxysmal and associated with homonymous hemianopia.

**Case Report:** Case 1. A 46-year-old male with a history of craniopharyngioma presented with bilateral visual loss and vertical diplopia for 2 years. He had best corrected visual acuity (BCVA) 20/16 OD, 20/25 OS, a left relative afferent pupillary defect, and left optic atrophy. Perimetry showed dense bitemporal hemianopias together with a central and superior defect in the left eye. Every minute he developed paroxysmal ~30s jerk HSSN (upper poles beating towards the left, right eye beating up, left eye beating down) superimposing in baseline vertical strabismus (let eye hypertropic) and spontaneous right beating nystagmus.

Case 2. A 32-year-old male with a longstanding history of right parietal arteriovenous malformation presented with BCVA 20/16 OD, 20/12.5 OS. Perimetry showed a dense left homonymous hemianopia. Additionally, there was continuous pendular HSSN. Importantly, infratentorial graviceptive pathways were spared in both cases.

**Discussion:** Pendular SSN has only been described in bitemporal hemianopia while Jerk HSSN has only been described in brainstem lesions. SSN seems to mostly reflect miscalibration of retinal error signals usually sent to the visuo-vestibular apparatus via the AOS, while HSSN mostly arises from interruption of the graviceptive pathways. In case 1, the sparing of infratentorial graviceptive pathways suggests that higher-order visual or visuo-vestibular mechanisms may play a greater role than previously thought in jerk HSSN. In case 2, the observation of a strictly hemicycle of pendular HSSN in the context of homonymous hemianopia expands SSN topographic associations beyond bitemporal visual field defects, suggesting that an asymmetric impairment of the cortical or subcortical visual motion processing regions related to the AOS, might generate an asymmetric form of pendular SSN.

**Disclosures:** The authors have nothing to disclose.

16. **The natural course and prognostic factors of microvascular ocular motor palsies**

André Sobral-Pinho^1,2^, André Costa^3^, Ana Inês Martins^4,5^, Daniela Pereira^5,6^, André Jorge^4,5^, João Lemos^4,5^

^1^Neurology Department, Lisboa Ocidental Local Health Unit, Lisbon, Portugal; ^2^NOVA Medical School, Universidade Nova de Lisboa, Lisbon, Portugal; ^3^Neurology Department, Trás-os-Montes e Alto Douro Local Health Unit, Vila Real, Portugal; ^4^ Neurology Department, Coimbra Local Health Unit, Coimbra, Portugal; ^5^ Faculty of Medicine, Coimbra University, Coimbra, Portugal; ^6^ Neuroradiology Department, Coimbra Local Health Unit, Coimbra, Portugal

**Introduction**: Microvascular ocular motor palsies (MOMP) result from an ischemic insult of the third (3NP), fourth (4NP) or sixth cranial nerve (6NP). While underlying vascular risk factors are established causes of MOMP, the natural course and prognostic factors of MOMP deserve further study.

**Methods**: Observational, retrospective cohort study based on the review of clinical and paraclinical data from adult patients referred to our neuro-ophthalmology clinic, between April 2023 to April 2024, with a final diagnosis of MOMP. Patients were categorized into 3NP, 4NP and 6NP groups in order to evaluate the natural course of MOMP and identify associated prognostic factors. Demographic and clinical variables were compared between groups using Kruskal-Wallis test and further correlated with MOMP duration using Spearman’s test. A p value < 0.05 was considered significant.

**Results**: We included 89 (3NP, 19; 4NP, 22; 6NP, 48) age-matched patients. Associated ipsilateral periorbital pain was present in 42 (47.2%) patients and was equally present between groups (p=0.383). Diabetes was near-significantly less common in 4NP than 3NP (p=0.069), dyslipidemia was significantly less common in 6NP than in 3NP (p=0.046), while hypertension was similarly prevalent between groups (p=0.205). Diplopia duration was similar between groups (81.4+/-55 days; p=0.932). Additionally, diplopia duration was positively correlated with erythrocyte sedimentation rate (ESR) and C-reactive protein (CRP) levels (R= 0.640, p=0.014; R=0.613, p=0.020) in 3NP, and with abduction excursion (R=0.364, p=0.019) and hematocrit levels (R=0.320, p=0.044) in 6NP. The degree of ocular misalignment (prism diopters and/or duction excursion) at presentation was not a prognostic factor of 4NP or 3NP duration. Only 6 (7%) patients remained with diplopia after 6 months. One 6NP patient underwent surgery, and one other received a temporary prism.

**Discussion**: MOMP show a favorable prognosis, with complete recovery rates exceeding 90% in all groups at 6 months. Inflammatory biomarkers at MOMP presentation seem to have a role in the prognosis of microvascular 3NPs, while the severity of paresis at MOMP presentation seems to be particularly useful in determining 6NPs prognosis. In 3NP and 4NP, a precise quantification of paresis severity is usually more difficult to achieve, which may explain why no significant correlations with diplopia duration were observed. The latter findings, together with a trend for discrepant prevalence of individual vascular risk factors in each palsy, suggests that microvascular damage might entail different mechanisms for 3NP, 4NP and 6NP.

**Disclosures**: The authors report no disclosures.

19. **Strabismus surgery is an effective option for patients with abducens palsy caused by neurovascular conflict**

Annabel Groot

Radboud University Medical Center, Nijmegen, the Netherlands

Neurovascular compression of the abducens nerve is a rare but increasingly recognized cause of slowly progressive horizontal diplopia and esotropia. It typically occurs at the root-exit zone, where the abducens nerve is most vulnerable to pulsatile compression from the basilar, vertebral, or AICA arteries, occasionally associated with dolichoectasia on high-resolution MRI. Reported symptoms often evolve over years, beginning with intermittent diplopia and mild abduction deficits that gradually progress to constant misalignment. While observation, blood-pressure control, prism glasses and microvascular decompression have all been described, evidence for durable improvement is limited, and only one isolated report exists on strabismus surgery as treatment.

I present three patients with neurovascular compression–related abducens palsy who declined neurosurgical decompression and underwent strabismus surgery. Procedures included medial rectus recession, lateral rectus resection, and, in one severe case, Hummelsheim transposition with Foster augmentation. All patients achieved satisfactory postoperative alignment, with resolution of diplopia in primary gaze.

These cases demonstrate that strabismus surgery offers an effective, lower-risk therapeutic alternative to neurosurgical intervention, particularly in patients with long-standing symptoms or limited abduction, though long-term stability requires further study.

20. **Diagnosis and management of myasthenia in a neuro-ophthalmology service**

Ayman Khaier^1^, Muzzammil Hussain^1^

^1^Department of Ophthalmology, Queen’s Hospital, Barking Havering and Redbridge NHS University Trust, Romford, England

We share our experience in the diagnosis and management of myasthenia patients in our institution. All patients presenting to our neuro-ophthalmology service and diagnosed with myasthenia gravis between 2020 and 2025 were reviewed, to evaluate the clinical patterns in diagnosis, symptom onset, and treatment response.

Eighty-six patients were suspected with MG on first presentation, and 52 had the diagnosis confirmed. Majority of referrals (83%) came from community optometrists and general practitioners, and 17% from neurology.

The highest prevalence of MG was observed in the 61-80 years age group (34.6%), and the rest distributed across other age groups. MG seemed more prevalent in younger females (<40 years) but older males (>60 years).

Most common symptoms on presentation where diplopia (86.5%), Ptosis (84.6%), or a combination (75%). Bulbar or skeletal symptoms were seen in 5.8% and 32.6% respectively. Average duration from symptom onset to diagnosis was 8 months.

Almost a third (30.7%) of patients were diagnosed as generalised MG on final analysis, but only 9.6% were diagnosed as general, having started with pure ophthalmic symptoms.

AChR antibodies were detected in 60% of cases, and MuSK was positive MG in 5.8%. 35% of patients were seronegative. sfEMG was done in 25 patients, of whom 20 were positive (80%).

Positive response to Pyridostigmine at initiation was 58%, but on final analysis only 27% had pyridostigmine as monotherapy. Steroids were used in 37% of the cases; and 42% were maintained on dual or triple therapy. On final review, all treated patients remained on treatment.

**Conclusion:** Referrals from non-specialist sources had longer delays in diagnosis compared with median. However, delays of diagnosis seem also affected by the delayed confirmation by tests. We discuss the correlation of diagnostic tests with patient subgroups. Early referral to neurology/neuro-ophthalmology optimises management and shortens delays. Faster access to electromyography and antibody testing correlated with improved diagnosis outcomes.

41. **Topical neostigmine ophthalmic solution for alleviating myasthenic ptosis: A phase 1 dose-finding study**

Chuthamas Ongprakobkul^1^, Supanut Apinyawasisuk^1^, Supharat Jariyakosol^1^, Pajaree Chariyavilaskul^2^, Kornvalee Meesilpavikkai^3^, Yuda Chongpison^4^

^1^Department of Ophthalmology, Faculty of Medicine, Chulalongkorn University and Ophthalmology Department, King Chulalongkorn Memorial Hospital, The Thai Red Cross Society, Bangkok, Thailand; ^2^Center of Excellence in Clinical Pharmacokinetics and Pharmacogenomics, Department of Pharmacology, Faculty of Medicine, Chulalongkorn University, Bangkok, Thailand; ^3^Department of Microbiology, Faculty of Medicine, Chulalongkorn University, Bangkok, Thailand; ^4^Biostatistics Excellence Center, Research Affairs and Center of Excellence for Skin and Allergy Research, Faculty of Medicine, Chulalongkorn University, Bangkok, Thailand

**Introduction**: The study aimed to investigate the safety, optimal biologic dose, and preliminary efficacy of topical neostigmine ophthalmic solution (TNOS) in patients with myasthenia gravis (MG) presenting with ptosis, as well as the sterility of the TNOS formulation.

**Methods**: The TNOS samples were cultured for microbial detection. A phase 1 dose-finding study was then conducted, enrolling a cohort of three MG patients with ptosis at each step. The first cohort received 1.5 mg/mL TNOS, which was identified as the pharmacologically active dose in our preclinical animal models. Vital signs, visual acuity, intraocular pressure, pupillary sizes, slit-lamp examinations, and abnormal ocular symptoms were assessed as safety measures. The vertical palpebral fissure height over time and self-reported alleviation of ptosis were assessed as efficacy measures. After completing these assessments, subsequent cohorts received the selected dose of TNOS in accordance with the safety and efficacy protocols to determine the optimal biologic dose.

**Results**: No systemic or ocular adverse effects were observed in any participants. Vertical palpebral fissure height increased by more than 2 mm in 83.3% and 33.3% of participants receiving 1.5 mg/mL and 1.0 mg/mL TNOS, respectively. A significant increase in mean vertical palpebral fissure height compared to baseline was observed in the 1.5 mg/mL group (maximal mean difference 3.30 mm; 95% confidence interval 1.44 to 5.16; *p* = 0.005). All participants in the 1.5 mg/mL group and 33.3% in the 1.0 mg/mL group reported alleviation of ptosis. No microorganisms were detected in any TNOS samples.

**Discussion**: The 1.5 mg/mL TNOS was considered the optimal biologic dose for relieving myasthenic ptosis without adverse events. This study enhanced the possibility of developing TNOS as a novel diagnostic tool or local ocular therapy for MG with fewer side effects than systemic medications.

**Disclosures**: This study was supported by the 90^th^ Anniversary of Chulalongkorn University Scholarship, Ratchadapiseksompotch Fund and Ratchadapiseksompotch Fund, Faculty of Medicine, Chulalongkorn University, grant RA67/030. None of the authors has any conflicts of interest to disclose.

66. **Facial neuropathy in patients with recurrent painful ophthalmoplegic neuropathy**

Jae-Myung Kim,^1^ Kyung Wook Kang,^1^ Kang-Ho Choi,^1^ Dong Uk Kim^2^, Seung-Han Lee^1^

^1^Department of Neurology, Chonnam National University Hospital and Chonnam National University Medical School, Gwangju, South Korea; ^2^Malgeunmeori Neurology Clinic, Gwangju, South Korea

**Introduction**: Involvement of non–ocular motor cranial nerves, such as the trigeminal, facial, or hypoglossal nerves, has rarely been reported in recurrent painful ophthalmoplegic neuropathy (RPON). This study aimed to determine the incidence and clinical significance of multi–cranial nerve involvement in RPON.

**Methods**: Eight patients (females = 5; mean age = 53.3 ± 15.6 years) who met ICHD-3 diagnostic criteria for RPON were enrolled at a single tertiary hospital. Repeated evaluations were performed for differential diagnosis, including cranial nerve examination, brain MRI, cerebrospinal fluid analysis, and serologic testing for autoimmune and tumor markers. Clinical features, neuroimaging findings, and laboratory results were reviewed.

**Results**: Five patients (62.5%) had a present or past history of migraine. Among 25 ophthalmoplegic attacks, the abducens nerve was most frequently affected (60%), followed by the oculomotor nerve (32%). Most attacks were preceded by ipsilateral headache (4.0 ± 2.9 days; 88%). Facial nerve involvement was common (7/8, 87.5%); two patients had three recurrent facial neuropathies on the same side, and one showed simultaneous ipsilateral oculomotor and facial neuropathies. All facial neuropathies were peripheral. Of three patients who underwent MRI for facial neuropathy, two demonstrated focal facial nerve enhancement. Postauricular pain usually preceded facial neuropathy and lacked migrainous features. Both facial neuropathy and ophthalmoplegia resolved within days to weeks.

**Discussion**: Facial neuropathy may be more common in RPON than previously recognized. Despite similarities to Bell’s palsy, our findings support considering facial neuropathy within the RPON spectrum and suggest that recurrent, multifocal, and alternating cranial neuropathy may underlie its pathophysiology.

**Disclosures**: None.

77. **Peculiarities of stereoacuity and eye micromovements during a static fixation task in children aged 6 to 13 years with retinopathy of prematurity after laser photocoagulation and without it**

Iryna Boichuk^1^, Oleksandr Artamonov^1^, Serhiy Katsan^1^

^1^State Institution “V.P. Filatov Institute of Eye Diseases and Tissue Therapy of the National Academy of Medical Sciences of Ukraine”, Odesa, Ukraine

**Purpose**: To reveal peculiarities of patterns, including saccades and fixations in premature children with retinopathy of prematurity who underwent laser coagulation and in children without signs of retinopathy, and full-term children with emmetropia, during a static fixation task using high-resolution eye-tracking technology who achieved 6–10 y. o.

**Methods**: A total of 125 children participated in the study. Eye movement recordings were performed using the EyeLink 1000 Plus eye tracker (SR Research Ltd., Canada) at a sampling rate of 2000 Hz. Participants completed a static fixation task, during which they were instructed to maintain their gaze on a central circular target for 10 seconds without any change in the stimulus location. The experiment was designed using Experiment Builder software, and the recorded data were analyzed with Data Viewer. Binocular vision was defined by Worth Test, Stereovision thresholds were determined using the Lang II test and the Titmus Stereo fly test.

**Results**: It was found that disorders of conjugate eye movements and saccades were statistically significantly more often (75%) in the group of children after LC than in the group of full-term children (χ2 =33,18, p = 0,0000 …), (χ2 =4,24, p=0,003) 26,9% respectively. These findings were confirmed with recorded eye-movements by eye-tracking technology. The fixation retention test revealed significant intergroup differences in two parameters. The mean saccade amplitude was significantly lower in children with retinopathy of prematurity after laser coagulation (2.72°) compared to the control group of full-term children with emmetropia (4.61°; p = 0.030). Similarly, the mean pupil diameter was smaller in the laser-treated group (1269 μm) compared to emmetropic controls (1987 μm; p < 0.001).

Premature infants without PH had normal stereoacuity by Titmus Stereofly test in 76,2% of cases, and by the test Lang II in 80,4% of children. Only two could not define stereovision (3.8%). In premature children who underwent LK 38,8% of children did not show stereoscopic thresholds using the Titmus Stereofly test and the test Lang II; in 26.8% of children, stereoscopic thresholds were normal.

**Conclusion**: The study showed that children with retinopathy of prematurity after laser coagulation had reduced mean saccade amplitude (2.72° vs. 4.61°) and smaller pupil diameter (1269 μm vs. 1987 μm) compared with full-term emmetropic controls. These findings indicate long-term visual system alterations affecting fixation control, visual acuity and stereovision. The results allow us to recommend eye exercises to develop motility, as well as exercises to develop binocular and stereoscopic vision for children who have undergone LC for RP as early as possible.

87. **Saccadic dysmetria and delayed latency induced by severe globe deformity following encircling scleral buckling: a correlation of mri and video-oculography findings**

Ileok Jung

Neurology, Konkuk University Medical Center, Seoul, South Korea

**Purpose:** Saccadic abnormalities such as delayed latency and hypometria are typically associated with central nervous system pathologies. However, mechanical restriction in the orbit can mimic these neurological deficits. We report a case of a patient presenting with gaze-evoked amaurosis and asymmetric saccadic movements following retinal detachment surgery, demonstrating that severe globe deformity caused by a tight encircling scleral buckle can induce significant alterations in ocular motor dynamics.

**Methods:** (Case Report): A 37-year-old female presented with transient visual obscuration (gaze-evoked amaurosis), oscillopsia, and metamorphopsia in her left eye upon eye movement or head tilting. She had a history of retinal detachment repair via pars plana vitrectomy and encircling scleral buckling (using #276 tire and #240 band) in the left eye. To investigate the pathophysiology, we performed high-resolution Orbit MRI and quantitative saccadic recording (Video-oculography).

**Results:** Orbit MRI revealed a severe “hourglass” deformity of the left globe, characterized by marked equatorial constriction and axial elongation due to the encircling band. The buckling element was visualized indenting the globe beneath the rectus muscles, creating a mechanical fulcrum. Saccadic analysis showed significant asymmetry between the eyes. While the right eye exhibited normal saccadic metrics, the left eye demonstrated marked hypometria and delayed saccadic latency (increased reaction time), particularly during abduction and adduction. These findings correlate with the “viscous drag” and “tethering effect” caused by the physical indentation of the buckle, acting as a “speed bump” for the extraocular muscles.

**Conclusions:** This case illustrates that saccadic dysmetria and latency prolongation can result from purely mechanical mechanisms rather than neurological deficits. The structural deformity induced by a tight encircling buckle alters the length-tension relationship and pulley mechanics of the extraocular muscles.

96. **Bilateral third nerve palsy as the initial manifestation of disseminated tuberculosis with central nervous system involvement**

Laura Vigués-Jorba^1^, Ruth Martin Pujol^1^, Laura Sanchez Vela^1^

^1^Hospital Universitari Vall d’Hebron, Barcelona, Spain

**Introduction**: Central nervous system tuberculosis may present as tuberculous meningitis or intracranial tuberculomas, leading to neuro-ophthalmologic manifestations such as visual loss or cranial nerve palsies. The most frequent oculomotor nerve involvement is sixth nerve palsy secondary to elevated intracranial pressure, whereas third nerve palsy is less common. Bilateral third nerve involvement is rare and may result from basilar meningitis or direct nerve inflammation. We report a case of disseminated tuberculosis presenting with bilateral third nerve palsy.

**Case Report**: A 31-year-old man from Senegal, residing in Spain and with no relevant medical history, presented with fever and headache following a recent visit to his home country. He progressively developed right upper limb weakness, left ptosis, and diplopia, prompting hospital admission.

Neuro-ophthalmologic examination revealed a best-corrected visual acuity of 0.8 in the right eye and 0.4 in the left eye. Left ptosis, mydriasis, and a large-angle exotropia exceeding 100 prism diopters were observed. Ocular motility examination showed moderate limitation of depression in the right eye and severe limitation of adduction and depression, with mild limitation of elevation in the left eye, consistent with bilateral third cranial nerve involvement. Anterior segment examination and funduscopy were unremarkable.

Brain magnetic resonance imaging demonstrated three intra-axial lesions located in the left cerebral peduncle/mesencephalon, right parasagittal parietal cortex, and left parasagittal occipital cortex, with heterogeneous enhancement and central necrosis. Imaging also revealed neuritis of the intracisternal segments of both oculomotor nerves and partial thrombosis of the left transverse sinus and posterior superior sagittal sinus. Thoracoabdominal computed tomography showed axillary and retroperitoneal lymphadenopathy and lytic vertebral lesions involving L5 and S1.

Axillary lymph node biopsy confirmed necrotizing granulomatous lymphadenitis with Langhans giant cells, and mycobacterial culture was positive, establishing the diagnosis of disseminated tuberculosis. Treatment was initiated with a four-drug antituberculous regimen, dexamethasone, and anticoagulation therapy. Follow-up magnetic resonance imaging one month later showed a reduction in lesion size.

After nine months of treatment, the patient reported subjective improvement in extraocular motility. Visual acuity improved to 1.0 in the right eye and 0.6 in the left eye. Ocular motility examination showed partial recovery, although significant limitation of adduction and depression in the left eye persisted, along with a large-angle exotropia, mydriasis, and ptosis. Upper limb strength recovered after intensive rehabilitation.

**Discussion**: This case illustrates that disseminated tuberculosis may present with bilateral oculomotor nerve palsy as the predominant neuro-ophthalmologic manifestation, even in the absence of pulmonary symptoms. The bilateral but asymmetric involvement suggests basilar meningitis with inflammation of the intracisternal segments of the oculomotor nerves, supported by neuroimaging findings. Partial neurological recovery after treatment highlights the importance of early recognition and prompt initiation of antituberculous therapy and corticosteroids in patients from tuberculosis-endemic regions presenting with atypical cranial neuropathies.

**Disclosures**: The authors report no conflicts of interest.

107. **Occam meets Hickam: Dual genetic diagnoses in a patient with ataxia and preserved vision**

Marius Bertram Maartensson^1^, Dina Abdelsalam^2^, Safa Ibrahim^2^, Andrew Go Lee^2^

^1^Department of Ophthalmology, Copenhagen University Hospital – Rigshospitalet, Copenhagen, Denmark; ^2^Department of Ophthalmology, Blanton Eye Institute, Houston Methodist Hospital, Houston, TX, USA

**Introduction**: Leber hereditary optic neuropathy (LHON) is a mitochondrial disorder most often associated with subacute central vision loss, though heteroplasmy may result in incomplete or atypical phenotypes. LHON can be representing with extra-ocular features such as ataxia named LHON plus.

Spinocerebellar ataxia type 27B (SCA27B), caused by a GAA repeat expansion in the FGF14 gene, is a recently recognized autosomal dominant disorder characterized by adult-onset gait ataxia and downbeat nystagmus.

We report a case illustrating the evolving nature of neurogenetic diagnostics and the coexistence of two distinct genetic disorders in neuro-ophthalmology.

**Case Report**: A 63-year-old man presented with progressive intermittent non-vertiginous dizziness lasting seconds to minutes, recurrent falls, and difficulty tracking his golf ball in 2019. Past medical history included Hodgkin’s lymphoma in total remission and a dilated aortic root followed by cardiology.

Neuro-ophthalmic examination showed visual acuity of 20/20 in both eyes, normal color vision, no relative afferent pupillary defect, and downbeat nystagmus in lateral and downgaze. Visual fields showed no defects and optical coherence tomography was unremarkable except a mild papillomaculo bundle dropout in his right eye. Extensive metabolic, autoimmune, paraneoplastic blood workup and magnetic resonance imaging were unremarkable. A comprehensive ataxia workup, including testing for common spinocerebellar ataxias, Friedreich ataxia, and cerebellar ataxia with neuropathy and vestibular areflexia syndrome, was negative.

Whole genome sequencing was also performed and identified a pathogenic mitochondrial DNA mutation (m.11778G>A in MT-ND4) in a heteroplasmic state of 52.5 %, consistent with LHON.

Family history was notable for multiple neurologically affected relatives. His older brother exhibited intermittent leg numbness, slurred speech, imbalance, and multiple falls and upon testing he carried the same mutation with approximately 55 % heteroplasmy.

His sister reported balance difficulties and reading problems, however she didn’t have LHON. Her adult daughter developed balance impairment. The patient’s late mother developed late-onset gait disturbance.

Interestingly, the patient, who is very informed about his LHON disease, went to a mitochondrial conference recently. This resulted in a courage to pursue a new genome sequencing because of the recent discovery of SCA27B presented at the conference.

This revealed a pathogenic GAA repeat expansion in FGF14 (357 repeats), confirming a diagnosis of SCA27B in 2025.

**Discussion**:This case highlights the complex, ever-evolving nature of neuro-genetics anchoring in neuro-ophthalmology, particularly when earlier genetic testing predates newly recognized diseases. While LHON may present with variable penetrance and extra-ocular features, the identification of SCA27B provided a more coherent explanation for the patient’s downbeat nystagmus and late-onset neurological symptoms.

The coexistence of mitochondrial and nuclear genetic disorders supports the long-standing battle between Hickam’s dictum versus Occam’s razor and underscores the rapidly evolving field of neurogenetic diagnostics.

To our knowledge, this represents the first reported case of coexisting LHON and SCA27B.

**Disclosures**: The authors declare no conflicts of interest.

111. **Ocular motor characteristics of infantile nystagmus syndrome in clinical practice using standardized eye movement recordings**

Colin Colmant^1^, Ana Coito^2^, Mathias Abegg^1^

^1^University of Zurich, Switzerland; ^2^machineMD AG, Bern, Switzerland

**Introduction**: Nystagmus diagnosis and clinical management are still largely based on bedside observation and subjective interpretation of eye movements, sometimes supported by magnification using Frenzel goggles. However, the ocular motor characteristics of nystagmus provide critical diagnostic information, allowing differentiation between benign physiological forms and acquired patterns that may indicate serious neurological disease. Although experimental eye movement recording techniques have demonstrated the value of quantitative waveform analysis for classification and pathophysiological understanding, such tools are rarely available in routine clinical practice. Until recently no certified medical device allowed an assessment of all clinically relevant nystagmus features. The recently introduced neos® system enables standardised recordings across gaze positions, smooth pursuit, and convergence tasks within routine care. This study aims to describe the frequency of established ocular motor characteristics of infantile nystagmus syndrome (INS) and to explore their ability to differentiate INS from other forms of nystagmus in a clinical cohort.

**Methods**: This retrospective, single centre, consecutive, cross-sectional observational case series included all patients with a documented clinical diagnosis of nystagmus of any subtype who had at least one available neos® eye movement recording obtained during routine clinical care between June 2022 and January 2026, and a written informed consent. A comparator group without nystagmus was identified from individuals who underwent neos recordings in routine care without a clinical diagnosis of nystagmus. Recordings were analysed in coded form by blinded raters who were unaware of clinical information. Predefined ocular motor characteristics of INS included: presence of nystagmus, conjugacy, presence of a null zone, accelerating slow phase, convergence dampening, and pursuit-related changes.

**Results**: Patients with INS showed a characteristic pattern of ocular motor findings. The most frequent features included horizontal but not vertical slow-phase drifts, the presence of a gaze-dependent null zone, identification of accelerating slow phases, associated conjugate horizontal movements, and dampening of nystagmus during convergence, in decreasing order of frequency. The combined features reliably distinguished INS from other nystagmus forms.

**Conclusions**: Standardized automated eye movement recordings obtained with a commercially available device allow an objective and quantitative documentation of all clinically relevant INS characteristics that are impossible to quantify by observation alone. The combined features allow a reliable diagnosis of INS. This type of nystagmography may be useful in quantifying nystagmus to monitor treatment efficacy.

**Disclosures**: Mathias Abegg and Ana Coito are employees and shareholders of the company machineMD, which manufactures neos®.

112. **Acquired fourth nerve palsy after cervical spinal manipulation: A case report**

Matteo Baldi^1^, Michele Iester^1^, Aldo Vagge^1^

^1^University Eye Clinic of Genoa, Department of Neuroscience, Rehabilitation, Ophthalmology, Genetics, Maternal and Child Health (DiNOGMI), University of Genoa - IRCCS “San Martino” Polyclinic Hospital - Genova, Italy

**Introduction**: Cervical spinal manipulation is widely used for the management of neck pain and stiffness. Although serious neurological complications are considered rare, a variety of neuro-ophthalmological adverse events have been reported, most commonly in association with vascular involvement of the posterior circulation. To our knowledge, isolated acquired fourth cranial nerve palsy in temporal association with cervical spinal manipulation has not been well documented in the literature.

**Case Report**: A 65-year-old man presented with acute vertical binocular diplopia and blurred vision occurring a few hours after cervical spinal manipulation performed for neck pain and stiffness that had been present for several days. The patient reported that the procedure involved a rapid, passive movement of the neck with combined rotation and extension, similar to maneuvers he had previously undergone for recurrent cervical pain. Neurological examination revealed no additional focal deficits. Computed tomography of the brain was unremarkable. High-resolution diffusion-weighted magnetic resonance imaging (MRI) and computed tomography angiography were performed three days after symptom onset and showed no acute ischemic lesions or arterial dissection; however, a fusiform dilation of the proximal basilar artery consistent with vertebrobasilar dolichoectasia was noted.Ophthalmological examination demonstrated left hypertropia increasing in right gaze and left head tilt, mild limitation of infraduction of the left eye in adduction, inferior oblique overaction, objective excyclotorsion, and reduced vertical fusional amplitudes, consistent with an isolated acquired left fourth nerve palsy. Conservative management was adopted. The patient reported that diplopia persisted for several weeks after onset and then gradually improved over subsequent months, with complete resolution achieved within five months. At six-month follow-up, ocular alignment was normal with no residual diplopia.

**Discussion**: This case illustrates a close temporal association between cervical spinal manipulation and isolated acquired fourth nerve palsy. The presence of vertebrobasilar dolichoectasia may represent a predisposing vascular factor, potentially increasing susceptibility to transient hemodynamic disturbances during cervical rotation and extension. The absence of diffusion restriction on MRI does not exclude a transient ischemic or hypoperfusion-related mechanism, particularly when imaging is performed in the subacute phase. The progressive spontaneous recovery observed is consistent with the natural history of acquired microvascular fourth nerve palsy and supports an initial conservative management approach when appropriate. While a causal relationship cannot be established, this case underscores the need for clinical vigilance regarding rare neuro-ophthalmological complications following cervical spinal manipulation, especially in patients with underlying vascular anomalies.

**Disclosures**: The authors report no conflicts of interest.

117. **Unilateral third cranial nerve palsy as a complication of MADSAM: A case report**

Maja Kostic^1^, Luciana Garcia^1^, Michael Miltich^2^, Elizabeth Colvin^2^

^1^Bascom Palmer Eye Institute, FL, USA; ^2^Duke University, Durham, NC, USA; ^3^UTHealth Houston, TX, USA

**Introduction**: Multifocal acquired demyelinating sensory and motor neuropathy (MADSAM) is an atypical variant of chronic inflammatory demyelinating polyneuropathy (CIDP) characterized by asymmetric sensorimotor deficits and multifocal demyelination in nerve trunks. While cranial nerve involvement occurs in approximately 48% of MADSAM cases, it is typically unilateral and most commonly affects facial and bulbar nerves. We report a case of MADSAM complicated by isolated unilateral third cranial nerve palsy.

**Case Description**: A 63-year-old male with type 2 diabetes presented in 2024 with a 5-year history of progressive asymmetric sensory symptoms affecting bilateral hands (D1-3 distribution) and burning foot pain. Electromyography demonstrated non-length-dependent sensorimotor polyneuropathy with demyelinating features. Lumbar puncture revealed elevated protein (107 mg/dL) with normal cell count. Serum studies showed markedly elevated anti-SGPG IgM antibodies (14,000; normal 3,000). MADSAM was diagnosed and intravenous immunoglobulin (IVIG) therapy initiated with initial clinical improvement.

In December 2025, following irregular IVIG administration, the patient developed acute-onset left eye ptosis and blurred vision. Examination revealed complete left third cranial nerve palsy without pupillary involvement. Comprehensive stroke workup including MRI/MRA brain was negative for acute infarction, mass lesion, or vascular abnormality. Ophthalmologic evaluation demonstrated complete visual field loss in the left eye with superior paracentral temporal scotoma, retinal nerve fiber layer thinning, and microhemorrhages. Following resumption of regular IVIG therapy (4-day course in January 2026), ptosis resolved with persistent diplopia and head tilt at follow-up.

**Conclusion**: This case illustrates that cranial nerve involvement in MADSAM can manifest as isolated oculomotor palsy, expanding the recognized spectrum of cranial neuropathies in this condition. The temporal relationship between IVIG interruption and symptom onset, with subsequent improvement following treatment resumption, suggests that consistent immunomodulatory therapy may be critical in preventing cranial nerve complications in MADSAM. Clinicians should maintain high suspicion for cranial nerve involvement in MADSAM patients, particularly during treatment interruptions.

References1.Different Electrophysiological Profiles and Treatment Response in ‘Typical’ and ‘Atypical’ Chronic Inflammatory Demyelinating Polyneuropathy. Kuwabara S, Isose S, Mori M, et al. Journal of Neurology, Neurosurgery, and Psychiatry. 2015;86(10):1054–9. doi:10.1136/jnnp-2014-308452.254244352.Chronic Inflammatory Demyelinating Polyneuropathy. Kuwabara S, Misawa S. Advances in Experimental Medicine and Biology. 2019;1190:333–343. doi:10.1007/978-981-32-9636-7_21.317606543.Cranial Nerve Involvement in Typical and Atypical Chronic Inflammatory Demyelinating Polyneuropathies. Shibuya
K, Tsuneyama
A, Misawa
S, et al. European Journal of Neurology. 2020;27(12):2658–2661. doi:10.1111/ene.14497.32876980
4.Sonographic Multifocal Cranial Nerve Enlargement in Multifocal Acquired Demyelinating Sensory and Motor Neuropathy. Oka
Y, Tsukita
K, Tsuzaki
K, Takamatsu
N, Hamano
T.
Internal Medicine
(Tokyo, Japan). 2021;60(17):2867–2871. doi:10.2169/internalmedicine.6782-20.33746164
PMC84792185.Intravenous Immunoglobulin for Chronic Inflammatory Demyelinating Polyradiculoneuropathy. Bus SR, de Haan RJ, Vermeulen
M, van Schaik IN, Eftimov
F.
The Cochrane Database of Systematic Reviews. 2024;2:CD001797. doi:10.1002/14651858.CD001797.pub4.PMC10865446383533016.Progress in Diagnosis and Treatment of Chronic Inflammatory Demyelinating Polyradiculoneuropathy. Bunschoten C, Jacobs BC, Van den Bergh PYK, Cornblath DR, van Doorn PA. The Lancet. Neurology. 2019;18(8):784–794. doi:10.1016/S1474-4422(19)30144-31076244

130. **A sixth nerve palsy with too many friends**

Misha Pless^1^, Manisha Kotay^1^, Eric Eggenberger^1^

^1^Mayo Clinic, FL, USA

**History & Exam**: A 65-year-old man with hypertension and long standing diabetes presented with acute sub-occipital headache followed by binocular horizontal diplopia. Examination revealed an isolated right abduction deficit without other neurologic deficits. Initial MRI brain was unremarkable. He was initially diagnosed with diabetic sixth nerve palsy (CN6P). Two weeks later, he developed numbness in the right V1–V2 distribution, prompting repeat orbital MRI, which demonstrated enhancement in all branches of the right trigeminal nerve, segmental involvement of the facial nerve, and bilateral vidian nerves, without contiguous mass or brainstem involvement. Laboratory evaluation including ESR, CRP, ANA, and Lyme serologies was negative. PET/CT skull-to-thigh showed no FDG-avid systemic disease. Empiric prednisone 60mg daily partially improved headache, but sequential cranial nerve involvement persisted. Bone marrow biopsy and cerebrospinal fluid studies confirmed primary diffuse large B-cell CNS lymphoma. Post-intervention MRI demonstrated interval decrease in cranial nerve enhancement, with residual mild enhancement along the left V2 division and vidian nerves. Post-treatment neuro-ophthalmic examination revealed 20/20 visual acuity OU, 2-4 prism diopter alternating esotropia corrected with Fresnel prisms, and residual right facial dysesthesias. Headaches resolved.

**Case Summary with References**: Painful, sequential cranial neuropathies initially misdiagnosed as ischemic CN6P revealed underlying primary CNS lymphoma. Initial imaging may be normal (1) which can lead to inaccurate diagnosis. Here we see a diabetic abducens mimicker. The case fit so well that without further progression the diagnosis would have been delayed or missed. In a short time span this patient showed new cranial nerve deficits. Progression should prompt repeated and more in-depth workup (2). Early diagnosis and treatment is imperative to prevent morbidity and mortality (2). Corticosteroids provided partial symptomatic relief, but definitive chemotherapy (MATRix and R-CHOP) stabilized disease. Functional outcomes were favorable with preserved vision and prism-corrected binocular alignment.

**Struggle/Dilemma of the Clinical Presentation Description**: The challenge was distinguishing a benign microvascular sixth nerve palsy from an infiltrative process. Initial normal imaging and isolated CN VI involvement suggested a vascular etiology. Sequential involvement of multiple cranial nerves, particularly painful V1–V2 neuropathy, prompted repeat imaging and biopsy. The dilemma highlights that painful, multifocal cranial neuropathies—even when initial studies are unrevealing—require careful localization and targeted evaluation. Early recognition is essential to avoid diagnostic delay and initiate appropriate therapy.

**Describe why your presentation is unique**: This case presented as a diabetic abducens might and imaging was obtained but unrevealing. The progression of lesions involving multiple cranial nerves lead to further workup and eventual diagnosis that was life saving.

**Teaching Points**

1) Red flags to cranial neuropathies: Painful, multifocal, sequential

2) Imaging can be unrevealing early in the course

References1.Low
J, Sharma
A, Lin
YH, Romba
M, Mrugala
M.
When the Imaging is No Help – A Rare Presentation of Primary CNS Lymphoma (P3.163). *Neurology*. 2017;88(16_supplement). doi:10.1212/wnl.88.16_supplement.p3.1632.Watane
GV, Pandya
SP, Atre
ID, Kothari
FN.
Multiple hypertrophic relapsing remitting cranial neuropathies as an initial presentation of primary CNS lymphoma without any brain or spinal cord lesion. Indian J Radiol Imaging. 2016
Jan-Mar;26(1):135–9. doi: 10.4103/0971-3026.178364. PMID: 27081238; PMCID: PMC4813064.27081238
PMC48130643.von Baumgarten L, Illerhaus
G, Korfel
A, Schlegel
U, Deckert
M, Dreyling
M.
The Diagnosis and Treatment of Primary CNS Lymphoma. Dtsch Arztebl Int. 2018 Jun
22;115(25):419–426. doi: 10.3238/arztebl.2018.0419. PMID: 29999484; PMCID: PMC6056710.29999484
PMC6056710

144. **Marcus-Gunn jaw winking ptosis – One can only laugh for so long …**

Grzegorz Rotuski

Military Institute of Aviation Medicine, Warsaw, Poland

Marcus-Gunn phenomenon, also known as jaw-winking syndrome, is a disease primarily from a psychosocial perspective. It results from abnormal connections between branches of the trigeminal nerve and the oculomotor nerve, which reach the pterygoid and levator palpebrae superioris muscles respectively, causing eyelid elevation during jaw movement. While this condition does not generally pose a threat to vision or health, it can lead to discrimination or ridicule among peers. We present three cases of adult patients who decided to seek surgical treatment at different stages of their lives.

158. **Positional opsoclonus in ataxia telangiectasia-like disorder type 1**

Teresa Santana^1^, Francisca Ferreira^2^, Ana Inês Martins^3,4^, João Durães^3^, André Jorge^3,4^, João Lemos^3,4^

^1^Neurology Department, Garcia de Orta Hospital, Almada, Portugal; ^2^Neurology Department, São João University Hospital Centre, Porto, Portugal; ^3^Neurology Department, Coimbra University Hospital Centre, Coimbra, Portugal; ^4^Faculty of Medicine, Coimbra University, Coimbra, Portugal

**Introduction**: Opsoclonus consists of saccadic oscillations without an intersaccadic interval, along the horizontal, vertical and torsional planes, commonly observed in straight head position. Although its pathophysiology is not fully understood, it is thought to involve cerebellar and brainstem dysfunction, with impaired inhibition of omnipause neurons and burst neurons in pons and midbrain. Positional forms are exceptionally rare, with transient vertical opsoclonus described only in healthy neonates. We report strictly positional opsoclonus in a patient with ataxia telangiectasia-like disorder (ATLD) type 1.

**Case Report**: A 22-year-old woman with genetically confirmed ATLD type 1 (MRE11A homozygous mutation) presented with long-standing gait ataxia and dysarthria. Ocular motor exam revealed frequent saccadic intrusions, gaze-evoked nystagmus, decomposed pursuit, delayed, hypometric and slow saccades, deficient head impulse, ocular counterroll disinhibition, and concomitant esophoria. During positional testing, there was persistent apogeotropic positional nystagmus in the Pagnini-McClure position and reproducible bursts of opsoclonus in head hanging position and less consistently in the Dix-Hallpike position. Head impulses and voluntary saccades did not precipitate additional opsoclonus. Brain MRI showed pancerebellar atrophy.

**Discussion**: ATLD is an autosomal recessive chromosomal instability syndrome with prominent oculomotor involvement, saccadic dysfunction being usually an early feature and abnormalities in nystagmus, smooth pursuit and VOR emerging later. Opsoclonus exclusively triggered by head positioning has not been reported before and might reflect transient fastigial nuclei shutdown, apparently precipitated by interaction with nodular-uvular network, ultimately activating the brainstem saccadic circuitry.

**Disclosures**: The authors report no disclosures.

**NEURODEGENERATIVE AND SYSTEMIC DISEASES**

32. **Neuro-ophthalmic manifestations of systemic lupus erythematosus: Spectrum, mechanisms, and management**

Matteo Capobianco^1,2^, Caterina Gagliano^3^, Mark Zeppieri^4^

^1^Università degli Studi di Catania, Catania, Italy; ^2^Università Vita-Salute San Raffaele Milano, Milan, Italy; ^3^Department of Medicine and Surgery, University of Enna “Kore”, Mediterranean Foundation “G.B. Morgagni”, Enna, Italy; ^4^Department of Ophthalmology, University Hospital of Udine and Department of Medicine, Surgery and Health Sciences, University of Trieste, Trieste, Italy

**Introduction**: Systemic lupus erythematosus (SLE) may involve the central and peripheral visual pathways, causing vision-threatening complications that reflect high systemic activity.

Key neuro-ophthalmic entities include optic neuritis, cranial neuropathies, idiopathic intracranial hypertension (IIH), lupus retinopathy, and lupus choroidopathy.

**Methods**: We performed a narrative synthesis of peer-reviewed clinical studies, case series, and contemporary reviews (2000–2025) retrieved from PubMed, Scopus, and Web of Science.

Search terms included “SLE optic neuritis,” “lupus retinopathy,” “lupus choroidopathy,” “pseudotumor cerebri SLE,” and “neuro-ophthalmic lupus,” and reporting followed good-practice guidance for reviews.

Outcomes summarized were prevalence, clinical features, imaging findings, pathophysiology, and therapeutic approaches.

**Results**: Optic neuritis in SLE is uncommon but often severe, frequently bilateral or rapidly sequential, and may result from demyelination or ischemia secondary to small-vessel vasculitis or prothrombotic states.

Cranial neuropathies (particularly VI and VII) occur in active disease via microangiopathy or immune-mediated neuritis.

IIH-like presentations have been reported in association with systemic inflammation and, in a subset, venous outflow impairment or antiphospholipid antibodies, with headache, papilledema, and raised cerebrospinal fluid opening pressure.

Lupus retinopathy ranges from subclinical cotton-wool spots and intraretinal hemorrhages to occlusive vasculitis with arterial or venous occlusion, often correlating with disease flare and antiphospholipid antibodies.

Optical coherence tomography (OCT), OCT-angiography, and fluorescein angiography assist in detecting macular edema, ischemia, and vascular leakage, including subclinical microvascular changes on OCT-A.

Lupus choroidopathy presents with serous retinal detachments and indocyanine-green angiographic hypoperfusion, frequently in the context of nephritis or hypertensive crisis, reflecting immune-mediated and hemodynamic mechanisms.

**Discussion**: Across entities, early recognition and mechanism-guided therapy are crucial.

High-dose systemic corticosteroids are first-line for inflammatory presentations, with adjunctive immunosuppression (e.g., cyclophosphamide or mycophenolate) for severe disease.

Anticoagulation is recommended when antiphospholipid syndrome or retinal/venous thrombosis is documented.

IIH management in SLE includes acetazolamide and corticosteroids (plus CSF diversion when indicated), after exclusion of venous sinus thrombosis.

Adjunctive ocular interventions (laser, anti-VEGF) may be considered for ischemic or edematous complications, but systemic disease control remains central.

Multidisciplinary management and targeted imaging (MRI brain/orbits, OCT/OCT-A, fluorescein/ICG angiography) help prevent irreversible visual loss.

**Disclosures**: None.

50. **VEXAS – The neuro-ophthalmologic chameleon syndrome, a case report.**

Daniela Suter-Starosta

University Eye Hospital Basel, Switzerland

**Introduction**: Vacuoles, E1-enzyme, X-linked, Autoinflammatory, Somatic (VEXAS)-Syndrome is a recently described, adult-onset autoinflammatory syndrome involving systemic inflammation and blood abnormalities. The aetiology of the condition is attributed to a somatic mutation of the E1 ubiquitin conjugating enzyme (UBA1) gene. The general manifestations comprise vasculitis, arthritis, chrondritis, and dermatosis, in addition to haematological abnormalities, such as thrombosis, cytopenia, and vacuolation of bone marrow precursors. A notable heterogeneity has been observed in the previously reported ophthalmic manifestations, encompassing orbital inflammation, dacryoadenitis, myositis, uveitis, scleritis, episcleritis, and periorbital oedema.

**Case Report**: The following case is hereby reported: a 78-year-old male patient initially presented at the clinic in question with symptoms including transient and painless diplopia, scalp pain, fever and elevated C-reactive protein (CRP) levels. The initial suspected diagnosis was giant cell arteritis (GCA), and treatment with oral steroids was initiated on an empirical basis. However, the unremarkable results of the eye examination (normal fundus examination, normal motility, symmetric but habitual exophthalmos, pseudophakia), the absence of the halo sign in Doppler ultrasonography, the absence of stenosing fibrosis of the intima in the temporal artery biopsy, and the absence of signs of cerebral vasculitis in magnetic resonance imaging (MRI) ruled out the GCA. The MRI revealed an inspecific mild pachymeningeal contrast enhancement and Positron Emission Computer Tomography (PET/CT) revealed a consolidated lung mass.

The diagnosis was Legionnaires’ disease, treated with antibiotics. Two weeks later, the patient had returned with diplopia, painless exophthalmos, ptosis, elevation and adduction deficit, redness of the right eye. Corrected visual acuity was symmetrically 20/20. The pupil reaction was intact, with no relative afferent pupil deficit (RAPD). The right eye fundus exam showed a small parapapillary haemorrhage but ruled out papilloedema. The MRI scan revealed retroseptal orbital inflammation, accompanied by mild swelling of the rectus inferior and -lateral muscles. Intravenous antibiotic treatment was initiated to address a possible infection. However, the patient’s symptoms deteriorated within the next few days, with a decrease in visual acuity, an increase in conjunctival congestion and inflammation, lagophthalmos, corneal decompensation and papilledema. A conjunctival biopsy ruled out lymphoma or infection, showing only unspecific inflammation. This led to the initiation of oral steroids, which improved the ophthalmic symptoms within a few weeks.

Two years after the initial presentation, the patient returned with binocular diplopia and redness of the left eye and in the meantime with the genetically confirmed diagnosis of VEXAS Syndrome.

**Discussion**: Ophthalmologists must consider the possibility of autoinflammatory syndromes as a potential cause of various neuro-ophthalmological symptoms. The recently characterised VEXAS syndrome should be considered in elderly male patients with concomitant haematological disorders. Early anti-inflammatory treatment with steroids should be initiated in view of the risk of sight-threatening disease.

Daniela Suter-Starosta, University Eye Hospital Basel, Switzerland

**Disclosures:** Advisory Board for Amgen Switzerland.

61. **Immune-mediated multisystem disease mimicking Vogt–Koyanagi–Harada: Bilateral vision loss with granulomatous uveitis, cerebrovascular infarctions, and arrhythmia revealing hodgkin lymphoma**

Tugce Kizilay^1^˒^2^, Rabia Karabiyik^1^, Medine Asli Yildirim^3^, Kiymet Kasapoglu^4^, Nurhan Kaya^1^, Nilufer Kale^1^

^1^Department of Neurology, University of Health Sciences, Bagcilar Training and Research Hospital, Istanbul, Turkey; ^2^Department of Neurology, University of Health Sciences, Sehit Dr. Ilhan Varank Sancaktepe Training and Research Hospital, Istanbul, Turkey; ^3^Department of Ophthalmology, University of Health Sciences, Bagcilar Training and Research Hospital, Istanbul, Turkey; ^4^Department of Ophthalmology, University of Health Sciences, Haseki Training and Research Hospital, Istanbul, Turkey

**Introduction**: Bilateral granulomatous uveitis accompanied by visual loss has a broad differential diagnosis including infectious, inflammatory, and neoplastic conditions. Systemic malignancies may present as masquerade syndromes with ocular and neurological involvement, leading to diagnostic delay. We present a complex case that was initially suspected to be tuberculous uveitis or Vogt–Koyanagi–Harada (VKH) disease, but was later found to be associated with Hodgkin lymphoma.

**Case Report**: The patient had a history of high myopia and was followed for progressive visual deterioration over several years. Four years prior to presentation, bilateral granulomatous uveitis was diagnosed, at which time best-corrected visual acuity was recorded as 0.4 in the right eye and counting fingers at 2 meters in the left eye. Given a history of tuberculosis exposure, presumed ocular tuberculosis was considered and anti-tuberculous therapy was administered; however, no clinical response was achieved.

Two years prior to presentation, following the development of acute visual loss, brain magnetic resonance imaging revealed bilateral acute parieto-occipital infarctions. Digital subtraction angiography (DSA) demonstrated sequelae of a previous basilar artery thrombosis, left vertebral artery stenosis, and additional structural changes within the vertebrobasilar circulation. Cardiac evaluation revealed frequent ventricular extrasystoles and episodes of ventricular tachycardia, and the patient underwent multiple ablation procedures.

At presentation, neuro-ophthalmologic examination revealed a visual acuity of 0.16 in the right eye and hand motion in the left eye. Slit-lamp examination showed granulomatous keratic precipitates, Koeppe nodules, and posterior synechiae. Fundus examination demonstrated myopic degenerative changes, vitreous degeneration, peripapillary atrophy, and optic disc pallor. Although the interferon-gamma release assay (IGRA) was positive, no clinical or laboratory findings supporting active infection were identified.

The diagnostic turning point was the detection of a nasopharyngeal mass and bilateral cervical lymphadenopathy. Excisional biopsy findings were consistent with nodular sclerosis–type classical Hodgkin lymphoma.

**Discussion**: This case highlights the “great imitator” nature of Hodgkin lymphoma. The uveitis initially attributed to tuberculosis or VKH disease was most likely related to paraneoplastic, immune-mediated inflammation. Occipital infarctions and cardiac arrhythmias may be explained by malignancy-associated hypercoagulability, systemic inflammation, and possible infiltrative myocardial involvement. Visual loss resulted from the combined effects of high myopia, chronic granulomatous uveitis, optic atrophy, vitreous degeneration, and cortical visual impairment secondary to occipital infarctions. Hematologic malignancies should be considered in patients with treatment-resistant uveitis accompanied by unexplained cerebrovascular events.

**Disclosures**: No financial support. No conflicts of interest.A large language model–based artificial intelligence tool was used to improve linguistic clarity and academic fluency of this abstract. The content was reviewed by the authors, who retain full responsibility for the final version.

63. **Orbital Inflammation in a Patient with Common Variable Immunodeficiency: A Case Report**

Elisa Salamin^1^, Aki Kawasaki^1^

^1^Jules-Gonin Eye Hospital, Lausanne, Switzerland

**Introduction**: Non-thyroid orbital inflammatory diseases comprise a heterogeneous group of orbital disorders, distinct from thyroid-associated orbitopathy. They encompass a wide spectrum of aetiologies, including idiopathic forms, also referred to as orbital pseudotumor; infectious diseases, systemic inflammatory conditions such as sarcoidosis, granulomatosis with polyangiitis, IgG4-related disease and neoplastic lesions.

Common variable immunodeficiency (CVID), the most frequent primary immunodeficiency, is characterized by recurrent respiratory tract infections. In addition to infectious susceptibility, CVID is associated with non-infectious complications, including autoimmune disease, lymphoproliferative disorders, malignancies, and granulomatous inflammation involving multiple organs.

Ocular involvement in CVID is rare and has been reported only in isolated cases, mainly as granulomatous uveitis, multifocal choroiditis, or retinal vasculitis. Orbital involvement is particularly uncommon. One case report described sequential orbital B-cell lymphoma and Kaposi sarcoma in a CVID patient. Two additional reports described unilateral, non-granulomatous extraconal orbital masses with lacrimal gland infiltration and no extraocular muscle involvement.

Herein, we report a patient with CVID who developed painless, progressive ophthalmoplegia due to a granulomatous orbital inflammation with lacrimal gland infiltration and bilateral extraocular muscle involvement.

**Case Report**: A 74-year-old woman with a 5-year history of CVID was referred for evaluation of progressive diplopia. She had a combined IgG and IgA deficiency and a history of recurrent bronchopulomary and ENT infections including sinusitis, parotiditis and chronic sialadenitis. She developed granulomatous complications of CVID such as granulomatous–lymphocytic interstitial lung disease (GLILD) and granulomatous inguinal lymphadenitis, confirmed by biopsy. She reported binocular horizontal diplopia on left gaze for 18 months, with progression over the preceding 6 months. There was no ocular pain, redness, or visual loss. Examination revealed moderate limitation of abduction in the left eye, limitation of supraduction and infraduction of the right eye, and a subtle impression of left proptosis. Laboratory work-up was negative for myasthenia gravis, thyroid dysfunction and autoimmune disease. Orbital MRI demonstrated enlargement and enhancement of the left lateral rectus and right inferior rectus muscles, as well as the left lacrimal gland. Biopsy of the left lacrimal gland revealed chronic inflammation with fibrotic remodeling, associated with non-necrotizing chronic granulomatous inflammation, characterized by multiple small, naked granulomas containing giant cells.

**Discussion**: Granulomatous inflammation is a well-recognized complication of CVID, however orbital involvement is exceedingly rare. To our knowledge, this case of granulomatous orbital inflammation is novel for combined lacrimal gland and extraocular muscle involvement. The presence of multiple small non-necrotizing granulomas, together with the patient’s known other systemic granulomatous manifestations, supports immune dysregulation as the underlying pathogenic mechanism.

In conclusion, this case highlights the importance of considering CVID-related disease in atypical orbital inflammatory presentations.

**Disclosures**: The authors declare no disclosures.

64. **Functional in vivo confocal microscopy reveals corneal immune cells as peripheral biomarkers of neurodegenerative disease**

Emma Grace Oreskovic^1,2^

^1^Svjetlost Eye Clinic, Zagreb, Croatia; ^2^UCL Queen Square Institute of Neurology, London, UK

**Purpose**: Neurodegenerative diseases are associated with early and progressive immune dysregulation, yet accessible in vivo biomarkers reflecting these processes remain limited. The cornea represents a uniquely accessible, transparent, and highly regulated tissue in which resident immune cells can be visualised non-invasively. This study explores functional in vivo confocal microscopy (*Fun*-IVCM) as a method for assessing corneal immune cell behaviour as a potential peripheral biomarker of neurodegenerative disease, extending beyond classical sub-basal nerve plexus paramater analysis.

**Methods**: Laser-scanning in vivo confocal microscopy was performed with repeated volume acquisition of the corneal sub-basal nerve plexus and anterior stroma. Sequential image stacks from the same anatomical region were registered to generate time-lapse sequences, enabling analysis of immune cell dynamics in vivo. Functional parameters included immune cell displacement, instantaneous speed, arrest coefficient, and depth-dependent behaviour, analysed alongside conventional static measures of immune cell density and morphology. Standardised acquisition protocols were used to allow longitudinal and between-group comparisons.

**Results**: Fun-IVCM enabled reliable tracking of individual corneal immune cells within the living human cornea, revealing dynamic surveillance behaviours not captured by static imaging alone. Functional metrics demonstrated substantial inter-individual variability despite similar cell densities, indicating that immune cell behaviour provides additional biological information beyond morphometry. Depth-specific differences in immune cell dynamics were observed between epithelial and stromal compartments, supporting the feasibility of layered functional assessment in vivo.

**Conclusion**: Functional IVCM enables non-invasive, real-time assessment of corneal immune cell behaviour, offering a novel window into peripheral immune alterations relevant to neurodegenerative disease. By capturing dynamic cellular activity rather than structure alone, Fun-IVCM has potential as a sensitive peripheral biomarker for disease characterization, progression, and therapeutic response. This approach complements existing neural imaging modalities and supports the cornea as a translational platform for biomarker development in neurodegeneration.

65. **Syphilis resurgence: Neuro-ophthalmic manifestations**

Emma Spowart^1^, Aashna Jain^1^, Sinéad Connolly^1^, Sarah Walpole^2^, Ashley Price^2^, Ewan Hunter^2^, Ranjit Pandit^1^.​

^1^Newcastle Eye Centre, Royal Victoria Infirmary, Newcastle-upon-Tyne Hospitals NHS Foundation Trust​, Newcastle, UK; ^2^Department of Infectious Diseases and Tropical Medicine, Royal Victoria Infirmary, Newcastle-upon-Tyne Hospitals NHS Foundation Trust​, Newcastle, UK

**Introduction:** Following a recent increase in case numbers of ophthalmic syphilis in the north east of England seen at the Newcastle Eye Centre, we present a summary of those with optic nerve involvement.

**Methods:** Cases of ocular syphilis with optic nerve changes between 2003 and 2025 were reviewed. Data were collected on demographics; symptoms and signs; ocular comorbidities; HIV status; systemic symptoms; investigation and treatment before diagnosis; and visual outcomes at most recent follow up. Data collection and processing were in accordance with Caldicott principles and approved by information governance. Data collection and statistical analyses were performed in Microsoft Excel using t-test for numerical variables and chi-squared tests for categorical variables.

**Results:** Forty patients presented with ocular syphilis with optic nerve involvement. Patient numbers have significantly increased over time: 19 patients between 2002-2023 (average 0.86 and range 0-3 per year), 8 patients in 2024, and 13 patients in 2025. Seven patients had coexisting HIV infection. 30 had no ocular co-pathologies; 2 had cataract, 2 had amblyopia, 1 had amblyopia and previous retinal detachment, 3 had other retinal pathologies, 1 had glaucoma, and 1 keratoconus.

Systemic symptoms were present in 32 patients, most commonly rash (19), oral/genital ulcers (9), fevers/night sweats (7), hearing loss/tinnitus (8), headache (5), weight loss (5), joint pains (2), rectal bleeding (2), and confusion/disinhibition (2).

Five patients were referred from infectious diseases or sexual health with a confirmed diagnosis of syphilis, and 5 from other ophthalmology departments. 17 patients had previously presented to other healthcare services with systemic symptoms of syphilis, of whom 3 patients underwent lymph node biopsy and 1 gastroscopy. Five patients (age 56-68, mean 61.2) were initially treated as GCA with high dose steroids; 2 underwent temporal artery biopsies.

Visual acuity at presentation ranged from 6/4 to HM (-0.18-2.10 LogMAR, mean 0.52 LogMAR). 21 patients had anterior segment inflammation, 24 vitritis, and 22 retinal changes. 10 had only optic nerve signs. Optic disc findings were optic atrophy in 2 patients and optic disc swelling in 38, one of whom also had disc neovascularisation. At follow up (35 patients), mean VA was 0.35 LogMAR, with 10 patients losing vision and 19 patients gaining vision.

13 patients had an RAPD at presentation, 1 patient had no record of RAPD examination, and 1 patient had unreactive pupils but had reduced light brightness appreciation in the affected eye. 25 patients (19 bilateral, 6 unilateral) did not have an RAPD.

**Discussion:** This series highlights the importance of considering syphilis as a differential diagnosis in the presence of neuro-ophthalmic signs and symptoms. Early consideration avoids unnecessary investigations and ensures timely treatment with an aim to improve functional clinical outcome. Further work is underway to identify causative factors for the recent significant increase in case numbers, feeding into a review of provision and access of sexual health services in the region.

**Disclosures:** None of the authors have any relevant disclosures.

94. **Visual field defects in posterior cortical atrophy patients are not associated with retinal ganglion cell loss**

Laura Dell’Arti^1^, Claudia Mainetti^1^, Giorgio Fumagalli^2^, Francesco Bordoni^1^, Marco Nassisi^1^, Chiara Fornari^2^, Francesco Viola^1^

^1^Department of Clinical Sciences and Community Health, University of Milan, Milan, Italy; ^2^Center for Mind/Brain Sciences , University of Trento, Rovereto, TN, Italy

**Introduction**: Posterior cortical atrophy (PCA) is a neurodegenerative disorder that mainly affects the parieto-occipital cerebral cortex, which is involved in the processing of visual information. Previous studies have reported that hemianopic field defects are detected in PCA patients and that these hemianopias correlate with the side of greater cortical atrophy. Moreover, it is established that in organic diseases affecting the visual pathways, the lateralization of perimetric defects corresponds to homonymous atrophy of retinal ganglion cells (GCL) and reduction of peripapillary retinal nerve fibres (RNFL). However, to date, no study has evaluated GCL and RNFL in PCA patients presenting with hemianopia. Therefore, the aims of the study are:

1. To determine whether optical coherence tomography (OCT) reveals thinning of the GCL and RNFL in patients with PCA compared with healthy controls.
2. To assess the correlation between OCT parameters, visual field defects, and posterior cortical atrophy as assessed by magnetic resonance imaging (MRI)

**Methods**: Patients with a diagnosis of PCA were enrolled, along with a group of age-matched healthy controls. All participants underwent ophthalmological and neurological assessment, automated visual field testing, OCT scans and brain MRI.

A protocol of visual rating scales of atrophy, as previously described, was applied independently by two raters. In particular, the posterior scale was divided into five right and left subscales (dorsal parietal, posterior cingulate, precuneus, parieto-occipital, occipital). Volumetric measurements of the parietal and occipital regions inside the brain areas (defined by the automated anatomical labelling atlas) were assessed.

**Results**: 24 subjects were included in the study: 12 patients with PCA and 12 age- and sex-matched controls. Mean age at scanning was 69.4 ± 9.3 years in the PCA group. Mean disease duration was 4.1 ± 2.6 years. Mean visual acuity in the PCA group was 0.12 ± 0.60 LogMAR.

OCT data suitable for analysis were available for 10 patients. No statistically significant differences in GCL or RNFL thickness were observed between PCA patients and healthy controls. No statistically significant correlations were found between GCL/RNFL thickness and volumetric data.

Twenty-two visual fields of 24 eyes from PCA patients were suitable for analysis. In 14/22 eyes ≥3 quadrants were affected. A lateralized defect was present in 63.6% of visual fields, (left hemianopia in 50% of eyes, right hemianopia in 13.6%). The most frequent pattern was

a predominantly inferior visual field defect (61.1% eyes). Based on visual rating scales, the occipital and dorsal parietal regions showed a greater correspondence with the side of hemianopia compared with the other parameters.

**Discussion**: Our OCT data showing the absence of GCL thinning and MRI findings support the notion that, in PCA, hemianoptic visual field deficit arises from extrastriate occipital degeneration, with relative sparing of V1.

**Disclosures**: None.

98. **Giant cell arteritis in overdrive: Increased incidence and severity of ocular involvement and the role of early tocilizumab use**

Lea Kovac^1^, Ana Fakin^1^, Marko Hawlina^1^

^1^Eye Hospital, University Medical Centre Ljubljana, Slovenia

**Introduction**: Giant cell arteritis (GCA) is a sight-threatening vasculitis requiring prompt diagnosis and treatment. High-dose systemic glucocorticoids remain the cornerstone of initial therapy, while conventional immunomodulatory agents, most commonly leflunomide, are typically introduced as steroid-sparing treatment after disease stabilization or in cases of relapse. Tocilizumab, an interleukin-6 receptor inhibitor, is currently reserved for refractory or relapsing disease. In recent clinical practice, an apparent increase in disease incidence and severity has been observed, raising questions regarding optimal timing and choice of immunomodulatory therapy. We aimed to assess recent trends in GCA severity and to explore the role of early tocilizumab use in glucocorticoid (GC) resistant cases.

**Methods**: A retrospective review was conducted of all patients with ischemic ocular manifestations who were diagnosed with giant cell arteritis (GCA) at a tertiary referral centre between 2021 and 2025. Inclusion criteria comprised a confirmed diagnosis of GCA based on temporal artery ultrasound and/or biopsy, together with evidence of ocular ischemia, including optic neuropathy, retinal artery occlusion, or transient visual obscurations. Stabilisation or progression of ocular ischaemia despite immediate administration of high-dose intravenous glucocorticoid was recorded, as well as the use of early immunomodulatory therapy in glucocorticoid-resistant cases. Descriptive statistics were applied, and the number of treated patients and proportion of glucocorticoid-resistant cases in 2025 was compared with the preceding four years using Fisher’s exact test.

**Results**: A total of 30 patients were included. Annual case numbers increased in 2025 (12 cases) compared with 4–7 cases per year in 2021–2024. Female patients predominated overall. Glucocorticoid-resistant disease accounted for 6 of 12 cases (50%) in 2025, compared with 4 of 18 cases (20%) in 2021–2024, corresponding to a fourfold higher odds of severe presentation (odds ratio 4.0, p=0.12). Prompt immunomodulatory therapy was initiated in seven patients, five of whom did not respond to high dose glucocorticoid treatment. Three of these patients received early tocilizumab in addition to glucocorticoids, resulting in stabilization of visual function.

**Discussion**: Our findings suggest a recent increase in the proportion of glucocorticoid-resistant ocular GCA presentations, accompanied by more frequent early use of immunomodulatory therapy. Although limited by small sample size, the observed trend toward increased severity highlights the need for timely escalation of treatment in high-risk patients. Tocilizumab demonstrated favourable outcomes in selected glucocorticoid-resistant cases and may warrant consideration as a first-line immunomodulatory agent alongside glucocorticoids in vision-threatening GCA, rather than being reserved for later use after conventional agents such as leflunomide. Further prospective studies are required to define its optimal role in treatment algorithms.

**Disclosures** None.

**ARTIFICIAL INTELLIGENCE**

6. **Prospective AI-based fundus image identification of neuro-ophthalmic disorders in a tertiary emergency department**

Ayse Gungor^1,2,3^, Ana Banc^1,2^, Samy Zaher^1^, Ilias Sarbout^1,2,3^, Stefana Croitoru^1^, Ruth Huna-Baron^2^, Bouchra Touzani^1,2^, Cedric Lamirel^1^, Daniel Racoceanu^3^, François Audren^1,4^, Pearse A. Keane^5,6^, Dan Milea^1,2^

^1^Department of Neuro-Ophthalmology, Rothschild Foundation Hospital, Paris, France; ^2^Rothschild Computational and Visual Neurosciences Laboratory, Rothschild BRAIN Lab, Paris, France; ^3^Sorbonne Université, Institut du Cerveau—Paris Brain Institute—ICM, Inria, Inserm, CNRS, APHP, Hôpital de la Pitié Salpêtrière, Paris, France; ^4^Emergency Department, Rothschild Foundation Hospital, Paris, France; ^5^NIHR Biomedical Research Centre at Moorfields Eye Hospital NHS Foundation Trust, London, UK; ^6^Institute of Ophthalmology, University College London, London, UK

**Introduction**: Foundation models represent a new generation of self-supervised artificial intelligence (AI) trained on millions of fundus images, capable of learning visual representations of ophthalmic pathologies. We aimed to refine and prospectively evaluate a large-scale pretrained AI model for classification of neuro-ophthalmic disorders in an emergency department (ED).

**Methods**: Prospective clinical study enrolling consecutive patients presenting to a tertiary ophthalmology and neurology ED.

We built upon RETFound^1^, a vision-transformer–based foundation model pretrained on 1.6 million unlabeled fundus images using a self-supervised contrastive learning framework. To reinforce the model for neuro-ophthalmic multi-class classification, we performed supervised fine-tuning on a curated dataset of 6,240 expert-annotated fundus images (4,256 patients) including papilledema, pseudopapilledema, arteritic/non-arteritic anterior ischemic optic neuropathies [A/NAION], normal retinas, and “other” retinal and optic nerve conditions. Ground-truth diagnosis were independently established by two expert neuro-ophthalmologists through combined assessment of fundus images and clinical data, with adjudication by a senior expert in cases of disagreement. A novel preprocessing pipeline was developed to enhance inter-device consistency, and synthetic minority oversampling was applied to mitigate class imbalance.

**Results**: Among the 5,715 included patients presenting for ophthalmic and/or neurological complaints, this interim analysis evaluated the first 2,000 ED patients (1,164 women, 836 men) diagnosed with papilledema, pseudopapilledema, A/NAION, normal retinas, and “other” conditions. The model achieved a micro-averaged AUC of 0.97. Class-wise AUCs (95% CI) were: papilledema 0.97 (0.94–0.99), pseudopapilledema 0.98 (0.89–0.99), A/NAION 0.97 (0.92–0.99), normal retinas 0.93 (0.87–0.97) and “other” conditions 0.92 (0.85-0.99).

**Conclusion**: A fine-tuned large-scale vision-transformer–based model achieves high accuracy in identifying neuro-ophthalmic disorders from prospectively collected real-world ED fundus images. Extended multi-class evaluation will be performed in this ongoing prospective study.

**Disclosures:** D.M. is a consultant on the scientific advisory board of Optomed, Finland.

**Funding:** This study is supported by a research grant from VISIO Foundation, France.

^1^Zhou Y et al. A foundation model for generalizable disease detection from retinal images. *Nature*. 2023;622(7981):156-163.

17. **How much data is enough data in visual field analysis?**

Angelica Simonetti^1^, Catarina P. Coutinho^1,2^, Ferdinando Zanchetta^1^, Michele Carbonelli^3^, Marco Battista^4^, Alice Galzignato^2^, Chiara La Morgia^3,5,6^, Giulia Amore^3^, Martina Romagnoli^5^, Luigi Brotto^7^, Paolo Nucci^7^, Maria Lucia Cascavilla^4^, Claudio Fiorini^5^, Leonardo Caporali^5^, Francesco Bandello^4^, Valerio Carelli^3,5^, Rita Fioresi^1^, Piero Barboni^2,4^

^1^Department of Pharmacy and Biotechnology, University of Bologna, Bologna, Italy; ^2^Studio Oculistico d’Azeglio, Bologna, Italy; ^3^Department Biomedical and Neuromotor Sciences, University of Bologna, Bologna, Italy; ^4^Department of Ophthalmology, University Vita-Salute, IRCCS Ospedale San Raffaele, Milan, Italy; ^5^IRCCS, Istituto delle Scienze Neurologiche, Programma di Neurogenetica, Bologna, Italy; ^6^IRCCS, Istituto delle Scienze Neurologiche, UOC Clinica Neurologica; ^7^Department of Clinical Science and Community Health, University of Milan, Milan, Italy

**Disclosures**: The research of Catarina P. Coutinho was supported by a research scholarship funded by the European Union-Next Generation EU, Missione 4 Component 1 CUP J33C24001310009. The research of Rita Fioresi and Ferdinando Zanchetta was supported by CaLISTA CA 21109, CaLIGOLA MSCA-2021-SE-01-101086123, MSCA-DN CaLiForNIA—101119552, PNRR MNESYS, the PNRR National Center for HPC, Big Data, and Quantum Computing CUP J33C22001170001, SimQuSec CUP J13C2200068000; INFN Sezione Bologna, Gast initiative and GNSAGA Indam.

**Introduction**: “How much data is enough data?” is of the questions clinicians most frequently pose to quantitative scientists. This topic, central when statistical or mathematical analyses of data are planned, has become even more central as deep learning (DL) methods, known to be data hungry, gain traction in the field of neuro-ophthalmology. In glaucoma research, for instance, autoencoder models have emerged as an effective and versatile tool to study visual field (VF) data. In this study, we estimated how much data is needed to train from scratch state of art autoencoder architectures for the reconstruction of glaucomatous VFs.

**Methods**: We considered the open source Humphrey field analyzer (HFA) dataset from the Department of Ophthalmology at the University of Washington. It consists of 28943 VFs from 7248 eyes of 3871 patients comprising both healthy patients and patients affected by glaucoma. We trained a DL autoencoder belonging to the family of convolutional neural networks on the total deviation (TD) data available in the dataset. We normalized the TD values to be in the range 0-1. We used the data of 2787 patients to train our algorithm and the remaining ones for validation (701 patients) and testing (383 patients) purposes. We assessed the performance of our algorithm using both the mean pointwise TD reconstruction error (L1 score) and the Structural Similarity Index (SSIM), which provides a more perceptually and clinically relevant assessment than traditional pixel-wise metrics by accounting for the spatial relationships between visual field locations. We chose as baseline the performance obtained by training the model on the whole training set, fine-tuning its hyperparameters on the validation set to avoid overfitting. Then we retrained the algorithm on systematically generated subsets containing 75%, 50%, 25%, 10%, and 5% of the original training data evaluating its corresponding performance on the test set.

**Results**: Our findings demonstrate that reconstruction performance remains remarkably resilient to substantial reductions in data volume. In particular, while the model, trained on the full dataset, reached a SSIM score of 0.000486 and an L1 score of 0.000155 on the test set, we obtained comparable scores 0.000579 and 0.000168 respectively using only 10% of the data, that is, using data of less than 300 patients only. The performance sharply declined only at the 5% level.

**Discussion**: This case study suggests that to train certain Deep Learning algorithms in the context of analysis of VFs, the “data floor” might be lower than often assumed in DL paradigms. In particular, we found that effective autoencoder algorithms can be trained from scratch even on VFs datasets comprising a few hundred, not thousands, of patients. This opens the doors to deploying these algorithms, for example for disease progression recognition, also in clinical settings where data is scarce.

35. **Visual field transfer learning: From glaucoma to hereditary optic neuropathies**

Catarina P. Coutinho^1,2^, Ferdinando Zanchetta^1^, Angelica Simonetti^1^, Michele Carbonelli^3^, Marco Battista^4^, Alice Galzignato^2^, Chiara La Morgia^3, 5, 6^, Giulia Amore^3^, Martina Romagnoli^5^, Luigi Brotto^7^, Paolo Nucci^7^, Maria Lucia Cascavilla ^4^, Claudio Fiorini^5^, Leonardo Caporali^5^, Francesco Bandello^4^, Valerio Carelli^3,5^, Rita Fioresi^1^, Piero Barboni^2,4^

^1^Department of Pharmacy and Biotechnology, University of Bologna, Bologna, Italy; ^2^Studio Oculistico d’Azeglio, Bologna, Italy; ^3^Department Biomedical and Neuromotor Sciences, University of Bologna, Bologna, Italy; ^4^Department of Ophthalmology, University Vita-Salute, IRCCS Ospedale San Raffaele, Milan, Italy; ^5^IRCCS, Istituto delle Scienze Neurologiche, Programma di Neurogenetica, Bologna, Italy; ^6^IRCCS, Istituto delle Scienze Neurologiche, UOC Clinica Neurologica; ^7^Department of Clinical Science and Community Health, University of Milan, Milan, Italy

**Disclosures**: The research of Catarina P. Coutinho was supported by a research scholarship funded by the European Union-Next Generation EU, Missione 4 Component 1 CUP J33C24001310009. The research of Rita Fioresi and Ferdinando Zanchetta was supported by CaLISTA CA 21109, CaLIGOLA MSCA-2021-SE-01-101086123, MSCA-DN CaLiForNIA—101119552, PNRR MNESYS, the PNRR National Center for HPC, Big Data, and Quantum Computing CUP J33C22001170001, SimQuSec CUP J13C2200068000; INFN Sezione Bologna, Gast initiative and GNSAGA Indam.

**Introduction**: Visual field (VF) testing allows to analyse the functional damage in different optic neuropathies. Glaucoma, the most prevalent optic neuropathy, is typically associated with peripheric VF damages, on the other hand hereditary optic neuropathies like Dominant Optic Atrophy (DOA) and Leber Hereditary Optic Neuropathy (LHON) are rare diseases, generally characterized by central VF loss patterns. Building on previous works applying deep learning (DL) models (e.g. autoencoders) to glaucoma VF analysis, we explore transfer learning as a way to analyse VF reconstruction under limited data availability. In particular, we hypothesized that pre-training a DL model on glaucomatous VFs would lead to good performance when applying the model for the analysis of DOA and LHON VFs, since the underlying nature of the data is the same.

**Methods**: The open-access UWHVF dataset with 28 943 visual fields (VFs) of 3871 patients^1^was used for training and validation of a deep learning autoencoder. Internal databases of 280 VFs of DOA and 220 VFs of LHON molecularly confirmed patients were used for testing. All VFs were obtained with the Humphrey field analyzer (Carl Zeiss Meditec). Total deviation values were normalized between 0 and 1 and used as input to the convolutional neural network autoencoder. Performance was assessed with the L1 loss and visual comparison between the autoencoder reconstructed VFs, and the original VFs was carried out. The latent space was visually inspected using principal component analysis PCA for VF reduction to 2 dimensions. In addition, as a critical step for feature extraction and consequently decoder reconstruction, disease-specific drawn archetypes were plotted in the latent space to assess the VFs compression into a lower-dimension space by the encoder.

**Results**: For the Glaucoma validation (5210 VFs) and testing (2893 VFs) sets, losses of 0.000172 and 0.000155 were achieved. Even if trained on glaucoma VFs only, also low losses were obtained for both the DOA (0.000239) and LHON (0.00104) testing datasets. Visual comparison between the DL reconstructed VFs and original VFs, showed a high degree of detail at the pixel-level for different types of VF loss patterns. When analysing the latent space, archetypes were shown to lie along the perimetry enclosing the data cloud correspondent to the respective disease.

**Discussion**: Just as a deep learning model trained to recognize cats can be adapted to recognize dogs because they share features like eyes and ears; we trained an autoencoder architecture for the reconstruction of glaucoma VFs and evaluated its performance for the VF reconstruction of two distinct hereditary optic neuropathies. Preliminary results are promising, suggesting that, with model fine-tuning, transfer learning might result as a technique when data availability is limited.

Reference
^1^
Giovanni
Montesano, Andrew
Chen, Randy
Lu, Cecilia S.
Lee, Aaron Y.
Lee; UWHVF: A Real-World, Open Source Dataset of Perimetry Tests From the Humphrey Field Analyzer at the University of Washington. *Trans. Vis. Sci. Tech*. 2022;11(1):2. doi: 10.1167/tvst.11.1.1.PMC874253134978561

90. **Artificial intelligence-based classification of Parkinson’s disease using virtual reality-derived oculometrics**

Erika Han^1^, Ana Coito^2^, Hassan Fadavi^3^, Bruno Hauser^2^, Pia Massatsch^2^, Valentina Stozitzky^4^, Dongli Li^4^, Bettina Balint^1^, Helen Dawes^3^, Konrad P. Weber^1^

^1^University Hospital of Zurich, Switzerland; ^2^machineMD, Switzerland; ^3^University of Exeter, UK; ^4^gaitQ, UK

**Introduction**: Parkinson’s disease (PD) diagnosis remains largely based on clinical evaluation, creating a need for objective and quantifiable biomarkers to enhance diagnostic accuracy, enable earlier detection, and support longitudinal monitoring. Eye movement and pupillary abnormalities are increasingly recognized as sensitive indicators of PD-related dysfunction in ocular motor control pathways. Advances in virtual reality (VR)–based eye tracking allow standardized measurement of such parameters (“oculometrics”) and provide promising inputs for artificial intelligence (AI)–driven classification models. This study aimed to develop and evaluate machine learning approaches for distinguishing PD from healthy individuals using quantitative ocular motor and pupillary metrics acquired with a VR-based medical eye-tracking device.

**Methods**: Individuals with idiopathic PD were recruited at two clinical centres: Zurich (Switzerland) and Exeter (UK). Participants completed two study visits including standardized VR-based ocular motor testing alongside routine clinical examinations. Recordings with poor tracking quality or excessive signal loss were excluded. The analysis included 132 PD examinations and 148 examinations from an independent healthy control dataset. Each PD case was matched to an age- and sex-matched control using a globally optimal matching algorithm. Group differences were quantified using standardized mean differences (SMDs) and Welch’s t-tests. A supervised machine learning classifier was trained using the most discriminative oculometric features to distinguish PD from controls. Model performance was assessed separately for each site.

**Results**: The strongest PD–control differences were observed in saccadic measures (accuracy, peak velocity, and main sequence relationships), vergence parameters, ocular alignment, and pupillary dynamics including constriction/dilation velocity and response latency. The classifier demonstrated high diagnostic discrimination across both sites. In Zurich, performance reached an AUC of 0.96 with sensitivity of 0.96 and specificity of 0.89. In Exeter, the model achieved an AUC of 0.91 (F1-score 0.84; sensitivity 0.89; specificity 0.79).

**Discussion**: These preliminary findings suggest that VR-derived oculometrics provide robust biomarkers capable of differentiating individuals with PD from healthy controls. AI-based analysis of ocular motor and pupillary features may offer a scalable and objective tool to complement clinical assessment. Future work will focus on expanding cohorts, integrating datasets across centres, and correlating oculometric parameters with clinical disease measures to further improve diagnostic performance.

**Disclosures**: Ana Coito, Erika Han, Bruno Hauser and Pia Massatsch are employees of machineMD (Switzerland). Konrad P. Weber is part of the Medical Advisory Board of machineMD. Valentina Stozitzky and Dongli Li are employees of gaitQ (UK).

**MISCELLANEOUS**

8. **When neuro-ophthalmic disease signals hidden systemic pathology: Lessons from three contrasting cases**

Alaeldin Nour^1^, Nima Ghadiri^2^, Anna Visca^2^

^1^St Paul’s Eye Unit, Royal Liverpool University Hospital, Liverpool, UK; ^2^Liverpool University Hospitals NHS Trust, Liverpool, UK.

**Introduction**: Neuro-ophthalmic involvement is a recognised manifestation of autoimmune disease and a rare but important feature of paraneoplastic syndromes. Paraneoplastic or autoimmune optic neuropathy, cancer-associated or autoimmune retinopathy, and ocular motor syndromes within paraneoplastic neurological syndromes have all been described in association with malignancy, sometimes preceding cancer diagnosis and at other times emerging in the context of known systemic disease.

In clinical practice, interpretation is often challenging. Antibody findings vary in specificity, systemic imaging may be equivocal, and classical paraneoplastic patterns are not always present. Thus, diagnostic reasoning frequently requires integration of multiple clinical and investigative perspectives rather than reliance on a single test.

**Case Report**: We describe three patients presenting with neuro-ophthalmic disease who underwent comprehensive clinical assessment, autoimmune and paraneoplastic serology, neuroimaging, and targeted systemic imaging, with multidisciplinary review forming part of the diagnostic pathway.

The three cases illustrate complementary diagnostic scenarios within the same clinical spectrum.

The first patient presented with progressive ophthalmoplegia and imbalance alongside a non-classical antibody profile. Subsequent systemic investigation revealed mantle cell lymphoma, illustrating that ocular motor and cerebellar features may prompt consideration of paraneoplastic neurological syndromes even in the absence of classical antibody patterns.

Take-home 1: Non-classical or low-specificity antibodies may be encountered in patients ultimately found to have malignancy.

The second patient exhibited a more typical optic neuropathy phenotype, with bilateral disc swelling progressing to atrophy and positive anti-α-enolase antibodies, alongside abnormal initial lung imaging. Multidisciplinary review, however, confirmed inflammatory interstitial lung disease rather than malignancy, highlighting the limited tumour specificity of some antibodies and the importance of contextual interpretation.

Take-home 2: Even with apparently supportive serology and imaging, clinical context and multidisciplinary assessment remain essential.

The third patient presented with bilateral disc swelling responsive to corticosteroids but evolving into optic atrophy, with negative autoimmune and paraneoplastic serology. Subsequent systemic imaging nevertheless identified underlying malignancy.

Take-home 3: Seronegativity does not necessarily exclude underlying malignancy; phenotype and clinical trajectory should guide ongoing surveillance.

**Discussion**: This series offers three contrasting clinical perspectives within a rare and diagnostically challenging group of disorders, illustrating the complexity of autoimmune and paraneoplastic neuro-ophthalmic disease. Rather than supporting reliance on any single marker or investigation, these cases emphasise the importance of integrating clinical phenotype, objective neuro-ophthalmic findings, multidisciplinary interpretation, and longitudinal assessment.

In each case, systemic diagnosis led to targeted specialist referral and re-framing of treatment goals, shifting management from isolated ophthalmic care to coordinated systemic management. In such rare and diagnostically uncertain conditions, cautious synthesis of multiple data points remains central to appropriate patient care and timely identification of underlying systemic disease.

**Disclosure**: The authors declare no conflict of interest.

27. **How to diagnose cerebral visual impairment (CVI): Recommendations for clinical practice**

Bianca Huurneman^1^, Jannet Philip^2^, Nomdo M. Jansonius^3^, Frounke Nienke Boonstra^4^

^1^Royal Dutch Visio, Nijmegen, The Netherlands; Department for Cognitive Neuroscience, Radboud University Medical Center, Nijmegen, the Netherlands; ^2^Royal Dutch Visio, National Foundation for the Visually Impaired and Blind, Huizen, The Netherlands; Department of Ophthalmology, University of Groningen, University Medical Center Groningen, Groningen, The Netherlands; ^3^Department of Ophthalmology, University of Groningen, University Medical Center Groningen, Groningen, The Netherlands; ^4^Royal Dutch Visio, National Foundation for the Visually Impaired and Blind, Huizen, The Netherlands; Department of Ophthalmology, Radboud University Medical Center, Nijmegen, The Netherlands

**Introduction**: Cerebral visual impairment (CVI) is a heterogeneous condition and diagnosing CVI can be challenging. In 2022, multidisciplinary guidelines for diagnosis and referral in CVI were published. Current research activities are aimed at increasing diagnostic clarity by using data-driven subtyping of CVI.

**Methods**: The diagnostic guidelines were developed based on the GRADE method. Articles were selected on five topics: 1) medical history and CVI-questionnaires, 2) ophthalmological and orthoptic assessment, 3) neuropsychological assessment, 4) neuroradiological evaluation and magnetic resonance imaging, and 5) genetic assessment. The subtyping studies focused on: 1) comparing CVI subtypes identified by a limited (4 measures: visual acuity, contrast sensitivity, ocular alignment, and visual perception) and extensive test battery (10 measures), and 2) identifying CVI subtypes in a verbally testable cohort of children with CVI and comparing structural and functional measures to those of age-matched peers. For the subtyping studies, principal component analysis was followed by cluster analysis to determine the optimum number of CVI subtypes.

**Results**: The CVI guidelines indicate that in medical history perinatal damage and genetic factors are important. In ophthalmological and orthoptic assessment, distant and near vision, crowding, eye movements, accommodation, contrast sensitivity, visual fields and OCT should be evaluated. Screening for visual perceptive functioning is recommended and can guide further assessment. MRI abnormalities (e.g. lesion severity) can be associated with visual function impairment. Additional assessment (neurologic or genetic) is necessary to obtain the cause (etiology) of CVI if there is no perinatal damage in history.

The subtyping studies indicate that using an extensive above a limited test battery is preferable, especially in picking up lower-level visual function deficits and abnormal eye movements. Finally, adding structural measures such as OCT to CVI assessments provide the benefit of revealing structural and functional outcomes.

**Discussion**: In CVI diagnosis there are three key components of assessment: 1) Medical history; birth-related characteristics and etiology, 2) visual function measures and 3) structural abnormalities associated with CVI (e.g. OCT, MRI). The diagnostic guidelines provide direction for health professionals in prescribing the choice of measures to diagnose CVI. The subtyping studies indicate that CVI is not a single visual disorder and that within the group of children with CVI, there are subtypes with distinct functional and structural profiles. Across subtyping studies, consistent evidence is found for two subtypes: 1) a group with (near) normal ophthalmological assessment but pronounced higher level visual and structural deficits (often associated with prematurity and perinatal hypoxia), and 2) a global low and high level visual deficits group (often associated with genetic abnormalities).

**Discolusures**: The authors declare that this research was conducted in the absence of any commercial or financial relationships that could be construed as a potential conflict of interest.

29. **Binocular pupillography with virtual neutral density filtering detects early afferent gain**

Bjørn André Helland-Hansen^1^, Goran Petrovski,^2^ Alexander Sverstad,^2^ Stig Larsen^3^

^1^Bulbitech, Norway; ^2^Center for Eye Research and Innovative Diagnostics (CERID), University of Oslo, Norway; ^3^Meddoc Research, Oslo, Norway

**Purpose**: To investigate whether early and intermediate non-exudative age-related macular degeneration (AMD) is associated with measurable dysfunction of the afferent pupillary pathway, and to characterise the underlying mechanism using quantitative binocular pupillography with programmatic neutral density (ND) filter equivalents.

**Methods**: Twelve patients with mild to moderate non-exudative AMD and ten age-matched healthy controls underwent binocular pupillary light reflex testing using high-frequency infrared video-oculography. A computerised swinging-flashlight paradigm was implemented by alternately stimulating each eye with calibrated light intensities spanning a defined inter-ocular ratio range. This approach replaces physical ND filters with *virtual ND filters*, allowing continuous, symmetric, and precisely quantified attenuation to be applied independently to each eye. Linear regression with bootstrapping was used to derive two key metrics: RAPDlog, representing the virtual ND filter value required to equalise bilateral pupillary responses, and *Response*, defined as the change in pupil diameter per 0.3 ND unit and reflecting absolute afferent gain. AMD patients completed six repeated sessions to assess repeatability and stability. Diagnostic performance was evaluated using ROC analysis.

**Results**: Both RAPDlog and Response significantly differentiated AMD patients from healthy controls (p < 0.05). RAPDlog demonstrated good population-level discrimination (AUC 0.84), indicating subtle inter-ocular imbalance in afferent input. However, Response showed superior within-subject repeatability and stability, while remaining diagnostically informative (AUC 0.69). This indicates a consistent reduction in overall afferent drive to the pupillary system in AMD, independent of inter-eye asymmetry.

**Conclusions**: Quantitative pupillography using virtual ND filters enables mechanistic separation of afferent asymmetry and absolute afferent gain. In early AMD, reduced pupillary Response suggests diminished cone-driven input to intrinsically photosensitive retinal ganglion cells, leading to lower overall pupillary gain even in symmetric disease. Absolute afferent gain may therefore represent a more robust and physiologically meaningful functional biomarker than asymmetry-based RAPD measures alone. These findings support binocular pupillography as a sensitive, non-invasive tool for neuro-ophthalmic assessment of early retinal dysfunction.

33. **The neck mass behind Horner syndrome: A case of cervical paraganglioma**

Carlos Leis-Cofiño^1^, María Castro Rebollo^1^

^1^Ophthalmology Department, Hospital Universitario La Paz, Madrid, Spain

**Introduction**: Tumors of the carotid body account for less than 0.5% of all neoplasms. Among these rare tumors are paragangliomas, which are neuroendocrine tumors that rarely arise in the head and neck region, where they typically present as slow-growing, sometimes non-painful masses. Although these tumors generally produce non-specific symptoms, they may present with Horner syndrome in approximately 8% of cases. Here, we present a case of a carotid body paraganglioma presenting with Horner syndrome.

**Case Report**: A 71-year-old woman presented with a two-year history of left upper eyelid drooping. On further questioning, she also reported intermittent episodes of dysphonia during the same period. Ophthalmologic examination revealed mild ptosis, with a palpebral fissure width of 11 mm in the right eye and 9 mm in the left eye. Pupillary examination showed anisocoria, with a miotic left pupil that was more pronounced under scotopic conditions. The 0.5% apraclonidine test reversed the anisocoria and equalized the palpebral fissure width. Physical examination revealed a painless mass on the left lateral aspect of the neck. Given the suspicion of Horner syndrome in the presence of a cervical mass, a cervicothoracic computed tomography (CT) scan was requested, revealing a highly vascularized mass centered at the left carotid bifurcation, surrounding anterior margin of the cervical interna carotid artery. An otolaryngology consultation was obtained for further evaluation and surgical intervention. Imaging and histopathological findings confirmed the diagnosis of a carotid body paraganglioma as the cause of Horner syndrome.

**Discussion**: These tumors are composed of chromaffin cells derived from the neural crest and may be located in the adrenal gland (their most common site), where they are known as pheochromocytomas, or in extra-adrenal locations, where they are referred to as paragangliomas; those arising in the head and neck account for only approximately 3% of cases. They are generally benign, and surgical excision is curative in most cases. Only about 2% of paragangliomas secrete catecholamines; therefore, compressive symptoms are more common than systemic manifestations. Neuro-ophthalmologic symptoms are uncommon; among them, Horner syndrome is the most frequently reported. Less commonly, they may present with papilledema secondary to impaired venous outflow when the tumor is located in the jugular region, as described in the case series by Lertakyamanee et al.

The presence of Horner syndrome represents a diagnostic challenge for ophthalmologists due to the wide range of possible differential diagnoses. This case highlights the critical role of thorough physical examination in guiding the diagnostic process, complemented by appropriate imaging studies.

**Disclosure**: No potential conflict of interest was reported by the authors. Patient consent was obtained.

53. **Movement and sight: A case of type 7 spinocerebellar ataxia diagnosed in a small regional hospital**

Diana Rotaru^1^, Ion Rotaru^2^

^1^Department of Ophthalmology, „Elena Beldiman” Emergency Hospital, Barlad, Romania; ^2^Department of Neurology, „Elena Beldiman” Emergency Hospital, Barlad, Romania

**Introduction**: Spinocerebellar ataxias comprise a large and increasingly numerous category of autosomal dominant inherited neurodegenerative disorders. Their main characteristic is cerebellar ataxia, accompanied by a range of other neurological and non-neurological manifestations that help distinguish between various types of spinocerebellar ataxias.

**Case Report**: We describe the case of a 59-year-old man who was referred to our Neurology Department by the regional social services. He was solitary, without silblings or children, bedridden, and had not undergone a neurological evaluation throughout his more than ten-year disease history.

Previously a shepherd, he reported that the first symptoms manifested at 49, when he observed that he could not chase after his flock anymore. By the age of 55, he had lost the ability to walk and needed assistance from his neighbors to carry out his basic needs. Notably, he has a positive family history: his mother had similar, evolutive symptoms starting at the age of 40.

At the time of his first visit, his neurological exam was that of a severe pure cerebellar ataxia: prominent postural and action tremor of limbs and head, limb and truncal ataxia, he could not stand or walk, had cerebellar dysarthria, severe visual impairment, and no pyramidal, extrapyramidal, sensory signs, or cognitive impairment. A noteworthy feature was that he was constantly squinting his eyes, even in the room light, suggestive of photophobia.

An ophthalmology examination was undertaken. His visual acuity was recorded at 3/50; funduscopy revealed severe generalized retinal degeneration with nummular confluent and non-confluent atrophic lesions at the macula, around the optic disc, contiguous to vascular arcades, optic disc pallor, and granular pigmentary deposits. Optical coherence tomography features included loss of the ellipsoid zone, disruption of the inner-outer segment junction of photoreceptors.

Cerebral MRI findings were consistent with severe cerebellar atrophy, more prominent in the vermis. We do not have the possibility for genetic testing in our hospital. Nonetheless, the typical neurological and ophthalmological presentation, with a long period of disease evolution, slow progression, and a positive familial history, allows us to suspect the diagnosis of spinocerebellar ataxia type 7 with a considerable degree of certainty.

The treatment started was propranolol 120 mg/day and clonazepam 1.5 mg/day, with unexpected clinical improvement at the 6-month reevaluation, he had no head tremor and significantly improved limb tremor so that he could take the meals and do some basic tasks independently. Regrettably, he was unable to regain the ability to walk or stand independently.

**Conclusions**: We hope to have the opportunity to present this clinical case to emphasize once more the importance of collaboration between neurologists and ophthalmologists, and the necessity of a thorough clinical evaluation, which can lead to a diagnosis, even in a setting where available explorations are limited.

**Disclosures**: We do not have any disclosures to make.

56. **Triangulating relative afferent pupillary defect assessment: Functional, structural and clinical perspectives**

Dominik Brügger^1^, Melina Katsimpoura^2^, Julian Lehmann^1^, Bruno Hauser^1^, Emilie Reuter^2^, Justus Luerweg^2^, Johanna Haselon^2^, David Bratek^2^, Ralf Gold^2^, Jeremias Motte^2^, Ruth Schneider^2^, Anke Salmen^2^

^1^machineMD, Bern, Switzerland; ^2^Dept. of Neurology, St. Josef-Hospital, Ruhr-University Bochum, Bochum, Germany

**Introduction**: Relative afferent pupillary defect (RAPD) is a key clinical sign of asymmetric optic nerve dysfunction. Its assessment is limited by the subjectivity of clinical examination and the restricted sensitivity of structural imaging using optical coherence tomography (OCT), particularly in the acute phase. In the absence of a reliable gold standard, discordance between clinical, structural, and functional measures is expected. This study aimed to compare these modalities and to assess the contribution of quantitative pupillometry as a continuous functional measure for interpreting RAPD under real-world diagnostic conditions.

**Methods**: Data from 520 subjects were analyzed, including 132 healthy controls, 265 patients with multiple sclerosis–related disorders, 58 with other demyelinating diseases, and 38 with neurodegenerative conditions. RAPD was assessed clinically and quantified using the RAPD neoscore derived from binocular pupillometry (neos, machineMD). Clinical RAPD assessment was derived from prospectively collected routine clinical documentation, reflecting real-world neurological practice rather than a standardized examination protocol within a clinical trial setting. Peripapillary retinal nerve fibre layer (pRNFL) thickness asymmetry was available in a subset of participants (n=103).Abnormality was defined separately for each modality using predefined thresholds. Patterns of concordance and discordance between clinical assessment, RAPD neoscore, and OCT were analyzed to characterize complementary detection across modalities rather than accuracy against a reference standard.

**Results**: RAPD neoscore showed concordance with clinical RAPD assessment, demonstrating good discriminative performance relative to clinician impression (ROC AUC 0.82). In addition, functional afferent asymmetry as reflected by RAPD neoscore was observed in cases without OCT-detectable unilateral RNFL thinning, including cases with and without concordant clinical RAPD assessment. In clinically borderline or uncertain examinations, RAPD neoscore yields a continuous directional representation of afferent asymmetry without reliance on dichotomizing thresholds. Overall, overlap between modalities was partial, with each method identifying distinct but intersecting subsets of cases.

**Conclusions**: Clinical examination, structural OCT, and quantitative pupillometry capture different aspects of afferent visual pathway dysfunction, and no single modality provides a complete assessment of RAPD. In the absence of a definitive gold standard, analysis of detection overlap highlights the complementary nature of these approaches. RAPD neoscore contributes objective functional information beyond both clinical impression and structural imaging, supporting its role as a clinically useful adjunct in routine RAPD assessment, including situations characterized by diagnostic uncertainty.

72. **Slowly progressive Leigh Syndrome associated with NDUFAF3 Mutation**

Alice Galzignato,^1^ Anna Ardissone,^2^ Marco Battista,^3^ Leonardo Caporali,^4^ Claudio Fiorini,^4^ Luigi Brotto,^5^ Paolo Nucci,^5^ Valerio Carelli,^4,6^ Piero Barboni^1,3^

^1^Studio Oculistico d’Azeglio, Bologna, Italy; ^2^Unit of Child Neurology, Fondazione IRCCS Istituto Neurologico “Carlo Besta”, Milan, Italy; ^3^Department of Ophthalmology, University Vita-Salute, IRCCS Ospedale San Raffaele, Milan, Italy; ^4^IRCCS, Istituto delle Scienze Neurologiche, Programma di Neurogenetica, Bologna, Italy; ^5^Department of Clinical Science and Community Health, University of Milan, Milan, Italy; ^6^Department of Biomedical and Neuromotor Sciences, University of Bologna, Bologna, Italy

**Introduction**: Leigh syndrome (LS) is a mitochondrial encephalopathy characterized by bilateral symmetric lesions of the basal ganglia and brainstem, typically presenting in infancy with severe neurological deterioration and high mortality. Mutations affecting mitochondrial complex I assembly factors, including *NDUFAF3*, are recognized causes of LS. Neuro-ophthalmological features are often overlooked due to the high severity of the disease. We report a mild LS phenotype associated with *NDUFAF3* compound heterozygous variants, initially detected through progressive visual impairment.

**Case Report**: A 7-year-old boy was referred for neuro-ophthalmologic evaluation due to bilateral decreased visual acuity and optic disc pallor. Previous orthoptic examinations at age 4 were normal, despite concerns raised by both parents and teachers regarding his visual function. At presentation, best-corrected visual acuity was 3/10 in the right eye and 2/10 in the left eye, with esotropia, sluggish pupillary responses, and optic disc pallor in both eyes. Optical coherence tomography showed severe thinning of the retinal nerve fiber layer and ganglion cell layer, and visual fields revealed diffuse cecocentral scotomas.Brain MRI demonstrated bilateral putaminal and caudate nucleus signal abnormalities with diffusion restriction and optic nerve hypotrophy. Genetic testing identified compound heterozygosity for a pathogenic *NDUFAF3* variant (c.489_490 p.Gly164Serfs*29) and a novel variant of uncertain significance (c.5C>A; p.Ala2Asp), consistent with mitochondrial disease. Neurological examination confirmed a mild Leigh syndrome phenotype with global motor delay and fine motor incoordination. Treatment with riboflavin, thiamine, and coenzyme Q10 was initiated. Over two years of follow-up, visual, cognitive, and motor functions remained stable. Introduction of idebenone therapy is planned according to pharmacogenomic profiling of NQO1.

**Discussion**: This case expands the phenotypic spectrum of *NDUFAF3*-related disease by highlighting optic neuropathy as the leading manifestation in a clinically mild form of LS. Unlike classic LS, which is rapidly progressive and often fatal in early childhood, this patient showed predominant visual involvement with subclinical basal ganglia lesions and minimal neurological impairment. Visual presentations in *NDUFAF3* mutations are rarely reported, likely due to the typically severe course of the disease. Early neuroimaging and genetic testing in children with unexplained optic neuropathy are crucial for diagnosis. Close monitoring and early targeted treatment, including idebenone, may help preventing progression of cerebral lesions and improve long-term outcomes.

**Disclosure**: The authors declare no conflicts of interest. Written informed consent was obtained from the patient’s parents for publication of this case report.

75. **An unresolved case of polyradiculitis and retinal atrophy,**

Virginie Buehler^1^, Sabine Fenzl^2^, Florian Heussen^1^, and Hilary M. Grabe^1^

^1^Department of Ophthalmology, Inselspital, Bern University Hospital, Bern, Switzerland; ^2^University Institute of Diagnostic and Interventional Neuroradiology, Inselspital, Bern University Hospital, Bern, Switzerland

**Introduction**: We present a challenging case of rapidly progressive polyradiculitis with cranial nerve involvement and progressive retinal atrophy.

**Case Report**: A 57-year-old woman with a history of Stage IVA follicular lymphoma on rituximab maintenance therapy and mild pulmonary infection one month prior presented with worsening vision and diplopia. She complained of spots in her visual field and difficulty focusing starting 10 days prior to presentation. Her ophthalmologist documented normal visual acuity (VA) in both eyes and mild vitreous opacities. She developed diplopia three days later followed by leg weakness, difficulty walking and paresthesias of the hands, feet and chin.

Upon admission she had mild upper and moderate lower extremity weakness with absent patellar and achilles reflexes. Lumbar puncture (LP) showed pleocytosis and elevated protein; cytology was negative. Ophthalmic examination revealed a VA of 0.5 on the right and 0.2 on the left with a mild left relative afferent pupillary defect, left gaze palsy and small right hypertropia. Fundus examination demonstrated bilateral pigment mottling with white-gray pigment surrounding the macula. OCT demonstrated distortion and loss of the outer retinal layers, retinal pigment epithelium atrophy and subretinal deposits. Fundus Autofluorescence showed patchy hypofluorescence with a hyperfluorescent rim. An electroretinogram was not consistent with cancer associated retinopathy.

MRI showed subtle asymmetric enhancement of the cisternal oculomotor nerves and the facial nerves and enhancement of the cauda equina. She was treated for possible Guillain Barre Syndrome (GBS) with intravenous immunoglobulin (IVIG) and plasmapheresis without improvement. Anti-ganglioside antibodies were negative, although the sensitivity was decreased due to immunoglobulin G deficiency. Positron emission tomography–computed tomography (PET-CT) showed no evidence of lymphoma or other malignancy.

She rapidly deteriorated with tetraplegia, loss of all cranial nerve function, and hypesthesia. Her VA worsened to count fingers with progressive retinal atrophy. Enhancement of the oculomotor and facial cranial nerves and cauda equina in the MRI increased significantly. Electroneuromyography was consistent with progressive demyelination.

Extensive workup, including multiple LPs, bone marrow biopsy, and retinal and vitreous biopsy, yielded no diagnosis. Based on the rapidly progressive retinal atrophy, a presumptive diagnosis of ocular lymphoma was made and she was treated with methotrexate and cytarabine with no improvement. Palliative care was instituted and the patient died several hours later, less than three months after her initial symptoms.

**Discussion**: The diagnosis remains elusive. Given her rapid neurologic progression and history of follicular lymphoma, neurolymphomatosis was considered; however, PET-CT was negative and she did not respond to methotrexate. Ocular lymphoma remains likely based on retinal atrophy, but PET-CT and multiple LPs showed no evidence of lymphoma to explain her neurologic symptoms. A paraneoplastic variant of GBS is also possible, though there was no treatment response and retinal atrophy is not explained by this diagnosis.

**Disclosures**: None.

76. **Ophthalmological findings in children with a primary brain tumor: The visual function up to two years after diagnosis**

Inge J.M. Corver^1,2^, Giorgio L. Porro^1^, Inge Stegeman^1,3,4^, Antoinette Y.N. Schouten-van Meeteren^2^, Saskia M. Imhof^1^

^1^Department of Ophthalmology, University Medical Center Utrecht, the Netherlands; ^2^Department of Neuro-Oncology, Princess Máxima Center for pediatric oncology, the Netherlands; ^3^Department of Otorhinolaryngology and Head & Neck Surgery University Medical Center Utrecht, the Netherlands; ^4^Brain Center, University Medical Center Utrecht, the Netherlands

**Background and Aims**: Visual impairment can be a progressive and irreversible adverse event in children with a brain tumor. In addition, visual impairment can highly impact a child’s quality of life and daily participation. The prevalence and types of ophthalmological findings in children with a brain tumor are unknown, and there are no standardized international guidelines for screening and follow-up. Overall, it is unclear how visual functions develop during and after treatment. Therefore, this study aims to describe the relationship between visual function up to two years after diagnosis, the course of disease, and different oncological treatments in children with a primary brain tumor.

**Methods**: In this prospective cohort study, children (0-18 years) diagnosed with a primary brain tumor (between May 2019 and September 2021) were enrolled in four hospitals in the Netherlands. A standardized ophthalmological examination and Optical Coherence Tomography (OCT) were performed within 4 weeks of brain tumor diagnosis. Follow-up visits were planned at 6, 12, 18, and 24 months. The main ophthalmological endpoints were visual acuity and visual field. Data analysis was performed using descriptive statistics.

**Results**: Of the 170 included children (median age 8.3 years), 78.8% presented with abnormal ophthalmological findings at diagnosis. These findings included abnormal orthoptic evaluation (e.g., nystagmus, gaze deficits, or strabismus), papilledema, abnormal split lamp examination, or visual impairment. Among the patients with visual impairment, 13 had bilateral decreased visual acuity, and 32 had a visual field defect at diagnosis. At 6, 12, 18, and 24 months, bilateral decreased visual acuity was present in 9, 8, 5, and 6 of the evaluable patients. Visual field examination was abnormal in 35, 29, 34, and 39 patients, respectively.

**Conclusions**: These results confirm a high prevalence of abnormal ophthalmological findings in children at primary brain tumor diagnosis and after treatment. Our study highlights the importance of standardizing ophthalmological follow-up in this group. Our future research will focus on defining an end-of-follow-up group within pediatric brain tumor patients and the role of OCT in predicting visual outcomes in these children.

78. **The obesity paradox in korean patients with diabetes: An inverse relationship between higher body mass index and the risk of non-arteritic anterior ischemic optic neuropathy**

Jaeryung Kim^1^, Kyungdo Han^2^, Sei Yeul Oh^3^, Kyung-Ah Park^1^

^1^ Department of Ophthalmology, Samsung Medical Center, Sungkyunkwan University School of Medicine, Seoul, South Korea; ^2^Department of Statistics and Actuarial Science, Soongsil University, South Korea; ^3^Department of Ophthalmology, Kim's Eye Hospital, Seoul, South Korea

**Introduction**: While Type 2 diabetes mellitus (T2DM) is an established risk factor for non-arteritic anterior ischemic optic neuropathy (NAION), the precise impact of obesity metrics like body mass index (BMI) and waist circumference (WC) on NAION development in this patient population has not been thoroughly investigated. This study sought to clarify this association, aiming to deepen the understanding of NAION’s pathophysiology and help refine clinical management approaches.

**Methods**: A retrospective, population-based cohort study was conducted using the nationwide Korean National Health Insurance Service database. The study included 2,178,910 Korean adults diagnosed with T2DM who were enrolled between 2015 and 2016. These participants were monitored through 2022, for an average follow-up period of 5.74 years. Cases of NAION were identified based on the ICD-10 code H47.0, supplemented by specific criteria related to healthcare service use. BMI was stratified into five categories according to the Asia-Pacific obesity classification, while WC was grouped based on sex-specific thresholds. To analyze the link between BMI, WC, and NAION risk, Cox proportional hazards regression models were utilized, with adjustments for a comprehensive set of confounding variables such as age, sex, income, lifestyle factors, co-existing health conditions, fasting blood glucose levels, the duration of diabetes, and medication use.

**Results**: Over a total of 12,512,222.58 person-years of observation, 13,524 new cases of NAION were recorded. In comparison to the reference group (BMI 18.5–23.0 kg/m^2^), individuals in higher BMI categories demonstrated a significantly reduced risk of developing NAION. Specifically, the hazard ratio (HR) was 0.917 (95% CI: 0.878–0.958) for the BMI 25.0–30.0 kg/m^2^group and 0.782 (95% CI: 0.725–0.843) for the BMI ≥30.0 kg/m^2^group. A similar protective effect was observed with waist circumference; the group with the largest WC had a 12.6% lower risk of NAION (HR: 0.874, 95% CI: 0.816–0.935) relative to its reference category (WC 85–90 cm for men, 80–85 cm for women).

**Discussion**: This study reveals that among Korean patients with T2DM, elevated BMI and WC are significantly correlated with a decreased risk of NAION. These results contest the conventional understanding of obesity as a risk factor for NAION and offer critical insights into the disease’s pathophysiology within the diabetic population. This could have important implications for clinical practice, particularly in light of ongoing discussions about the use of GLP-1 receptor agonists and their potential connection to NAION risk.

**Disclosures**: The authors have no competing financial interests to declare.

79. **A multicenter analysis of the clinical spectrum of anti-GQ1b antibody syndrome**

Jasmine Yaowei Ge^1^, Wang Yu Hang^1^, Umapathi N Thirugnanam^1,2,3^, Loo Jing Liang^1,2^, Christen Lim LS^3^

^1^Singapore National Eye Centre, Singapore; ^2^Singapore Eye Research Institute, Singapore; ^3^National Neuroscience Institute, Singapore

**Purpose**: Anti-GQ1b antibody syndrome encompasses a spectrum of autoimmune neurological disorders with clinical manifestations. Traditionally, Miller Fisher syndrome (MFS) —defined by the triad of ophthalmoplegia, ataxia, and areflexia — has been the hallmark phenotype linked to these antibodies. However, acute ophthalmoplegia without accompanying ataxia represents an important and increasingly recognized phenotype within this syndrome. The study seeks to determine the incidence of acute ophthalmoplegia without ataxia in patients who tested positive to the anti-GQ1b antibody.

**Methods**: A retrospective review of patients with positive anti-GQ1b antibody sent to the neuromuscular lab at the National Neuroscience Institute between 2020 to 2025 was reviewed. Data was extracted across three hospitals in Singapore to characterize the demographic, clinical, immunological, and treatment outcome profiles of patients diagnosed with Anti-GQ1b antibody syndrome and its various clinical variants - including Miller Fisher syndrome (MFS), Guillain-Barré syndrome (GBS), ophthalmoplegia (AO), and other related phenotypes. Clinical data includes demographics, preceding infections, ophthalmological as well as neurological symptoms and signs; laboratory data comprised ganglioside antibody profiles, cerebrospinal fluid analysis, and neuroimaging. Statistical comparisons were made between phenotypic subgroups to identify significant differences in presentations and outcomes.

**Results**: A total of 113 patients were retrospectively analyzed, with 24 MFS, 19 GBS, 23 AO and 47 other presentation patients. Of note, there was no age, gender or ethnic difference in their presentations. A preceding infection was most commonly seen in MFS variants (83%) followed by AO (52%), which was statistical significant. Anti-GQ1b antibodies were present in all MFS and AO cases and in over a third of GBS and other variants, confirming its central role in pathogenesis. Other ganglioside antibodies such as GT1a was positive in 48% of AO patients, while GD1a was 53% positive in GBS patients, suggesting a possible role of these markers in the disease. Immunotherapy was administered in over half the cases, yielding favorable outcomes with median recovery times ranging from 35 to 110 days depending on phenotype severity.

**Conclusion**: This study highlights the clinical heterogeneity and immunological complexity of Anti-GQ1b antibody syndrome. The consistent presence of GQ1b antibodies across phenotypes reinforces its diagnostic relevance. Early identification and immunomodulatory treatment facilitate effective recovery, although clinical manifestations and recovery timelines vary by subtype. Therefore it is important to consider AO as a differential in acute isolated ophthalmoplegia.

References1.Lee
SU, Kim
HJ, Choi
JY, Choi
KD, Kim
JS.
Expanding Clinical Spectrum of Anti-GQ1b Antibody Syndrome: A Review. JAMA Neurol. 2024 Jul
1;81(7):762–770. doi: 10.1001/jamaneurol.2024.1123.38739407
2.Noioso
CM, Bevilacqua
L, Acerra
GM, Valle
PD, Serio
M, Pecoraro
A, Rienzo
A, De Marca
U, De Biasi
G, Vinciguerra
C, Piscosquito
G, Toriello
A, Tozza
S, Barone
P, Iovino
A.
The spectrum of anti-GQ1B antibody syndrome: beyond Miller Fisher syndrome and Bickerstaff brainstem encephalitis. Neurol Sci. 2024
Dec;45(12):5657–5669.38987510
10.1007/s10072-024-07686-33.Lo
YL, Chan
LL, Pan
A, Ratnagopal
P.
Acute ophthalmoparesis in the anti-GQ1b antibody syndrome: electrophysiological evidence of neuromuscular transmission defect in the orbicularis oculi. J Neurol Neurosurg Psychiatry. 2004
Mar;75(3):436–40.14966161
10.1136/jnnp.2003.023630PMC17389634.Chiba
A, Kusunoki
S, Obata
H, Machinami
R, Kanazawa
I.
Serum anti-GQ1b IgG antibody is associated with ophthalmoplegia in Miller Fisher syndrome and Guillain-Barré syndrome: clinical and immunohistochemical studies. Neurology. 1993
Oct;43(10):1911–7. doi: 10.1212/wnl.43.10.1911. PMID: 8413947.8413947
5.Paparounas
K.
Anti-GQ1b ganglioside antibody in peripheral nervous system disorders: pathophysiologic role and clinical relevance. Arch Neurol. 2004
Jul;61(7):1013–6. doi: 10.1001/archneur.61.7.1013. PMID: 15262730.15262730
6.Ryu
WY, Kim
YH, Yoon
BA, Park
HT, Bae
JS, Kim
JK.
Pattern of Extraocular Muscle Involvements in Miller Fisher Syndrome. J Clin Neurol. 2019
Jul;15(3):308–312. 10.3988/jcn.2019.15.3.30831286701
PMC6620438

83. **Bilateral optic neuritis and transverse myelitis with overlapping MOG-IgG and Lyme seropositivity**

João Mendes

Department of Ophthalmology, Hospital do Espírito Santo de Évora, Évora, Portugal.

**Introduction**: Myelin oligodendrocyte glycoprotein antibody-associated disease (MOGAD) can present with optic neuritis and/or transverse myelitis, mimicking infectious or other autoimmune demyelinating conditions. False-positive Lyme serology, particularly isolated IgM reactivity, is a known diagnostic pitfall that may lead to unnecessary antibiotic treatment.

We report two cases of central nervous system demyelination with concurrent MOG-IgG and Borrelia burgdorferi IgM seropositivity and review CDC criteria for accurate Lyme disease adjudication.

**Case Report**: A 55-year-old woman developed bilateral optic neuritis with positive serum MOG-IgG and Borrelia IgM but no IgG seroconversion or cerebrospinal fluid (CSF) abnormalities. She responded to high-dose corticosteroids, with residual dyschromatopsia.

A 28-year-old pregnant woman presented with acute transverse myelitis, CSF pleocytosis, weakly positive Borrelia IgM, and high-titer MOG-IgG. She improved following corticosteroid therapy and empiric ceftriaxone. In both cases, clinical and paraclinical features were consistent with MOGAD rather than neuroborreliosis, and the isolated IgM positivity was likely false-reactive.

**Discussion**: Dual MOG-IgG and Lyme IgM seropositivity may occur and represents a diagnostic challenge. According to CDC guidelines, IgM reactivity is only valid within 30 days of symptom onset and requires at least 2 of 3 specific Western blot bands; beyond this timeframe, IgM testing is unreliable and prone to cross-reactivity.

These cases underscore the importance of strict adherence to CDC Lyme serologic criteria to prevent misdiagnosis and inappropriate antibiotic use in MOGAD.

Unique to these cases is the coexistence of MOG-IgG positivity and isolated Lyme IgM reactivity, emphasizing the need for careful interpretation of infectious serologies in autoimmune demyelination.

**Disclosures**: The authors report no conflicts of interest.

85. **A collaborative approach to chronic dizziness or persistent postural-perceptual dizziness (3PD) and vestibular migraine: The Utah chronic dizziness collaborative (UCDC) and dizzy school**

Judith Warner,^1,2^ Kathleen Digre,^1,2^ Sravanthi Vegunta,^1^ Meagan Seay,^1,2^ Janene Holmberg,^3^

^1^Department of Ophthalmology and Visual Science, Moran Eye Center, University of Utah, Salt Lake City, UT, USA; ^2^Department of Neurology, University of Utah, Salt Lake City, UT, USA; ^3^Intermountain Dizziness and Balance Center, Murray, UT, USA

**Introduction**: Dizziness is a common complaint accounting for 5.6 million clinic visits in the U.S. yearly. Up to 30% of cases develop chronic dizziness or Persistent Postural-Perceptual Dizziness (3PD). 3PD is a medical condition defined by a persistent feeling of dizziness or unsteadiness following a triggering event and is associated with reduced quality of life. Vestibular Migraine (VM), a separate entity, frequently overlaps with 3PD, but is also a separate entity. Although vestibular testing is often non-explanatory, there is growing definable pathophysiology related to maladaptation of central networks responsible for space/motion perception. Sufferers often see multiple physicians, undergo costly inconclusive tests, ineffective rehabilitation, and experience suboptimal outcomes. The Utah Chronic Dizziness Collaborative (UCDC) was established in 2021 to develop a multidisciplinary approach to evaluation and treatment.

Description: The UCDC held monthly virtual meetings from 2021-2023 with vestibular therapists, audiologists, physicians, psychologists, and a wellness coach. UCDC consulted with experts in the field of dizziness and 3PD as invited speakers. The team methodically reviewed literature regarding chronic dizziness to determine how to effectively evaluate and treat patients. The literature review was synthesized into quality of evidence tables and best practice recommendations were made for both 3PD and VM.

**Conclusions:** The UCDC recommends that patients referred for chronic dizziness undergo screening for medical conditions by referring providers, and if indicated, have evaluation with vestibular testing. Co-morbid vestibular and neurologic disorders are sought, including VM. For 3PD, based on available evidence, treatments include 1) customized vestibular rehabilitation with physical therapists, 2) low dose serotonin-based medication, and 3) cognitive behavioral therapy. For VM, treatment directed at migraine and its co-morbidities is preferred, along with vestibular therapy tailored to the condition. As education is critical to success, we created the Dizzy School website for patient and provider information. The Dizzy School website currently receives 2000 unique views per month.

The UCDC is actively working on dissemination of this approach through local, national, and international meetings and workshops, with thousands reached thus far.

Supported in part by an Unrestricted Grant from Research to Prevent Blindness, New York, NY, to the Department of Ophthalmology & Visual Sciences, University of Utah.

86. **Dural veinous thrombosis case series**

Karima Nesnas^1^, Sneha Melmane^1^, Thushara Mannampat^1^

^1^Frimley Health NHS Foundation Trust, UK

**Background:** Dural venous sinus thrombosis (DVST) is an uncommon but potentially life‑threatening condition with a wide spectrum of clinical manifestations. Its ability to mimic other neurological and ophthalmic disorders often leads to delayed diagnosis. Early recognition is essential to prevent serious complications.

**Methods:** We describe a case series of three patients with DVST, each presenting with distinct initial symptoms. The cases highlight the heterogeneity of clinical presentation and the diagnostic challenges encountered.

**Case 1:** A 34‑year‑old woman with inflammatory bowel disease on Adalimumab and a history of smoking presented with headache and malaise while being treated for mastoiditis and otitis. Lumbar puncture demonstrated raised intracranial pressure, and ophthalmologic assessment revealed bilateral disc oedema. MRI confirmed extensive transverse sinus thrombosis.

**Case 2:** A 52‑year‑old man presented with an inferior visual field defect and unilateral disc oedema, initially diagnosed as non‑arteritic anterior ischemic optic neuropathy (NAION). Three weeks later, he developed bilateral disc oedema, prompting further investigation. MRI revealed left sigmoid sinus thrombosis.

**Case 3:** A 34‑year‑old pregnant woman reported transient episodes of dark visual shadows that subsequently improved. Examination showed bilateral grade 4 papilledema. MRI and MRV confirmed extensive cerebral venous sinus thrombosis.

**Conclusion:** This case series underscores the diverse and often deceptive presentations of DVST, ranging from raised intracranial pressure and visual disturbances to atypical optic nerve findings. Missed or delayed diagnosis can result in significant morbidity. Clinicians should maintain a high index of suspicion, particularly in patients with known risk factors such as inflammatory disease, pregnancy, infection, or pro‑thrombotic states, to facilitate timely diagnosis and appropriate management.

91. **Reality check: Virtual tools in functional vision disorders**

**Functional and structural visual loss: Insights from virtual reality perimetry in five complex cases**

Manisha Kotay^1^and Eric Eggenberger^1^

^1^Mayo Clinic Florida Department of Ophthalmology, FL, USA

**Purpose**: Describe the utility of virtual perimetry in workup of patients with functional visual loss (FVL).

**Methods**: Retrospective review of 9 patients ages 19-60 years presenting with visual defects on automated perimetry due to functional visual loss. The patients presented with varied visual symptoms including abnormal automated perimetry in contrast to intact confrontational perimetry and normal structural exam. Virtual perimetry (neos, machineMD) was performed and compared to automated perimetry.

**Results**: While standard automated perimetry revealed varied defects, VR perimetry documented largely normal visual fields, one patient had a deficit inconsistent with AF. 2 patients had monocular deficits on perimetry and normal virtual perimetry; 2 patients with monocular deficit on automated perimetry showed a homonymous pattern on virtual;1 patients with right homonymous defects on automated perimetry had normal virtual perimetry; 2 patient showed bilateral constriction AF with normal VR; 1 had central scotoma OU with normal VR; and 1 had variable deficits measured over time on AF with normal VR.

**Conclusion**: VR-based perimetry provides a novel tool for assessing visual function and may be a valuable adjunct in evaluating patients with suspected functional visual loss. The usefulness of virtual perimetry in functional patients may be related to a binocular paradigm with monocular data acquisition.

**Keywords**: Functional visual loss, virtual reality perimetry, visual field, optical coherence tomography, photoreceptor dysfunction

**Introduction**: Functional visual loss (FVL) presents a significant challenge in clinical practice, characterized by visual impairment without an identifiable organic cause. If misdiagnosed it can lead to superfluous and invasive workup and treatment. Recent studies highlight the complex interplay between psychological factors and neurological conditions in the manifestation of FVL. Bennett et al. discuss assessment strategies for visual function in pediatric populations, emphasizing the need for comprehensive evaluation [1].

In response to these challenges, virtual reality (VR) perimetry has emerged as a promising tool for assessing visual function in individuals with FVL. Malyukova et al. describe the efficacy of VR headsets in perimetry testing, noting their potential to enhance diagnostic accuracy, patient engagement, and comfort [2]. Zhao et al. investigated a novel remote perimetry application in a VR environment, demonstrating feasibility for functional visual assessment in community-based and primary care settings [3].

These advancements underscore the importance of integrating innovative technologies like VR perimetry into the assessment of FVL. By bridging the gap between subjective symptoms and objective testing, VR perimetry holds promise for refining diagnostic approaches and tailoring interventions for individuals experiencing functional visual impairments.

**Case Presentations**

**Case 1**

- **Age/Sex**: 45-year-old with history of CSF leak, VP shunt placement presenting with episodic blur OD>OS, temporal field constriction OD, starbursts
- **Examination**: VA 20/20 OU; slit-lamp/fundus exam normal; OCT normal
- **Visual Fields**: AF: right homonymous; VR: intact OU
- **Ancillary**: MRV with transverse sinus stenosis; LP opening pressures 14 cm H_2_O

**Case 2**

- **Age/Sex**: 23-year-old history of lifelong headaches, autonomic spells, EDS; prior LP opening pressure 34 cm H_2_O presenting with episodic blur 2/week; no optic disc changes
- **Examination**: VA 20/20 OU; OCT normal
- **Visual Fields**: AF: loss OD and nasal OS; VR: normal

**Case 3**

- **Age/Sex**: 19-year-old with history of chronic peri-OU headaches, allergic fungal sinusitis; prior accommodative esotropia presenting with episodic temporal spots OS; blurring
- **Examination**: VA 20/20 OU; OCT normal
- **Visual Fields**: AF: Temporal paracentral defect OS; normal OD; VF: normal
- **Ancillary**: Asymmetric photoreceptor dysfunction R>L, scotopic and photopic responses affected

**Case 4**

- **Age/Sex**: 42-year-old with history of sinus/fungal surgery; right hemicranial headaches presenting with episodic blur OD
- **Examination**: VA 20/20 OU; OCT normal
- **Visual Fields**: AF: diffuse deficits OD and VF: normal

**Case 5**

- **Age/Sex**: 35-year-old with history of T2DM, transgender male; acute onset OS visual decline presenting with blur OS, episodic hemifield loss
- **Examination**: VA 20/20 OD, LP OS; OCT normal; no RAPD
- **Visual Fields**: AF: left hemianopia; VF: left homonymous

**Case 6**

- **Age/Sex**: 19-year-old lifelong headaches, autonomic spells, EDS, blur OU
- **Examination**: VA 20/20 OD, LP OS; OCT normal; no RAPD
- **Ancillary**: prior LP opening pressure 34 cm H_2_O presenting with episodic blur 2/week
- **Visual Fields**: AF: loss OD and nasal OS; VF: normal

**Case 7**

- **Age/Sex**: 61-year-old history of T2DM, HTN, HLD, OSA, 70lb weight loss, Carotid stenosis with intermittent blur OU
- **Examination**: VA 20/20 OD, LP OS; OCT normal; no RAPD
- **Ancillary**: ERG: normal
- **Visual Fields**: AF: central scotoma OU; VF: normal

**Case 8**

- **Age/Sex**: 53-year-old with history of Pseudotumor cerebri, OSA with headache and blur OU
- **Examination**: VA 20/20 OU; OCT normal; no RAPD
- **Visual Fields**: AF: constriction OU; VF: relatively intact virtual perimetry with only 2 abnormal sites OU

**Case 9**

- **Age/Sex**: 38-year-old with history of generalized anxiety disorder/panic. Depressive disorder. Fibromyalgia. High myopia. T2DM
- **Examination**: VA20/40; 20/30; OCT normal; no RAPD
- **Visual Fields**: AF: constriction OU; VF: normal

**Discussion**: VR perimetry can detect subtle or functional deficits missed by conventional testing. This may be due to the complex nature of the patient interface with perimetry. Case 5 demonstrates VR’s ability to reveal a left hemianopia not captured on Humphrey fields. If the patient was even subconsciously overcoming the objectivity of the automated field, they were not able to reproduce the deficit on virtual perimetry. This is important in determining next steps in a patient with concern for structural etiology of deficit. Case 6, a patient with idiopathic intracranial hypertension and likely functional overlay demonstrates utility in differentiating functional field loss. The ERG in Case 3 shows how multimodal evaluation can distinguish structural from functional disease. Integration of VR testing enhances patient engagement and objective monitoring. These cases illustrate the spectrum of FVL presentation, from isolated functional defects to mixed structural-functional processes.

**Conclusion**: Virtual reality perimetry is a valuable adjunct in evaluating patients with suspected functional visual loss. It provides objective, reproducible data, helping to differentiate functional from structural pathology and guiding management strategies.

**Table 1.** Visual Acuity and OCT Findings

Case	VA OD	VA OS	OCT OD	OCT OS	Notes
1	20/20	20/20	Normal	Normal	Traditional VF defect OD
2	20/20	20/20	Normal	Normal	VF constriction OU
3	20/20	20/20	Normal	Normal	Temporal paracentral VF defect OS
4	20/20	20/20	Normal	Normal	Automated diffuse deficits OD Normal VF on confrontation OU
5	20/20	LP	Normal	Normal	Automated hemianopia OS
6	20/20	20/20	Normal	Normal	Automated loss OD and nasal OS; Virtual normal
7	20/20	20/20	Normal	Normal	Automated scotomas OU, virtual normal
8	20/20	20/20	Normal	Normal	Automated constriction OU, virtual normal
9	20/30	20/40	Normal	Normal	Automated constriction

**Table 2.** Visual Fields (VR vs Traditional)

Case	Traditional Perimetry	VR Perimetry	Notes
1	Right homonymous inferior quadrantanopia	Intact OU	Functional vision loss
2	Generalized constriction OU	Normal OU	Functional vision loss
3	Temporal paracentral OS	Normal OD, paracentral OS	Functional vision loss
4	Diffuse deficits OD	Normal	Functional vision loss
5	Automated hemianopia OS	Left homonymous OS	Functional vision loss
6	Automated loss OD and nasal OS	Normal	IIH with functional overlay
7	Automated scotomas OU	Normal	Functional vision loss
8	Constriction OU	Normal	Functional vision loss
9	Constriction OU	Normal	Functional vision loss

References1.Raviskanthan
S, Wendt
S, Ugoh
PM, Mortensen
PW, Moss
HE, Lee
AG.
Functional vision disorders in adults: a paradigm and nomenclature shift for ophthalmology. Surv Ophthalmol. 2022
Jan-Feb;67(1):8–18. doi: 10.1016/j.survophthal.2021.03.002. Epub 2021 Mar 15. PMID: 33737039; PMCID: PMC9159904.33737039
PMC91599042.Malyukova
I, et al. Virtual and augmented reality for simulating visual impairments. *npj Digit Med*. 2019;2:22.31304369
3.Zhao
B, et al. Remote virtual reality perimetry for functional visual assessment. *J Ophthalmol*. 2023;138(12):1024–1035.

97. **When vision affects driving: Healthcare professionals’ approaches to discussing fitness to drive**

L. R. Hepworth^1,2^, J. Wyche^1^, C. Howard^1,2^

^1^Institute of Population Health, University of Liverpool, Liverpool, UK; ^2^Northern Care Alliance NHS Foundation Trust, Salford, UK

**Background**: Many individuals with visual impairments do not currently receive consistent, reliable information from healthcare professionals (HCPs) regarding driving regulations. The study aim was to explore current knowledge and practices regarding vision guidelines for driving amongst HCPs and how this is relayed to patients.

**Methods**: An online survey was distributed to HCPs who advise patients with visual impairments on driving vision regulations. Two open-ended questions asked how respondents determine whether a patient drives and how they communicate to patients that they do not meet the vision standards for driving. Responses were analysed using categorisation or inductive content analysis to identify overarching themes, followed by quantitative content analysis.

**Results**: A total of 411 responses were included in the analysis from HCPs across all four nations of the UK. Over two-thirds had been qualified for >10 years. Driving status was mostly determined by a direct question (63%), with a further 16% asking whether patients were current drivers. Less frequently, HCPs assessed driving status through contextual questions about recent driving behaviour or travel to appointments (2%). More specific questions, such as type of vehicle driven (22%), and details about the driver’s licence type and license validity (20%) were reported. Almost half of respondents (46%) reported asking only one question, most often a direct enquiry about driving status; those asking multiple questions typically combined this with questions on licence, vehicle, employment, or future driving intentions.

How HCPs reported informing patients that they did not meet driving vision standards generated three themes; the professional’s manner of delivery, elements of the patient–HCP interaction, and whether the professional took action or advised the patient on what to do next. Only 38% of respondents reported directly advising patients not to drive when vision standards for driving were not met. These discussions were most commonly apologetic or framed around hope of returning to driving, although around a fifth were coded as blunt or unsupportive. Driving regulations were referenced in 27% of responses, with 9% explicitly explaining how patients failed to meet them. Less than half (40%) informed patients of their responsibility to self-report to driving regulatory bodies.

**Conclusions:** These findings suggest a lack of consistency and confidence in discussing driving cessation related to visual impairment with patients. This has important public safety implications, particularly given that in less than half of cases patients were explicitly told to stop driving, despite not meeting legal vision driving standards. More training, clearer clinical guidance, and provision of resources may help better equip healthcare professionals to communicate driving advice that is consistent, accurate and delivered in an appropriate, sensitive way, thereby improving patient understanding of their fitness to drive and overall road safety.

103. **Post-retinal visual pathway abnormalities and visual function in Friedreich Ataxia**

Lucilla Barbano^1^, Lucia Ziccardi^1^, Carmen Dell’Aquila^1^, Mattia D’Andrea^2^, Antonio Di Renzo^1^, Gaspare Colacino^3^, Carlo Casali^4^, Vincenzo Parisi^1^

^1^IRCCS - Fondazione Bietti, Rome, Italy; ^2^Department of Sense Organs, Faculty of Medicine and Dentistry, Sapienza University of Rome, Rome, Italy; ^3^UOC Oftalmologia- San Giovanni Evangelista Hospital Tivoli, Tivoli, Italy; ^4^Department of Medical and Surgical Sciences and Biotechnologies, Sapienza University of Rome, Italy;

**Introduction**: Although the majority of Friedreich Ataxia (FA) patients have no ocular symptoms, the visual system is often involved in this disorder with optic nerve or retinal involvement (optic neuropathy, optic radiation impairment and retinitis pigmentosa). The function of the whole visual pathway, from the photoreceptors to the cerebral cortex, can be assessed by Visual Evoked Potentials (VEP) responses that may be influenced by macular or retinal ganglion cells (RGC) impairment.

In FA patients, VEP abnormalities have been detected, but it is unclear whether these abnormalities could be ascribed to a dysfunction occurring at the level of macular or RGC and/or at the level of the post-retinal visual pathways.

This study aim to evaluate RGC function and post-retinal neural conduction in FA patients, excluding those with macular morpho-functional impairment assessed by multifocal electroretinogram (mfERG) and Optical Coherence Tomography (OCT). Secondary aim is the study of the relationship between visual pathway dysfunction, best corrected visual acuity (BCVA), age and disease duration.

**Methods**: Twenty FA patients (providing 20 eyes, mean age 31.2 ± 7.3 years) and twenty age-matched healthy controls (providing 20 eyes) were enrolled in this observational case series study. FA patients were divided into two groups based on BCVA: FA1 (reduced BCVA) and FA2 (normal BCVA). All subjects underwent comprehensive ophthalmological and neurological examinations. Macular structure and function were assessed using OCT and mfERG. RGC function and visual pathway conduction were evaluated through simultaneous pattern electroretinogram (PERG) and VEP recordings using 60′ and 15′ checkerboard stimuli. Retinocortical time (RCT) was calculated as the difference between VEP P100 and PERG P50 implicit times. Statistical analyses included ANOVA, Tukey post-hoc tests and Pearson tests.

**Results**: All FA eyes showed normal morpho-functional macular status, since OCT and mfERG mean values were within normal limits. Mean PERG 15’parameters showed only mild P50-N95 amplitude reduction in a subset of eyes (50%). By contrast, mean VEP P100 implicit time (IT) showed significant increase in FA patients, particularly in the FA1 group. Compared with controls, FA patients showed significantly delayed VEP IT and increased RCT values for both stimulus sizes (p < 0.05). These abnormalities were more pronounced in FA1 than FA2 Group. RCT increase was detected in most FA eyes, even in the absence of macular or RGC dysfunction. In FA1 patients, VEP IT and RCT values were significantly correlated with BCVA reduction (p < 0.01), whereas no significant correlations were found with age or disease duration.

**Discussion**: In FA, visual dysfunction underlies primarily impairment of post-retinal neural conduction rather than macular dysfunction. Increased RCT indicates delayed neural transmission beyond the retina and represents a sensitive electrophysiological marker of visual pathway involvement in FA. The significant correlation between VEP/RCT abnormalities and reduced BCVA suggests a functional impact of post-retinal dysfunction on visual performance.

**Disclosures**: The authors report no conflicts of interest.

114. **Age-related changes in the biomechanical properties of the human optic nerve sheath**

Michael Dattilo,^1,2^ Anna Silverio,^1^ Dennis Jiang^1^, Andrew Feola^1,2,3^

^1^Emory Eye Center, Emory University School of Medicine, Atlanta GA, USA; ^2^Atlanta Veterans Affairs Medical Center, Decatur, GA 30033; ^3^Georgia Institute of Technology, Atlanta, GA, USA

**Introduction**: Although optic nerve sheath (ONS) structural changes have been described in several ophthalmic disorders, such as idiopathic intracranial hypertension (IIH)^1,2^, ONS biomechanical properties remain poorly understood, particularly in humans where there are limited studies. Although several studies have described ONS biomechanical properties^4-6^, the effect of age was not assessed. Here, we report human ONS (hONS) biomechanical properties obtained using unconfined microcompression testing.

**Methods**: The ONS from 3 male humans (ages 20, 36, and 63 yo) were dissected from the ON. A cylindrical sample of hONS (1 mm diameter), obtained using a biopsy punch, was placed on a custom-made unconfined microcompression device platform in a PBS fluid bath at room temperature. Samples were pre-loaded with a compressive load of 2 mN, allowed to equilibrate, and then subjected to a 3-step stress relaxation protocol at 5%, 10%, and 15% compressive strain. Data from each step were analyzed independently using linear viscoelastic theory to determine hONS material properties.

**Results**: The hONS stiffness was 0.88+/-0.1 N/mm for the 2 younger subjects (20 and 36 yo) and 0.045 N/mm for the 63 yo subject. The stress after relaxation (Maxwell Ideal E_inf_) was 8.13+/-1.55 kPa for younger subjects and 4.10 kPa for the 63 yo subject. The time to relaxation (Maxwell Ideal Tau) was 23.31+/-12.42 seconds for the younger subjects and 9.00 seconds for the 63 yo subject.

**Conclusions**: Younger subjects ONSs tended to be stiffer, relax more slowly, and retain a higher stress after relaxation compared to the older subject ONS. Although we have a small sample size, this data suggests that human ONS biomechanical properties change with age. Further understanding of this difference may provide insights into the pathophysiology of IIH and may lead to the development of novel IIH treatments.

References1.Bidot
S, Bruce
BB.
Update on the Diagnosis and Treatment of Idiopathic Intracranial Hypertension. Semin Neurol
2015;35:527–38.26444398
10.1055/s-0035-15635692.Bidot
S, Saindane
AM, Peragallo
JH, Bruce
BB, Newman
NJ, Biousse
V.
Brain Imaging in Idiopathic Intracranial Hypertension. J Neuroophthalmol
2015;35:400–11.26457687
10.1097/WNO.00000000000003033.Lee
C, Rohr
J, Sass
A, et al. In vivo estimation of optic nerve sheath stiffness using noninvasive MRI measurements and finite element modeling. Journal of the Mechanical Behavior of Biomedical Materials
2020.10.1016/j.jmbbm.2020.103924329572194.Demer
JL.
Optic Nerve Sheath as a Novel Mechanical Load on the Globe in Ocular Duction. Invest Ophthalmol Vis Sci
2016;57:1826–38.27082297
10.1167/iovs.15-18718PMC48495495.Raykin
J, Forte
TE, Wang
R, et al. Characterization of the mechanical behavior of the optic nerve sheath and its role in spaceflight-induced ophthalmic changes. Biomech Model Mechanobiol
2017;16:33–43.27236645
10.1007/s10237-016-0800-7

143. **European Stroke Organisation vision guidelines. The recommendations for assessment and rehabilitation for visual disorders**

Fiona Rowe^1^, Lauren Hepworth^1^, Maria Begona Coco-Martin^2^, Celine Gillebert^3^, Luis Leal-Vega^2^, Anja Palmowski-Wolfe^4^, Eleni Papageorgiou^5^, Stephen Ryan^6^, Karolina Skorkovska^7^, Anne Hege Aamodt^6^

^1^University of Liverpool, UK; ^2^University of Valladolid, Spain; ^3^KU Leuven, Belgium; ^4^University of Basel, Switzerland; ^5^University Hospital of Larissa, Greece; ^6^Oslo University Hospital, Norway; ^7^Masaryk University, St Ann University Hospital Brno, Czech Republic

**Introduction**: Visual problems are frequent following stroke. The purpose of the European Stroke Organisation guideline is to provide evidence-based recommendations to assist clinicians in decision making around diagnosis and treatment on visual problems after stroke. We report the recommendations for visual disorders.

**Materials-Methods**: Through international discussion, and with independent peer review, we identified PICOs (Population/Intervention/Comparison/Outcomes) for review. For each, we performed a systematic literature review search with subsequent title/abstract screen followed by full text screen, completed independently by two authors. Data was extracted and risk of bias completed for each included study. Quality of evidence for each outcome measure was evaluated by GRADE (Grading quality of evidence and strength of recommendations).

**Results**: The Vision Guideline comprises 13 PICOs for screening and management of visual disorders for adult stroke survivors. Early vision screening should be undertaken to detect these disorders. This is feasible/acceptable within 3-4 days post onset of stroke; most can be assessed within 1 week. Screening should be undertaken by specialist eye team assessment or at least by using a validated vision screening tool. Screening improves the detection rate of presence of visual disorders whilst specialist visual assessment further improves the accuracy of detection of visual impairment. We suggest individualised intervention targeted at the specific type of visual problem that has arisen. We suggest referral to specialist eye services for the targeted management of these disorders.

**Conclusions**: The intention is that these guidelines will be of use to any clinician working with stroke survivors and aid standardisation of stroke/vision care in Europe.

**Disclosures:** In relation to this Guideline: FR (Funded research – NIHR, Norwegian Research Foundation, Stroke Association, Fight for Sight), LH (Funded research – Stroke Association), MBCM (None), CG (None), LLV (None), APW (None), EP (None), SR (None), KS (None), AHA (Funded research – Medtronic, Boehringer Ingelheim).

145. **Temporal bioavailability of intranasal recombinant human nerve growth factor in ocular and neural tissues in mice**

Rubina Novelli^1^, Annamaria Cimini^2^, Laura Brandolini^1^, Andrea Aramini^1^, Marcello Allegretti^1^

^1^Research & Early Development, Dompé farmaceutici S.p.A., Naples, Italy; ^2^Department of Life, Health and Environmental Sciences, University of L’Aquila, L’Aquila, Italy

**Introduction:** Recombinant human nerve growth factor (rhNGF) has shown therapeutic potential for neurodegenerative diseases. Intranasal administration offers a non-invasive method to bypass the blood-brain barrier, allowing for direct delivery to central nervous system (CNS) tissues. This study aimed to evaluate the bioavailability of rhNGF in the optic nerve, retina, brain, and cochlear perilymph following a single intranasal administration in mice.

**Methods:** A total of 15 male C57BL/6 mice (10 weeks old) were randomized into four groups: vehicle and sampling at 12 hours (h) post-administration (n=3), rhNGF-treated and sampling at 12h (n=4), 24h (n=4), or 48h (n=4) post-administration. Vehicle or rhNGF 2 mg/mL was delivered intranasally (10 µL/animal). The mice were euthanized at 12h, 24h, or 48h post-administration. Optic nerve, retina, brain, and cochlear perilymph were collected post-mortem, homogenized, and analyzed for rhNGF concentration using an ELISA. Mean ± SEM rhNGF values are reported. Statistical significance was assessed using t-tests to compare the rhNGF-treated groups to the vehicle group (*p* < 0.05).

**Results:** In optic nerve tissue, a significant increase in rhNGF was measured at 12h (463.0 ± 52.1 pg/mL) compared to vehicle (67.4 ± 21.3 pg/mL; *p* < 0.05). rhNGF concentrations at 24h (117.0 ± 10.3 pg/mL) and 48h (71.9 ± 9.1pg/mL) post-treatment were not significantly increased.

In retinal tissue, a significant increase in rhNGF was measured at 12h and 24h (89.8 ± 8.4 pg/mL and 242.4 ± 24.7 pg/mL, respectively) compared to vehicle (5.3 ± 2.6 pg/mL at 12h; both *p* < 0.05). The rhNGF concentration at 48h (61.0 ± 8.9 pg/mL) post-treatment was not significantly increased.

In brain tissue, a significant increase in rhNGF was measured at 12h (449.9 ± 45.0 pg/mL) compared to vehicle (82.2 ± 3.4 pg/mL; *p* < 0.05). rhNGF concentrations at 24h (140.7 ± 16.3 pg/mL) and 48h (79.9 ± 8.5 pg/mL) post-treatment were not significantly increased.

In cochlear perilymph tissue, a significant increase in rhNGF was measured at 12h (832.2 ± 73.0 pg/mL) compared to vehicle (7.3 ± 1.4 pg/mL; *p* < 0.05). rhNGF concentrations at 24h (92.8 ± 17.7 pg/mL) and 48h (6.8 ± 2.5 pg/mL) post-treatment were not significantly increased.

**Discussion:** A single intranasal administration of rhNGF led to a significant increase in rhNGF concentrations in the optic nerve, retina, brain, and cochlear perilymph when compared to vehicle, reaching a peak concentration at 12h post-treatment in the optic nerve, brain and cochlear perilymph, and 24h post-treatment in the retina. These findings demonstrate that intranasally administered rhNGF enables effective distribution to visual, CNS, and auditory tissues. Studies evaluating intranasal rhNGF efficacy in non-arteritic anterior ischemic optic neuropathy and CNS disorders are ongoing.

**Disclosures:** Rubina Novelli – Dompé farmaceutici S.p.A., employee. Annamaria Cimini – None to report

Laura Brandolini - Dompé farmaceutici S.p.A., employee. Andrea Aramini - Dompé farmaceutici S.p.A., employee. Marcello Allegretti - Dompé farmaceutici S.p.A., employee

150. **A severe case of Tolosa-Hunt Syndrome: Differential diagnosis and review of current treatment options.**

Sarah Marti^1,2^, Konrad P. Weber^3^

^1^Neurologische Praxis Zollikerberg, Zollikerberg, Switzerland; ^2^Consultant Neurologist, Spital Uster, Uster, Switzerland; ^3^Neurology Department, Zurich University Hospital, Zurich, Switzerland

A 55-year-old obese, but otherwise healthy, female presented to the emergency department of a district hospital with a first-ever set of symptoms: progressive throbbing, right-sided orbital pain for a few days, followed by oblique double vision with gaze straight ahead, and in all other gaze directions except to the right, as well as right-sided ptosis for one day. She did not report fever, phono- or photophobia, nausea or tearing, nasal congestion or conjunctival injection. She had not been diagnosed with migraine before. The clinical exam on admission revealed almost complete right-sided ptosis and right-sided partial, pupil-sparing oculomotor (IIIrd) nerve palsy. There were no other focal neurological deficits. Magnetic resonance (MR) tomography of the head showed a contrast-enhancing lesion in the right cavernous sinus extending to the orbital apex. A thorough diagnostic workup, consisting of laboratory tests (CRP, blood cell count, screening for vasculitis, ACE, TSH, B12, HIV, Lyme disease), CSF and whole-body PET-CT scan, did not reveal any specific underlying cause. A neurosurgical evaluation of the lesion was regarded as being too invasive. Eventually, a diagnosis of Tolosa-Hunt Syndrome was made based on the current diagnostic criteria and treatment with oral corticosteroids (1mg/kg KG) was started. After only three days, pain and IIIrd nerve palsy had resolved completely. However, tapering of corticosteroids, despite being executed over several weeks, twice led to relapses of pain and IIIrd nerve palsy, when the daily dosage was reduced to 10 mg. About nine months after onset of the disease, the patient received a single infusion of Rituximab (375 mg/m^2^body surface). After this, corticosteroids could be tapered without any further relapses of either orbital pain or IIIrd nerve palsy. We discuss the differential diagnosis of painful orbitopathies and the different treatment options for Tolosa-Hunt Syndrome, in addition to the overlap with IgG4-related orbital disease.

152. **The ultrasound dopplerography in mixed astigmatism arising against the background of central nervous system pathologies**

Sevinj Salmanova^1^, Elmar Kasimov^1^, Saida Gadzhiyeva^1^, Afet Mammadzada^1^

^1^National Center of Ophthalmology by academician Zarifa Aliyeva, Baku, Azerbaijan

**Introduction**: The visual system represents an integral part of the central nervous system (CNS), and its functional state largely depends on the integrity of neural regulation and vascular supply. Pathologies of the CNS are frequently associated with ophthalmological disorders, including refractive abnormalities, accommodative dysfunctions, and alterations in ocular blood circulation. These interactions form the basis of growing interest in the field of neuro-ophthalmology, particularly in the study of mechanisms linking neurological disorders with visual impairment. Among these conditions, mixed astigmatism represents a complex refractive anomaly characterized by the presence of both myopic and hyperopic components in different meridians of the same eye, often leading to significant impairment of visual acuity and binocular vision, especially when occurring in early childhood. While the optical aspects of mixed astigmatism have been extensively investigated, its vascular and neurogenic components remain insufficiently.

**Purpose**: Development of a new method for studying the state of hemodynamic parameters of the ophthalmic artery in the mixed astigmatism developed against the background of the central nervous system (CNS) pathologies.

**Materials and Methods**: It the National Centre of Ophthalmology named after Academician Z. Aliyeva dopplerographic research studies were carried out on 11 patients aged 4–7 years (22 eyes) with a diagnosis of mixed astigmatism against the background of the CNS pathology. The studies were mainly carried out on the NEMIO XG SSA-580A system of colour a gray school triplex ultrasound examination (US) of “TOSHIBA” firm according to the method by Kasimov E.M., Gadzhiyeva S.A., and Salmanova S.Z. The study of blood flow in the eyeball vessels, primarily in the ophthalmic artery (OA), was carried out using B-mode, color Doppler mapping (CDM), power doppler mapping (EDM), a power doppler (PD). In the ophthalmic artery hemodynamic parameters were studied: maximum systolic velocity (Vmax), minimum diastolic velocity (Vmin) and resistance index (RI).

**Results and Discussion**: The results of our studies show that in young children with CNS pathology, compared with norm, changes in the hemodynamic parameters of blood flow are observed (diagrams 1 and 2).

As can be seen from the diagrams in the right eye (OD) according to average blood flow indicators the maximum systolic velocity (Vmax) is statistically significantly reduced compared to the norm and amounts to 31.66 ± 0.74 sm/s (p < 0.01) (norm - 36.19 ± 0.67 sm/s). The minimum diastolic velocity (Vmin) also statistically decreases to 5.28 ± 0.29 sm/s (p < 0.01) (norm 9.52 ± 0.32 sm/s). The resistance index (RI) statistically significantly (p < 0.01) increases to 0.83 ± 0.02 and exceeds normal values (norm 0.73 ± 0.01).

Analysis of the left eye (OS) demonstrates that, according to mean blood flow values, the maximum systolic velocity (Vmax) is statistically significantly reduced to 33.05 ± 0.80 cm/s (p < 0.05) compared with normal values (norm - 36.19 ± 0.67 sm/s). The minimum diastolic velocity (Vmin) is statistically significantly reduced to 6.48 ± 0.34 sm/s (p < 0.01) (norm 9.52 ± 0.32 sm/s). The resistance index (RI) is statistically significantly increased (p < 0.01) to 0.82 ± 0.01, exceeding normal values (norm 0.73 ± 0.01).

Thus, one of the branches of the internal carotid artery which is one of the brachiocephalic arteries and goes to the brain on the both sides of the neck, is the ophthalmic artery. This means that the deterioration of hemodynamic parameters of blood flow occurs not only in the a. ophthalmica pool but primarily due to deterioration of blood circulation in the circle of Willis of the brain, that is, an increase in the peripheral influence on vascular blood and, as consequence, a decrease in both linear velocities which leads to a weakening of the blood supply to the eyeball and finally to the appearance of mixed astigmatism which is a refractive anomaly in both eyes.

**Conclusions**:

1. The development of a new technique for triplex Doppler ultrasound opens up wide opportunities for expanding experimental and clinical research and is indispensable in diagnostics.
2. To prevent complications caused by dysfunction of the CNS in the eyeball, it’s necessary to initially use doppler ultrasound as a highly imperative method.
3. Triplex ultrasound dopplerography allows during diagnosis in the a. ophthalmica pool to deremine the state of hemodynamic parameters in patients with pathology before treatment, after treatment and in dynamics—both during conservative therapy and during surgical intervention (with development of complications). The effectiveness of treatment should be assessed using Doppler examination.

166. **Acute Horner syndrome identified by pupillography revealing internal carotid artery dissection**

Victoria Borisova^1^, Muriel Dysli^1^

^1^Eye Clinic, Cantonal Hospital Aarau, Switzerland

**Introduction:** Acute anisocoria with ipsilateral ptosis, particularly when the pupillary asymmetry is greater in the dark, should raise suspicion for Horner syndrome and prompt urgent evaluation for potentially life-threatening causes such as internal carotid artery (ICA) dissection. Early recognition in the neuro-ophthalmology setting is critical to prevent cerebral ischemic complications.

**Case Report**: A 38-year-old woman was referred by a private ophthalmologist for evaluation of anisocoria. Five days before presentation, she noticed a smaller right pupil and a mild right upper eyelid droop when looking in the mirror. She also reported new right-sided neck pain and a pressure-like sensation in the right face. The prior weekend she had attended a sports training course and had been physically very active. A few weeks earlier, she had experienced sinusitis and an upper respiratory infection.

On examination, anisocoria was present (right < left), with a larger inter-pupillary difference in dim illumination. Both pupils were briskly reactive to light. There was a mild right ptosis. The remainder of the ocular examination was unremarkable and age-appropriate, and the neuro-ophthalmological examination showed no other deficits.

Pupillography (neos® system) demonstrated a clear right-sided dilation deficit, providing objective confirmation of oculosympathetic dysfunction; therefore, pharmacological testing for Horner syndrome was not pursued. Given the acute onset with ipsilateral neck pain, the patient was referred emergently to the neurological emergency department for etiologic work-up. Contrast-enhanced computed tomography (CT) of the head and CT angiography (CTA) revealed a short-segment mural hematoma consistent with right ICA dissection, extending from the distal cervical segment to the transition into the petrous segment. No intracranial hemorrhage or cerebral ischemia was detected.

The patient was admitted for monitoring. Antiplatelet secondary prophylaxis with acetylsalicylic acid (ASA) was initiated. Duplex ultrasound of the cervical arteries showed no perfusion-relevant stenosis. In the absence of signs suggestive of a connective tissue disorder and given the history of intense physical activity, the dissection was interpreted as likely provoked. Continued ASA therapy was recommended until imaging-confirmed healing of the dissection.

**Discussion**: This case highlights a classic “red flag” constellation for ICA dissection: acute Horner syndrome accompanied by ipsilateral neck/facial pain. Because pupillary findings may be subtle, objective testing (e.g., pupillography) can substantiate clinical suspicion, accelerate emergency imaging, and in selected cases obviate the need for pharmacological confirmation. Prompt vascular imaging with CTA (or magnetic resonance angiography) is essential to establish the diagnosis. Early antithrombotic treatment—either antiplatelet therapy or anticoagulation, depending on the severity, extent, and location of the dissection and the overall clinical context—aims to reduce thromboembolic risk and prevent ischemic complications. Neuro-ophthalmologists and ophthalmologists should treat painful acute Horner syndrome as an emergency until ICA dissection is excluded.

**Disclosures**: The authors report no financial or non-financial disclosures.

**Table:** Abstract Index

Paper Reference	Paper Title	Author List	Page
1	How normal is a normal disc in retrobulbar opticneuritis? OCT reveals focal thickening in the acute stage of the disease	Ana Banc, Mehdi Ounissi, Aurore Sajust De Bergues De Escalup, Cédric Lamire, Caroline Papeix, Augustin Lecler, Romain Deschamps, Dan Milea	5
2	A case of Leber Hereditary Optic Neuropathy seemingly precipitated by an electric shock	Abhijeet Yadav	6
3	Optic Perineuritis in a 17-Year-Old Patient: A diagnostic challenge	Adriana Molino, Alessia Bargagli, Francesco Sicurelli, Alessandra Rufa, Nicola De Stefano	6
5	When NAION is not NAION: Bilateral optic disc edema during semaglutide therapy	Agata Anna Wykrota	7
7	Optic disc drusen masquerading as optic neuritis: An electrophysiological pitfall	Aida Jallouli, Imen Zone Abid, Yosra Maalej, Amira Trigui	8
10	Epidemiology and clinical outcomes of traumatic optic neuropathy in Taiwan: A multicenter retrospective study	Ching-Lung Chen, Meng-Hung Lin, Yung-Kang Chen, Nai-Hua Kuo, Ching-Yun Wang	9
12	Brain MRI abnormalities along the visual pathways in subacute Leber Hereditary Optic Neuropathy: Prevalence and clinical correlations	Giulia Amore, Michele Carbonelli, Martina Romagnoli, Caterina Tonon, Raffaele Lodi, Valerio Carelli, Chiara La Morgia	10
15	Differentiation between arteritic and nonarteritic anterior ischemic optic neuropathy using optical coherence tomography	Andreas Worm Bendtsen, Michael Stormly Hansen, Oliver Niels Klefter, Steffen Hamann	11
22	Clinical features and Genotype-Phenotype correlations in Bosch-Boonstra-Schaaf Optic Atrophy Syndrome	Samia Ghazali, Reda Ghazali, Veeral Shah, Christian Schaaf	12
23	Structure-function analysis of OPA1-associated autosomal dominant optic atrophy using AI-segmented OCT and visual field metrics	Vasileios Batis, Mehdi Ounissi.Elina Priquet, Ana Banc, Catherine Vignal-Clermont, Dan Milea	13
24	Visual prognosis in late-onset Leber hereditary optic neuropathy in a large Italian cohort	Marco Battista, Michele Carbonelli, Luigi Brotto, Giulia Amore, Catarina Coutinho, Martina Romagnoli, Alice Galzignato, Paulo Nucci, Leonardo Caporali, Claudio Fiorini, Chiara La Morgia, Valerio Carelli, Francesco Bandello, Piero Barboni	14
25	Analysis of parent-of-origin effects on clinical parameters in *OPA1* autosomal dominant optic atrophy	Berthold Pemp, Johannes Schrittwieser, Karl Kircher, Martin Bertich, Christoph Stapf, Reginald E. Bittner, Wolfgang M. Schmidt, Andreas Reitner	15
26	Retinal hyperreflective foci: A new potential biomarker in optic neuritis and multiple sclerosis	Betul Okutan, Michael Larsen, Jette Lautrup Frederiksen	15
30	Macular edema secondary to rozanolixizumab: A case report	Bernardo Sánchez Dalmau, Núria Domènech López, Pau Otal Aran, Diego Santolaria, Rafael Alcubierre Bailac, Covadonga Menéndez Acebal, Anna Camós Carreras, M. Sepúlveda	16
31	Optic neuritis: A single-centre case series	Alibhe Burke, Lola Ogunbowale	17
34	Retinal imaging features as diagnostic biomarkers in ARSACS	Maria Lucia Cascavilla, Antonio De Santis, Lorenzo Bianco, Maurizio Battaglia Parodi	17
36	Ocular syphilis with optic nerve involvement: A neuro-ophthalmologic case series from a tertiary center	Celso Costa, Telma Machado, Miguel Raimundo, Rosa Sá, Fernando Rodrigues, Cristina Fonseca, Pedro Fonseca	18
37	Neuro-ophthalmological findings in a Brazilian cohort of patients with Wolfram syndrome treated with idebenone compared with untreated controls	Filipe Chicani	19
38	Prospective study of retinal diffusion-weighted imaging in central retinal artery occlusion	Charlotte Pietrock, Milena Miszczuk, Eberhard Siebert, Leon Alexander Danyel	20
39	Recurrent missense variant in PPIB gene is associated with isolated late-onset dominant optic atrophy in Italian cohort	Chiara La Morgia, Claudio Fiorini, Alberto Pietro Pasti, Danara Ormanbekova, Piero Barboni, Michele Carbonelli, Giulia Amore, Marco Battista, Luigi Brotto, Paolo Nucci, Leonardo Caporali, Valerio Carelli	21
40	Retinal microRNA dynamics underlying the pathophysiology of traumatic optic neuropathy and the therapeutic effect exerted by an E3 ubiquitin ligase inhibitor	Ching-Lung Chen, Ching-Yun Wang, Shun-Ping Huang	22
42	Neuro-structural involvement of optic pathways in adult neurofibromatosis typw 1: A comparative study of Optical Coherence Tomography (OCT) and Magnetic Resonance Imaging (MRI)	Claudia Mainetti, Claudia Cesaretti, Marco Nassisi, Laura Dell’Arti, Cecilia Serra, Alessandro Draghi, Federica Natacci, Francesco Viola	23
43	Monoallelic variants in *UCHL1* are an emerging cause of autosomal dominant optic atrophy (ADOA)	Claudio Fiorini, Giada Capirossi, Chiara La Morgia, Xavier Dieu, Federico Sadun, Maria Lucia Cascavilla, Marco Battista, Lorenzo Bianco, Piero Barboni, Elisabetta Racano, Fiorenza Soli, Enza Maria Valente, Ilaria Palmieri, Neringa Jurkute, John Britton, Patrick Yu-Wai-Man, Danara Ormanbekova, Flavia Palombo, Eleonora Pizzi, Valentina Del Dotto,Valerio Carelli, Alessandra Maresca, Patrizia Amati-Bonneau, Leonardo Caporali	24
44	An unusual case of myelin oligodendrocyte glycoprotein optic neuritis with disc edema and prepapillary infiltration	Catharina Mönig, Walid Bouthour	25
46	A not-so-luxurious darkness	Covadonga Menendez Acebal, Elena Brotons Muñoz, Pau Marjalizo, Pau Otal Aran, Rafel Alcubierre Bailac, Anna Camos Carreras, Bernardo Sánchez Dalmau	26
47	Lenadogene nolparvovec gene therapy for lever hereditary optic neuropathy in the real-life setting	Catherine Vignal-Clermont, Chiara La Morgia, Valerio Carelli, Patrick Yu-Wai-Man, Mark L. Moster, Robert C. Sergott, Sarah Thornton, Sean P. Donahue, Hélène Dollfus, Thomas Klopstock, Claudia Priglinger, Rabih Hage, Vasily Smirnov, Catherine Cochard, Marie-Bénédicte Rougier, Emilie Tournaire-Marques, Pierre Lebranchu, Caroline Froment, Frédéric Pollet-Villard, Marie-Alice Laville, Claudia Prospero Ponce, Scott D. Walter, Francis Munier, Pauline Zoppe, Magali Taiel, José-Alain Sahel	26
48	Utility of propensity score weighting to improve external comparator matching in the LEROS study of idebenone efficacy in Lever hereditary optic neuropathy	Daniela Garavaglia, Nancy J. Newman, Patrick Yu-Wai-Man, Alfredo A. Sadun, Valerio Carelli, Rod Gossen, Karen Sison Lance, Caroline Fradette, Xavier Llòria, Thomas Klopstock	28
49	A retrospective longitudinal cohort study describing visual outcomes in patients with paediatric optic nerve gliomas and identifying predictors of poor visual outcomes.	Divya Agrawal, Emma Kerr, Brinda Muthusamy	29
51	Beyond peripheral nerves: Bilateral optic neuropathy in Charcot-MarieTooth type 2A2	Daniel Cardoso, Lígia Ribeiro, Dália Meira	30
52	Isolated optic neuropathy in Bosch–Boonstra–Schaaf syndrome due to a novel NR2F1 variant	David Alves, Ana Faria-Pereira, Olinda Faria, Sérgio Estrela Silva	30
54	Unilateral optic neuropathy resulting from vitreopapillary traction syndrome with concurrent epiretinal membrane	Danijel Pericic, Ari August, Andrew Garfield	31
55	Rarity within rarity: Unilateral Leber’s hereditary optic neuropathy	Michele Carbonelli, Anna Maria De Negri, Stefania Bianchi Marzoli, Giulia Amore, Piero Barboni, Marco Battista, Luigi Brotto, Catarina P. Coutinho, Martina Romagnoli, Valerio Carelli, Chiara La Morgia	32
58	I am not confused, I am just well mixed- the jar with double-negative (DN) optic neuritis	Durga Priyadarshini Santhakumar, Ambika Selvakumar, Shikha Bassi Talwar	33
67	Pediatric autosomal recessive LHON: A report of two cases	Eva Roomets	34
68	Optic disc edema in juvenile macular dystrophy	Francesca Allegrini	35
69	Silent retinal neurodegeneration in multiple sclerosis: Minimum rim width (MRW) as a possible early marker of progressive neuronal loss	Francesca Pilati, Francesca Bosello, Rocco Micciolo, Emilio Pedrotti, Chiara Zaffalon, Damiano Marastoni, Polinelli, Matilde Zambon	35
70	Functional–structural dissociation in hereditary optic neuropathies	Gabriella Cammarata, Lisa Melzi, Diletta Santarsiero, Alessandra Criscuoli, Virginia Raccagni, Stefania Bianchi Marzoli	36
71	The fairytale of the sight-saving cat and virus	Gabriella Szatmary, Siena Dhillon	37
73	Mitochondrial optic neuropathies: The shifting clinical trial landscape	Grace A. Borchert, Megan F. Baxter	38
74	OCT RNFL sector-specific analysis for papilloedema detection: PHOMS coexistence in 41% of cases does not impair diagnostic performance	Guillaume Mignot, Amna Irdan, Ramith Ginige, Shivona Chetty	39
81	Virus antibody responses in patients with ON as part of MOGAD versus RRMS	Jette Lautrup Frederiksen, Nicole Hartwig Trier, Rasa Petraitytė-Burneikienė, DanguolėŽiogienė, Rimantas Slibinskas, Evaldas Ciplys, Gunnar Houen	40
82	Idiopathic unilateral optic disc edema in a young man: A diagnostic challenge	Joana da Silva Rocha, Ana Rita Viana, Alberto Lemos	41
84	Inner retinal layer thinning, age and genetic variant class influence visual function in OPA1 autosomal dominant optic atrophy	Johannes Schrittwieser, Andreas Reitner, Karl Kircher, Martin Bertich, Christoph Stapf, Stephanie Lilja, Amina Paquay, Gregor S. Reiter, Reginald E. Bittner, Wolfgang M. Schmidt, Berthold Pemp	42
89	Late-onset Leber hereditary opyic neuropathy: A case series of 16 patients diagnosed above 65 years of age	Putcuijps Kato1, Lushchyk Tanya, Cassiman Catherine, Van Everdingen Judith	43
92	Reduction in retinal ganglion cell layer and macular nerve fiber layer loss with privosegtor in acute optic neuritis: Post-hoc analysis of OCT scans from the phase 2 ACUITY study	Leila S. Eppenberger, Martin S. Zinkernagel, Céline Louapre, Sophie Bonnin, Louise-Laure Mariani, Mikael Cohen, Caroline Froment Tilikete, Mark Kupersmith, Leonard A. Levin, Sabri Markabi, Caroline Papeix, Valérie Touitou, Rossella Medori, Arshad M. Khanani, Pablo Villoslada, Sebastian Wolf	44
93	Long-term OCT and visual outcomes after hypofractionated radiosurgery for optic nerve sheath meningioma: 48-month monocentric prospective study	Ludovica Gargiulo, Marcello Marchetti, Gabriella Cammarata, Paola Ciasca, Alberto Raggi, Giorgia Camarda, Sara Morlino, Laura Fariselli, Stefania Bianchi Marzoli	45
95	Migraine-related retinal arterial occlusion: Two cases supporting a vasospastic mechanism	Laura Sánchez Vela, Laura Vigués, Ruth Martín	46
100	Recessive variants in mitochondrial Complex I nuclear subunits are an underrated cause of optic atrophy	Claudio Fiorini, Neringa Jurkute, Alessandra Torraco, Chiara La Morgia, Daniele Ghezzi, Gaia Tioli, Laura Rigobello, Danara Ormanbekova, Alessandro Berghella, Alberto Pietro Pasti, Flavia Palombo, Piero Barboni, Maria Lucia Cascavilla, Federico Sadun, Annamaria De Negri, Enrico Bertini, Olimpia Musumeci, Anna Ardissone, Teresa Rizza, Giancarlo Iarossi, Gabriella Silvestri, Salvatore Rossi, Anastasia Altobelli, Antony T. Moore, Thomas Cullup, Andrew R Webster, Indran Davagnanam, Michel Michaelides, Samantha Malka, Hana Ptackova, Hana Stufkova, Marketa Tesarova, Petra Liskova, Leopold Zeng, Thomas Klopstock, Robert Kopajtich, Christiane Neuhofer, Holger Prokisch, Costanza Lamperti, Alfredo A. Sadun, Patrick Yu-Wai-Man, Valerio Carelli, Francesco Musiani, Luisa Iommarini, Rosalba Carrozzo, Gavin Arno, Leonardo Caporali	47
101	Comparative OCT neuroretinal profiles in acute LHON and atypical optic neuritis	Leonie F Keidel, Alexander Kufner, Jonathan Gernert, Jana Mattar, Patrick Yu-Wai Man, Claudia Priglinger, Bettina von Livonius, Thomas Klopstock, Linus Keidel, Siegfried G Priglinger, Joachim Havla, Neringa Jurkute	48
102	Adalimumab as a novel potential risk factor for Posterior Ischaemic Optic Neuropathy	Luca Seregni, Antonio Calcagni, Ailbhe Burke	49
104	Improvement of diabetic papillopathy with intravitreal anti-vascular endothelial growth factor: A case report	Mambretti Manuela, Bartoccini Matilde, Covelli Antonio, Radice Paolo	50
108	Assessment and quantification of RAPD using slit lamp	Marko Hawlina	51
109	Discovery of PHB1 as a novel candidate gene in dominant optic atrophy	Martina Jarc Vidmar, Ales Maver, Marko Hawlina, Borut Peterlin, Ana Fakin, Lea Kovac, Maja Sustar Habjan, Lucija Malinar, Sanja Petrovic Pajic, Tanja Visnjar, Nusa Trost, Rok Romih, Urska Dragin	51
113	Gross bilateral optic disc swelling with immune checkpoint inhibitor use in oesophageal adenocarcinoma: Leptomeningeal spread vs immune-related neurotoxicity?	Matthew Mo, Guillaume Mignot, Amna Irfan	52
115	Optic disc edema in APMPPE	Michele Fantini	53
118	Pediatric Myelin Oligodendrocyte Glycoprotein associated optic neuritis presenting with pre-retinal haemorrhage and raised intracranial pressure	Marco Piergentili, Vasiliki Panteli, Cheryl Hemingway, Richard Bowman	54
119	A recurrence after decades of silence	Mubarika Sami Sami	55
120	An unusual painful optic neuropathy in severe axial myopia mimicking optic neuritis	Kevin Muggler, Tim Beltraminelli, Aki Kawasaki	56
121	OCT device agreement is parameter-specific and disease-dependent in optic disc drusen	Christoph Mits, Karl Kircher, Elena, Terekhova, Berthold Pemp	57
124	When acute vision loss is not giant cell arteritis: A case of fungal sinusitis affecting the orbital apex	Nuala Pepper, Emily Stedman, Ruth Batty, Thomas Green	57
125	Sequential optic nerve injury in MOG antibody–associated optic neuritis: A mixed optic neuropathy	Nuno Álvaro Silva, Ricardo Reis, Olinda Faria, Ana Faria Pereira	58
126	Positive 5-year benefit/risk ratio of bilateral injection of Lenadogene nolparvovec gene therapy for Leber hereditary optic neuropathy	Nancy J. Newman, Patrick Yu-Wai-Man, Prem S. Subramanian, Sarah Thornton, An-Guor Wang, Sean P. Donahue, Bart P. Leroy, Valerio Carelli, Valérie Biousse, Catherine Vignal-Clermont, Alfredo A. Sadun, Robert C. Sergott, Gema Rebolleda Fernández, Bart K. Chwalisz, Rudrani Banik, Magali Taiel, José-Alain Sahel, for the LHON REFLECT Study Group.	59
127	FALCON natural history study: Identifying sensitive measures of changein OPA1-associated ADOA	Piero Barboni, Marc Bouffard, Julie Falardeau, Chiara La Morgia, Byron Lam, Michael Larsen, Yaping Joyce Liao, Raghu Mudumbai, Marcela Votruba, Patrick Yu-Wai-Man, Alice Wyse Jackson, Kelly Saluti, Charles Liu, Barry Ticho, Steven Gross	60
128	Peripapillary hyperreflective ovoid mass-like structures in a myopic adolescent mimicking papilledema	P. Giannopoulos, W.A.J. Birkhoff, G. Thepass, J.A.M. van Everdingen, T. Lushchyk	62
129	When flow goes wrong, the eyes speak strong: Case report of advanced ocular ischemic syndrome	Patrycja Duda, Iwona Grabska-Liberek	63
131	Optic disc swelling in emergency ophthalmology: Diagnostic and management pathways.	Joanna Przybek-Skrzypecka, Dominik Sokalski, Jan Bombuy Gimenez, Janusz Skrzypecki, Jacek P Szaflik, Wolf Lagrèze	63
132	OSPREY: A Phase 1, open-label study evaluating the safety and tolerability of the antisense oligonucleotide STK-002 in patients with OPA1 autosomal dominant optic atrophy	Patrick Yu-Wai-Man, Piero Barboni, Michael Larsen, Lyubomyr Lytvynchuk, Berthold Pemp, Katarina Stingl, Marcela Votruba, Karen Anderson,Jeff Hoger, Alice Wyse Jackson, Kelly Saluti, Barry Ticho, Steven Gross	64
134	Atypical cytomegalovirus optic neuritis in an immunocompetent patient: diagnostic challenges and therapeutic response	Radina Kirkova	65
135	Severe pain as an indicator of malignancy in patient with optic neuropathy	Rossul Al-Bahadili, Amarah Kiani, Anastasia Pilat	66
136	Clinical outcomes following long-term treatment with idebenone in patients with Leber’s hereditary optic neuropathy	Raluca Iorga, Andreea Moraru, Răzvana Munteanu-Dănulescu, Ciprian Danielescu	67
137	A unified structural and functional framework for visualizing and quantifying the spatial pattern of retinal neuron loss and tracking progression in optic neuropathies	Randy Kardon, Mona Garvin, Jui Kai Wang, Edward Linton, Young Kwon	68
138	Rarefaction of the deep retinal vascular plexus associated with reduced visual acuity in Fabry disease	Rebecca Wicklein, Claudia Regenbogen, Timon Kuchler, Elisabeth Wolf, Johanna Niederschweiberer, Bernhard Hemmer, Christoph Schmaderer, Marcus Deschauer, Benjamin Knier	69
139	The effect of disease-modifying therapy on retinal thinning in RRMS	Sanela Sanja Burgic, Mirko Resan, Zoran Vukojevic, Daliborka Tadic	70
140	Asian Optic Neuritis Study (AONS)–Preliminary results	Reuben Foo	71
141	Transient visual obscurations as the first sign of isolated leukemic optic nerve infiltration	Ruth Martin Pujol, Laura Vigués Jorba, Laura Sánchez Vela	72
146	Clinical-genetic analysis in Bulgarian patients with Leber’s hereditary optic neuropathy	S. Cherninkova, B. Zaharova, K. Kamenarova, K. Mihova, S. Atemin, T. Todorov, V. Haikin, A. Oscar, I. Tournev, R. Kaneva, A. Todorova	73
147	Arteritic anterior ischaemic optic neuropathy (AAION) without disc swelling and normal inflammatory markers	Samantha Siaw Zhen Sii, Shery Thomas, Rupa Patel	74
151	High-dose glucocorticosteroids and neuroprotection in acute optic neuritis: A multicenter retrospective cohort study	Sebastian Küchlin, Jörg Sahlmann, Wolf Lagrèze	75
153	Ethambutol optic neuropathy (EON): Mechanistic insights from proteomic analysis of a stem cell derived retinal ganglion cell model of EON	Shweta Singhal, Rayhan Al Awdady Bte Ismail, Muhammad Ikhsan Bin Muzakar, Zhou Lei	76
154	Optic nerve biopsy: When and why?	Silvia Muñoz, Eugènia Moix, Maravillas Abia	77
155	Non-invasive biomarkers for the differential diagnosis of acute anterior ischemic optic neuropathy in an emergency setting	Stefano Erba, Laura Fumagalli, Enrico Tombetti, Stefano De Angelis	78
156	Optic nerve and retinal ganglion cell changes following semaglutide exposure in healthy mice	Talal Salti, Katia Marzuq, Nitza Goldenberg-Cohen	79
157	Peripapillary Hyperreflective Ovoid Mass Like Structure (PHOMS): A nonspecific OCT marker in papilledema, pseudo disc edema and disc edema	Tapi Yalu, Swati Aalok Phuljhele, Rohit Saxena, Rebika Dhiman	79
160	Informative censoring in natural history control cohort influences the interpretation of the long-term efficacy of idebenone in treating visual impairment from Leber hereditary optic neuropathy	Thomas Klopstock, Alfredo A. Sadun, Nancy J. Newman, Patrick Yu-Wai-Man, Rod Gossen, Daniela Garavaglia, Karen Sison Lance, Caroline Fradette, Xavier Llòria, Valerio Carelli	80
161	The MOGnitude of NAION	Tim Beltraminelli, Kevin Müggler, Aki Kawasaki	81
162	A family affair….	Salvatore Sottosanti	82
163	Neuroprotective effects of Intravitreal Injections of Umbilical Cord Mesenchymal Stem Cell-Derived Exosomes in rats with traumatic optic neuropathy (TON)	Rong-Kung Tsai, Ruksana Akter Ruma, Tu-Wen Chen	83
164	Nonarteritic anterior ischemic optic neuropathy following sclerectomy for uveal effusion in nanophthalmos: A case report	Wei-Che Hung, Hui-Chen Cheng, An-Guor Wang, Shih-Jen Chen	84
165	Indirect comparison of lenadogene nolparvovec gene therapy versus Natural History In Patients with m.11778G>A MT-ND4 Leber Hereditary Optic Neuropathy Over 5 Years	Valérie Biousse, Patrick Yu-Wai-Man, Nancy J. Newman, Mark L. Moster, Valerio Carelli, Prem S. Subramanian, Catherine Vignal-Clermont, An-Guor Wang, Sean P. Donahue, Bart P. Leroy, Robert C. Sergott, Thomas Klopstock, Alfredo A. Sadun, Gema Rebolleda Fernández, Bart K. Chwalisz, Rudrani Banik, Magali Taiel, and José-Alain Sahel, for the LHON Study Group	85
168	Enhanced detection of treatment benefit using a novel composite efficacy endpoint to evaluate the visual impact of idebenone in subacute/dynamic and chronic patients with Leber hereditary optic neuropathy: Post-hoc analysis of the LEROS study	Xavier Llòria, Nancy J. Newman, Patrick Yu-Wai-Man, Alfredo A. Sadun, Valerio Carelli, Rod Gossen, Daniela Garavaglia, Karen Sison Lance, Caroline Fradette, Thomas Klopstock	86
169	Changepoint timing of retinal thinning after optic neuritis and its clinical implications	Yeji Moon, Byung Joo Lee, Eun-Jae Lee	87
171	Structural and functional changes in retina and optic nerve head in NAAION	Lepša Žorić	88
9	Repetitive transcranial magnetic stimulation for visual snow syndrome: symptoms relief and changes in functional brain dynamics. A case report.	Alessia Bargagli, Francesco Neri, David De Monte, Alberto Benelli	89
18	Charles Bonnet syndrome: Service audit of 160 patients (2017–2025) and management pathway update	Anna Visca, Nima Ghadiri	90
123	Vitamin deficiency in patients with visual snow syndrome	Nelli Galoyan	90
142	On the right track(t)	Rogan G Fraser, Musa China, Patrick Grover, Chandrashekar Hoskote, Eoin Finegan, Neringa Jurkute	91
13	Case report of papilledema associated with meningioma	Ana Srdoč Grbac, Maja Mrak, Tea Majnarić, Tamara Mišljenović Vučerić, Tea Čaljkušić-Mance	92
14	Visual risk-stratification and management in Idiopathic Intracranial Hypertension	Ana Belén Tárraga Ibáñez, Sara Khanji-Fustek, Teresa Albert-Albelda, Ángeles Bort-Marti	93
21	The role of transorbital ultrasonography and optical coherence tomography in the follow-up of idiopathic intracranial hypertension	Ayşe Kurt, Mert Göbel, Hülya Ertaşoğlu Toydemir, Hacı Ali Erdoğan, İbrahim Acır, Aslı Vural, Vildan Yayla	94
28	Papilledema quantification with OCT: An unsupervised Deep Learning Algorithm outperforms the Heidelberg commercial software	Bilal Omari, Mehdi Ounissi, Ana Banc, Dan Milea	95
45	Recurrent idiopathic intracranial hypertension following unilateral transverse venous sinus stenting	Casandra MacLeod, M. Shazam Hussain, Devon A. Cohen	96
59	The cautious seldom err!!!–pediatric idiopathic intracranial hypertensionour experience	Vidhya Dharani, Ambika Selvakumar	96
60	Differentiate with value or die with price!!–Risk factor analysis of visual outcomes of papilledema following ONSF in typical versus atypical IIH	Selvakumar Ambika, Padmalakshmi Krishnakumar	97
62	Asymptomatic bilateral optic disc edema in an 18-Year-Old myopic patient: Radiologic evidence of incidental diagnosis of idiopathic intracranial hypertension	Dudău Maria, Vlad Octavian Bolocan, Alexandru Dimancea, Silviu Dumitrescu	98
88	Early mortality for individuals with idiopathic intracranial hypertension	Soule Digre, C. O’Neill, J. Shaia, M. Newman, J.E.A. Warner	99
99	Neuroimaging outcomes in suspected papilledema	Leon Alexander Danyel	100
105	When BMI misleads: idiopathic intracranial hypertension in a non-obese Filipina but with recent rapid weight gain	Elinor Angelie M. Perez, Marianne Roque. Carabeo	101
106	Multidisciplinary management of pediatric papilledema using lumboperitoneal shunting	Mariantonietta Di Brina, Giacomo Maria Bacci, Barbara Spacca, Regina Mura, Antonio Laborante	102
110	Optical coherence tomography detection of shunt malfunction and recurrent raised intracranial pressure in an asymptomatic child with bilateral optic atrophy	Martina Suzani, Andrea Trezza, Giorgio Carrabba, Carlo Giussani, Michele Coppola	103
116	What are the benefits of using acetazolamide in Idiopathic Intracranial Hypertension? Insights from a subanalysis of the IIH:Weight Trial	Michael Lowe, Yousef F Hyder, Andreas Yiangou, James Mitchell, Kristian Brock, Susan P. Mollan, Alexandra J. Sinclair	104
133	What is vision threatening papilloedema?	Rebecca Rewbury, Palaniappan Muthiah, Irene Pepper, Simon Hickman, Joanna Jefferis	105
148	Ultrasound measurement of optic nerve sheath diameter for the purpose of evaluating elevated intracranial pressure with or without papilledema–Case series	Tamara Mišljenović Vučerić, Ana Srdoč, Tomislav Vidović, Jelena Metikoš, Tea Čaljkušić Mance	106
149	Effect of semaglutide and hypocaloric diet on visual outcomes in idiopathic intracranial hypertension: A longitudinal randomized study	Sanja Cejvanovic, Nadja Skadkær Hansen, Elisabeth Arnberg Wibroe, Dagmar Beier, Rigmor Højland Jensen, Steffen Hamann	106
159	Misdiagnosis in suspected idiopathic intracranial hypertension in Germany	Theresia Knoche	107
167	Intracranial hypertension associated with Terson’s syndrome after epidural blood patch: A case report	Vanessa Takou Tsapmene, Rim Maalej, Ana Banc, Dan Milea	108
170	Neuro-ophthalmological signs of intracranial hypertension in patients undergoing surgical closure of spontaneous cerebrospinal fluid leak	Youri Ducluzeau, Caroline Froment, Catherine Vignal	109
4	Pilot data of smooth pursuit measurement for multiple sclerosis patients with bulbicam infrared camera	Adriana Zelníková, Dominik Foltán, Marie Soukupová, Jakub Potměšil, Jana Lízrová Preiningerová	110
11	Two new forms of hemi-seesaw nystagmus: Associated with homonymous hemianopia and paroxysmal with infratentorial graviceptive pathway sparing	Alexandre Roldão Alferes, Daniela Pereira, Catarina Rato, Gonçalo Costa, João Nabais, Armando Lopes, Ana Inês Martins, André Jorge, João Lemos	111
16	The natural course and prognostic factors of microvascular ocular motor palsies	André Sobral-Pinho, André Costa, Ana Inês Martins, Daniela Pereira, André Jorge, João Lemos	112
19	Strabismus surgery is an effective option for patients with abducens palsy caused by neurovascular conflict	Annabel Groot	113
20	Diagnosis and management of myasthenia in a neuro-ophthalmology service	Ayman Khaier, Muzzammil Hussain	113
41	Topical neostigmine ophthalmic solution for alleviating myasthenic ptosis: A phase 1 dose-finding study	Chuthamas Ongprakobkul, Supanut Apinyawasisuk, Supharat Jariyakosol, Pajaree Chariyavilaskul, Kornvalee Meesilpavikkai, Yuda Chongpison	114
66	Facial neuropathy in patients with recurrent painful ophthalmoplegic neuropathy	Jae-Myung Kim, Kyung Wook Kang, Kang-Ho Choi, Dong Uk Kim, Seung-Han Lee	115
77	Peculiarities of stereoacuity and eye micromovements during a static f ixation task in children aged 6 to 13 years with retinopathy of prematurity after laser photocoagulation and without it	Iryna Boichuk, Oleksandr Artamonov, Serhiy Katsan	115
87	Saccadic dysmetria and delayed latency induced by severeglobe deformity following encircling scleral buckling: a correlation of mri and videooculography findings	Ileok Jung	116
96	Bilateral third nerve palsy as the initial manifestation of disseminated tuberculosis with central nervous system involvement	Laura Vigués-Jorba, Ruth Martin Pujol, Laura Sanchez Vela	117
107	Occam meets hickam: Dual genetic diagnoses in a patient with ataxia and preserved vision	Marius Bertram Maartensson, Dina Abdelsalam, Safa Ibrahim, Andrew Go Lee	118
111	Ocular motor characteristics of infantile nystagmus syndrome in clinical practice using standardized eye movement recordings	Colin Colmant, Ana Coito, Mathias Abegg	119
112	Acquired fourth nerve palsy after cervical spinal manupulation: A case report	Matteo Baldi, Michele Iester, Aldo Vagge	120
117	Unilateral third cranial nerve palsy as a complication of MADSAM: A case report	Maja Kostic, Luciana Garcia, Michael Miltich, Elizabeth Colvin	121
130	A sixth nerve palsy with too many friends	Misha Pless, Manisha Kotay, Eric Eggenberger	122
144	Marcus-Gunn jaw winking ptosis– One can only laugh for so long …	Grzegorz Rotuski	123
158	Positional opsoclonus in ataxia telangiectasia-like disorder type 1	Teresa Santana, Francisca Ferreira, Ana Inês Martins, João Durães, André Jorge, João Lemos	123
32	Neuro-ophthalmic manifestations of systemic lupus erythematosus: Spectrum, mechanisms, and management	Matteo Capobianco, Caterina Gagliano, Mark Zeppieri	124
50	VEXAS– The neuro-ophthalmologic chameleon syndrome, a case report.	Daniela Suter-Starosta	125
61	Immune-mediated multisystem disease mimicking Vogt–Koyanagi-Harada: Bilateral vision loss with granulomatous uveitis, cerebrovascular infarctions, and arrhythmia revealing hodgkin lymphoma	Tugce Kizilay, Rabia Karabiyik, Medine Asli Yildirim, Kiymet Kasapoglu, Nurhan Kaya, Nilufer Kale	126
63	Orbital Inflammation in a Patient with Common Variable Immunodeficiency: A Case Report	Elisa Salamin, Aki Kawasaki	127
64	Functional in vivo confocal microscopy reveals corneal immune cells as peripheral biomarkers of neurodegenerative disease	Emma Grace Oreskovic	128
65	Syphilis resurgence: Neuro-ophthalmic manifestations	Emma Spowart, Aashna Jain, Sinéad Connolly, Sarah Walpole, Ashley Price, Ewan Hunter, Ranjit Pandit	128
94	Visual field defects in posterior cortical atrophy patients are not associated with retinal ganglion cell loss	Laura Dell’Arti, Claudia Mainetti, Giorgio Fumagalli, Francesco Bordoni, Marco Nassisi, Chiara Fornari, Francesco Viola	129
98	Giant cell arteritis in overdrive: Increased incidence and severity of ocular involvement and the role of early tocilizumab use	Lea Kovac, Ana Fakin, Marko Hawlina	130
6	Prospective AI-based fundus image identification of neuro-ophthalmic disorders in a tertiary emergency department	Ayse Gungor, Ana Banc, Samy Zaher, Ilias Sarbout, Stefana Croitoru, Ruth Huna-Baron, Bouchra Touzani, Cedric Lamirel, Daniel Racoceanu, François Audren, Pearse A. Keane, Dan Milea	131
17	How much data is enough data in visual field analysis?	Angelica Simonetti, Catarina P. Coutinho, Ferdinando Zanchetta, Michele Carbonelli, Marco Battista, Alice Galzignato, Chiara La Morgia, Giulia Amore, Martina Romagnoli, Luigi Brotto, Paolo Nucci, Maria Lucia Cascavilla, Claudio Fiorini, Leonardo Caporali, Francesco Bandello, Valerio Carelli, Rita Fioresi, Piero Barboni	132
35	Visual field transfer learning: from glaucoma to hereditary optic neuropathies	Catarina P. Coutinho, Ferdinando Zanchetta, Angelica Simonetti, Michele Carbonelli, Marco Battista, Alice Galzignato, Chiara La Morgia, Giulia Amore, Martina Romagnoli, Luigi Brotto, Paolo Nucci, Maria Lucia Cascavilla, Claudio Fiorini, Leonardo Caporali, Francesco Bandello, Valerio Carelli, Rita Fioresi, Piero Barboni	133
90	Artificial intelligence-based classification of Parkinson’s disease using virtual reality-derived oculometrics	Erika Han, Ana Coito, Hassan Fadavi, Bruno Hauser, Pia Massatsch, Valentina Stozitzky, Dongli Li, Bettina Balint, Helen Dawes, Konrad P. Weber	134
8	When neuro-ophthalmic disease signals hidden systemic pathology: Lessons from three contrasting cases	Alaeldin Nour, Nima Ghadiri, Anna Visca	135
27	How to diagnose cerebral visual impairment (CVI): Recommendations for clinical practice	Bianca Huurneman, Jannet Philip, Nomdo M. Jansonius, Frounke Nienke Boonstra	136
29	Binocular pupillography with virtual neutral density filtering detects early afferent gain	Bjørn André Helland-Hansen, Goran Petrovski, Alexander Sverstad, Stig Larsen	137
33	The neck mass behind Horner syndrome: A case of cervical paraganglioma	Carlos Leis-Cofiño, María Castro Rebollo	138
53	Movement and sight: A case of type 7 spinocerebellar ataxia diagnosed in a small regional hospital	Diana Rotaru, Ion Rotaru	139
56	Triangulating relative afferent pupillary defect assessment: Functional, structural and clinical perspectives	Dominik Brügger, Melina Katsimpoura, Julian Lehmann, Bruno Hauser, Emilie Reuter, Justus Luerweg, Johanna Haselon, David Bratek, Ralf Gold, Jeremias Motte, Ruth Schneider, Anke Salmen	140
72	Slowly progressive Leigh Syndrome associated with NDUFAF3 Mutation	Alice Galzignato, Anna Ardissone, Marco Battista, Leonardo Caporali, Claudio Fiorini, Luigi Brotto, Paolo Nucci, Valerio Carelli, Piero Barboni	141
75	An unresolved case of polyradiculitis and retinal atrophy	Virginie Buehler, Sabine Fenzl, Florian Heussen, and Hilary M. Grabe	142
76	Ophthalmological findings in children with a primary brain tumor: The visual function up to two years after diagnosis	IngeJ.M.Corver, Giorgio L. Porro, Inge Stegeman, AntoinetteY. N.Schouten-van Meeteren, Saskia M. Imhof	143
78	The obesity paradox in korean patients with diabetes: An inverse relationship between higher body mass index and the risk of non-arteritic anterior ischemic optic neuropathy	Jaeryung Kim, Kyungdo Han, Sei Yeul Oh, Kyung-Ah Park	144
79	A multicenter analysis of the clinical spectrum of anti-GQ1b antibody syndrome	Jasmine Yaowei Ge, Wang Yu Hang, Umapathi N Thirugnanam, Loo Jing Liang, Christen Lim LS	145
83	Bilateral optic neuritis and transverse myelitis with overlapping MOG-IgG and Lyme seropositivity	João Mendes	146
85	A collaborative approach to chronic dizziness or persistent posturalperceptual dizziness (3PD) and vestibular migraine: The Utah chronic dizziness collaborative (UCDC) and dizzy school	Judith Warner, Kathleen Digre, Sravanthi Vegunta, Meagan Seay, Janene Holmberg	147
86	Dural veinous thrombosis case series	Karima Nesnas, Sneha Melmane, Thushara Mannampat	148
91	Reality check: Virtual tools in functional vision disorders Functional and structural visual loss: Insights from virtual reality perimetry in f ive complex cases	Manisha Kotay, Eric Eggenberger	148
97	When vision affects driving: Healthcare professionals’ approaches to discussing fitness to drive	L. R. Hepworth, J. Wyche, C. Howard	151
103	Post-retinal visual pathway abnormalities and visual function in Friedreich Ataxia	Lucilla Barbano, Lucia Ziccardi, Carmen Dell’Aquila, Mattia D’Andrea, Antonio Di Renzo, Gaspare Colacino, Carlo Casali, Vincenzo Parisi	152
114	Age-related changes in the biomechanical properties of the human optic nerve sheath	Michael Dattilo, Anna Silverio, Dennis Jiang, Andrew Feola	153
143	European Stroke Organisation vision guidelines. The recommendations for assessment and rehabilitation for visual disorders	Fiona Rowe, Lauren Hepworth, Maria Begona Coco-Martin, Celine Gillebert, Luis Leal-Vega, Anja Palmowski Wolfe, Eleni Papageorgiou, Stephen Ryan, Karolina Skorkovska, Anne Hege Aamodt	154
145	Temporal bioavailability of intranasal recombinant human nerve growth factor in ocular and neural tissues in mice	Rubina Novelli, Annamaria Cimini, Laura Brandolini, Andrea Aramini, Marcello Allegretti	155
150	A severe case of Tolosa-Hunt Syndrome: Differential diagnosis and review of current treatment options.	Sarah Marti, Konrad P. Weber	156
152	The ultrasound dopplerography in mixed astigmatism arising against the background of central nervous system pathologies	Sevinj Salmanova, Elmar Kasimov, Saida Gadzhiyeva, Afet Mammadzada	156
166	Acute Horner syndrome identified by pupillography revealing internal carotid artery dissection	Victoria Borisova, Muriel Dysli	158
